# A protein–miRNA biomic analysis approach to explore neuroprotective potential of nobiletin in human neural progenitor cells (hNPCs)

**DOI:** 10.3389/fphar.2024.1343569

**Published:** 2024-01-25

**Authors:** Sadaf Jahan, Uzair Ahmad Ansari, Ankur Kumar Srivastava, Sahar Aldosari, Nessrin Ghazi Alabdallat, Arif Jamal Siddiqui, Andleeb Khan, Hind Muteb Albadrani, Sana Sarkar, Bushra Khan, Mohd Adnan, Aditya Bhushan Pant

**Affiliations:** ^1^ Department of Medical Laboratory Sciences, College of Applied Medical Sciences, Majmaah University, Majmaah, 11952, Saudi Arabia; ^2^ Health and Basic Sciences Research Center, Majmaah University, 11952 Majmaah, Saudi Arabia; ^3^ Developmental Toxicology Laboratory, Systems Toxicology Group, CSIR-Indian Institute of Toxicology Research (CSIR-IITR), Vishvigyan Bhavan, 31, Mahatma Gandhi Marg, P.O. Box No. 80, Lucknow 226001, Uttar Pradesh, India; ^4^ Academy of Scientific and Innovative Research (AcSIR), Ghaziabad 201002, India; ^5^ Department of Biology, College of Science, University of Hail, Hail, Saudi Arabia; ^6^ Department of Biosciences, Faculty of Science, Integral University, Lucknow, Uttar Pradesh 226026, India; ^7^ Department of Clinical Laboratory Sciences, College of Applied Medical Sciences, Imam Abdulrahman Bin Faisal University, Dammam, Eastern Province 34212, Saudi Arabia

**Keywords:** sodium arsenate, nobiletin, human neural progenitor cells, miRNA, proteomics, neurodegeneration, neuroprotection

## Abstract

Chemical-induced neurotoxicity is increasingly recognized to accelerate the development of neurodegenerative disorders (NDs), which pose an increasing health burden to society. Attempts are being made to develop drugs that can cross the blood–brain barrier and have minimal or no side effects. Nobiletin (NOB), a polymethoxylated flavonoid with anti-oxidative and anti-inflammatory effects, has been demonstrated to be a promising compound to treat a variety of NDs. Here, we investigated the potential role of NOB in sodium arsenate (NA)-induced deregulated miRNAs and target proteins in human neural progenitor cells (hNPCs). The proteomics and microRNA (miRNA) profiling was done for different groups, namely, unexposed control, NA-exposed, NA + NOB, and NOB groups. Following the correlation analysis between deregulated miRNAs and target proteins, RT-PCR analysis was used to validate the selected genes. The proteomic analysis showed that significantly deregulated proteins were associated with neurodegeneration pathways, response to oxidative stress, RNA processing, DNA repair, and apoptotic process following exposure to NA. The OpenArray analysis confirmed that NA exposure significantly altered miRNAs that regulate P53 signaling, Wnt signaling, cell death, and cell cycle pathways. The RT-PCR validation studies concur with proteomic data as marker genes associated with autophagy and apoptosis (HO-1, SQSTM1, LC-3, Cas3, Apaf1, HSP70, and SNCA1) were altered following NA exposure. It was observed that the treatment of NOB significantly restored the deregulated miRNAs and proteins to their basal levels. Hence, it may be considered one of its neuroprotective mechanisms. Together, the findings are promising to demonstrate the potential applicability of NOB as a neuroprotectant against chemical-induced neurotoxicity.

## 1 Introduction

Neurodegenerative disorders (NDs) represent a growing global health challenge, affecting a substantial number of individuals characterized by the progressive loss of neural cells over time. While advanced age remains the predominant risk factor for the onset of NDs, emerging research underscores the intricate interplay between an individual’s genetic predisposition and environmental toxicants. The precise etiology of these processes remains elusive, and disease-modifying therapies have yet to become standard clinical practice (Wakhloo et al., 2022). There is no definitive cure for neurodegenerative disorders. A multifaceted approach combining pharmacological and non-pharmacological treatments offer the best chance of managing symptoms and improving patients’ quality of life. Ongoing research in areas like gene therapy and stem cell transplantation holds promise for future breakthroughs in treating these debilitating conditions (Sivandzade and Cucullo, 2021). Several *in vitro* studies report that various signaling pathways are involved in differentiating stem cells into neuronal cells. One of the studies reports that Notch signaling was involved in the differentiation of neural stem cells (NSCs) derived from human umbilical cord blood-derived mesenchymal stem cells (hUCB-MSCs) (Venkatesh et al., 2017). In the current era, the use of iPSC-derived various lineages of cells are utilized to form various *in vitro* models. The formation of neural progenitor cells (NPCs) from iPSCs involved BMP and SMAD signaling pathways (Shih et al., 2023).

MicroRNAs (miRNAs) play a crucial role in neural differentiation, a key process in the development of the nervous system and in the pathogenesis of neurodegenerative disorders (Cao et al., 2016). These small, non-coding RNA molecules function as post-transcriptional regulators of gene expression. During neural differentiation, specific miRNAs are involved in the regulation of gene expression patterns that determine the fate of neural progenitor cells, guiding them toward specific neural lineages. Miao et al. reported that miR-374b promotes neural differentiation by targeting the HEs1 protein (Wu et al., 2018). The report suggests that the sets of miRNAs were identified to promote the differentiation of neural progenitors into neurons, while others might influence the development of glial cells. The change in the expression profile of a group of identified miRNAs during the differentiation of mesenchymal stem cells to neural stem cells has been reported (Venkatesh et al., 2019). Similarly, the change in the miRNA profile was also reported during iPSC neural differentiation (Kulcenty et al., 2019). According to one study, it has been found that valproate (VPA) induces the differentiation of NSCs into neurons via changes in the expression of miRNAs like miR-29a-5p, miR-674-5p, miR-155-5p, miR-652-3p, and miR-210-3p (He et al., 2018).

Natural plant products have long been known to have therapeutic benefits; conventional healthcare systems have long used phytochemicals to treat a wide range of diseases. Ongoing research sheds light on the emerging role of these bioactive compounds, isolated from natural plant products, in both standalone and adjunctive treatments for a myriad of neurological conditions. Moreover, the synergistic effects observed when combined with other therapeutic techniques underscore the versatility and potential of plant-derived compounds (Silva et al., 2022).

Nobiletin, a naturally occurring flavonoid also known as 3′,4′,5,6,7,8-hexamethoxyflavone, is a bioactive polymethoxylated flavone present in the peels of citrus fruits (Saini et al., 2022). Polymethoxylated flavones are highly permeable across BBB, attributed to their high bioavailability and increased lipophilic nature (Ullah et al., 2020). Previous studies showed that Nobiletin is emerging as a medicinal candidate in the treatment of neurodegenerative disorders. The multifaceted mechanisms through which nobiletin exerts its neuroprotective effects range from anti-inflammatory activities to modulation of neural pathways (Yasuda et al., 2014). It has already been reported that nobiletin protects neurons against amyloid-induced cognitive impairment and improves hippocampal neurogenesis in mice with memory impairment (Ghasemi-Tarie et al., 2022; Xiong et al., 2023). A recent finding showed that nobiletin confers against amyloid toxicity in primary cultures of neurons (Hung et al., 2023). Nobiletin displays a wide range of beneficial effects against the features of NDs. However, further studies are needed to determine its primary molecular targets using proteomic-based approaches (Zhang et al., 2013; Braidy et al., 2017).

Techniques rooted in array-based biomic approaches, including proteomics and transcriptomics (encompassing both mRNA and microRNA profiling), offer advanced methods for meticulously analyzing the biological interactions influenced by pharmaceutical agents, chemicals, and environmental stressors. Utilizing these biomic methodologies facilitates the identification of pivotal cellular entities, specifically proteins and regulatory non-coding miRNAs, that dictate cellular physiological processes (Liu et al., 2019; Khan et al., 2022). Our previous finding reports that sodium arsenate (NA)-induced cellular damage and stress responses were rescued by nobiletin in neural progenitor cells (NPCs) derived from human-induced pluripotent stem cells (hiPSCs) (Jahan et al., 2022). In light of the previous findings, we sought to obtain a deeper understanding of the mechanism involved with the help of proteomics and miRNA profiling. In the present study, we explored the association between NA-induced deregulation in miRNAs and target proteins in human neural progenitor cells (hNPCs) and the potential applicability of NOB to rescue/restore these changes.

## 2 Results

### 2.1 Culture and characterization of hiPSC-derived neural progenitor cells

**FIGURE 1:**
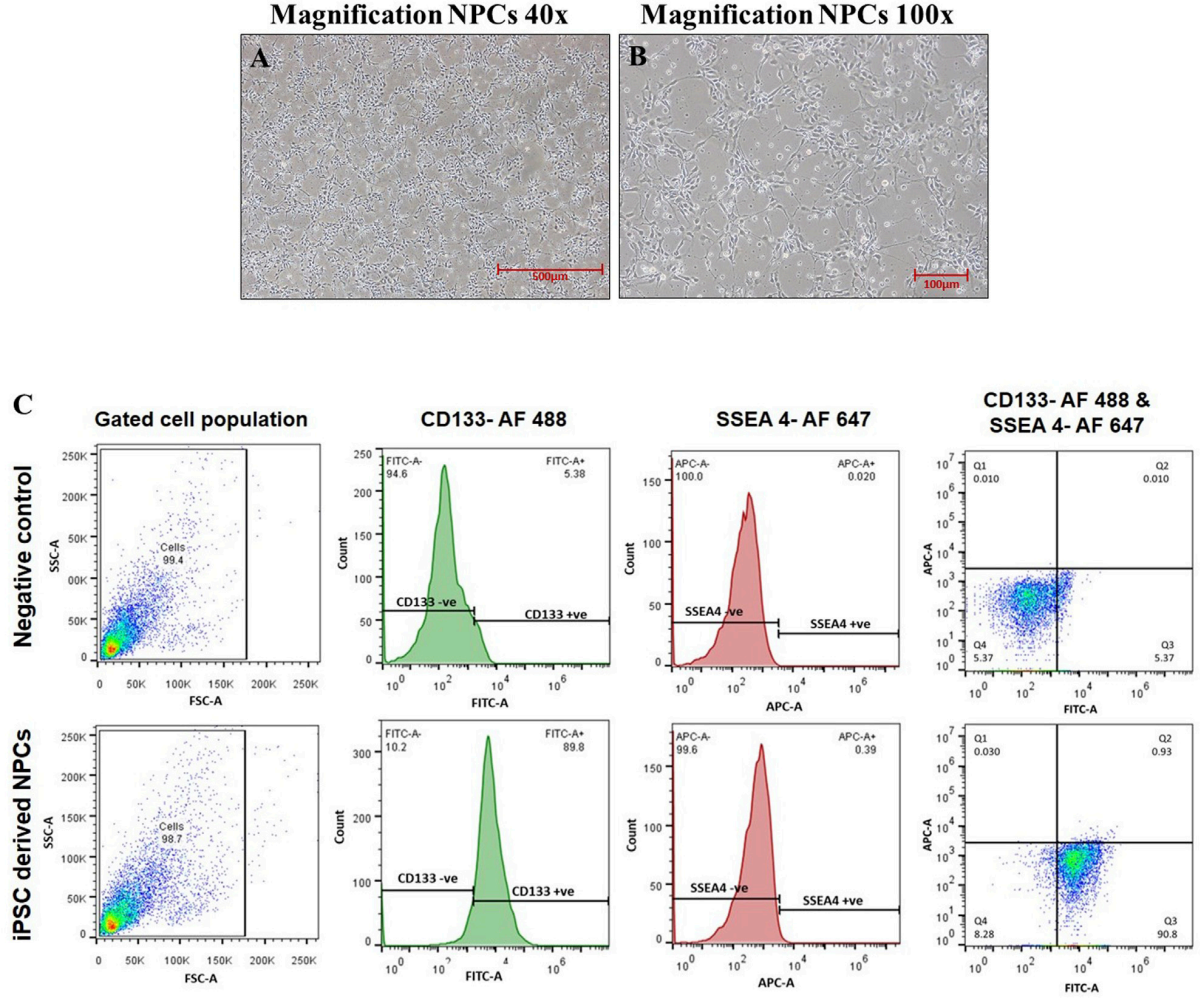
**(A–C)** Phase-contrast images of neural progenitor cells were taken at a magnification of **(A)** ×40 and **(B)** ×100 captured using a Nikon Eclipse Ti-S inverted microscope. **(C)** Characterization of hiPSC-derived NPCs via flow cytometry.

Human iPSC-derived neural progenitor cells were cultured and maintained in an NPC medium (DMEM/F12, N2, B27, and human 20 ng/mL bFGF) in a controlled environment with 5% CO_2_ at 37°C within a humidified incubator. The generation of these NPCs from hiPSCs involves various specific stages. The midline stages during the conversion of hiPSCs into NPCs were well characterized in our previous publication, and the complete procedure of NPC formation was adopted from our previously established protocol (Rajpurohit et al., 2020; Negi et al., 2023). Furthermore, hiPSC-derived NPCs were characterized for the positive expression of its specific surface marker, CD133. The flow cytometry analysis confirms that more than 89% of pure culture was CD133-positive NPCs (Figure 1). NPCs were cultured and exposed to NA 50 μM, NOB 50 μM, and co-exposure to NA 50 μM + NOB 50 μM for 48 h in accordance with our previous research for further experiments (Jahan et al., 2022).

### 2.2 Label-free quantitative proteomics

**FIGURE 2:**
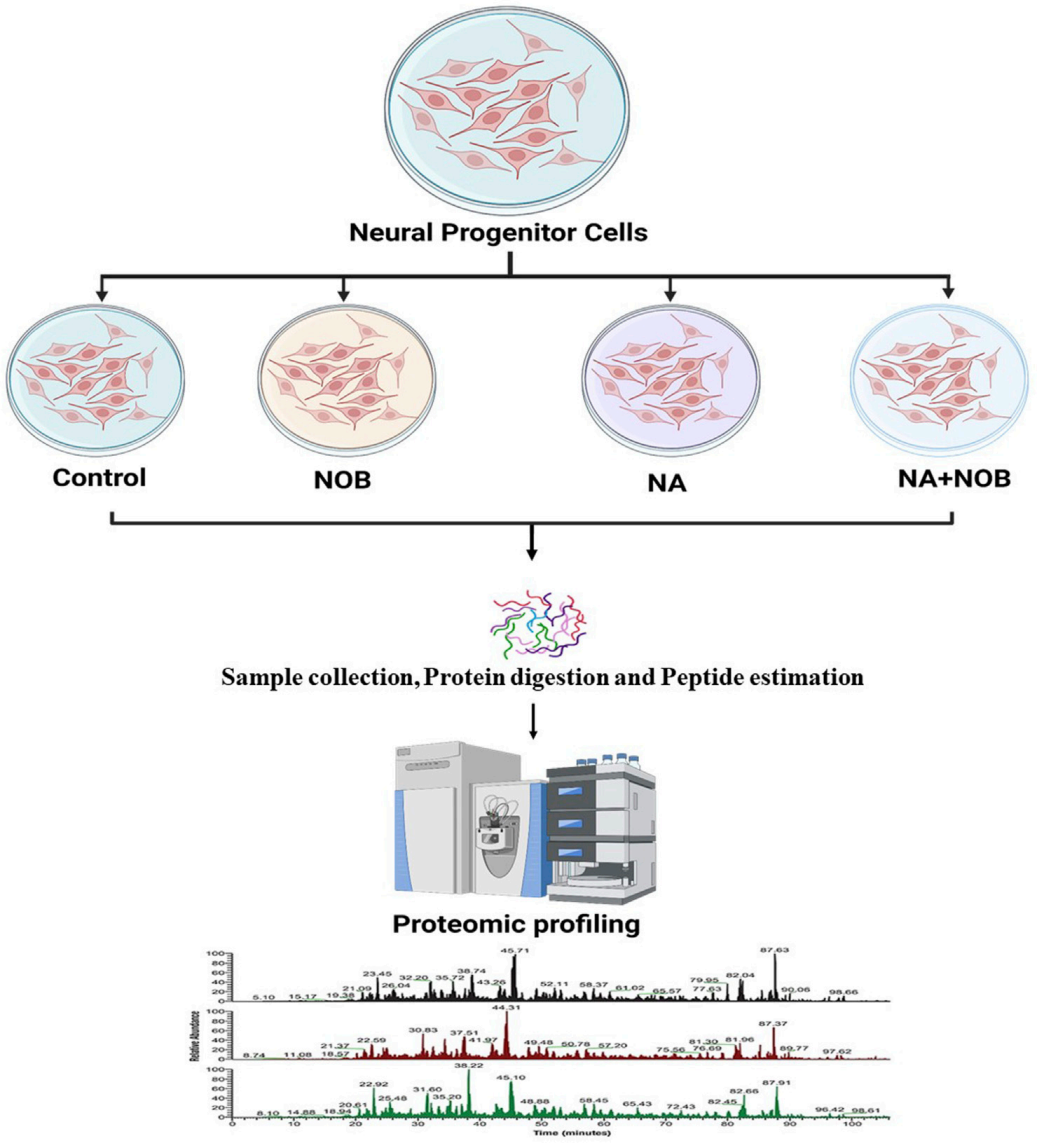
Schematic diagram of a proteomic experimental condition. Samples derived from all the four groups were digested with Trypsin/Lys-C mix of mass spec-grade for label-free quantification. An equal amount of sample peptides was subjected to nLC-MS/MS.

NPCs were exposed to previously identified biologically safe concentrations of NA (50 µM), NOB (50 µM), and co-exposure to NA + NOB (50 µM) for a period of up to 48 h followed by label-free short-gun proteomics under a high-resolution mass spectrometry (HRMS) experimental condition, as shown in Figure 2. A total of 2,234 proteins with at least two unique peptides with a 1% false discovery rate were found after the combined analysis of all four groups (control, NA exposed, NA + NOB exposed, and only NOB exposed). A total of 748 proteins (72 upregulated and 676 downregulated) were deregulated more than five-fold after exposure to NA. A list of all 748 differentially regulated proteins is provided in Supplementary Table S1. No significant protein change was found in the NOB group with respect to the control group. NA exposure induces a significant alteration (fold-change ranges from log_2_ −6.64 to log_2_ +5.99) in the proteins associated with mitochondrial bioenergetics, oxidative stress, autophagy, ubiquitin proteasome, protein synthesis, programmed cell death, etc.

NA exposure predominantly altered proteins that were related to mitochondria (MRPL4, DHODH, GATD3B, RHOT2, PRDX3 NDUFS1, etc.), causing alteration in energy production, but the co-exposure to NOB and NA restored its expression near to the control level. Similarly, the ubiquitin–proteasome system proteins (UBLCP1, USP14, UHRF1, UBL7, and RBBP6) were also deregulated. The proteins involved in the protein synthesis machinery (RPS15, MRPL45, RPL23, RPLP2, RPS28, RPLP1, RPL12, RPL17, RPS21 RPL30, RPF2, MRPL40, and MRPL28) were also deregulated after NA exposure. Apart from that, we also found that NA altered the ribosome biogenesis. Along with these key identified groups of proteins, NA exposure also induced significant alterations (fold-change from log_2_ −6.64 to log_2_ +5.99) in the expression of various other proteins. The co-exposure to NOB and NA results in the decreased magnitude of the alteration of proteins with respect to NA, which showed a protective response (Supplementary Table S2).

### 2.3 Differentially expressed proteins between NA vs. control groups

#### 2.3.1 Database for Annotation, Visualization, and Integrated Discovery analysis

**FIGURE 3:**
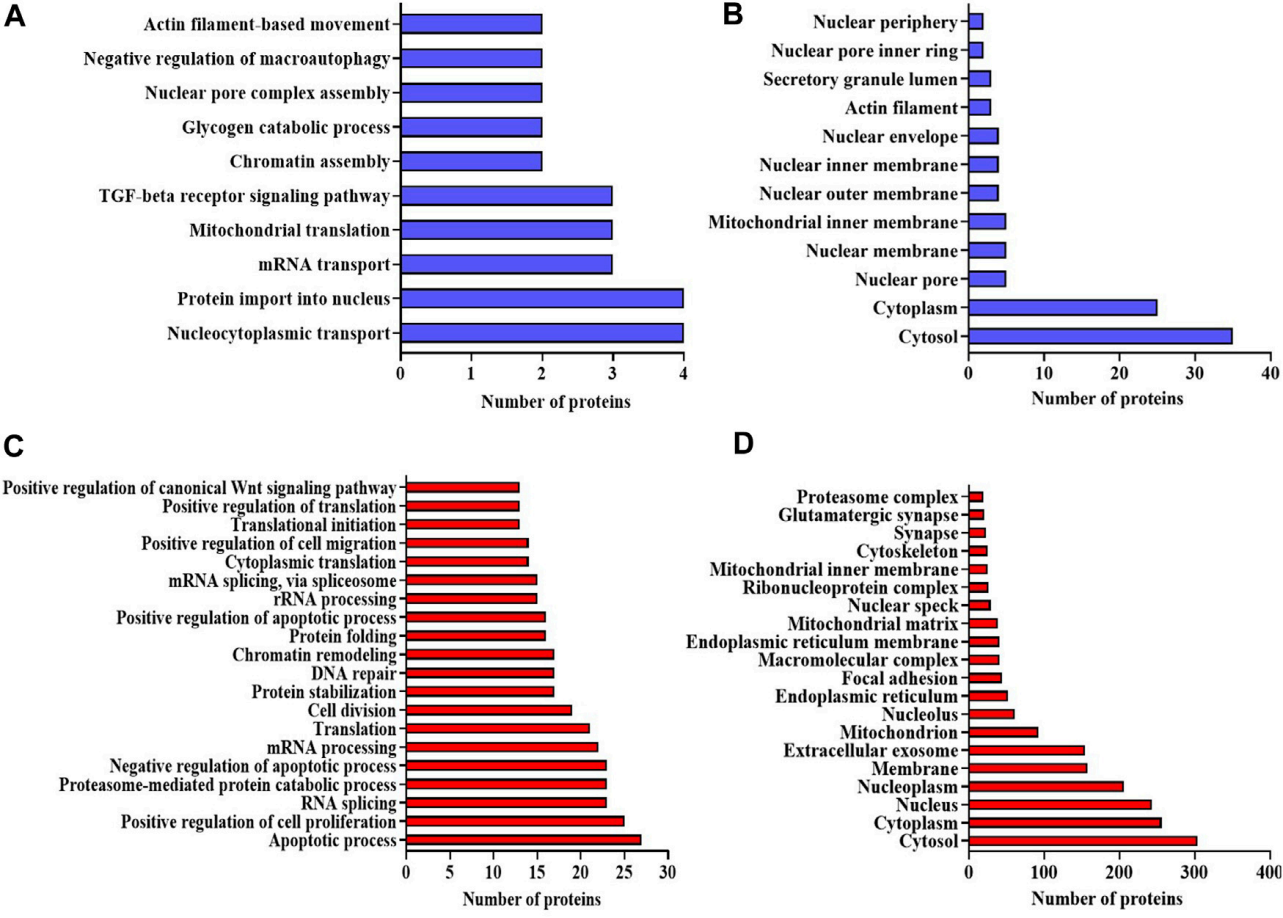
**(A–D)** Bioinformatic analysis of the DEPs in NA-exposed NPCs. GO enrichment analysis of upregulated proteins in the category of **(A)** biological process, and **(B)** cellular component. GO enrichment analysis of downregulated proteins in the category of **(C)** biological process and **(D)** cellular component. GO terms with p-value < 0.05 are considered significantly enriched analyzed using the DAVID online tool.

**FIGURE 4:**
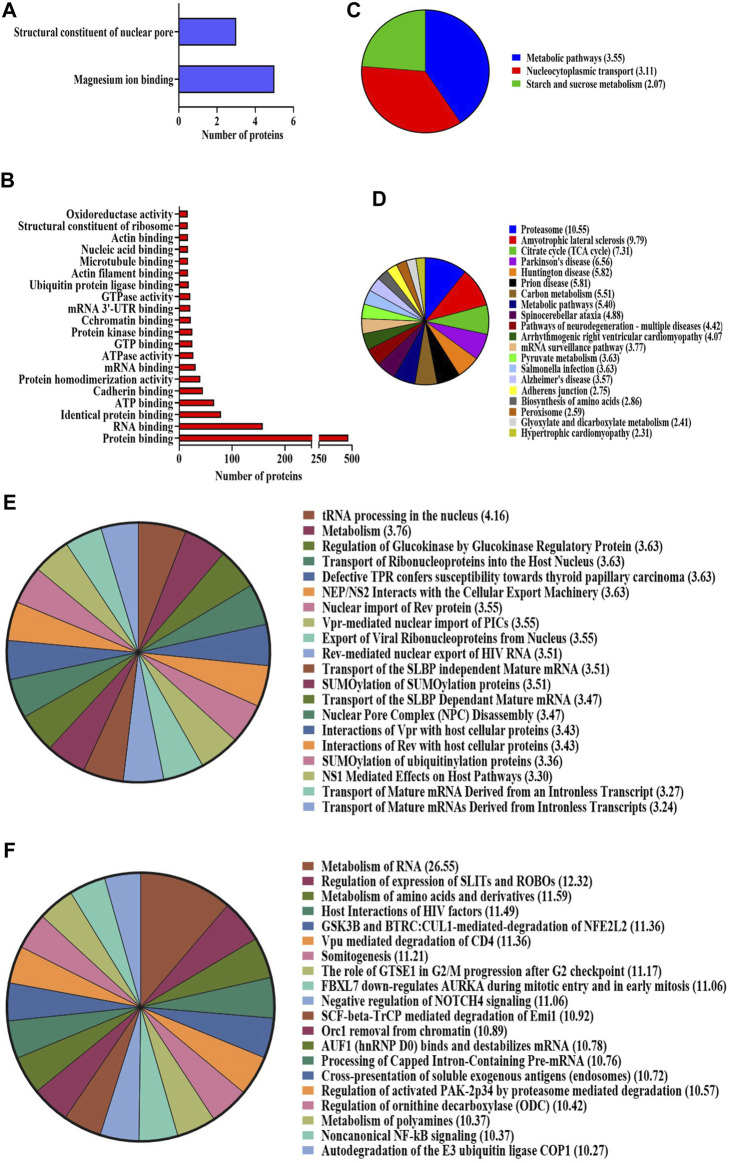
**(A–D)** GO enrichment analysis using the DAVID online tool. Molecular function of the **(A)** upregulated proteins and **(B)** downregulated proteins. KEGG pathway enrichment analysis for both **(C)** upregulated and **(D)** downregulated proteins. Pathways with a p-value < 0.05 are considered statistically significantly enriched. **(E,F)** Reactome enrichment analysis. Top 20 terms are shown in the pie chart. **(E)** Upregulated proteins and **(F)** downregulated proteins. Reactome terms with a p-value < 0.05 are considered significantly enriched analyzed using the DAVID online tool.

The bioinformatics tool, Database for Annotation, Visualization, and Integrated Discovery (DAVID), was employed to analyze proteomic data, yielding functional annotations and enriched Gene Ontology (GO) biological terms for the NA-exposed group compared to the control group. According to DAVID analysis, the biological processes (BPs) associated with differentially expressed proteins (DEPs) (upregulation and downregulation) in the NA/CON group were mainly involved in the negative regulation of neuron differentiation, apoptotic process, DNA repair, cell division, nucleocytoplasmic and mRNA transport, protein imports into the nucleus, chromatin and nuclear pore complex assembly processes, etc. Along with these biological processes, proteins were involved in various signaling pathways like TGF-beta receptor pathways and Wnt signaling pathways (Figures 3A, C). Among the DEPs, 16 proteins were related to the positive regulation of apoptosis, namely, ITGB1, MTCH1, PRKDC, HMGB1, CCAR1, SOD1, DNM2, ERCC3, C1QBP, CASP3, ACIN1, SAP18, CTNNB1, ITGA6, RNPS1, and QRICH1, 7 DEPs were related to the cellular response to oxidative stress (PRDX3, STAU1, GSR, PYCR1, ATP2A2, SOD2, and DHRS2), and 4 DEPs were involved in cell aging (NPM1, PDCD4, MIF, and SOD1). In the analysis of cellular components (CCs), DEPs exhibited a predominant localization in the cytosol, nucleoplasm, extracellular exosomes, nuclear inner and outer membranes, and the mitochondrial matrix (Figures 3B, D). Importantly, a significant proportion of these DEPs were localized to synapses. Among them, 20 DEPs were identified as glutaminergic synaptic proteins, while 23 DEPs were categorized as proteins associated with other synaptic functions. Furthermore, 17 proteins localized to the postsynaptic density exhibited downregulation. The identified molecular functions (MFs) affected by DEPs were GTPase activity, ATPase activity, and protein kinase activities (Figures 4A, B). The detailed list of all significantly identified BPs, CCs, and MFs is provided in Supplementary Tables S3, S4, and S5, respectively. In a comprehensive analysis of DEPs, we examined their enrichment in pivotal GO pathways. Notably, the KEGG pathway enrichment analysis showed that DEPs were enriched in metabolic processes, nucleocytoplasmic transport mechanisms, and several neurodegenerative disorders, including amyotrophic lateral sclerosis (ALS), Parkinson’s disease, and Huntington’s disease, as well as the broader category of multiple neurodegenerative disease pathways (Figures 4C, D). Furthermore, a Reactome pathway analysis unveiled a myriad of vital biological functions of the proteins. These encompass RNA and amino acid metabolism, negative regulation of NOTCH4 signaling, processing of capped intron-containing pre-mRNA, non-canonical NF-kB signaling, nuclear tRNA processing, glucokinase regulation, mature RNA transport, and SUMOylation of ubiquitination proteins (Figures 4E, F). A comprehensive, detailed list of all identified KEGG pathways and Reactome pathways with a *p*-value of less than 0.05 for DEPs from NA-exposed NPCs is shown in Supplementary Tables S6, S7, respectively. These data fortified that a wide range of pathways has been affected by the exposure of NPCs to NA.

#### 2.3.2 Metascape and STRING analysis

**FIGURE 5:**
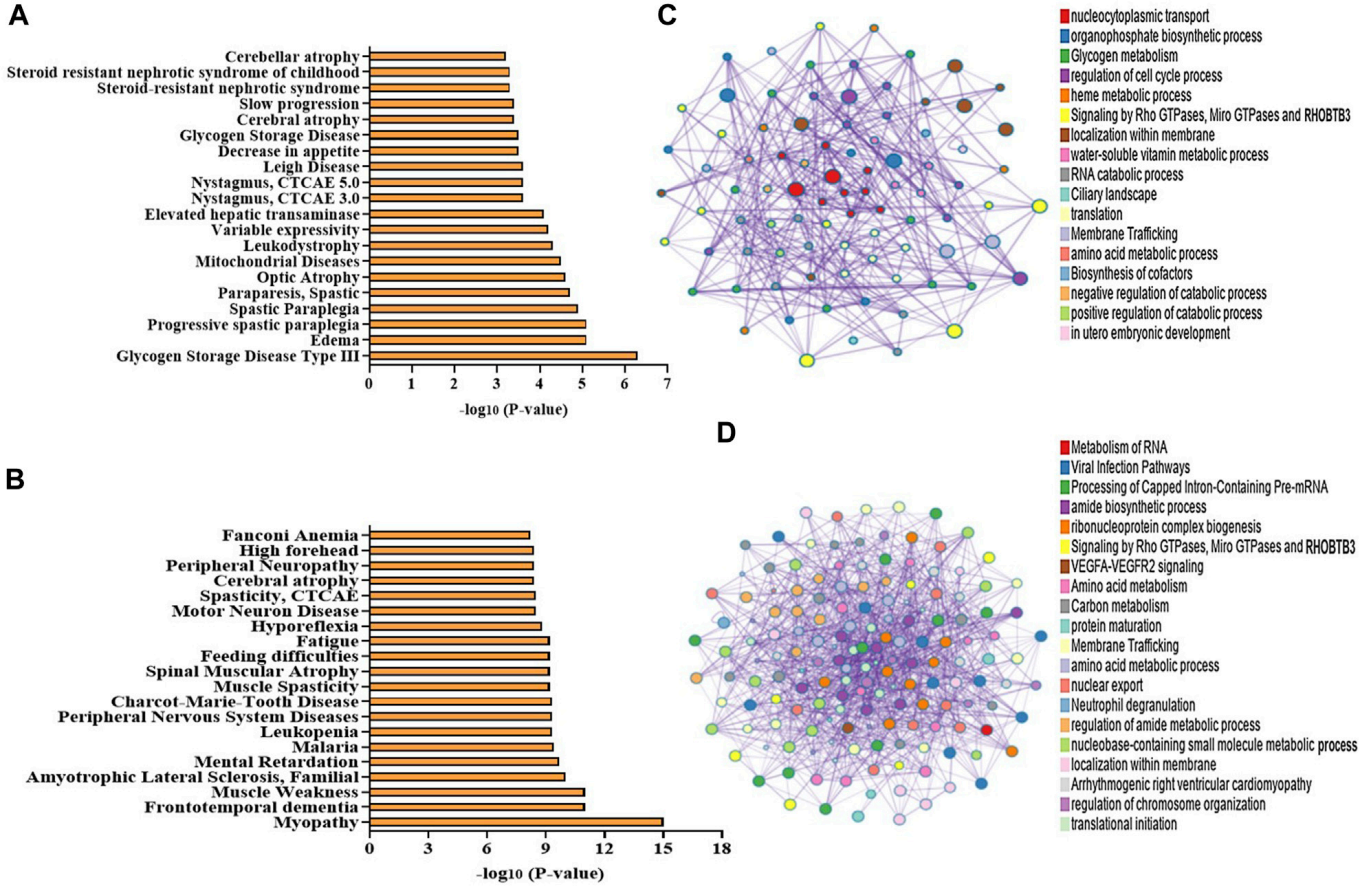
**(A–D)** Bioinformatic analysis of the DEPs in NA vs. control NPCs. Disease associations produced from the enrichment of **(A)** upregulated proteins and **(B)** downregulated proteins using the Metascape web-based portal (top 20 terms are included in the graph). **(C,D)** Network of enriched terms of the upregulated proteins and downregulated proteins obtained using a Metascape web-based portal. Colored by cluster ID, where each node represents an enriched term and is colored by its cluster. A subset of enriched terms has been selected and rendered as a network plot, where terms with a similarity > 0.3 are connected by edges.

Utilizing the protein–protein interaction (PPI) analysis tool, we investigated the interaction networks of DEPs, incorporated as a component of the PPI analysis executed via the Metascape and STRING algorithms. The outputs from Metascape result in the association of DEPs with various diseases like cerebellar atrophy, mitochondrial disease, motor neuron diseases, and spinal muscular dystrophy (Figures 5A, B). Furthermore, Molecular Complex Detection (MCODE) is a computational method used to identify and characterize protein complexes in biological systems. We found various MCODE clusters in DEPs (Figures 5C, D).

**FIGURE 6:**
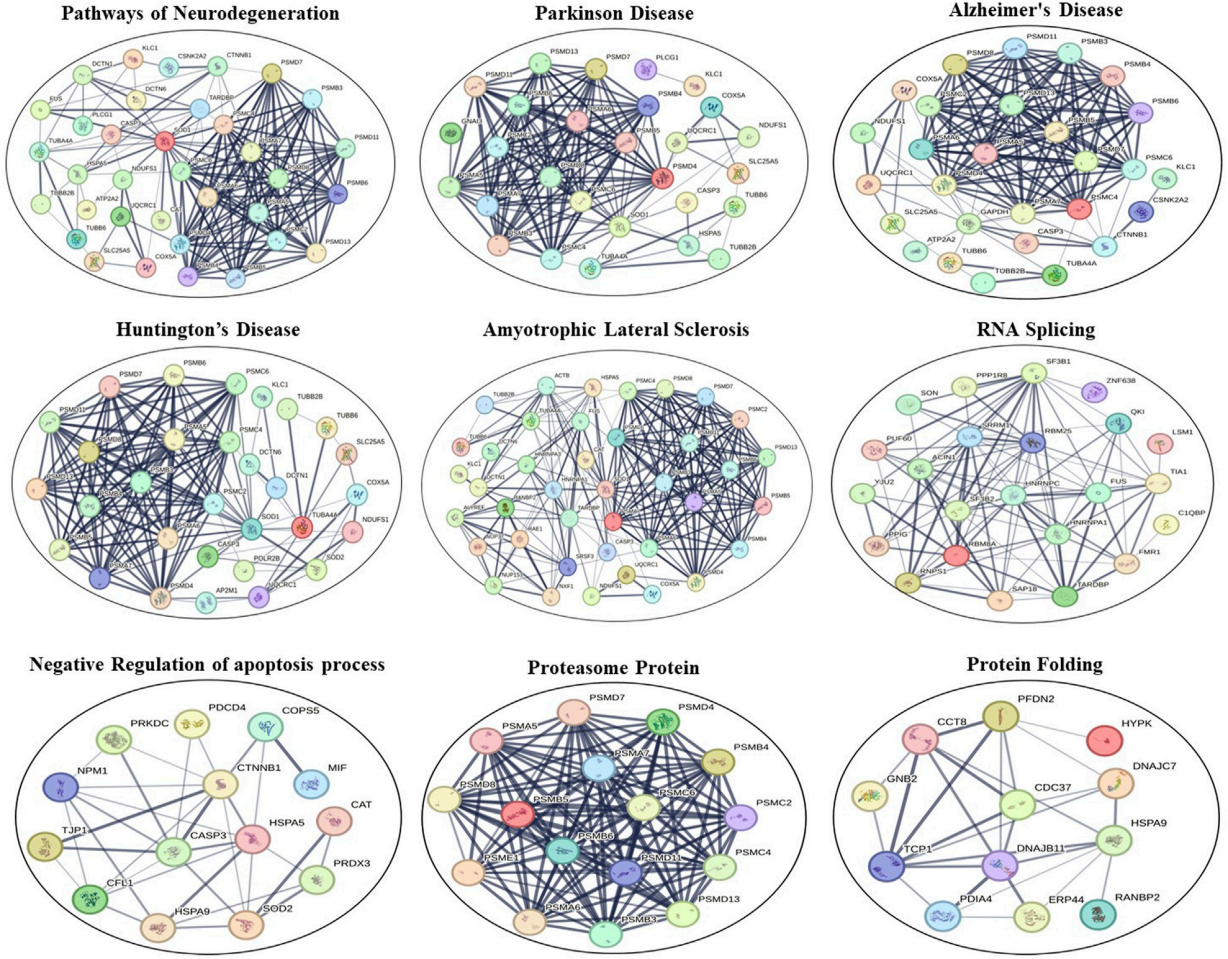
STRING interaction networks of DEPs using the online STRING database (version 12.0). Lines between proteins show the confidence of an interaction with thicker lines representing stronger interactions. Disconnected nodes in the network are removed.

STRING software underscored the potential pivotal roles of these deregulated proteins in several pathways and processes. Specifically, these proteins appeared to be integral to the pathways of neurodegeneration—encompassing multiple diseases, the citrate cycle (also known as the TCA cycle), mitochondrial calcium ion regulatory mechanisms, NADP metabolic processes, glycolysis/gluconeogenesis pathways, the organization of the inner mitochondrial membrane, responses to oxidative stress, cellular oxidant detoxification procedures, the mRNA surveillance pathway, proteasomal protein functionalities, synapse assembly processes, general metabolic pathways, nucleocytoplasmic transport mechanisms, and ALS. These findings are shown in Figure 6.

### 2.4 Differentially expressed proteins between NA/CON and NA + NOB/NA

We also compared protein expression profiles between two groups: NA/CON and NA + NOB/NA. This analysis specifically targeted the identification and categorization of DEPs inherent to various classes of proteins. Comparative analysis resulted in a significant alteration in the protein deregulation pattern following the exposure of NPCs to NA. Intriguingly, our results suggested that NOB treatment serves to modulate this deregulation, driving the protein expression dynamics similar to that observed in a normal physiological state, as shown in Supplementary Table S8. This shift in protein expression, in light of NOB treatment, provides compelling evidence of its potential therapeutic efficacy in rectifying NA-induced protein deregulation in NPCs.

### 2.5 Protective effects of NOB by the reversal of protein levels between the NA and NA + NOB groups

**FIGURE 7:**
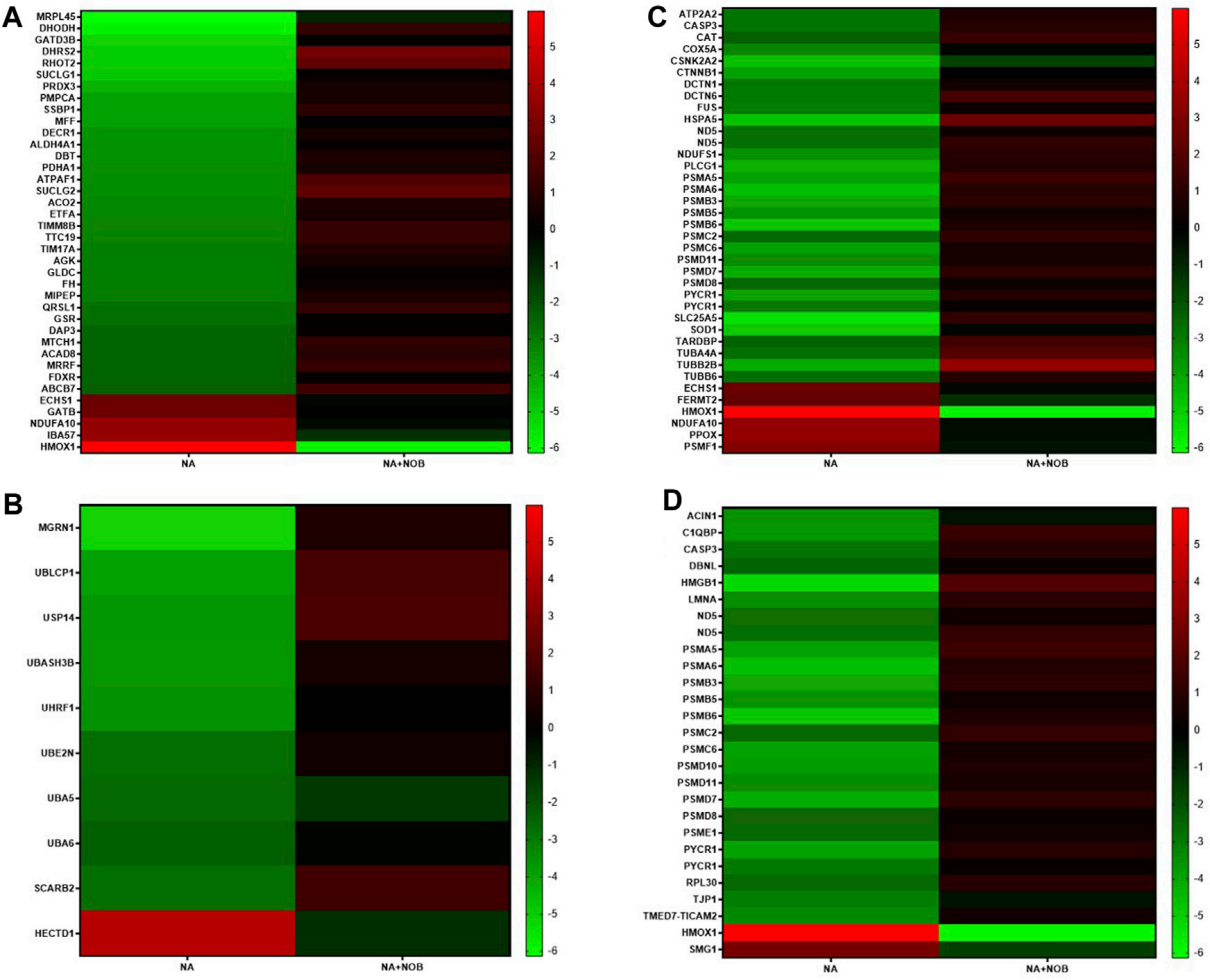
**(A–D)** Heatmap showing associated KEGG pathways (p < 0.05) of identified proteins in NA-exposed NPCs and their reverse effect by the NOB exposure. These proteins belong to **(A)** mitochondrial and oxidative stress groups, **(B)** ubiquitin–proteasome system, autophagy, chaperons, **(C)** neurodegeneration group, and **(D)** programmed cell death pathways.

In our comprehensive proteomic analysis, we investigated the proteins whose expression was altered due to NA exposure, particularly focusing on those essential for maintaining cellular homeostasis, and also assessed the potential role of NOB in protecting against the deleterious effects induced by NA. Our investigations identified a total of 113 proteins whose expression patterns were notably reversed between the NA and NA + NOB groups. Amongst them, 14 proteins exhibited an upregulated trend, while 99 proteins were markedly downregulated. Intriguingly, the presence of NOB led to a reversal in these expression trajectories. Notably, these proteins played pivotal roles in pathways such as mitochondrial and oxidative stress response, neurodegeneration, ubiquitin–proteasome system, chaperone-mediated protein folding, and programmed cell death pathways. The spatial distribution analysis further revealed that these proteins were ubiquitously localized across various cellular compartments, highlighting their widespread impact. The reversal of expression of these proteins results in the functioning of normal cell physiology, ultimately showcasing the protective effect of NOB (Figure 7).

### 2.6 Expression profiling of miRNAs and their functional enrichment

**FIGURE 8:**
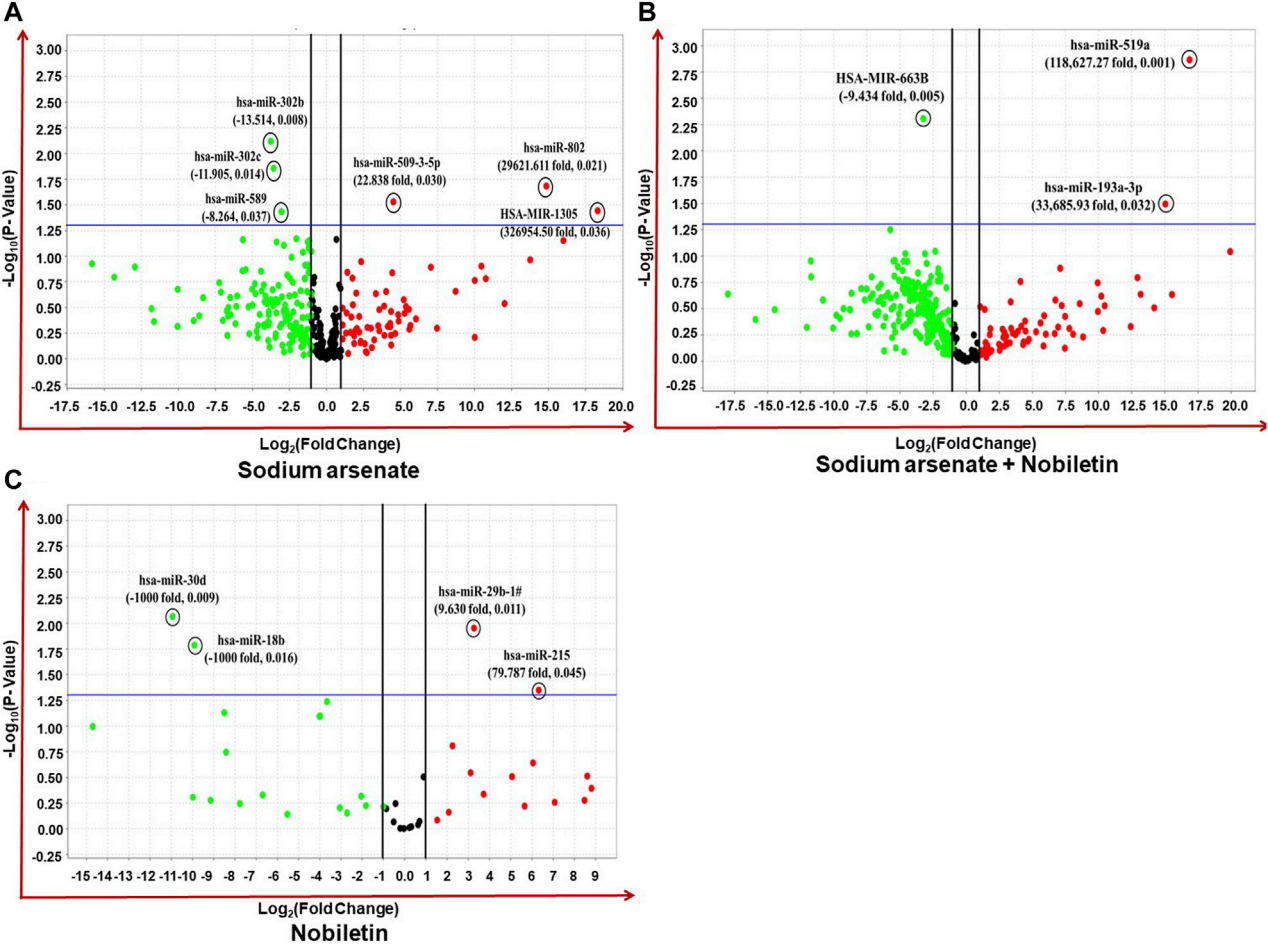
**(A–C)** Expression profiling of miRNAs by real-time PCR OpenArray in human iPSC-derived NPCs. **(A)** Alteration of miRNAs in the NA-exposed group, **(B)** alteration of miRNAs in the NA + NOB group, and **(C)** alteration of miRNAs in the NOB group.

The high-throughput OpenArray-based miRNA profiling revealed the NA-mediated significant dysregulation of miRNA. A volcano plot is drawn using log_2_ values of the fold difference, and the *p*-value shows the effect of NA exposure on NPC miRNAs (Figure 8).

**FIGURE 9:**
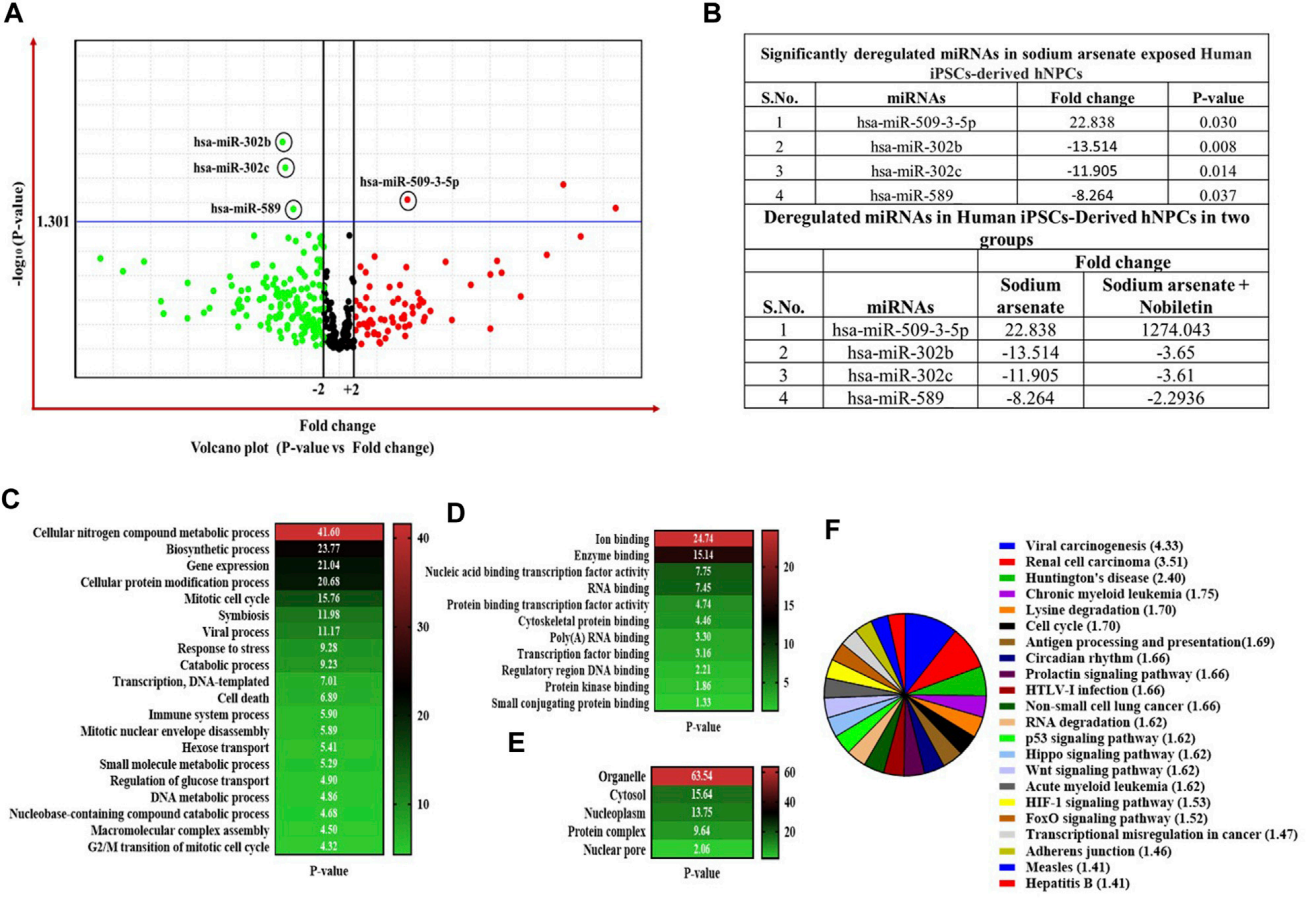
**(A–F)** Expression profiling of miRNAs by real-time PCR-based OpenArray. **(A)** Volcano plot of miRNA expression. The volcano plot is plotted between *p*-value and fold-change. The single horizontal line in the volcano plot represents the *p*-value of the *t*-test, a threshold set as 0.05, while the additional two vertical lines represent the cut-off boundary (± two-fold) for downregulated and upregulated miRNA expression. Above the vertical line, all the green dots indicate the significantly downregulated miRNAs, and all the red dots indicate the significantly upregulated miRNAs. **(B)** List of significantly deregulated miRNAs. Expression of miRNAs is represented as relative quantification. All the experiments were performed in three biological replicates. **(C,D)** Bioinformatic analysis of the deregulated miRNAs in NA-exposed NPCs. GO enrichment analysis of miRNAs is shown in the category of **(C)** biological process, **(D)** molecular function, and **(E)** cellular component using a DIANA-miRPath v3.0 online web portal. Top 20 terms are shown in the GO graph. GO terms with a *p*-value less than 0.05 are considered significantly enriched. **(F)** *In silico* KEGG pathway enrichment of significantly deregulated miRNAs. The DIANA-miRPath v3.0 online web portal was used. KEGG pathways and terms with a *p*-value less than 0.05 are considered significantly enriched.

Four miRNAs (miR-509-3-5p, miR-302b, miR-302c, and miR-589) were found to be dysregulated after NA exposure. The observed deregulation in the expression of miR-509-3-5p, miR-302b, miR-302c, and miR-589 in the NA + NOB-exposed group was compensated by NOB (Figures 9A,B). The *in silico* approach was employed to assess the GO enrichment of miRNAs that displayed significant deregulation upon exposure to NA in NPCs. This GO enrichment analysis discerned the involvement of these differentially expressed miRNAs across three primary domains: biological processes, molecular functions, and cellular components. Utilizing the DIANA-miRPath v3.0 online portal, we identified miRNAs associated with a range of biological processes, including but not limited to the biosynthetic process, gene expression, cellular protein modification, stress response, cell death, DNA metabolism, cytokine-mediated signaling, nucleocytoplasmic transport, and unfolded protein response activation (Figure 9C). In the domain of molecular function, these miRNAs were linked to enzyme binding, RNA binding, transcription factor binding, protein kinase binding, and cytoskeletal protein binding. From a cellular component perspective, their association spanned organelles, cytosol, nucleoplasm, and protein complexes, as shown in Figures 9D,E. The KEGG pathway analysis of significantly deregulated miRNAs showed the involvement of different numbers of miRNAs in various biological pathways, including FOXO signaling, Huntington’s disease, HIF-1 signaling pathway, P53 signaling, adherens junction, Wnt signaling pathway, Hippo signaling, cell cycle, RNA degradation, p53 signaling pathway, RNA degradation, stress-activated MAPK cascade, and cell death (Figure 9F).

### 2.7 Real-time PCR analysis

**FIGURE 10:**
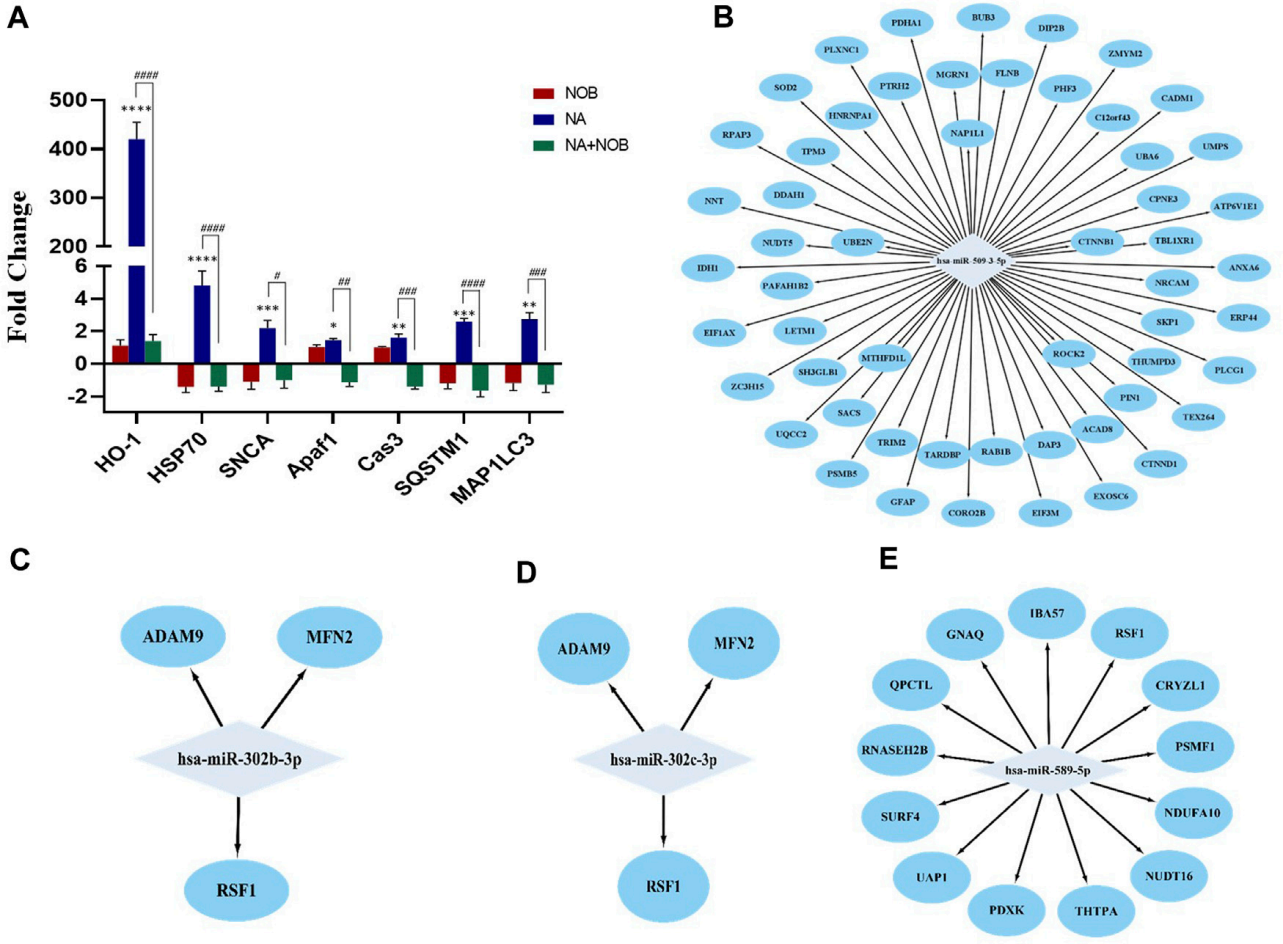
**(A–E) (A)** Transcriptional changes in the expression level of various marker genes. Bar graphs show the values for different mRNA expression levels in fold-change. Beta-Actin was used as the endogenous control to normalize the data. Data are presented as the ± SEM of three separate experiments performed in biological replicates (n = 3). The changes in the expression pattern are statistically significant as indicated by *p < 0.05, **p < 0.01, ***p < 0.001, and ****p < 0.0001 vs. control NPCs and # < 0.05, ## < 0.01, ### < 0.001, and #### < 0.0001 compared with NA. **(B–E)** MicroRNA–protein regulatory modules showing the co-relation between miRNAs and proteins. **(B)** MicroRNA–protein regulatory modules showing upregulated miRNAs and downregulated proteins. **(C,D, and E)** MicroRNA–protein regulatory modules showing downregulated miRNAs and upregulated proteins.

The qPCR analysis was performed in genes related to oxidative stress autophagy and apoptosis. The relative expression of heme oxygenase 1 (HO-1) was found to be highly upregulated (400-fold). In addition, other genes like *HSP70*, *MAP1LC3*, *SNCA*, *APAF1*, *CASPASE-3*, and *SQSTM* were also upregulated after NA exposure. The co-exposure to NOB and NA results in the downregulation of these genes compared to the NA-exposed group. The expression of these genes was found to be statically nonsignificant in the NOB-exposed group compared to the control group (Figure 10A).

### 2.8 Correlation among differentially expressed proteins and miRNAs

The correlation between miRNAs and proteins affected by NA exposure in NPCs was investigated. The potential targets of specific miRNAs were pinpointed using the TargetScan database. The detailed examination of individual miRNAs and associated proteins revealed that 13 proteins possess binding sites specific to miR-589, and these proteins exhibited increased expression in protein profiling. In parallel, both miR-302b and miR-302c possess binding sites for three proteins that demonstrated elevated expression levels. Furthermore, the upregulated miR-509-3-5p is associated with over 20 target sites, all of which are observed to have downregulated expression (Figures 10B–E). These findings suggested a direct regulatory relationship between the identified miRNAs and protein expression levels.

## 3 Discussion

Various citrus fruits contain a dietary polymethoxylated flavonoid known as NOB. Recent research has demonstrated that NOB is a multipurpose pharmaceutical ingredient. Various studies cover the different aspects of the neuroprotective potential of nobiletin; however, studies interpreting high-throughput proteomics and miRNA profiling and their correlation are yet to be performed. In the present study, through proteomic and miRNA studies, we investigated changes in the expression level of the various classes of proteins and miRNAs in NPCs after exposure to NA alone and co-exposure to NA and NOB.

The proteomic profile demonstrates that the DEPs in NA/CON groups are primarily associated with mitochondrial bioenergetics, oxidative stress, autophagy, ubiquitin–proteasome, protein synthesis, and programmed cell death. Previous studies reveal that arsenic exposure causes oxidative stress, autophagy, and apoptosis (Yen et al., 2011; Gupta et al., 2021). The expression of HO-1 was found to be very high at proteomic and mRNA levels after the exposure of NPCs to NA. The preceding research showed that exposure of HK-2 cells to arsenic caused dose- and time-dependent increases in HO-1 expression in both protein and enzymatic forms (Gong et al., 2016). Arsenic exposure has already been proven to induce the oxidative stress in neuronal cells, and the neuron increases the expression of HO-1 to resist oxidative stress-mediated cell death (Lu et al., 2014; Wu and Hsieh, 2022). The co-exposure to NOB and NA results in the reversal of HO-1 expression to the basal level. Arsenic exposure causes neurotoxic effects by stimulating the expression of caspase-3 and HO-1. It has been found that HO-1 showed a protective effect on arsenite-mediated neurotoxicity in primary cultured cortical neurons (Teng et al., 2013). Nobiletin, a notable compound, can counteract ischemic brain injuries by suppressing NF-kB expression and concurrently stimulating the Nrf2/HO-1 and Akt/CREB pathways (Nakajima and Ohizumi, 2019). Research conducted by (Amarsanaa et al. 2021) suggests that nobiletin offers partial protection against the inhibition of mitochondrial complex I by bolstering HO-1 and Nrf2 signaling mechanisms. In a parallel observation, our study on NA-exposed groups revealed an augmented expression of HO-1 and caspase-3, indicating the presence of stress and apoptosis in NA-exposed NPCs. Interestingly, there was a noticeable attenuation in the elevated levels of HO-1 and caspase-3 activity upon NOB exposure, emphasizing its potential as an anti-stress and anti-apoptotic agent. In our recent proteomic analysis, we identified an extensive array of proteins associated with mitochondrial functionality, oxidative stress, the ubiquitin–proteasome system (UPS), and endoplasmic reticulum (ER) stress, as well as processes related to translation, transcription, and nuclear transport. Numerous studies have identified mitochondrial dysfunction as a key factor associated with the onset of oxidative stress, which is a pivotal event in chemical-induced neurotoxicity and developmental neurotoxicity induced by arsenic exposure (Medda et al., 2020; Zhang et al., 2023). Notably, the exposure of NPCs to NA has exhibited marked neurotoxicity, characterized by the generation of reactive oxygen species (ROS), upregulated expression of HO-1, and the enhanced presence of antioxidant proteins, such as PDXK, GSTM3, and NDUFA10. In addition, the downregulation of mitochondrial resident proteins, such as RHOT2, NDUFS1, COX5A, PMPCA, and MFF, leads to the abnormal functioning of mitochondria causing neurotoxic effects. Under conditions of stress or pathology, cellular mechanisms orchestrate alterations in gene expression, protein folding, and global protein biosynthesis to optimize cell survival (Advani and Ivanov, 2019). Empirical evidence has demonstrated that oxidative stress induced by arsenic exposure perturbs protein translation processes and protein folding. This disturbance is mediated by the modulation of the functional activity of key translation factors and associated regulatory proteins (Thakur et al., 2021). Our findings revealed that NA exposure caused the downregulation of ribosome-associated proteins, such as RPS15, MRPL45, RPL23, SBDS, NIP7, RPLP2, EXOSC2, RPSA, RPS28, RPLP1, RPL12, RPL17, RPS2, and RPS21, which causes the inhibition of the normal protein synthesis. The co-exposure to NOB and NA results in the reversal of the deregulation of these ribosomal proteins to the basal level, showing the protective effect. Various studies also show that nobiletin provides protection against endoplasmic stress (Takano et al., 2007). Protein folding is also a major phenomenon for cell survival. (Moon and Cho 2016) reported that nobiletin elicits defensive autophagic response to concurrent endoplasmic reticulum-stress-driven apoptotic processes in SNU-16 human gastric cancer cells.

The dysfunction of E3 ubiquitin ligase has been implicated in developmental anomalies, including aberrations in left–right axis patterning and the emergence of congenital heart defects and neurodegenerative effects (Zhang et al., 2014; Upadhyay et al., 2016). In our experimental findings, NPCs exposed to NA exhibited modulated expression levels of RENF40, MGRN1, and ATG7, suggesting perturbations in the UPS. Our RT-PCR analysis further revealed an upregulation in the mRNA levels of proteins, such as HSP70, SQSTM, and MAP1LC3. Collectively, these markers serve as indicators of disrupted UPS functionality and heightened autophagic activity in NA-exposed NPCs. Conversely, exposure to NOB led to normalization in the expression of UPS and autophagy-related proteins, positioning NOB as a potential therapeutic agent against arsenic-mediated UPS impairment.

Notably, DNA/RNA-binding proteins like fused in sarcoma/translated in liposarcoma (FUS/TLS) and TAR DNA-binding protein (TDP)-43 have been pinpointed as the primary etiological factors in the familial forms of the neurodegenerative condition of ALS (Zhang et al., 2014). Our proteomic analysis revealed that FUS and TARDBP expression levels were diminished in NPCs exposed to NA. Additionally, several other proteins, including ATP2A2, CASP3, CAT, COX5A, CSNK2A2, CTNNB1, DCTN1, DCTN6, and HSPA5, exhibited increased expression levels in response to NA exposure in NPCs. Given their association with neurodegenerative pathways, these findings underscore the pronounced neurotoxicity in NPCs subjected to NA. Intriguingly, NPCs treated with NOB demonstrated a recuperative trend in the expression of these markers, suggesting that NOB holds promise as an herbal therapeutic agent for counteracting chemical-induced neurodegeneration.

Recent studies have elucidated that nobiletin, a citrus flavonoid, exerts its anticancer efficacy through the modulation of miRNA expression in various cancer types. (Bayoumi et al. 2021) demonstrated that nobiletin normalizes miRNA expression, contributing to its protective effects in skin cancer. The loss and gain function studies on miR-200 overexpression in breast cancer cells (MCF-7) treated with nobiletin have been linked to improved outcomes in these cells (Wang et al., 2021). Additionally, a synergistic effect of nobiletin, tangeretin, and hesperidin—a combination termed as citrus flavonoid purity mixture (CFPM)—has been shown to enhance lipid metabolism in HepG2 cells by suppressing miR-33 and miR-122 (Su et al., 2019). Moreover, the downregulation of miR-15-5p upon nobiletin exposure has been observed to inhibit non-small-cell lung cancer in A549 cells (Han et al., 2021). Comprehensive research indicates that nobiletin’s regulation of miRNA expression plays a pivotal role in modulating various oncogenic pathways. In the context of neurodegenerative disorders, the quantitative analysis of miRNAs in NPCs exposed to a post-neurotoxic agent (NA) revealed alterations in four specific miRNAs: hsa-miR-509-3-5p, hsa-miR-302b, hsa-miR-302, and hsa-miR-589. Prior literature, including (Singh et al. 2014), suggested that miR-302 targets the cyclin D2 gene (CCND2), leading to ethanol-induced neuronal apoptosis through non-mitochondrial pathways. Furthermore, (Yadav et al. 2011) identified an association between increased miR-302b levels and caspase-3-induced apoptosis in SH-SY5Y cells. Previous reports showed the protective effects of nobiletin via regulating the normal expression of miRNA against different cancers (Bayoumi et al., 2021; Wang et al., 2021). The quantitative analysis of miRNAs in neural NPCs exposed to NA revealed alterations in four specific miRNAs: hsa-miR-509-3-5p, hsa-miR-302b, hsa-miR-302, and hsa-miR-589. Previous literature suggests that miR-302 primarily targets the CCND2, instigating ethanol-induced neuronal apoptosis via non-mitochondrial mechanisms (Singh et al., 2014). Furthermore, the elevated levels of miR-302b have been associated with caspase-3-induced apoptosis in SH-SY5Y cells (Yadav et al., 2011). Investigations using a miR-302-deficient mouse model revealed that the absence of miR-302 disrupted processes such as cell proliferation, apoptosis, differentiation, and neural tube closure during early neurodevelopment (Parchem et al., 2015). This underlines the pivotal role of miR-302 in neuronal differentiation and embryonic viability. Our observations demonstrated that NOB exposure augmented the expression levels of miR-302b and miR-302c relative to the NA-treated group. It is noted that NOB preserves the essential expression levels of these miRNAs, consequently safeguarding NPC proliferation and viability. The targets of the altered miRNAs encompass proteins active in the FOXO signaling cascade. The FOXO transcription factor family modulates gene expression pivotal for cellular events like apoptosis, cell cycle regulation, glucose metabolism, resistance to oxidative stress, and longevity (Tia et al., 2018; Rodriguez-Colman et al., 2023). FOXOs are integral for neural stem cell (NSC) dormancy and the eradication of reactive oxygen species within neural stem cell niches (Santo and Paik, 2018). Several other molecular pathways, including the Wnt signaling pathway, pathways associated with Huntington’s disease, and the HIF-1 signaling pathway, are influenced by miRNAs disrupted in NPCs exposed to NA. Nonetheless, the combined exposure of NPCs to NA and NOB minimizes the deregulation of miRNAs linked to apoptotic and cell death pathways. This suggests that NOB might protect NPCs from NA-induced miRNA alterations that are associated with cellular apoptosis and death.

## 4 Materials and methods

### 4.1 Generation and characterization of NPCs and their propagation

Human episomal iPSC lines were procured from Thermo Fisher Scientific and cultured using the Essential 8 medium on Matrigel-coated 60-mm dishes and maintained in a controlled environment at 37°C with 5% CO_2_ for optimal growth. To derive NPCs, iPSC colonies were manually segregated and suspended in ultra-low attachment plates for forming embryoid bodies (EBs). These EBs were further cultured on Matrigel-coated dishes to generate NPCs following the previously published protocol (Rajpurohit et al., 2020). The iPSC-derived NPCs underwent flow cytometry analysis through a BD FACSCanto-II flow cytometry apparatus (Becton Dickinson, San Jose, CA, USA). During this process, the cells were incubated with specific surface markers, SSEA-4 and CD133. These markers were conjugated with Alexa Fluor 647 and 488 fluorescent dyes, respectively. Following the incubation period, the cells were washed thrice using PBS and then reconstituted in 0.5 mL PBS solution. Concurrently, a comparative group of cells, unexposed to the antibody and maintained under similar conditions, was utilized as a control group. Standardized acquisition protocols were followed. The cell populations were initially categorized based on their forward and side scatter (FSC vs. SSC) characteristics. For each sample, 10,000 events were consistently recorded at a medium flow rate. A subsequent analysis of the cell population percentages was conducted using FACSDiva software and FlowJo V10 (USA). Our study comprised four groups: unexposed control, NPCs exposed to 50 µM NA, NPCs exposed to 50 µM NOB, and a co-exposure to NA and NOB at 50 µM each for 48 h based on prior experiments (Jahan et al., 2022).

### 4.2 Proteomic studies by LC-MS/MS

The label-free quantification methodology was executed using advanced quantitative proteomic techniques, employing a state-of-the-art high-resolution mass spectrometer in conjunction with a nanoflow reversed-phase chromatography system (nano-LC).

#### 4.2.1 Sample preparation

The whole-cell protein was extracted from all four experimental groups utilizing a lysis buffer comprising 100 mM Tris-HCL, 0.15 M NaCl, 1 mM EDTA, 1% NP-40, 0.15% sodium deoxycholate, 1 mM PMSF, a protease inhibitor cocktail at 10 mL/mL, and 0.5 mM DTT. To eliminate detergents, we employed Pierce detergent removal spin columns (Catalog# 8776). The Pierce BCA protein assay kit (Catalog# 23227) was used for protein quantification. An in-solution digestion method for LC-MS/MS was adopted, paralleling the methodology delineated in a previously published protocol (Srivastava et al., 2020). The proteolytic enzyme, Trypsin/Lys-C, was introduced and incubated overnight at 37°C to ensure comprehensive protein digestion. Subsequent tryptic peptides were processed through Pierce C18 columns, following the guidelines provided by the manufacturer. Following column elution, the samples underwent lyophilization using a vacuum evaporator. These lyophilized samples were reconstituted in 0.1% formic acid solution.

#### 4.2.2 Generation of the spectral data by data-dependent acquisition

The raw spectral data were collected using the EASY-nLC 1200 system (Thermo Fisher Scientific, Waltham, MA) connected in tandem with a Q Exactive mass spectrometer, equipped with a nano-electrospray ion source controlled using Xcalibur software. A dual-column chromatographic setup was employed. Peptide separation was achieved using a C18 reversed-phase column with a 180-min gradient at 0.3 μL/min. The gradient initiated at 10% B and underwent a linear increase to 35% B over 130 min and 50% B over 40 min, followed by a 10-min increase to 95% B. The electrospray ionization voltage was set at 2.3 kV, and the heated capillary temperature for mass spectrometry was maintained at 300°C. The Orbitrap mass analyzer was used for data-dependent MS/MS acquisition (DDA), applying a dynamic exclusion duration of 60 m. During MS1, a resolution of 70,000 was used with an automatic gain control set at 3 × 10^6^ and a maximum injection time of 100 m. For MS2, settings included a resolution of 17,500, an automatic gain control target of 1 × 10^5^, and a maximum injection time of 60 m. Each group of samples was run in two biological replicates with their technical triplicate.

#### 4.2.3 Label-free quantitative proteomic analysis

The acquired spectral data were analyzed using the Proteome Discoverer 2.4 software (Thermo Fisher Scientific, Waltham, MA) and matched against the UniProt *Homo sapiens* database (SwissProt TaxID 9606), with mass tolerances defined at 10 ppm. Variable modifications encompassed oxidation (M) and protein N-terminal acetylation, while carbamidomethyl (C) was designated as the fixed modification. A 1% false discovery rate was established for peptide discernment. Quantitative analysis was based on relative abundance expressed as log_2_ values with significance *p*-value ≤0.05.

### 4.3 Transcriptional analysis

#### 4.3.1 Expression profiling of miRNAs

In order to profile miRNA expression patterns, all four experimental NPCs underwent total RNA extraction via the mirVana miRNA extraction kit (Catalog # AM1561, Thermo Fisher Scientific). Profiling of these miRNAs was executed through specialized TaqMan human miRNA panel OpenArray plates designed specifically for the 754-target miRNA assay (Cat. # 4470187 Thermo Fisher Scientific). The reverse transcription was facilitated by the TaqMan miRNA reverse transcription kit using the Megaplex Primer Pools (PN 4444750), which employed stem-loop reverse transcription primers for the aforementioned 754 miRNAs. Subsequently, a pre-amplification stage of the synthesized cDNA was initiated using the TaqMan PreAmp Master Mix in conjunction with an OpenArray primer pool (Catalog # 4444748). The pre-amplified products were then subjected to a four-fold dilution using 1X Tris-EDTA buffer followed by amplification on OpenArray plates specifically designed for the 754 target miRNAs. This was achieved on the 12K Flex Real-Time PCR system (Thermo Fisher Scientific). The entire procedure was done according to the manufacturer’s protocol. The −ΔΔCt analytical method was employed for relative quantification using mammalian U6 snRNA as the endogenous reference control.

#### 4.3.2 Gene expression analysis by real-time PCR

The isolated RNA was employed for quantitative real-time PCR analyses. The concentration of RNA was ascertained using the Eppendorf BioSpectrometer, while its quality was verified via electrophoresis on a 1% agarose gel. cDNA was synthesized using the High-Capacity cDNA Reverse Transcription Kit, according to the manufacturer’s guidelines. Quantitative PCR was executed using SYBR Green chemistry. The −ΔΔCt method was employed for relative quantification with data normalized to the mammalian housekeeping gene ACTB/GAPDH as an endogenous control. Primer sequences employed for real-time PCR are provided in Supplementary Table S10.

#### 4.3.3 Bioinformatic analysis

DEPs underwent GO annotation through DAVID, encompassing cellular components, molecular activities, and biological mechanisms, KEGG pathways, and Reactome pathways. The functional annotation and construction of protein–protein interaction networks were also done using the Metascape database (https://metascape.org/). STRING version 11.0 software facilitated the construction of PPI networks. The TargetScan platform was utilized to establish the interaction between detected altered miRNAs and the identified DEPs in NA-exposed NPCs. GO and KEGG were employed to explore pathways and diverse biological and molecular roles. The analysis of altered miRNAs based on GO annotations and KEGG pathways was performed using the DIANA-miRPath V2.0 tool (Http://www.microrna.gr/miRPathv2).

## 5 Conclusion

In the present investigation, arsenic exposure results in oxidative stress, mitochondrial dysfunction, apoptosis, autophagy, and increased expression of neurodegeneration-associated proteins and miRNAs in NPCs. These changes are primarily attributed to arsenic-induced neurotoxicity in NPCs due to structural and functional abnormalities, with oxidative stress playing a pivotal role. The exposure to NOB mitigated the neurotoxic effects of sodium arsenate, particularly restoring protein expression involved in cell homeostasis. The presence of NOB led to a reversal in these protein expression trajectories, particularly the proteins playing pivotal roles in pathways such as mitochondrial and oxidative stress response, neurodegeneration, ubiquitin–proteasome system, chaperone-mediated protein folding, and programmed cell death pathways. The RT-PCR data further confirmed that NOB restores apoptosis, autophagy, and stress markers to the basal level in NA-exposed NPCs. In addition to the protein and mRNA analysis, miRNA profiling revealed a significant dysregulation of four miRNAs (miR-509-3-5p, miR-302b, miR-302c, and miR-589) due to NA exposure. NOB compensated for the observed dysregulation in the expression of these miRNAs in NA-exposed NPCs. High-throughput proteomics and miRNA profiling elucidated the mechanisms of arsenic toxicity in NPCs and the protective role of NOB. In summary, we identified several pathways and underlying mechanisms through which NOB demonstrates its potential as a therapeutic agent against chemical-induced neurotoxicity. Future *in vivo* studies should delve deeper into these mechanisms to reveal more specific targets to assess the potency and efficacy of NOB in combating chemical-induced neurotoxicity.

## Data Availability

The original contributions presented in the study are included in the article/Supplementary Material; further inquiries can be directed to the corresponding author. The data presented in the study are deposited in the MassIVE repository, accession number PXD047456.
