# Supplementary material for: A protein–miRNA biomic analysis approach to explore neuroprotective potential of nobiletin in human neural progenitor cells (hNPCs)
Source: Front Pharmacol. 2024 Jan 25;15:1343569. doi: 10.3389/fphar.2024.1343569 (PMC10860404; doi:10.3389/fphar.2024.1343569)
Supplement: Supplementary file 1 [file Table1.DOCX]

**Supplementary Table S1**

**A. Up-regulated Proteins**

| **S. No.** | **Accession** | **Description** | **Gene Symbol** | **Unique Peptides** | **MW [kDa]** | **Coverage [%]** | **Log_2_ Fold change: (NA) / (CON)** | **Log_2_ Fold change: (NA_NOB) / (CON)** | **Log2 Fold change: (NA_NOB) / (NA)** |
| --- | --- | --- | --- | --- | --- | --- | --- | --- | --- |
| 1 | Q96DI8 | Heme oxygenase | HMOX1 | 3 | 32.8 | 17 | 5.99 | 0.78 | -5.89 |
| 2 | G1UI32 | Pyridoxal kinase | PDXK | 2 | 19 | 13 | 5.97 | 4.17 | -1.82 |
| 3 | A0A2R8Y761 | Ribonuclease H2 subunit B | RNASEH2B | 2 | 32.8 | 9 | 5.7 | 4.9 | -0.8 |
| 4 | Q8IZ83 | Aldehyde dehydrogenase family 16 member A1 | ALDH16A1 | 3 | 85.1 | 12 | 5.34 | 5.14 | -0.22 |
| 5 | G9FP35 | Guanine nucleotide binding protein | GNAI1 | 2 | 42.1 | 10 | 5.14 | 6.46 | 1.47 |
| 6 | J3QRM1 | CDK5 regulatory subunit-associated protein 3 | CDK5RAP3 | 2 | 30.2 | 22 | 5.13 | 3.12 | -1.95 |
| 7 | A4UJ43 | Glutathione S-transferase | GSTM3 | 2 | 24.8 | 13 | 5.01 | 3.83 | -1.16 |
| 8 | E9PEI0 | Cell division cycle-associated protein 2 | CDCA2 | 2 | 110.9 | 4 | 4.75 | 2.41 | -2.34 |
| 9 | Q6P486 | Nucleoporin 205 protein | NUP205 | 3 | 70 | 12 | 4.66 | 5.21 | 0.73 |
| 10 | Q04446 | 1,4-alpha-glucan-branching enzyme | GBE1 | 2 | 80.4 | 7 | 4.59 | 4.9 | 0.31 |
| 11 | H9KV28 | Protein diaphanous homolog 1 | DIAPH1 | 3 | 136.8 | 6 | 4.49 | 4.16 | -0.42 |
| 12 | D3DSY9 | Farnesyltransferase, CAAX box, alpha, isoform | FNTA | 3 | 52.6 | 14 | 4.48 | 4.56 | 0.52 |
| 13 | C9JIZ0 | U6 snRNA-associated Sm-like protein LSm8 | LSM8 | 3 | 8.1 | 65 | 4.44 | 3.83 | -0.6 |
| 14 | A0AVL1 | ADAM Metallopeptidase domain 9 protein | ADAM9 | 2 | 78.5 | 6 | 4.37 | 4.57 | 0.55 |
| 15 | Q96DE0 | U8 snoRNA-decapping enzyme | NUDT16 | 3 | 21.3 | 26 | 4.36 | 5.84 | 1.96 |
| 16 | Q9ULT8 | E3 ubiquitin-protein ligase HECTD1 | HECTD1 | 5 | 289.2 | 4 | 4.28 | 3.13 | -1.15 |
| 17 | E9PLW6 | 4'-phosphopantetheinyl transferase | AASDHPPT | 2 | 22.2 | 15 | 4.15 | 3.29 | -0.86 |
| 18 | B0I1S0 | DYNC2H1 variant protein | DYNC2H1 | 4 | 492.3 | 2 | 4.14 | 4.11 | -0.02 |
| 19 | A8KA19 | Exportin-T | XPOT | 4 | 109.8 | 9 | 4.12 | 2.34 | -1.79 |
| 20 | Q96QC0 | Serine/threonine-protein phosphatase 1 regulatory subunit 10 | PPP1R10 | 3 | 99 | 7 | 4.07 | 5.45 | 1.38 |
| 21 | P57740 | Nuclear pore complex protein Nup107 | NUP107 | 4 | 106.3 | 10 | 3.83 | 2.6 | -1.23 |
| 22 | F8VXI9 | ARF GTPase-activating protein GIT2 | GIT2 | 2 | 78.8 | 9 | 3.71 | 2.76 | -0.94 |
| 23 | H7C1Z3 | Nucleolar protein 10 | NOL10 | 2 | 28.3 | 23 | 3.63 | 4.17 | 1.27 |
| 24 | A0A494C0D4 | Protoporphyrinogen oxidase | PPOX | 2 | 40.3 | 14 | 3.57 | 3.18 | -0.39 |
| 25 | Q5T440 | Putative transferase CAF17, mitochondrial | IBA57 | 3 | 38.1 | 16 | 3.55 | 2.31 | -1.25 |
| 26 | E7ESZ7 | NADH dehydrogenase [ubiquinone] 1 alpha subcomplex subunit 10, mitochondrial | NDUFA10 | 2 | 44.7 | 12 | 3.51 | 3.2 | -0.31 |
| 27 | B4E318 | BRCA2 and CDKN1A-interacting protein | BCCIP | 3 | 29.8 | 17 | 3.5 | 2.21 | -1.29 |
| 28 | Q9NQG5 | Regulation of nuclear pre-mRNA domain-containing protein 1B | RPRD1B | 3 | 36.9 | 19 | 3.44 | 2.23 | -1.2 |
| 29 | Q9C0C2 | 182 kDa tankyrase-1-binding protein | TNKS1BP1 | 5 | 181.7 | 8 | 3.43 | 2.55 | -0.88 |
| 30 | B1AA17 | Vacuolar protein sorting-associated protein 53 homolog | HCCS1 | 2 | 76.3 | 8 | 3.36 | 1.34 | -2.03 |
| 31 | O00401 | Actin nucleation-promoting factor WASL | WASL | 4 | 54.8 | 18 | 3.35 | 1.77 | -1.58 |
| 32 | B2RWN5 | HEAT repeat-containing protein 1 | GNAQ | 5 | 242.1 | 4 | 3.33 | 3.61 | 0.28 |
| 33 | A0A140VKC0 | Testis tissue sperm-binding protein Li 37a | UAP1 | 2 | 57 | 10 | 3.21 | 2.57 | -0.64 |
| 34 | B4DMP4 | Protein VAC14 homolog | VAC14 | 2 | 80.1 | 7 | 3.18 | 3.24 | 0.06 |
| 35 | Q5QPM7 | Proteasome inhibitor PI31 subunit | PSMF1 | 2 | 28.9 | 16 | 3.17 | 2.66 | -0.51 |
| 36 | Q5T8U7 | Surfeit locus protein 4 | SURF4 | 2 | 21.1 | 12 | 3.16 | 3.21 | 0.05 |
| 37 | Q8IWY9 | Codanin-1 | CDAN1 | 2 | 134 | 4 | 3.15 | 3.82 | 0.67 |
| 38 | B4E1L0 | Adenylosuccinate synthetase isozyme 2 | ADSS | 2 | 47.9 | 6 | 3.14 | 3.08 | -0.06 |
| 39 | Q5SRE5 | Nucleoporin NUP188 | NUP188 | 2 | 195.9 | 2 | 3.13 | 2 | -1.13 |
| 40 | Q9BU02 | Thiamine-triphosphatase | THTPA | 2 | 25.6 | 13 | 3.12 | 2.82 | -0.3 |
| 41 | E9PDP5 | Ankyrin repeat and KH domain-containing protein 1 | ANKHD1 | 6 | 166.6 | 9 | 3.1 | 2.65 | -0.45 |
| 42 | A0A1W2PPB7 | Kinase D-interacting substrate of 220 kDa | KIDINS220 | 2 | 146.4 | 3 | 3.04 | 2.37 | -0.67 |
| 43 | Q59GA5 | Insulysin variant | IDE | 2 | 69.4 | 8 | 3.02 | 1.36 | -1.65 |
| 44 | A6NHJ8 | Quinone oxidoreductase-like protein 1 | CRYZL1 | 2 | 36.1 | 15 | 3.01 | 3.46 | 0.44 |
| 45 | O75663 | TIP41-like protein | TIPRL | 2 | 31.4 | 16 | 2.98 | 2.44 | -0.54 |
| 46 | M0R0P8 | Unconventional myosin-IXb | MYO9B | 2 | 243.2 | 1 | 2.86 | 3.16 | 0.3 |
| 47 | J3KRA9 | Non-specific serine/threonine protein kinase | SMG1 | 2 | 398.6 | 2 | 2.85 | 1.24 | -1.61 |
| 48 | Q96T23 | Remodeling and spacing factor 1 | RSF1 | 2 | 163.7 | 5 | 2.83 | 2.12 | -0.71 |
| 49 | Q53GY1 | BCL2-associated athanogene 3 variant | BAG3 | 3 | 61.6 | 9 | 2.8 | 1.23 | -1.57 |
| 50 | B2RB57 | Ubiquitin-like modifier-activating enzyme ATG7 | ATG7 | 2 | 77.9 | 6 | 2.8 | 3.6 | 0.8 |
| 51 | O60518 | Ran-binding protein 6 | RANBP6 | 4 | 124.6 | 9 | 2.79 | 2.84 | 0.05 |
| 52 | Q8TEM1 | Nuclear pore membrane glycoprotein 210 | NUP210 | 3 | 205 | 3 | 2.78 | 2.48 | -0.3 |
| 53 | Q53GN7 | Mitochondrial ribosomal protein S30 variant | MRPS30 | 2 | 50.3 | 8 | 2.74 | 2.96 | 0.22 |
| 54 | A0A024R4A5 | Trinucleotide repeat containing 15, isoform | TNRC15 | 6 | 149.9 | 8 | 2.72 | 1.94 | -0.78 |
| 55 | A8JZZ8 | BCS1-like protein | BCS1L | 2 | 47.5 | 7 | 2.69 | 3.39 | 0.69 |
| 56 | Q9H9B7 | Coatomer subunit gamma | COPG | 3 | 89.3 | 9 | 2.65 | 3.46 | 0.81 |
| 57 | G1UI17 | 4-alpha-glucanotransferase | AGL | 2 | 143.6 | 4 | 2.64 | 3.13 | 0.49 |
| 58 | D6REA0 | Glutamyl-tRNA(Gln) amidotransferase subunit B, mitochondrial | GATB | 6 | 57.6 | 22 | 2.62 | 2.58 | -0.04 |
| 59 | A0A384MDW7 | Enoyl Coenzyme A hydratase, short chain, 1, mitochondrial | ECHS1 | 5 | 31.4 | 29 | 2.52 | 2.31 | -0.21 |
| 60 | A5D8Z4 | WNK1 protein | WNK1 | 5 | 159.4 | 7 | 2.51 | 3.12 | 0.61 |
| 61 | Q59ET9 | Mevalonate kinase | MVK | 3 | 44.7 | 13 | 2.48 | 3.31 | 0.83 |
| 62 | Q9P0N4 | HSPC245 | HSPC245 | 2 | 14.1 | 46 | 2.46 | 1.42 | -1.04 |
| 63 | Q5VZ89 | DENN domain-containing protein 4C | DENND4C | 3 | 212.6 | 3 | 2.44 | 1.88 | -0.56 |
| 64 | G3V5V3 | Nuclear export mediator factor NEMF | NEMF | 2 | 113.9 | 3 | 2.43 | 0.9 | -1.53 |
| 65 | Q96IR5 | PIGS protein | PIGS | 5 | 52.8 | 23 | 2.36 | 0.93 | -1.43 |
| 66 | Q9NXS2 | Glutaminyl-peptide cyclotransferase-like protein | QPCTL | 2 | 42.9 | 5 | 2.35 | 2.16 | -0.19 |
| 67 | E9PK47 | Alpha-1,4 glucan phosphorylase | PYGL | 4 | 94 | 9 | 2.34 | 2.73 | 0.39 |
| 68 | A0A6Q8PFJ4 | Mitofusin-2 | MFN2 | 2 | 91.7 | 6 | 2.33 | 3.46 | 1.12 |
| 69 | A0A0U1RRM8 | Fermitin family homolog 2 | FERMT2 | 4 | 61.9 | 19 | 2.33 | 1.14 | -1.2 |
| 70 | Q9Y3B5 | CGI-111 protein | CGI-111 | 2 | 22.1 | 24 | 2.33 | 1.84 | -0.49 |
| 71 | H3BST1 | Biogenesis of lysosome-related organelles complex 1 subunit 6 | BLOC1S6 | 2 | 20.3 | 24 | 2.33 | 1.67 | -0.66 |
| 72 | Q13084 | 39S ribosomal protein L28, mitochondrial | MRPL28 | 2 | 30.1 | 18 | 2.33 | 2.61 | 0.29 |

**B. For Down-regulated Proteins**

| **S. No.** | **Accession** | **Description** | **Gene Symbol** | **Unique Peptides** | **MW [kDa]** | **Coverage [%]** | **Log_2_ Fold change:**  **(NA) / (CON)** | **Log_2_ Fold change: (NA_NOB) / (CON)** | **Log_2_ Fold change: (NA_NOB) / (NA)** |
| --- | --- | --- | --- | --- | --- | --- | --- | --- | --- |
| 1 | D3DRP5 | Chromosome 9 open reading frame 19, isoform | C9orf19 | 3 | 37.8 | 13 | -6.64 | -3.32 | 4.56 |
| 2 | P46087 | Probable 28S rRNA (cytosine(4447)-C(5))-methyltransferase | NOP2 | 3 | 89.2 | 12 | -6.64 | -5.85 | 0.79 |
| 3 | O95881 | Thioredoxin domain-containing protein 12 | TXNDC12 | 2 | 19.2 | 23 | -6.35 | -5.07 | 1.51 |
| 4 | Q15293 | Reticulocalbin-1 | RCN1 | 4 | 38.9 | 27 | -6.27 | -5.51 | 0.75 |
| 5 | B4DJQ5 | Glucosidase 2 subunit beta | GluIIβ | 3 | 60.1 | 16 | -6.31 | -6.23 | -0.62 |
| 6 | K7EM56 | 40S ribosomal protein S15 | RPS15 | 3 | 13 | 45 | -6.13 | -6.64 | -0.94 |
| 7 | A0A087X2D5 | 39S ribosomal protein L45, mitochondrial | MRPL45 | 2 | 35.3 | 10 | -6.15 | -5.23 | 1.01 |
| 8 | V9HW35 | Peroxiredoxin-5 | HEL-S-55 | 3 | 17 | 25 | -5.98 | -4.67 | 0.84 |
| 9 | P16930 | Fumarylacetoacetase | FAH | 4 | 46.3 | 21 | -5.86 | -4.67 | 1.24 |
| 10 | I3NI32 | Dihydroorotate dehydrogenase (quinone), mitochondrial | DHODH | 2 | 42.6 | 12 | -5.87 | -4.92 | 1.11 |
| 11 | E9PQ57 | Rae1 protein homolog | RAE1 | 4 | 47.8 | 27 | -5.68 | -4.77 | 1.12 |
| 12 | A0A087WUT0 | Myelin expression factor 2 | MYEF2 | 3 | 58.6 | 16 | -5.57 | -5.18 | 0.85 |
| 13 | A0A4Z1 | CADM1 protein | CADM1 | 4 | 42.7 | 23 | -5.59 | -3.61 | 1.98 |
| 14 | Q53FA7 | Quinone oxidoreductase PIG3 | TP53I3 | 2 | 35.5 | 16 | -5.56 | -3.22 | 2.43 |
| 15 | M0QWZ7 | Seryl-tRNA synthetase | SARS2 | 2 | 58.1 | 9 | -5.56 | -4.77 | 0.99 |
| 16 | Q9NTX5 | Ethylmalonyl-CoA decarboxylase | ECHDC1 | 2 | 33.7 | 12 | -5.5 | -4.35 | 1.14 |
| 17 | Q9UFN0 | Protein NipSnap homolog 3A | NIPSNAP3A | 3 | 28.4 | 26 | -5.49 | -4.82 | 0.22 |
| 18 | Q05DH1 | Proteasome subunit alpha type | PSMA7 | 6 | 26.7 | 34 | -5.44 | -4.31 | 0.85 |
| 19 | Q6NVC0 | SLC25A5 protein | SLC25A5 | 2 | 35.3 | 12 | -5.42 | -4.22 | 1.2 |
| 20 | H0Y3M2 | BUB3-interacting and GLEBS motif-containing protein ZNF207 | ZNF207 | 2 | 21.3 | 15 | -5.37 | -5.82 | -0.7 |
| 21 | Q14DU5 | Non-specific serine/threonine protein kinase | ROCK2 | 2 | 82.1 | 4 | -5.32 | -5.3 | -0.14 |
| 22 | A0A2R8Y4Y7 | Paraplegin | SPG7 | 3 | 83.6 | 10 | -5.24 | -4.43 | 0.95 |
| 23 | A0A6Q8KRG2 | Golgin subfamily A member 2 | GOLGA2 | 6 | 111.6 | 11 | -5.25 | -4.96 | 0.3 |
| 24 | Q5T7C4 | High mobility group protein B1 | HMGB1 | 2 | 18.3 | 18 | -5.25 | -3.36 | 1.92 |
| 25 | A0A2L0RI76 | Butyryl-CoA dehydrogenase | IVD | 3 | 46.6 | 20 | -5.28 | -5.31 | 0.57 |
| 26 | G8JLA1 | Retinol dehydrogenase 13 | RDH13 | 2 | 24 | 10 | -5.2 | -3.89 | 1.84 |
| 27 | H0Y8G5 | Heterogeneous nuclear ribonucleoprotein D0 | HNRNPD | 5 | 29.6 | 14 | -5.19 | -4.37 | 0.58 |
| 28 | Q7Z7M4 | Superoxide dismutase | SOD2 | 5 | 23.7 | 37 | -5.23 | -5.7 | -0.71 |
| 29 | A0A2R8YEA7 | Actin, cytoplasmic 1 | ACTB | 2 | 17.5 | 69 | -5.24 | -2.67 | 2.61 |
| 30 | A0A096LNH5 | Glutamine amidotransferase-like class 1 domain-containing protein 3B, mitochondrial | GATD3B | 3 | 19.4 | 28 | -5.14 | -4.07 | 1.07 |
| 31 | B3KMB1 | Structural maintenance of chromosomes protein | SMC2 | 6 | 135.5 | 12 | -5.18 | -4.96 | 0.13 |
| 32 | K7EPJ5 | E3 ubiquitin-protein ligase MGRN1 | MGRN1 | 2 | 65 | 14 | -5.18 | -4.47 | 0.69 |
| 33 | Q9BXK5 | Bcl-2-like protein 13 | BCL2L13 | 6 | 52.7 | 30 | -5.18 | -4.01 | 1.17 |
| 34 | Q12972 | Nuclear inhibitor of protein phosphatase 1 | PPP1R8 | 4 | 38.5 | 21 | -5.15 | -4.38 | 0.77 |
| 35 | A0A384P5V3 | Epididymis secretory sperm binding protein | ELSPBP1 | 4 | 72.9 | 7 | -5.16 | -4.96 | 0.19 |
| 36 | Q13867 | Bleomycin hydrolase | BLMH | 3 | 52.5 | 14 | -5.05 | -4.41 | 0.64 |
| 37 | Q5RKV6 | Exosome complex component MTR3 | EXOSC6 | 2 | 28.2 | 19 | -5.03 | -4.93 | 0.51 |
| 38 | H3BMM9 | RNA-binding protein with serine-rich domain 1 | RNPS1 | 2 | 31.7 | 15 | -5.03 | -4.12 | 0.9 |
| 39 | Q9NPH2 | Inositol-3-phosphate synthase 1 | ISYNA1 | 3 | 61 | 12 | -4.94 | -2.18 | 2.77 |
| 40 | Q13268 | Dehydrogenase/reductase SDR family member 2, mitochondrial | DHRS2 | 3 | 29.9 | 31 | -4.99 | -3.59 | 1.4 |
| 41 | Q8IXI1 | Mitochondrial Rho GTPase 2 | RHOT2 | 5 | 68.1 | 21 | -4.91 | -3.96 | 0.95 |
| 42 | B5BTZ8 | Small nuclear ribonucleoprotein polypeptide B'' | SNRPB2 | 2 | 25.4 | 10 | -4.93 | -4.02 | 0.91 |
| 43 | A0A0S2Z4Z6 | Serine/arginine repetitive matrix 1 isoform 2 | SRRM1 | 2 | 103.9 | 5 | -4.94 | -3.23 | 2.28 |
| 44 | Q53GF5 | Proteasome subunit alpha type | PSMA1 | 7 | 25.8 | 43 | -4.9 | -5.12 | -0.22 |
| 45 | Q96D15 | Reticulocalbin-3 | RCN3 | 3 | 37.5 | 22 | -4.92 | -4.38 | 0.54 |
| 46 | O43865 | S-adenosylhomocysteine hydrolase-like protein 1 | AHCYL1 | 6 | 58.9 | 20 | -4.88 | -5.02 | -0.1 |
| 47 | O75475 | PC4 and SFRS1-interacting protein | PSIP1 | 5 | 60.1 | 17 | -4.87 | -4.4 | 0.47 |
| 48 | O15069 | NAC-alpha domain-containing protein 1 | NACAD | 2 | 161 | 3 | -4.86 | -4.64 | -0.16 |
| 49 | P00441 | Superoxide dismutase [Cu-Zn] | SOD1 | 4 | 15.9 | 54 | -4.86 | -5.03 | -0.17 |
| 50 | P28072 | Proteasome subunit beta type-6 | PSMB6 | 3 | 25.3 | 33 | -4.85 | -4.19 | 0.66 |
| 51 | Q01780 | Exosome component 10 | EXOSC10 | 3 | 100.8 | 6 | -4.85 | -4.43 | 0.13 |
| 52 | B3KQT9 | Protein disulfide-isomerase | PDI | 10 | 54.1 | 32 | -4.85 | -4.45 | 0.46 |
| 53 | Q6IAL5 | Succinate--CoA ligase [ADP/GDP-forming] subunit alpha, mitochondrial | SUCLG1 | 2 | 35 | 21 | -4.85 | -5.06 | 0.4 |
| 54 | C9J126 | Cadherin-2 | CDH2 | 8 | 90.6 | 19 | -4.82 | -5.1 | -0.21 |
| 55 | D6RBZ0 | Heterogeneous nuclear ribonucleoprotein A/B | HNRNPAB | 3 | 35.7 | 10 | -4.86 | -3.35 | 1.51 |
| 56 | Q96JM3 | Chromosome alignment-maintaining phosphoprotein 1 | CHAMP1 | 2 | 89 | 4 | -4.86 | -3.09 | 2.11 |
| 57 | G3V3H3 | Kinesin light chain | KLC1 | 3 | 68.7 | 8 | -4.85 | -1.89 | 2.96 |
| 58 | B4DLP6 | Serine/threonine-protein phosphatase | PPP | 4 | 54.9 | 12 | -4.79 | -3.95 | 0.84 |
| 60 | A0A5F9ZHC5 | Aminoacyl tRNA synthase complex-interacting multifunctional protein 1 | AIMP1 | 3 | 29.9 | 27 | -4.78 | -5.22 | -0.42 |
| 61 | B4DDB6 | Heterogeneous nuclear ribonucleoprotein A3, isoform | HNRNPA3 | 6 | 37 | 16 | -4.78 | -3.92 | 1.18 |
| 62 | A0A0A0MSH6 | Lipid droplet-associated hydrolase | LDAH | 2 | 32.7 | 10 | -4.77 | -3.58 | 1.18 |
| 63 | P19784 | Casein kinase II subunit alpha | CSNK2A2 | 2 | 41.2 | 8 | -4.71 | -5.93 | -1.65 |
| 64 | J3QRS3 | Myosin regulatory light chain 12A | MYL12A | 3 | 20.4 | 33 | -4.71 | -4.24 | 0.46 |
| 65 | P11021 | Endoplasmic reticulum chaperone BiP | HSPA5 | 3 | 72.3 | 37 | -4.72 | -2.15 | 2.57 |
| 66 | B3KRM8 | Component 3 of promoter of RISC | TSN | 2 | 20.9 | 8 | -4.67 | -3.85 | 0.82 |
| 67 | P49756 | RNA-binding protein 25 | RBM25 | 3 | 100.1 | 5 | -4.64 | -2.01 | 2.63 |
| 68 | Q96B43 | Methionine aminopeptidase | METAP | 2 | 28.2 | 14 | -4.65 | -4.84 | -0.19 |
| 69 | P60900 | Proteasome subunit alpha type-6 | PSMA6 | 4 | 27.4 | 26 | -4.64 | -3.94 | 0.88 |
| 70 | A0A1S5RA93 | Bifunctional polynucleotide phosphatase/kinase | PNKP | 5 | 57 | 22 | -4.62 | -4.04 | 0.58 |
| 71 | Q9Y2V2 | Calcium-regulated heat-stable protein 1 | CARHSP1 | 2 | 15.9 | 35 | -4.61 | -1.57 | 3.04 |
| 72 | P08754 | Guanine nucleotide-binding protein G(i) subunit alpha-3 | GNAI3 | 4 | 40.5 | 24 | -4.6 | -4.53 | 0.13 |
| 73 | Q14789 | Golgin subfamily B member 1 | GOLGB1 | 5 | 375.8 | 4 | -4.62 | -2.83 | 1.79 |
| 74 | P07602 | Prosaposin | PSAP | 9 | 58.1 | 28 | -4.56 | -4.19 | 0.37 |
| 75 | C9JTN7 | Nucleolysin TIA-1 isoform p40 | TIA1 | 2 | 31.6 | 26 | -4.57 | -4.61 | -0.04 |
| 76 | Q8IX12 | Cell division cycle and apoptosis regulator protein 1 | CCAR1 | 4 | 132.7 | 7 | -4.52 | -4.23 | 0.38 |
| 77 | A0A087WWS1 | THO complex subunit 1 | THOC1 | 3 | 75.6 | 11 | -4.53 | -4.95 | -0.42 |
| 78 | Q9NX24 | H/ACA ribonucleoprotein complex subunit 2 | NHP2 | 2 | 17.2 | 37 | -4.5 | -2.21 | 3.23 |
| 79 | B4DRV4 | Osteonectin | SPARC | 2 | 24.7 | 16 | -4.51 | -4.39 | 0.11 |
| 80 | A0A384MTQ5 | Terpene cyclase/mutase family member | TCs | 3 | 83.2 | 4 | -4.49 | -3.64 | 0.34 |
| 81 | Q05DK5 | ADD2 protein | ADD2 | 2 | 78.8 | 8 | -4.48 | -3.04 | 0.71 |
| 82 | F5H6I7 | Atlastin-3 | ATL3 | 4 | 58.7 | 16 | -4.47 | -3.92 | 0.54 |
| 83 | A0A499FI48 | Protein disulfide-isomerase | PDIA4 | 11 | 73 | 27 | -4.47 | -4.68 | -0.21 |
| 84 | B3KNB9 | HBS1-like protein | HBS1L | 2 | 75.4 | 6 | -4.43 | -3.88 | 0.55 |
| 85 | B4DWZ4 | Flap endonuclease 1 | FEN1 | 3 | 38.8 | 19 | -4.45 | -4.87 | -0.68 |
| 86 | D3DNF9 | Sodium/potassium-transporting ATPase subunit beta | ATP1B3 | 4 | 29.7 | 32 | -4.43 | -3.27 | 1.16 |
| 87 | A0A024R137 | SWI/SNF related, matrix associated, actin dependent regulator of chromatin, subfamily d, member 1, isoform | SMARCD1 | 3 | 66 | 9 | -4.43 | -3.21 | 1.22 |
| 88 | A0A087X2D0 | Serine/arginine-rich-splicing factor 3 | SRSF3 | 2 | 10.3 | 22 | -4.43 | -3.91 | 0.72 |
| 89 | Q96PU8 | KH domain-containing RNA-binding protein QKI | QKI | 4 | 37.6 | 16 | -4.42 | -4.41 | 0.02 |
| 90 | E9PCX7 | Proton-translocating NAD(P)(+) transhydrogenase | NNT | 5 | 99.7 | 13 | -4.35 | -2.46 | 1.9 |
| 91 | A0A024R2Z6 | Guanine nucleotide-binding protein-like 3 | GNL3 | 4 | 60.5 | 12 | -4.35 | -4.01 | 0.58 |
| 92 | P30048 | Thioredoxin-dependent peroxide reductase, mitochondrial | PRDX3 | 4 | 27.7 | 26 | -4.36 | -3.9 | 0.45 |
| 93 | Q9H1A4 | Anaphase-promoting complex subunit 1 | ANAPC1 | 3 | 216.4 | 4 | -4.35 | -1.69 | 2.67 |
| 94 | A0A1B0GV47 | Kinesin-like protein KIF21A | KIF21A | 4 | 184.6 | 7 | -4.32 | -3.7 | 0.62 |
| 95 | P61106 | Ras-related protein Rab-14 | RAB14 | 2 | 23.9 | 21 | -4.32 | -2.92 | 1.4 |
| 96 | Q4LE43 | Phosphoinositide phospholipase C | PLCG1 | 5 | 161.2 | 7 | -4.33 | -3.51 | 0.82 |
| 97 | C9JD32 | 60S ribosomal protein L23 | RPL23 | 4 | 9.7 | 44 | -4.33 | -4.7 | 0.16 |
| 98 | H3BRV9 | Nuclear transport factor 2 | NUTF2 | 2 | 12.2 | 40 | -4.28 | -5.44 | -1.15 |
| 99 | O94760 | N(G),N(G)-dimethylarginine dimethylaminohydrolase 1 | DDAH1 | 4 | 31.1 | 28 | -4.29 | -4.73 | -0.57 |
| 100 | Q53HN4 | DNAation factor, 45kDa, alpha polypeptide isoform 1 variant | DFAP | 4 | 36.6 | 21 | -4.29 | -3.71 | 0.58 |
| 101 | B3KUQ5 | Thioredoxin-disulfide reductase | TrxR | 7 | 50 | 33 | -4.28 | -4.85 | -0.57 |
| 102 | Q59EH3 | Low molecular weight cytosolic acid phosphatase | ACP1 | 2 | 18.7 | 25 | -4.27 | -4.21 | -0.44 |
| 103 | A8K8K1 | RNA 3'-terminal phosphate cyclase | RTCA | 4 | 39.3 | 23 | -4.27 | -4.37 | -0.1 |
| 104 | F8VZQ9 | SAP domain-containing ribonucleoprotein | SARNP | 2 | 24.1 | 20 | -4.26 | -3.79 | 1.38 |
| 105 | P23229 | Integrin alpha-6 | ITGA6 | 10 | 126.5 | 13 | -4.24 | -3.2 | 1.03 |
| 106 | Q9Y3B4 | Splicing factor 3B subunit 6 | SF3B6 | 2 | 14.6 | 21 | -4.24 | -4.21 | 0.71 |
| 107 | A0A087X020 | Ribosome maturation protein SBDS | SBDS | 2 | 28.8 | 16 | -4.21 | -3.06 | 1.15 |
| 108 | A0A087X2D8 | C-Jun-amino-terminal kinase-interacting protein 4 | SPAG9 | 2 | 145.1 | 2 | -4.22 | -3.63 | 0.58 |
| 109 | E9PB61 | THO complex subunit 4 | ALYREF | 4 | 27.5 | 28 | -4.21 | -3.92 | 0.29 |
| 110 | A8QI98 | DIS3 | DIS3 | 4 | 109 | 8 | -4.22 | -3.83 | 0.39 |
| 111 | H3BNT7 | 26S proteasome non-ATPase regulatory subunit 7 | PSMD7 | 5 | 20.1 | 48 | -4.21 | -3.11 | 1.1 |
| 112 | P62879 | Guanine nucleotide-binding protein G(I)/G(S)/G(T) subunit beta-2 | GNB2 | 5 | 37.3 | 48 | -4.21 | -2.71 | 1.5 |
| 113 | Q9BZK7 | F-box-like/WD repeat-containing protein TBL1XR1 | TBL1XR1 | 5 | 55.6 | 20 | -4.22 | -2.87 | 1.35 |
| 114 | B3KUZ8 | Aspartate aminotransferase | GOT1 | 7 | 41.3 | 30 | -4.19 | -4.52 | -0.32 |
| 115 | Q9BV44 | tRNA (guanine(6)-N2)-methyltransferase THUMP3 | THUMPD3 | 2 | 57 | 13 | -4.17 | -2.53 | 1.64 |
| 116 | A0A6Q8PGE6 | Ras-related protein Rab-7a | RAB7A | 5 | 21 | 43 | -4.19 | -3.16 | 1.03 |
| 117 | Q5T6H7 | Xaa-Pro aminopeptidase 1 | XPNPEP1 | 5 | 62.1 | 23 | -4.18 | -3.15 | 1.03 |
| 118 | Q9BVA1 | Tubulin beta-2B chain | TUBB2B | 6 | 49.9 | 66 | -4.17 | -0.62 | 3.55 |
| 119 | B3KT18 | Glutamate dehydrogenase | GLUD | 2 | 55.9 | 8 | -4.19 | -4.3 | -0.11 |
| 120 | M0R2A0 | ER membrane protein complex subunit 10 | EMC10 | 3 | 39 | 12 | -4.19 | -3.13 | 1.06 |
| 121 | Q9UBW7 | Zinc finger MYM-type protein 2 | ZMYM2 | 3 | 154.8 | 7 | -4.17 | -3.44 | 0.73 |
| 122 | Q13263 | Transcription intermediary factor 1-beta | TRIM28 | 11 | 88.5 | 23 | -4.16 | -3.98 | 0.18 |
| 123 | Q96DB5 | Regulator of microtubule dynamics protein 1 | RMDN1 | 4 | 35.8 | 20 | -4.16 | -4.17 | 0 |
| 124 | H0YNG3 | Signal peptidase complex catalytic subunit SEC11 | SEC11A | 2 | 18.6 | 7 | -4.15 | -1.87 | 2.27 |
| 125 | B4DNB9 | AP-2 complex subunit mu | AP2M1 | 3 | 52.3 | 12 | -4.14 | -3.4 | 0.74 |
| 126 | P14550 | Aldo-keto reductase family 1 member A1 | AKR1A1 | 3 | 36.6 | 18 | -4.13 | -3.21 | 0.91 |
| 127 | Q9UN70 | Protocadherin gamma-C3 | PCDHGC3 | 4 | 101 | 5 | -4.13 | -1.93 | 2.2 |
| 128 | A0A087WYV9 | Cytochrome c oxidase assembly protein COX16 homolog, mitochondrial | SYNJ2BP-COX16 | 2 | 20.5 | 23 | -4.12 | -2.64 | 1.49 |
| 129 | A0A0A0MRM8 | Unconventional myosin-6 | MYO6 | 3 | 144.9 | 5 | -4.11 | -3.46 | 0.65 |
| 130 | B4DVQ5 | Eukaryotic translation initiation factor 3 subunit C | EIF3C | 2 | 103.6 | 5 | -4.12 | -3.69 | -0.22 |
| 131 | P14174 | Macrophage migration inhibitory factor | MIF | 3 | 12.5 | 33 | -4.11 | -5.24 | -1.13 |
| 132 | P49720 | Proteasome subunit beta type-3 | PSMB3 | 5 | 22.9 | 39 | -4.11 | -3.05 | 1.06 |
| 133 | A0A0F7NGI8 | Leucine rich repeat (In FLII) interacting protein 1, isoform | LRRFIP1 | 3 | 82.6 | 10 | -4.08 | -3.58 | 0.49 |
| 134 | Q53G71 | Calreticulin variant | CALR | 9 | 46.9 | 46 | -4.09 | -4.42 | -0.34 |
| 135 | Q09028 | Histone-binding protein RBBP4 | RBBP4 | 4 | 47.6 | 31 | -4.07 | -2.47 | 2.13 |
| 136 | Q9Y221 | 60S ribosome subunit biogenesis protein NIP7 homolog | NIP7 | 3 | 20.4 | 37 | -4.06 | -3.13 | 0.93 |
| 137 | A0A024R8Q1 | Glucosidase, alpha acid (Pompe disease, glycogen storage disease type II), isoform | GAA | 5 | 105.3 | 14 | -4.05 | -3.9 | 0.15 |
| 138 | A0A6I8PU89 | DnaJ homolog subfamily C member 7 | DNAJC7 | 3 | 39.5 | 16 | -4.07 | -3.22 | 0.85 |
| 139 | A0A2Z6ATB6 | Drebrin A | DBN1 | 6 | 76.3 | 18 | -4.03 | -3.53 | 0.5 |
| 140 | Q6FI54 | RAB5B protein | RAB5B | 3 | 23.7 | 27 | -4.04 | -3.41 | 0.63 |
| 141 | Q9BW83 | Intraflagellar transport protein 27 homolog | IFT27 | 2 | 20.5 | 22 | -4.04 | -4.33 | -0.3 |
| 142 | Q53HG2 | Complex I-49kD | NADH | 4 | 52.5 | 16 | -4.04 | -4.02 | 0.02 |
| 143 | Q9UPN7 | Serine/threonine-protein phosphatase 6 regulatory subunit 1 | PPP6R1 | 3 | 96.7 | 9 | -4.01 | -3.49 | 0.51 |
| 144 | B3KQQ0 | Peptidylprolyl isomerase | PPIs | 6 | 63 | 21 | -3.99 | -3.92 | -0.24 |
| 145 | Q59F20 | Neuropilin-1 variant | NRP1 | 4 | 99.6 | 7 | -3.98 | -3.89 | 0.05 |
| 146 | A0A0U1RR70 | Serine/threonine-protein kinase DCLK2 | DCLK2 | 4 | 78.3 | 8 | -4 | -4.15 | -0.15 |
| 147 | Q6P432 | Peptidyl-prolyl cis-trans isomerase | PPIG | 3 | 27.5 | 26 | -3.99 | -4.48 | -0.56 |
| 148 | H3BQZ7 | HCG2044799 | HNRNPUL2-BSCL2 | 6 | 84.6 | 17 | -3.96 | -2.45 | 1.5 |
| 149 | E2QRB3 | Pyrroline-5-carboxylate reductase | PYCR1 | 3 | 30.2 | 23 | -3.97 | -3.21 | 0.88 |
| 150 | Q2TAL8 | Transcriptional regulator QRICH1 | QRICH1 | 3 | 86.4 | 9 | -3.97 | -4.4 | -0.44 |
| 151 | H0Y7L6 | Transcription factor Sp3 | SP3 | 2 | 77.9 | 4 | -3.97 | -2.77 | 1.2 |
| 152 | A0A6Q8PHR6 | Tripartite motif-containing protein 2 | TRIM2 | 5 | 70.7 | 16 | -3.97 | -2.92 | 1.05 |
| 153 | C9J8H1 | V-type proton ATPase subunit E 1 | ATP6V1E1 | 3 | 23.5 | 21 | -3.97 | -3.38 | 0.59 |
| 154 | Q53FM7 | Complex I-30kD | NADH | 3 | 30.1 | 15 | -3.97 | -4.39 | -0.71 |
| 155 | P12107 | Collagen alpha-1(XI) chain | COL11A1 | 6 | 181 | 10 | -3.93 | -3.01 | 0.93 |
| 156 | Q10713 | Mitochondrial-processing peptidase subunit alpha | PMPCA | 6 | 58.2 | 23 | -3.95 | -2.78 | 1.17 |
| 157 | Q9H4A4 | Aminopeptidase B | RNPEP | 4 | 72.5 | 11 | -3.95 | -3.39 | 0.56 |
| 158 | Q8WVY7 | Ubiquitin-like domain-containing CTD phosphatase 1 | UBLCP1 | 3 | 36.8 | 19 | -3.94 | -2.97 | 1.68 |
| 159 | A0A2R8Y7Z0 | Catenin beta-1 | CTNNB1 | 13 | 85.3 | 33 | -3.95 | -4 | -0.05 |
| 160 | G5EA30 | CUG triplet repeat, RNA binding protein 1, isoform | CELF1 | 3 | 55.1 | 11 | -3.92 | -3.28 | 0.64 |
| 161 | Q8NI22 | Multiple coagulation factor deficiency protein 2 | MCFD2 | 3 | 16.4 | 57 | -3.91 | -3.38 | 1.08 |
| 162 | Q567U8 | COPS7A protein | COPS7A | 2 | 27.3 | 15 | -3.92 | -4.57 | -0.69 |
| 163 | A0A087X2I1 | 26S proteasome regulatory subunit 10B | PSMC6 | 5 | 45.8 | 20 | -3.93 | -3.42 | 0.51 |
| 164 | A6NMQ1 | DNA polymerase | POLA1 | 3 | 166.4 | 4 | -3.92 | -2.9 | 1.03 |
| 165 | Q567R6 | Single-stranded DNA-binding protein, mitochondrial | SSBP1 | 2 | 17.3 | 22 | -3.91 | -3.85 | 0.07 |
| 166 | P28066 | Proteasome subunit alpha type-5 | PSMA5 | 4 | 26.4 | 31 | -3.93 | -2.49 | 1.44 |
| 167 | Q8WUH6 | Transmembrane protein 263 | TMEM263 | 2 | 11.7 | 36 | -3.89 | -2.05 | 1.53 |
| 168 | Q53FW7 | GA binding protein transcription factor, alpha subunit (60kD) variant | GABP1 | 4 | 51.2 | 25 | -3.9 | -2.85 | 1.05 |
| 169 | O00425 | Insulin-like growth factor 2 mRNA-binding protein 3 | IGF2BP3 | 4 | 63.7 | 12 | -3.89 | -3.86 | 0.04 |
| 170 | B3KRG2 | DNA excision repair protein ERCC-3 | ERCC3 | 2 | 82 | 6 | -3.9 | -3.18 | 0.72 |
| 171 | A0A0A0MS29 | Mitochondrial fission factor | MFF | 5 | 25.3 | 41 | -3.89 | -3.28 | 0.61 |
| 172 | O95400 | CD2 antigen cytoplasmic tail-binding protein 2 | CD2BP2 | 5 | 37.6 | 35 | -3.91 | -3.94 | -0.03 |
| 173 | Q92905 | COP9 signalosome complex subunit 5 | COPS5 | 5 | 37.6 | 31 | -3.89 | -4.05 | -0.16 |
| 174 | B7ZLJ8 | SEZ6L protein | SEZ6L | 3 | 110.3 | 9 | -3.88 | -2.18 | 1.7 |
| 175 | A8KAP9 | Argininosuccinate synthase | ASS | 4 | 46.5 | 15 | -3.86 | -2.66 | 1.2 |
| 176 | K7EJD3 | Galectin-3-binding protein | LGALS3BP | 2 | 22 | 24 | -3.85 | -3.61 | -0.29 |
| 177 | A0A0S2Z4G7 | Nucleophosmin | NPM1 | 7 | 29.4 | 42 | -3.85 | -3.47 | 0.38 |
| 178 | P05387 | 60S acidic ribosomal protein P2 | RPLP2 | 6 | 11.7 | 86 | -3.85 | -4.13 | -0.28 |
| 179 | Q9UHX1 | Poly(U)-binding-splicing factor PUF60 | PUF60 | 3 | 59.8 | 22 | -3.83 | -3.41 | 0.42 |
| 180 | Q8WU90 | Zinc finger CCCH domain-containing protein 15 | ZC3H15 | 4 | 48.6 | 20 | -3.83 | -3.11 | 0.71 |
| 181 | D3DR67 | CWF19-like 1, cell cycle control (S. pombe), isoform | CWF19L1 | 3 | 33.5 | 22 | -3.83 | -3.53 | 0.3 |
| 182 | Q53GD7 | FUS interacting protein (Serine-arginine rich) 1 isoform 2 variant | SRSF10 | 2 | 31.3 | 10 | -3.83 | -2.83 | 1 |
| 183 | Q0VGD6 | HNRPR protein | HNRPR | 2 | 67.8 | 29 | -3.84 | -4.15 | -0.31 |
| 184 | H0YLF5 | Pre-B-cell leukemia transcription factor 1 | PBX1 | 3 | 37 | 19 | -3.85 | -3.15 | 0.69 |
| 185 | K7ERF1 | Eukaryotic translation initiation factor 3 subunit K | EIF3K | 3 | 22.1 | 27 | -3.81 | -3.71 | 0.1 |
| 186 | F8VQE1 | LIM domain and actin-binding protein 1 | LIMA1 | 3 | 67 | 12 | -3.8 | -2.98 | 0.82 |
| 187 | I3L1Q5 | Pre-rRNA-processing protein TSR1 homolog | TSR1 | 4 | 41.4 | 20 | -3.8 | -4.19 | -0.39 |
| 188 | Q96AG4 | Leucine-rich repeat-containing protein 59 | LRRC59 | 8 | 34.9 | 27 | -3.79 | -3.49 | 0.3 |
| 189 | B4DYN5 | Succinate dehydrogenase [ubiquinone] flavoprotein subunit, mitochondrial | SDH | 8 | 72.6 | 28 | -3.78 | -3.06 | 0.72 |
| 190 | B1AJY5 | 26S proteasome non-ATPase regulatory subunit 10 | PSMD10 | 2 | 20.2 | 17 | -3.78 | -3.09 | 0.69 |
| 191 | R9WQY1 | Synaptic functional regulator FMR1 | FMR1 | 4 | 63.9 | 12 | -3.76 | -3.78 | -0.02 |
| 192 | A0A6Q8PH91 | ADP-ribose glycohydrolase MACROD1 | MACROD1 | 2 | 36.4 | 12 | -3.77 | -3.94 | -0.18 |
| 193 | Q59EI9 | ADP,ATP carrier protein, liver isoform T2 variant | AAC | 2 | 35.4 | 16 | -3.77 | -3.94 | -0.17 |
| 194 | A0A3F2YNX8 | Protein-L-isoaspartate O-methyltransferase | PCMT1 | 2 | 26.3 | 20 | -3.75 | -4.33 | -1.15 |
| 195 | Q9BVJ6 | U3 small nucleolar RNA-associated protein 14 homolog A | UTP14A | 4 | 87.9 | 14 | -3.75 | -3.56 | 0.19 |
| 196 | B4DZC9 | Non-specific serine/threonine protein kinase | FRAP1 | 3 | 119.8 | 5 | -3.74 | -3.62 | 0.12 |
| 197 | H0YIQ2 | YLP motif-containing protein 1 | YLPM1 | 5 | 180.2 | 7 | -3.74 | -2.94 | 0.8 |
| 198 | A3KFL2 | Ribosomal RNA-processing protein 4 | EXOSC2 | 4 | 32 | 20 | -3.71 | -4.01 | -0.29 |
| 199 | B2RDE0 | Propanoyl-CoA:carbon dioxide ligase subunit alpha | PCCA | 3 | 77.4 | 8 | -3.7 | -3.8 | -0.11 |
| 200 | D6RIC3 | Nucleolar protein 16 | NOP16 | 2 | 17.2 | 14 | -3.69 | -3.45 | 0.24 |
| 201 | F8VTY2 | Nucleoporin Nup37 | NUP37 | 2 | 23.3 | 16 | -3.69 | -3.7 | -0.01 |
| 202 | Q8WXX5 | DnaJ homolog subfamily C member 9 | DNAJC9 | 2 | 29.9 | 15 | -3.68 | -3.81 | -0.13 |
| 203 | C9J9T0 | Ribulose-phosphate 3-epimerase | RPE | 3 | 21 | 29 | -3.69 | -4.18 | -0.49 |
| 204 | E9PFL3 | Dehydrogenase/reductase SDR family member 4-like 2 | DHRS4L2 | 4 | 20.9 | 42 | -3.68 | -2.97 | 0.71 |
| 205 | O75396 | Vesicle-trafficking protein SEC22b | SEC22B | 3 | 24.7 | 19 | -3.68 | -3.99 | -0.31 |
| 206 | Q86W50 | RNA N6-adenosine-methyltransferase METTL16 | METTL16 | 2 | 63.6 | 9 | -3.67 | -3.68 | -0.01 |
| 207 | P62760 | Visinin-like protein 1 | VSNL1 | 4 | 22.1 | 28 | -3.69 | -3.01 | 0.68 |
| 208 | Q8WWQ0 | PH-interacting protein | PHIP | 2 | 206.6 | 2 | -3.66 | -3.16 | 0.5 |
| 209 | M0R1B0 | ER membrane protein complex subunit 8 | EMC8 | 2 | 11.7 | 33 | -3.65 | -3.9 | -0.25 |
| 210 | G3V340 | Striatin-3 | STRN3 | 3 | 36.2 | 17 | -3.67 | -3.13 | 0.54 |
| 211 | D3DUG9 | Ubiquitin carboxyl-terminal hydrolase | USP14 | 6 | 53.1 | 31 | -3.66 | -1.93 | 1.74 |
| 212 | P52788 | Spermine synthase | SMS | 12 | 41.2 | 60 | -3.64 | -3.37 | 0.27 |
| 213 | P56589 | Peroxisomal biogenesis factor 3 | PEX3 | 2 | 42.1 | 13 | -3.65 | -3.68 | -0.03 |
| 214 | Q07021 | Complement component 1 Q subcomponent-binding protein, mitochondrial | C1QBP | 8 | 31.3 | 41 | -3.64 | -3.08 | 1.36 |
| 215 | H3BNC9 | 40S ribosomal protein S17 | RPS17 | 7 | 64.5 | 11 | -3.64 | -2.98 | 0.86 |
| 216 | D6W5Y5 | Cold-inducible RNA-binding protein | CIRBP | 3 | 31.9 | 15 | -3.65 | -3.02 | 0.64 |
| 217 | P46013 | Proliferation marker protein Ki-67 | MKI67 | 5 | 358.5 | 4 | -3.65 | -3.77 | -0.12 |
| 218 | Q8TF42 | Ubiquitin-associated and SH3 domain-containing protein B | UBASH3B | 5 | 72.6 | 17 | -3.64 | -3.14 | 0.5 |
| 219 | E9PPJ0 | Splicing factor 3B subunit 2 | SF3B2 | 6 | 98.1 | 15 | -3.64 | -2.97 | 0.66 |
| 220 | B7Z9M9 | Actin-depolymerizing factor | ADF | 4 | 16.5 | 34 | -3.63 | -3.45 | 0.18 |
| 221 | B4E1E2 | Hepatocyte growth factor-regulated tyrosine kinase substrate | HGS | 2 | 74.8 | 4 | -3.63 | -4.25 | -0.46 |
| 222 | A0A1B0GU92 | Peptidase A1 domain-containing protein | PA1 | 5 | 59.8 | 16 | -3.63 | -3.91 | -0.32 |
| 223 | B2RDD7 | Protein arginine N-methyltransferase 5 | PRMT5 | 5 | 72.7 | 15 | -3.63 | -2.78 | 0.85 |
| 224 | Q3B7X1 | TMX3 protein | TMX3 | 3 | 48.5 | 13 | -3.63 | -2.81 | 0.82 |
| 225 | O94888 | UBX domain-containing protein 7 | UBXN7 | 3 | 54.8 | 13 | -3.63 | -3.25 | 0.38 |
| 226 | Q92896 | Golgi apparatus protein 1 | GLG1 | 9 | 134.5 | 16 | -3.62 | -3.2 | 0.42 |
| 227 | Q86UP2 | Kinectin | KTN1 | 15 | 156.2 | 19 | -3.63 | -3.4 | 0.23 |
| 228 | A0A248RGE3 | 40S ribosomal protein S27a | RPS27A | 4 | 16.7 | 32 | -3.61 | -5.02 | -1.43 |
| 229 | B3KQF5 | Calumenin | CALU | 7 | 37 | 36 | -3.61 | -3.85 | -0.24 |
| 230 | E9PGT6 | COP9 signalosome complex subunit 8 | COPS8 | 3 | 19.3 | 46 | -3.61 | -3.58 | 0.03 |
| 231 | A0A087WTW0 | RING-type E3 ubiquitin transferase | UHRF1 | 4 | 96.6 | 11 | -3.6 | -3.64 | -0.04 |
| 232 | B4DHR2 | Nucleolar GTP-binding protein 1 | NGP-1 | 3 | 60 | 10 | -3.61 | -1.99 | 1.62 |
| 233 | S4R3H4 | Apoptotic chromatin condensation inducer in the nucleus | ACIN1 | 7 | 145.4 | 10 | -3.59 | -4.04 | -0.45 |
| 234 | B4DJ81 | NADH-ubiquinone oxidoreductase 75 kDa subunit, mitochondrial | NDUFS1 | 7 | 66.9 | 22 | -3.6 | -2.63 | 0.96 |
| 235 | Q9UIU0 | Dihydropyridine receptor alpha 2 subunit | CACNA2D1 | 4 | 125.2 | 5 | -3.6 | -3.49 | 0.1 |
| 236 | H0YJ66 | Dehydrogenase/reductase SDR family member 7 | DHRS7 | 3 | 44.8 | 12 | -3.59 | -3.62 | -0.03 |
| 237 | P52655 | Transcription initiation factor IIA subunit 1 | GTF2A1 | 2 | 41.5 | 16 | -3.58 | -1.93 | 1.66 |
| 238 | Q9Y5K6 | CD2-associated protein | CD2AP | 3 | 71.4 | 10 | -3.59 | -5.64 | -2.05 |
| 239 | J3KN67 | Tropomyosin alpha-3 chain | TPM3 | 2 | 33.2 | 11 | -3.59 | -3.96 | -0.37 |
| 240 | P28074 | Proteasome subunit beta type-5 | PSMB5 | 5 | 28.5 | 33 | -3.59 | -3.2 | 0.39 |
| 241 | P49773 | Adenosine 5'-monophosphoramidase HINT1 | HINT1 | 4 | 13.8 | 40 | -3.57 | -3.27 | 0.3 |
| 242 | F4ZW64 | NF90a | None | 13 | 75.9 | 26 | -3.58 | -3.25 | 0.33 |
| 243 | Q16698 | 2,4-dienoyl-CoA reductase [(3E)-enoyl-CoA-producing], mitochondrial | DECR1 | 4 | 36 | 14 | -3.57 | -3.02 | 0.55 |
| 244 | B3KPA6 | Acyl-Coenzyme A dehydrogenase, very long chain, isoform | ACADVL | 6 | 62.5 | 33 | -3.55 | -2.72 | 0.83 |
| 245 | Q59GY2 | 60S ribosomal protein L4 | RPL4 | 6 | 49 | 18 | -3.56 | -3.27 | 0.28 |
| 246 | A8K0C2 | ESCRT-I complex subunit MVB12A | MVB12A | 3 | 28.8 | 18 | -3.54 | -4.05 | -0.5 |
| 247 | B4DKZ6 | THO complex subunit 2 | THOC2 | 3 | 97.1 | 9 | -3.53 | -2.98 | 0.56 |
| 248 | J3KSG2 | Cytospin-B | SPECC1 | 2 | 60.1 | 5 | -3.54 | -2.73 | 0.81 |
| 249 | F8VY35 | Nucleosome assembly protein 1-like 1 | NAP1L1 | 11 | 31 | 61 | -3.54 | -3.15 | 0.4 |
| 250 | Q9NX55 | Huntingtin-interacting protein K | HYPK | 3 | 13.6 | 45 | -3.54 | -3.54 | -0.01 |
| 251 | Q53HF1 | Heme oxygenase (biliverdin-producing) | HO | 4 | 35.9 | 21 | -3.54 | -3.11 | 0.43 |
| 252 | P30038 | Delta-1-pyrroline-5-carboxylate dehydrogenase, mitochondrial | ALDH4A1 | 6 | 61.7 | 24 | -3.52 | -3.26 | 0.26 |
| 253 | F8WAN9 | GMP reductase | GMPR2 | 2 | 38.2 | 15 | -3.53 | -3.36 | 0.17 |
| 254 | Q53HU7 | Eukaryotic translation initiation factor 3, subunit 2 beta, 36kDa variant | EIF3 | 4 | 36.5 | 33 | -3.51 | -4.3 | -0.8 |
| 255 | P11182 | Lipoamide acyltransferase component of branched-chain alpha-keto acid dehydrogenase complex, mitochondrial [ | DBT | 5 | 53.5 | 27 | -3.51 | -2.94 | 0.66 |
| 256 | Q6FGX3 | RAB6A protein | RAB6A | 4 | 23.5 | 27 | -3.51 | -3.43 | 0.08 |
| 257 | C9JLV6 | Cytosolic malate dehydrogenase | MDH1 | 3 | 12.4 | 35 | -3.5 | -3.99 | -0.5 |
| 258 | Q14566 | DNA replication licensing factor MCM6 | MCM6 | 5 | 92.8 | 11 | -3.49 | -3.47 | 0.02 |
| 259 | Q92626 | Peroxidasin homolog | PXDN | 4 | 165.2 | 7 | -3.5 | -3.19 | 0.31 |
| 260 | B4DJX1 | Acetyltransferase component of pyruvate dehydrogenase complex | PDC | 10 | 62.7 | 28 | -3.5 | -2.77 | 0.73 |
| 261 | Q53FB6 | Mitochondrial aldehyde dehydrogenase 2 variant | ALDH2 | 5 | 56.3 | 19 | -3.48 | -3.77 | -0.29 |
| 262 | Q16891 | MICOS complex subunit MIC60 | IMMT | 7 | 83.6 | 20 | -3.48 | -3.93 | -0.45 |
| 263 | Q9Y5S9 | RNA-binding protein 8A | RBM8A | 3 | 19.9 | 32 | -3.47 | -3.55 | -0.07 |
| 264 | B2RB32 | Alpha-1,4 glucan phosphorylase | α-GPs | 2 | 97 | 5 | -3.46 | -2.34 | 1.12 |
| 265 | P46109 | Crk-like protein | CRKL | 2 | 33.8 | 10 | -3.45 | -2.88 | 0.58 |
| 266 | O43491 | Band 4.1-like protein 2 | EPB41L2 | 9 | 112.5 | 17 | -3.46 | -2.47 | 0.99 |
| 267 | P08559 | Pyruvate dehydrogenase E1 component subunit alpha, somatic form, mitochondrial | PDHA1 | 6 | 43.3 | 29 | -3.45 | -2.99 | 0.46 |
| 268 | A0A6Q8PFJ0 | Prelamin-A/C | LMNA | 5 | 80.9 | 11 | -3.45 | -2.47 | 0.99 |
| 269 | C9J634 | Pyruvate dehydrogenase E1 component subunit beta | PDHB | 4 | 38.4 | 20 | -3.44 | -4.04 | -0.6 |
| 270 | C9J9K3 | 40S ribosomal protein SA | RPSA | 6 | 29.4 | 39 | -3.44 | -2.93 | 0.51 |
| 271 | A0A0S2Z462 | ArfGAP with FG repeats 1 isoform 2 | AGFG1 | 2 | 62.6 | 7 | -3.44 | -3.09 | 0.35 |
| 272 | C9JME2 | FERM, ARHGEF and pleckstrin domain-containing protein 1 | FARP1 | 2 | 122 | 3 | -3.44 | -3.42 | 0.03 |
| 273 | Q32Q12 | Nucleoside diphosphate kinase | NME1-NME2 | 5 | 32.6 | 36 | -3.45 | -3.6 | -0.15 |
| 274 | P49792 | E3 SUMO-protein ligase RanBP2 | RANBP2 | 17 | 358 | 11 | -3.44 | -3.25 | 0.2 |
| 275 | P51148 | Ras-related protein Rab-5C | RAB5C | 5 | 23.5 | 33 | -3.44 | -3.34 | 0.1 |
| 276 | P80303 | Nucleobindin-2 | NUCB2 | 3 | 50.2 | 13 | -3.44 | -3.29 | 0.15 |
| 277 | H0YEW4 | ATP synthase mitochondrial F1 complex assembly factor 1 | ATPAF1 | 2 | 19.9 | 17 | -3.42 | -3.33 | 0.09 |
| 278 | P62857 | 40S ribosomal protein S28 | RPS28 | 2 | 7.8 | 23 | -3.43 | -1.57 | 1.85 |
| 279 | F8VU90 | Peptidylprolyl isomerase | FKBP11 | 2 | 19.8 | 30 | -3.43 | -3.92 | -0.48 |
| 280 | Q6I9U9 | Asparaginyl endopeptidase | LGMN | 2 | 49.3 | 10 | -3.43 | -2.84 | 0.59 |
| 281 | A0A1B0GVW0 | ATPase H(+)-transporting lysosomal accessory protein 2 | ATP6AP2 | 4 | 38.4 | 19 | -3.4 | -2.94 | 0.47 |
| 282 | F8WAE5 | Eukaryotic translation initiation factor 2A | EIF2A | 5 | 64.4 | 20 | -3.41 | -3.31 | 0.1 |
| 283 | Q8TB01 | Similar to cytoskeleton-associated protein 4 | CKAP4 | 8 | 62 | 19 | -3.42 | -3.67 | -0.25 |
| 284 | F8W6I7 | Helix-destabilizing protein | HNRNPA1 | 8 | 33.1 | 29 | -3.42 | -2.49 | 0.93 |
| 285 | B8ZZX6 | Metalloreductase STEAP3 | STEAP3 | 2 | 50.6 | 10 | -3.41 | -2.87 | 0.99 |
| 286 | O75131 | Copine-3 | CPNE3 | 4 | 60.1 | 14 | -3.41 | -3.7 | -0.29 |
| 287 | P10155 | RNA-binding protein RO60 | RO60 | 5 | 60.6 | 19 | -3.41 | -4.45 | -1.04 |
| 288 | P50570 | Dynamin-2 | DNM2 | 2 | 98 | 11 | -3.41 | -2.87 | 0.54 |
| 289 | B2R5M9 | Procollagen-lysine 5-dioxygenase | PLOD | 6 | 83.5 | 12 | -3.41 | -2.88 | 0.53 |
| 290 | D6RFN0 | COP9 signalosome complex subunit 4 | COPS4 | 7 | 49.7 | 26 | -3.4 | -3.2 | 0.2 |
| 291 | O75976 | Carboxypeptidase D | CPD | 5 | 152.8 | 9 | -3.39 | -3.46 | -0.07 |
| 292 | O00231 | 26S proteasome non-ATPase regulatory subunit 11 | PSMD11 | 7 | 47.4 | 24 | -3.4 | -2.86 | 0.54 |
| 293 | Q9BXP5 | Serrate RNA effector molecule homolog | SRRT | 6 | 100.6 | 13 | -3.39 | -2.45 | 0.94 |
| 294 | A8K7Q1 | Nucleobindin-1 | NUCB1 | 5 | 53.9 | 14 | -3.38 | -3.41 | -0.03 |
| 295 | X6RAC9 | Eukaryotic translation initiation factor 4C | EIF1AX | 2 | 13.2 | 38 | -3.38 | -4.13 | -0.75 |
| 296 | Q9UBS4 | DnaJ homolog subfamily B member 11 | DNAJB11 | 2 | 40.5 | 14 | -3.37 | -2.51 | 0.87 |
| 297 | A0A2R8Y5Q8 | Tubulin-folding cofactor E | TBCE | 4 | 61.8 | 14 | -3.38 | -3.15 | 0.23 |
| 298 | B4DFG0 | Protein DEK | DEK | 2 | 39.5 | 7 | -3.38 | -3.13 | 0.26 |
| 299 | Q5T1M5 | FK506-binding protein 15 | FKBP15 | 3 | 133.5 | 4 | -3.36 | -4.23 | -0.86 |
| 300 | E9PDQ8 | Succinate--CoA ligase [GDP-forming] subunit beta, mitochondrial | SUCLG2 | 2 | 41.4 | 12 | -3.37 | -2.77 | 0.6 |
| 301 | P05386 | 60S acidic ribosomal protein P1 | RPLP1 | 3 | 11.5 | 91 | -3.37 | -1.12 | 2.25 |
| 302 | K7EN45 | Peptidyl-prolyl cis-trans isomerase | PIN1 | 2 | 9.9 | 36 | -3.36 | -2.66 | 0.7 |
| 303 | H0YIY8 | Serine/threonine-protein phosphatase 4 regulatory subunit 3A | PPP4R3A | 4 | 62.2 | 10 | -3.34 | -2.63 | 0.72 |
| 304 | B4DKX4 | Programmed cell death protein 4 | PDCD4 | 7 | 50.2 | 30 | -3.35 | -2.9 | 0.46 |
| 305 | B4DY08 | Heterogeneous nuclear ribonucleoproteins C1/C2 | HNRNPC | 2 | 32 | 24 | -3.34 | -2.17 | 1.17 |
| 306 | P52597 | Heterogeneous nuclear ribonucleoprotein F | HNRNPF | 6 | 45.6 | 39 | -3.33 | -2.76 | 0.57 |
| 307 | Q7Z3K3 | Pogo transposable element with ZNF domain | POGZ | 4 | 155.2 | 7 | -3.34 | -2.79 | 0.55 |
| 308 | Q99798 | Aconitate hydratase, mitochondrial | ACO2 | 3 | 85.4 | 29 | -3.33 | -4.42 | -1.08 |
| 309 | I3L2R6 | Thioredoxin domain-containing protein 17 | TXNDC17 | 2 | 11.3 | 34 | -3.34 | -2.66 | 0.68 |
| 310 | Q8NE02 | AKAP8 protein | AKAP8 | 2 | 76.1 | 3 | -3.33 | -2.76 | 0.57 |
| 311 | C9JZR2 | Catenin delta-1 | CTNND1 | 6 | 104.8 | 11 | -3.34 | -2.11 | 1.23 |
| 312 | A0A590UKA8 | Disks large homolog 1 | DLG1 | 5 | 98.3 | 14 | -3.33 | -3.83 | -0.49 |
| 313 | J3QT28 | Mitotic checkpoint protein BUB3 | BUB3 | 3 | 31.7 | 28 | -3.34 | -3.42 | -0.08 |
| 314 | A0A0A6YYA0 | Protein TMED7-TICAM2 | TMED7-TICAM2 | 2 | 21.2 | 21 | -3.33 | -2.9 | 0.43 |
| 315 | Q13308 | Inactive tyrosine-protein kinase 7 | PTK7 | 8 | 118.3 | 16 | -3.33 | -3.1 | 0.22 |
| 316 | Q9BRX8 | Peroxiredoxin-like 2A | PRXL2A | 2 | 25.7 | 12 | -3.33 | -2.09 | 1.23 |
| 317 | Q8N766 | ER membrane protein complex subunit 1 | EMC1 | 8 | 111.7 | 15 | -3.32 | -3.2 | 0.12 |
| 318 | Q9H444 | Charged multivesicular body protein 4b | CHMP4B | 2 | 24.9 | 19 | -3.32 | -3.92 | -0.59 |
| 319 | O75367 | Core histone macro-H2A.1 | MACROH2A1 | 5 | 39.2 | 23 | -3.31 | -3 | 0.3 |
| 320 | Q9NQS1 | Cell death regulator Aven | AVEN | 4 | 38.5 | 24 | -3.31 | -3.63 | -0.31 |
| 321 | B3KMI0 | Cleavage and polyadenylation specificity factor subunit 2 | CPSF2 | 4 | 73 | 9 | -3.29 | -3.75 | -0.46 |
| 322 | A0A024R2K4 | Leucine rich repeat (In FLII) interacting protein 2, isoform | LRRFIP2 | 4 | 84.1 | 14 | -3.29 | -2.84 | 0.45 |
| 323 | A0A0B4J210 | La-related protein 1 | LARP1 | 5 | 90.8 | 11 | -3.29 | -0.87 | 2.42 |
| 324 | A0A0G2JQ62 | Lysophosphatidylcholine acyltransferase 1 | LPCAT1 | 2 | 54.4 | 10 | -3.29 | -4.32 | -1.05 |
| 325 | P05556 | Integrin beta-1 | ITGB1 | 5 | 88.4 | 13 | -3.29 | -3.04 | 0.26 |
| 326 | P78318 | Immunoglobulin-binding protein 1 | IGBP1 | 2 | 39.2 | 8 | -3.29 | -2.36 | 0.93 |
| 327 | Q6WKZ4 | Rab11 family-interacting protein 1 | RAB11FIP1 | 5 | 137.1 | 9 | -3.28 | -2.92 | 0.36 |
| 328 | B4DN41 | DEAD box protein 5 | Dbp5 | 5 | 67.7 | 27 | -3.28 | -2.81 | 0.47 |
| 329 | Q6FI37 | Isocitrate dehydrogenase [NADP] | IDH1 | 6 | 46.6 | 27 | -3.28 | -2.72 | 0.57 |
| 330 | P13804 | Electron transfer flavoprotein subunit alpha, mitochondrial | ETFA | 7 | 35.1 | 34 | -3.29 | -3.19 | 0.09 |
| 331 | A0A087X1G7 | Selenoprotein F | SELENOF | 2 | 17.9 | 27 | -3.27 | -2.98 | 0.29 |
| 332 | Q4VC31 | Protein MIX23 | MIX23 | 2 | 16.6 | 25 | -3.27 | -3.08 | 0.19 |
| 333 | Q9UKD1 | Glucocorticoid modulatory element-binding protein 2 | GMEB2 | 3 | 56.4 | 18 | -3.27 | -3.68 | -0.41 |
| 334 | A0A024QZW7 | Nucleoporin 153kDa, isoform | NUP153 | 4 | 153.9 | 7 | -3.25 | -3.66 | -0.41 |
| 335 | B4E0K9 | Alpha-mannosidase | MAN2B1 | 3 | 107.4 | 8 | -3.24 | -2.49 | 0.75 |
| 336 | V9HW39 | Epididymis secretory protein Li 100 | HEL-S-100 | 5 | 40.3 | 35 | -3.24 | -2.84 | 0.4 |
| 337 | Q8N1C8 | 75 kDa glucose-regulated protein | HSPA9 | 15 | 73.8 | 29 | -3.24 | -2.85 | 0.39 |
| 338 | A0A1B0GW23 | N-acyl-L-amino-acid amidohydrolase | ABHD14A-ACY1 | 5 | 65.4 | 21 | -3.24 | -2.91 | 0.32 |
| 339 | Q6PD74 | Alpha- and gamma-adaptin-binding protein p34 | AAGAB | 3 | 34.6 | 32 | -3.23 | -3.26 | -0.03 |
| 340 | Q9UQ35 | Serine/arginine repetitive matrix protein 2 | SRRM2 | 4 | 299.4 | 3 | -3.24 | -4.21 | -0.97 |
| 341 | Q9Y512 | Sorting and assembly machinery component 50 homolog | SAMM50 | 4 | 51.9 | 15 | -3.24 | -3.24 | -0.01 |
| 342 | P20674 | Cytochrome c oxidase subunit 5A, mitochondrial | COX5A | 5 | 16.8 | 33 | -3.24 | -3.4 | -0.16 |
| 343 | Q13151 | Heterogeneous nuclear ribonucleoprotein A0 | HNRNPA0 | 4 | 30.8 | 29 | -3.22 | -4.81 | -1.59 |
| 344 | P08133 | Annexin A6 | ANXA6 | 8 | 75.8 | 21 | -3.23 | -2.55 | 0.67 |
| 345 | P68402 | Platelet-activating factor acetylhydrolase IB subunit alpha2 | PAFAH1B2 | 3 | 25.6 | 34 | -3.21 | -3.66 | -0.45 |
| 346 | A0A024R394 | Cysteine and histidine-rich domain (CHORD)-containing 1, isoform | CHORDC1 | 2 | 37.5 | 12 | -3.21 | -2.89 | 0.32 |
| 347 | Q53GB2 | GATOR complex protein SEC13 | GATOR | 2 | 35.5 | 14 | -3.21 | -4.43 | -1.22 |
| 348 | B4DHN0 | Mitogen-activated protein kinase | MAPK | 4 | 40.4 | 22 | -3.21 | -4.67 | -1.46 |
| 349 | F8W930 | Insulin-like growth factor 2 mRNA-binding protein 2 | IGF2BP2 | 3 | 66.7 | 9 | -3.22 | -2.91 | 0.31 |
| 350 | Q9Y5J9 | Mitochondrial import inner membrane translocase subunit Tim8 B | TIMM8B | 2 | 9.3 | 30 | -3.21 | -1.98 | 1.23 |
| 351 | B2R7D2 | 2,3-bisphosphoglycerate 3-phosphatase | 2,3-BPG | 3 | 55.1 | 11 | -3.2 | -4.47 | -1.4 |
| 352 | Q6LET3 | Hypoxanthine phosphoribosyltransferase | HPRT1 | 2 | 24.6 | 20 | -3.2 | -4.4 | -1.2 |
| 353 | D6RA00 | Enolase-phosphatase E1 | ENOPH1 | 2 | 19.2 | 28 | -3.2 | -2.33 | 0.87 |
| 354 | A0A6Q8PHI5 | Elongator complex protein 1 | ELP1 | 3 | 133.5 | 7 | -3.18 | -3.04 | 0.14 |
| 355 | Q6DKK2 | Tetratricopeptide repeat protein 19, mitochondrial | TTC19 | 2 | 42.4 | 9 | -3.19 | -1.1 | 2.09 |
| 356 | Q6FHZ0 | Malate dehydrogenase | MDH2 | 7 | 35.5 | 33 | -3.18 | -3.1 | 0.08 |
| 357 | A6NFX8 | ADP-sugar pyrophosphatase | NUDT5 | 4 | 25.9 | 30 | -3.18 | -3.1 | 0.08 |
| 358 | P29317 | Ephrin type-A receptor 2 | EPHA2 | 6 | 108.2 | 14 | -3.19 | -1.93 | 1.26 |
| 359 | P30050 | 60S ribosomal protein L12 | RPL12 | 2 | 17.8 | 19 | -3.18 | -2.97 | 0.21 |
| 360 | Q92576 | PHD finger protein 3 | PHF3 | 3 | 229.3 | 3 | -3.18 | -2.54 | 0.64 |
| 361 | A0A087WVM4 | Formyltetrahydrofolate synthetase | MTHFD1L | 10 | 99.2 | 27 | -3.17 | -2.86 | 0.31 |
| 362 | O43707 | Alpha-actinin-4 | ACTN4 | 13 | 104.8 | 31 | -3.17 | -3.74 | -0.57 |
| 363 | M0R2N5 | Very-long-chain enoyl-CoA reductase | TECR | 2 | 39.9 | 10 | -3.17 | -3.47 | -0.3 |
| 364 | P24534 | Elongation factor 1-beta | EEF1B2 | 7 | 24.7 | 72 | -3.16 | -2.85 | 0.31 |
| 365 | Q5JRA6 | Transport and Golgi organization protein 1 homolog | MIA3 | 5 | 213.6 | 7 | -3.16 | -3.73 | -0.57 |
| 366 | B4DWV5 | GrpE protein homolog | GrpE | 2 | 21.9 | 13 | -3.15 | -3.97 | -0.82 |
| 367 | B2RNR6 | Zinc finger RNA binding protein | ZFR | 5 | 116.9 | 9 | -3.14 | -3.6 | -0.46 |
| 368 | J3KRX5 | 60S ribosomal protein L17 | RPL17 | 3 | 20.2 | 21 | -3.15 | -3.51 | -0.36 |
| 369 | A0A024R875 | GTPase activating protein and VPS9 domains 1, isoform | GAPVD1 | 2 | 159.7 | 2 | -3.15 | -1.58 | 1.56 |
| 370 | D3DSQ1 | N-acylsphingosine amidohydrolase (Acid ceramidase) 1, isoform | ASAH1 | 5 | 60.6 | 15 | -3.15 | -2.97 | 0.18 |
| 371 | Q14966 | Zinc finger protein 638 | ZNF638 | 9 | 220.5 | 13 | -3.14 | -1.08 | 2.22 |
| 372 | A0A0S2Z404 | Regulator of chromosome condensation 1 isoform 2 | RCC1 | 11 | 48.1 | 47 | -3.14 | -2.63 | 0.52 |
| 373 | A0A087X0K9 | Tight junction protein ZO-1 | TJP1 | 3 | 187.7 | 5 | -3.14 | -3.62 | -0.48 |
| 374 | G5E9D5 | ElaC homolog protein 2 | ELAC2 | 8 | 90 | 22 | -3.13 | -2.26 | 0.87 |
| 375 | Q12789 | General transcription factor 3C polypeptide 1 | GTF3C1 | 7 | 238.7 | 8 | -3.13 | -2.46 | 0.67 |
| 376 | H0YGS7 | Tripartite motif-containing protein 65 | TRIM65 | 2 | 27.1 | 14 | -3.13 | -1.53 | 1.59 |
| 377 | Q59GS3 | Proteasome 26S ATPase subunit 5 variant | PSMC5 | 10 | 38.7 | 39 | -3.12 | -3.04 | 0.08 |
| 378 | H3BPB8 | Mannose-6-phosphate isomerase | MPI | 4 | 44.4 | 18 | -3.12 | -1.2 | 1.92 |
| 379 | A0A024R321 | Filamin B, beta (Actin binding protein 278), isoform | FLNB | 29 | 280.3 | 26 | -3.11 | -2.93 | 0.18 |
| 380 | Q8N5L9 | 40S ribosomal protein S2 | RPS2 | 3 | 31.3 | 15 | -3.11 | -1.53 | 1.58 |
| 381 | J3KP30 | Deoxynucleotidyl transferase terminal-interacting protein 2 | DNTTIP2 | 3 | 66.1 | 10 | -3.11 | -3.62 | -0.51 |
| 382 | H3BUW8 | Lon protease homolog 2, peroxisomal | LONP2 | 2 | 33.9 | 14 | -3.11 | -3.07 | 0.03 |
| 383 | Q7RU05 | Mitochondrial import inner membrane translocase subunit TIM17 | TIM17A | 2 | 17.9 | 25 | -3.1 | -2.26 | 0.84 |
| 384 | J3K000 | PEPD protein | PEPD | 4 | 54.5 | 16 | -3.1 | -3.93 | -0.83 |
| 385 | A0A1B0GWA2 | Alkylglycerone-phosphate synthase | AGPS | 4 | 60.9 | 17 | -3.09 | -2.57 | 0.53 |
| 386 | A0A4W8VX11 | Pericentriolar material 1 protein | PCM1 | 2 | 210 | 4 | -3.1 | -2.94 | 0.15 |
| 387 | B4DFL1 | Dihydrolipoyl dehydrogenase | DLD | 6 | 48.9 | 19 | -3.1 | -3.72 | -0.62 |
| 388 | E9PC15 | Acylglycerol kinase, mitochondrial | AGK | 4 | 43.8 | 16 | -3.09 | -1.93 | 1.16 |
| 389 | Q08AJ6 | ANP32A protein | ANP32A | 5 | 27.3 | 29 | -3.09 | -3.68 | -0.6 |
| 390 | P60174 | Triosephosphate isomerase | TPI1 | 8 | 30.8 | 45 | -3.08 | -3.43 | -0.35 |
| 391 | Q96CE4 | Stathmin | STMN1 | 2 | 17.3 | 9 | -3.07 | -2.37 | 0.7 |
| 392 | B1AK13 | 3-hydroxy-3-methylglutarate-CoA lyase | HMGCL | 2 | 31.7 | 12 | -3.07 | -2.77 | 1.01 |
| 393 | Q9BYK2 | 40S ribosomal protein S21 | RPS21 | 2 | 7.1 | 24 | -3.07 | -3.06 | 0.02 |
| 394 | A0A2R8Y602 | Caseinolytic peptidase B protein homolog | CLPB | 2 | 43.3 | 11 | -3.07 | -3.29 | -0.22 |
| 395 | P04406 | Glyceraldehyde-3-phosphate dehydrogenase | GAPDH | 17 | 36 | 73 | -3.07 | -2.52 | 0.56 |
| 396 | P12268 | Inosine-5'-monophosphate dehydrogenase 2 | IMPDH2 | 7 | 55.8 | 23 | -3.07 | -2.86 | 0.21 |
| 397 | Q59EK1 | Adducin 3 isoform a variant | ADD3 | 4 | 78.2 | 10 | -3.06 | -2.64 | 0.42 |
| 398 | A0A024R3R5 | 3-beta-hydroxysterol Delta (14)-reductase | LBR | 2 | 70.7 | 6 | -3.06 | -2.28 | 0.78 |
| 399 | B9ZVN9 | DNA-directed RNA polymerase subunit | POLR1A | 3 | 187.7 | 4 | -3.06 | -2.39 | 0.67 |
| 400 | H0YME5 | Non-specific serine/threonine protein kinase | EIF2AK4 | 2 | 161.1 | 3 | -3.06 | -2.56 | 0.5 |
| 401 | E5RGM3 | S-phase kinase-associated protein 1 | SKP1 | 2 | 11.6 | 44 | -3.06 | -4.96 | -2.14 |
| 402 | U3KQC1 | WD repeat-containing protein 18 | WDR18 | 5 | 43.3 | 23 | -3.05 | -3.5 | -0.45 |
| 403 | B2RAR2 | RNA-binding protein NOB1 | NOB1 | 2 | 46.6 | 8 | -3.06 | -3.08 | -0.01 |
| 404 | P23378 | Glycine dehydrogenase (decarboxylating), mitochondrial | GLDC | 5 | 112.7 | 11 | -3.05 | -1.98 | 1.07 |
| 405 | A0A2R8YDA1 | Transcription activator BRG1 | SMARCA4 | 3 | 113.1 | 5 | -3.05 | -2.88 | 0.17 |
| 406 | Q5JW30 | Double-stranded RNA-binding protein Staufen homolog 1 | STAU1 | 5 | 54.7 | 10 | -3.04 | -3.11 | -0.07 |
| 407 | P07954 | Fumarate hydratase, mitochondrial | FH | 9 | 54.6 | 40 | -3.04 | -2.77 | 0.27 |
| 408 | Q14257 | Reticulocalbin-2 | RCN2 | 11 | 36.9 | 42 | -3.03 | -1.72 | 1.31 |
| 409 | Q9Y3I0 | RNA-splicing ligase RtcB homolog | RTCB | 6 | 55.2 | 26 | -3.03 | -3.33 | -0.3 |
| 410 | Q9Y2Q2 | FtsH homolog | FtsH | 4 | 57.6 | 18 | -3.04 | -2.49 | 0.54 |
| 411 | Q8TBR3 | Fusion (Involved in t(1216) in malignant liposarcoma) | FUS | 2 | 53.4 | 7 | -3.04 | -2.74 | 0.3 |
| 412 | B2R6F5 | Protein XRP2 | XRP2 | 2 | 39.6 | 11 | -3.03 | -2.36 | 0.67 |
| 413 | F5H669 | Cleavage and polyadenylation-specificity factor subunit 7 | CPSF7 | 3 | 41.2 | 10 | -3.03 | -3.51 | -0.49 |
| 414 | Q96KP4 | Cytosolic non-specific dipeptidase | CNDP2 | 14 | 52.8 | 41 | -3.03 | -1.95 | 1.08 |
| 415 | Q99797 | Mitochondrial intermediate peptidase | MIPEP | 4 | 80.6 | 10 | -3.02 | -1.21 | 1.81 |
| 416 | A0A5H1ZRP4 | Negative elongation factor B | NELFB | 5 | 70 | 16 | -3.02 | -3.08 | -0.05 |
| 417 | Q8N7H5 | RNA polymerase II-associated factor 1 homolog | PAF1 | 3 | 59.9 | 11 | -3.03 | -3.05 | -0.03 |
| 418 | Q9Y4W2 | Ribosomal biogenesis protein LAS1L | LAS1L | 4 | 83 | 15 | -3.02 | -1.6 | 1.43 |
| 419 | B3KMC9 | 5'-3' exoribonuclease | XRN | 9 | 108.5 | 19 | -3.01 | -1.33 | 1.68 |
| 420 | H7BXQ8 | Armadillo repeat-containing protein 10 | ARMC10 | 2 | 20.9 | 18 | -3.01 | -4.23 | -2.59 |
| 421 | H7BXH2 | Serine/threonine-protein phosphatase 6 regulatory subunit 3 | PPP6R3 | 4 | 92.4 | 9 | -3.01 | -3.99 | -0.98 |
| 422 | A0A0G2JLX3 | NACHT, LRR and PYD domains-containing protein 2 | NLRP2 | 4 | 118 | 8 | -3 | -0.38 | 2.62 |
| 423 | O00422 | Histone deacetylase complex subunit SAP18 | SAP18 | 3 | 17.6 | 27 | -3 | -2.04 | 0.96 |
| 424 | Q9NYU2 | UDP-glucose:glycoprotein glucosyltransferase 1 | UGGT1 | 19 | 177.1 | 23 | -3 | -3.56 | -0.56 |
| 425 | Q8WYJ5 | Protein kinase C inhibitor-2 | iPKC | 2 | 13.9 | 29 | -2.99 | -3.4 | -0.43 |
| 426 | A0A087WUL9 | 26S proteasome non-ATPase regulatory subunit 13 | PSMD13 | 3 | 42.7 | 12 | -2.99 | -3.09 | -0.1 |
| 427 | C9J1V9 | EEF1E1-BLOC1S5 readthrough (NMD candidate) | EEF1E1-BLOC1S5 | 2 | 17 | 25 | -2.99 | -1.66 | 1.33 |
| 428 | P00558 | Phosphoglycerate kinase 1 | PGK1 | 8 | 44.6 | 31 | -2.99 | -3.29 | -0.3 |
| 429 | Q86XP3 | ATP-dependent RNA helicase DDX42 | DDX42 | 7 | 102.9 | 17 | -2.99 | -2.43 | 0.56 |
| 430 | Q8IVF2 | Protein AHNAK2 | AHNAK2 | 39 | 616.2 | 16 | -2.99 | -3.65 | -0.66 |
| 431 | K7EQ55 | DAZ-associated protein 1 | DAZAP1 | 2 | 43.3 | 8 | -2.98 | -3.26 | -0.27 |
| 432 | Q9BZF3 | Oxysterol-binding protein-related protein 6 | OSBPL6 | 2 | 106.2 | 5 | -2.97 | -2.02 | 0.95 |
| 433 | Q9NX62 | Golgi-resident adenosine 3',5'-bisphosphate 3'-phosphatase | BPNT2 | 4 | 38.7 | 29 | -2.97 | -6.23 | -2.97 |
| 434 | Q59GM2 | Calpain catalytic domain-containing protein | CAPN | 4 | 76.6 | 15 | -2.98 | -2.36 | 0.61 |
| 435 | Q6LAF7 | Cathepsin L | CTSL | 2 | 19.1 | 27 | -2.97 | -2 | 0.97 |
| 436 | J3KR12 | Pyrroline-5-carboxylate reductase | PYCR1 | 4 | 36.7 | 16 | -2.98 | -2.79 | 0.19 |
| 437 | J3KQ48 | Aminoacyl-tRNA hydrolase | PTRH2 | 2 | 19.3 | 16 | -2.97 | -2.46 | 0.51 |
| 438 | Q4R9M9 | Kinesin family member 1Bbeta isoform II | KIF1B | 4 | 203.5 | 3 | -2.97 | -3.14 | -0.18 |
| 439 | O00399 | Dynactin subunit 6 | DCTN6 | 2 | 20.7 | 17 | -2.97 | -1.29 | 1.67 |
| 440 | Q53FR4 | Vacuolar protein sorting 35 variant | VPS35 | 5 | 91.6 | 12 | -2.96 | -2.16 | 0.79 |
| 441 | V5IRT4 | Mitochondrial nucleoid factor 1 | UQCC2 | 2 | 14.8 | 13 | -2.96 | -3.46 | -0.5 |
| 442 | A0A024R1Y2 | ATP-citrate synthase | ACLY | 13 | 119.7 | 20 | -2.95 | -3.36 | -0.4 |
| 443 | O60486 | Plexin-C1 | PLXNC1 | 2 | 175.6 | 4 | -2.95 | -3.67 | -0.72 |
| 444 | Q02952 | A-kinase anchor protein 12 | AKAP12 | 15 | 191.4 | 18 | -2.95 | -3.97 | -1.03 |
| 445 | Q5IJ48 | Protein crumbs homolog 2 | CRB2 | 5 | 134.2 | 9 | -2.94 | -3.43 | -0.48 |
| 446 | B2R791 | Pre-mRNA-splicing factor 3 | PRP3 | 4 | 77.5 | 16 | -2.95 | -3.12 | -0.18 |
| 447 | P12814 | Alpha-actinin-1 | ACTN1 | 11 | 103 | 33 | -2.94 | -3.2 | -0.25 |
| 448 | Q9Y3D0 | Cytosolic iron-sulfur assembly component 2B | CIAO2B | 2 | 17.7 | 26 | -2.93 | -2.46 | 0.47 |
| 449 | Q5M7Z5 | GRHPR protein | GRHPR | 6 | 36.8 | 24 | -2.94 | -3.8 | -0.87 |
| 450 | P13984 | General transcription factor IIF subunit 2 | GTF2F2 | 3 | 28.4 | 25 | -2.93 | -2.35 | 0.59 |
| 451 | P43686 | 26S proteasome regulatory subunit 6B | PSMC4 | 10 | 47.3 | 50 | -2.93 | -2.98 | -0.05 |
| 452 | Q7L2H7 | Eukaryotic translation initiation factor 3 subunit M | EIF3M | 5 | 42.5 | 24 | -2.92 | -1.51 | 1.41 |
| 453 | E9PLD0 | Ras-related protein Rab-1B | RAB1B | 2 | 18.5 | 22 | -2.92 | -2.83 | 0.09 |
| 454 | A8K0T9 | F-actin-capping protein subunit alpha | α- CPA | 4 | 32.9 | 28 | -2.92 | -3.94 | -1.02 |
| 455 | E7EX90 | Dynactin subunit 1 | DCTN1 | 8 | 139 | 10 | -2.93 | -2.47 | 0.46 |
| 456 | A0A024RAB0 | Endothelin-converting enzyme 1 | ECE1 | 3 | 85.8 | 7 | -2.93 | -3.03 | -0.11 |
| 457 | A0A0S2Z537 | E3 ubiquitin protein ligase | RNF40 | 2 | 113.6 | 5 | -2.91 | -5.25 | -2.34 |
| 458 | O00151 | PDZ and LIM domain protein 1 | PDLIM1 | 2 | 36 | 15 | -2.91 | -3.15 | -0.24 |
| 459 | P18583 | Protein SON | SON | 4 | 263.7 | 9 | -2.91 | -3.34 | -0.43 |
| 460 | Q9UIG0 | Tyrosine-protein kinase BAZ1B | BAZ1B | 2 | 170.8 | 2 | -2.91 | -2.69 | 0.22 |
| 461 | Q53ET4 | Serine hydroxymethyltransferase | SHMT | 15 | 55.9 | 41 | -2.9 | -2.84 | 0.06 |
| 462 | E7EVA0 | Microtubule-associated protein | MAP4 | 3 | 245.3 | 8 | -2.89 | -2.05 | 0.84 |
| 463 | Q6UN15 | Pre-mRNA 3'-end-processing factor FIP1 | FIP1L1 | 5 | 66.5 | 18 | -2.89 | -3.61 | -0.72 |
| 464 | Q9BV86 | N-terminal Xaa-Pro-Lys N-methyltransferase 1 | NTMT1 | 3 | 25.4 | 25 | -2.88 | -3.22 | -0.34 |
| 465 | Q9GZS3 | SKI8 subunit of superkiller complex protein | SKIC8 | 5 | 33.6 | 32 | -2.89 | -2.65 | 0.24 |
| 466 | A0A0G2JNW7 | Solute carrier family 12 member 7 | SLC12A7 | 2 | 119.1 | 3 | -2.89 | -2.86 | 0.03 |
| 467 | Q53GL5 | IDP | IDP | 6 | 50.9 | 25 | -2.89 | -2.74 | 0.15 |
| 468 | Q7Z3T9 | Neuropilin | NRP | 3 | 104.7 | 5 | -2.88 | -3.02 | -0.14 |
| 469 | Q53HH4 | Ras-GTPase-activating protein SH3-domain-binding protein variant | G3BP | 4 | 52.1 | 14 | -2.88 | -2.14 | 0.74 |
| 470 | S4R468 | Calcium uniporter protein | MCU | 3 | 19 | 31 | -2.88 | -4.5 | -1.62 |
| 471 | P61011 | Signal recognition particle subunit SRP54 | SRP54 | 6 | 55.7 | 16 | -2.88 | -1.8 | 1.08 |
| 472 | Q96KA5 | Lipid scramblase CLPTM1L | CLPTM1L | 2 | 62.2 | 11 | -2.88 | -3.64 | -0.76 |
| 473 | A8K3R2 | Ribosome biogenesis protein BOP1 | BOP1 | 5 | 83.5 | 17 | -2.87 | -4 | -1.13 |
| 474 | Q09666 | Neuroblast differentiation-associated protein AHNAK | AHNAK | 38 | 628.7 | 14 | -2.87 | -2.55 | 0.32 |
| 475 | Q9NZJ4 | Sacsin | SACS | 5 | 520.8 | 3 | -2.87 | -0.81 | 2.06 |
| 476 | E7ET15 | U2 snRNP-associated SURP motif-containing protein | U2SURP | 7 | 118.2 | 14 | -2.86 | -1.78 | 1.09 |
| 477 | P16615 | Sarcoplasmic/endoplasmic reticulum calcium ATPase 2 | ATP2A2 | 14 | 114.7 | 25 | -2.84 | -2.13 | 0.72 |
| 478 | P61221 | ATP-binding cassette sub-family E member 1 | ABCE1 | 5 | 67.3 | 19 | -2.84 | -2.24 | 0.61 |
| 479 | Q15691 | Microtubule-associated protein RP/EB family member 1 | MAPRE1 | 5 | 30 | 39 | -2.85 | -1.8 | 1.04 |
| 480 | Q7Z4V5 | Hepatoma-derived growth factor-related protein 2 | HDGFL2 | 3 | 74.3 | 8 | -2.85 | -1.25 | 1.6 |
| 481 | Q53F20 | Acidic (Leucine-rich) nuclear phosphoprotein 32 family, member E variant | ANP32A | 6 | 30.4 | 35 | -2.85 | -3.39 | -0.54 |
| 482 | Q53HG0 | Eukaryotic translation initiation factor 3, subunit 3 gamma, 40kDa variant | EIF3 | 3 | 39.8 | 13 | -2.85 | -2.26 | 0.59 |
| 483 | A8K666 | Phenylalanyl-tRNA synthetase beta subunit | PheRS | 8 | 66 | 20 | -2.84 | -2 | 0.84 |
| 484 | B2RDX5 | Threonyl-tRNA synthetase | TRS | 6 | 82.1 | 14 | -2.83 | -1.64 | 1.19 |
| 485 | Q4LE35 | ITGA7 variant protein | ITGA7 | 7 | 130.7 | 10 | -2.84 | -3.11 | -0.28 |
| 486 | A0A087WUD7 | Sec1 family domain-containing protein 2 | SCFD2 | 4 | 47.8 | 18 | -2.83 | -0.33 | 2.5 |
| 487 | P42574 | Caspase-3 | CASP3 | 3 | 31.6 | 18 | -2.83 | -1.93 | 0.9 |
| 488 | P51114 | RNA-binding protein FXR1 | FXR1 | 5 | 69.7 | 13 | -2.83 | -1.86 | 0.97 |
| 489 | P61964 | WD repeat-containing protein 5 | WDR5 | 3 | 36.6 | 19 | -2.83 | -3.6 | -0.76 |
| 490 | A0A140VJN8 | Perilipin | PLIN3 | 12 | 46.9 | 46 | -2.83 | -3.22 | -0.39 |
| 491 | Q99653 | Calcineurin B homologous protein 1 | CHP1 | 2 | 22.4 | 19 | -2.82 | -2.09 | 0.73 |
| 492 | A0A087WTU3 | Testis-expressed protein 264 | TEX264 | 3 | 26 | 33 | -2.83 | -3 | -0.17 |
| 493 | Q5QPP4 | UDP-glucose 4-epimerase | GALE | 3 | 26.4 | 24 | -2.83 | -3 | -0.18 |
| 494 | A5YM53 | ITGAV protein | ITGAV | 9 | 116 | 17 | -2.83 | -3.42 | -0.59 |
| 495 | B4DEX8 | S-adenosylmethionine synthase | MAT2A | 6 | 39.7 | 40 | -2.82 | -3.9 | -1.08 |
| 496 | P07814 | Bifunctional glutamate/proline--tRNA ligase | EPRS1 | 19 | 170.5 | 21 | -2.82 | -1.98 | 0.84 |
| 497 | A0A0S2Z3I6 | G protein-coupled receptor kinase | ADRBK1 | 3 | 77.4 | 11 | -2.81 | -2.74 | 0.08 |
| 498 | P16152 | Carbonyl reductase [NADPH] 1 | CBR1 | 7 | 30.4 | 31 | -2.81 | -1.18 | 1.63 |
| 499 | Q9BY32 | Inosine triphosphate pyrophosphatase | ITPA | 4 | 21.4 | 42 | -2.8 | -3.13 | -0.33 |
| 500 | A0A087WZT2 | Methyltransferase-like protein 7B | METTL7B | 4 | 31.3 | 26 | -2.81 | -1.66 | 1.15 |
| 501 | B2R5U1 | Staphylococcal nuclease domain-containing protein | SND1 | 11 | 99.6 | 21 | -2.8 | -3.05 | -0.25 |
| 502 | P20700 | Lamin-B1 | LMNB1 | 10 | 66.4 | 25 | -2.79 | -3.14 | -0.34 |
| 503 | A0A494C1E2 | Phosphoacetylglucosamine mutase | PGM3 | 3 | 51 | 15 | -2.78 | -3 | -0.22 |
| 504 | A0A0C4DGA2 | Enoyl-CoA delta isomerase 2, mitochondrial | ECI2 | 5 | 40.2 | 32 | -2.78 | -2.5 | 0.28 |
| 505 | O95865 | N(G),N(G)-dimethylarginine dimethylaminohydrolase 2 | DDAH2 | 4 | 29.6 | 42 | -2.79 | -4.35 | -1.56 |
| 506 | P13489 | Ribonuclease inhibitor | RNH1 | 10 | 49.9 | 47 | -2.78 | -0.86 | 1.92 |
| 507 | Q9BS26 | Endoplasmic reticulum resident protein 44 | ERP44 | 3 | 46.9 | 14 | -2.78 | -3.28 | -0.5 |
| 508 | Q05CT2 | FAM98A protein | FAM98A | 4 | 20.7 | 51 | -2.77 | -1.64 | 1.13 |
| 509 | D7P639 | NADH-ubiquinone oxidoreductase chain 5 | ND5 | 2 | 66.7 | 4 | -2.77 | -2.86 | 0.36 |
| 510 | A0A024R652 | C-1-tetrahydrofolate synthase, cytoplasmic | MTHFD1 | 23 | 101.5 | 46 | -2.77 | -2.41 | 0.36 |
| 511 | P49903 | Selenide, water dikinase 1 | SEPHS1 | 6 | 42.9 | 33 | -2.77 | -2.05 | 0.71 |
| 512 | Q92520 | Protein FAM3C | FAM3C | 3 | 24.7 | 16 | -2.77 | -2.87 | -0.11 |
| 513 | A0A1L1ZH79 | NADH-ubiquinone oxidoreductase chain 5 | ND5 | 3 | 66.7 | 9 | -2.75 | -1.55 | 1.2 |
| 514 | P00390 | Glutathione reductase, mitochondrial | GSR | 9 | 56.2 | 36 | -2.76 | -2.39 | 0.36 |
| 515 | B2RBJ8 | Glutamyl-tRNA(Gln) amidotransferase subunit A, mitochondrial | QRSL1 | 3 | 57.4 | 17 | -2.75 | -4.48 | -1.73 |
| 516 | A7BI36 | p180/ribosome receptor | RRBP1 | 4 | 165.6 | 4 | -2.76 | -2.64 | 0.12 |
| 517 | A0A024R9K5 | Zinc finger, FYVE domain containing 19, isoform | ZFYVE19 | 2 | 32.9 | 19 | -2.75 | -1.02 | 1.73 |
| 518 | G8JLG1 | Structural maintenance of chromosomes protein | SMC1A | 3 | 140.8 | 4 | -2.75 | -2.44 | 0.31 |
| 519 | A0A0S2Z4X4 | Methylmalonyl-CoA isomerase | MUT | 8 | 75.5 | 24 | -2.74 | -2.95 | -0.21 |
| 520 | O75691 | Small subunit processome component 20 homolog | UTP20 | 3 | 318.2 | 2 | -2.74 | 0.31 | 3.05 |
| 521 | Q1JQ76 | Ribosomal protein | RPL10A | 4 | 23.5 | 20 | -2.73 | -2.05 | 0.68 |
| 522 | Q9BUF5 | Tubulin beta-6 chain | TUBB6 | 7 | 49.8 | 54 | -2.72 | -1.78 | 0.94 |
| 523 | Q9NRY5 | Protein FAM114A2 | FAM114A2 | 4 | 55.4 | 16 | -2.72 | -2.82 | -0.1 |
| 524 | A0A1B0GTL7 | Contactin-2 | CNTN2 | 2 | 110.3 | 6 | -2.73 | -2.34 | 0.39 |
| 525 | A0A1W2PRF6 | Lysosome membrane protein 2 | SCARB2 | 2 | 36.3 | 13 | -2.73 | -1.25 | 1.48 |
| 526 | Q8TDQ7 | Glucosamine-6-phosphate isomerase 2 | GNPDA2 | 2 | 31.1 | 23 | -2.72 | -1.78 | 0.94 |
| 527 | Q9UHV9 | Prefoldin subunit 2 | PFDN2 | 3 | 16.6 | 29 | -2.71 | -2.14 | 0.57 |
| 528 | O96019 | Actin-like protein 6A | ACTL6A | 4 | 47.4 | 21 | -2.72 | -3.72 | -1 |
| 529 | F8VRH0 | Poly(rC)-binding protein 2 | PCBP2 | 4 | 32 | 37 | -2.71 | -1.95 | 0.75 |
| 530 | H3BUY0 | 39S ribosomal protein L21, mitochondrial | MRPL21 | 2 | 21.5 | 20 | -2.7 | -1.55 | 1.14 |
| 531 | P61088 | Ubiquitin-conjugating enzyme E2 N | UBE2N | 2 | 17.1 | 32 | -2.7 | -2.3 | 0.4 |
| 532 | Q9HCN4 | GPN-loop GTPase 1 | GPN1 | 2 | 41.7 | 17 | -2.7 | -3.76 | -1.06 |
| 533 | Q9UKD2 | mRNA turnover protein 4 homolog | MRTO4 | 3 | 27.5 | 16 | -2.7 | -1.96 | 0.74 |
| 534 | C9JQ00 | Tubulin alpha chain | TUBA4A | 2 | 19.8 | 31 | -2.7 | -0.7 | 1.99 |
| 535 | A1X283 | SH3 and PX domain-containing protein 2B | SH3PXD2B | 2 | 101.5 | 5 | -2.69 | -2.37 | 0.32 |
| 536 | A0A024RAD5 | Dolichyl-diphosphooligosaccharide--protein glycosyltransferase 48 kDa subunit | DDOST | 10 | 50.7 | 40 | -2.68 | -1.48 | 1.2 |
| 537 | Q53FT8 | Proteasome subunit beta | PSMB5 | 2 | 26.5 | 16 | -2.68 | -2.41 | 0.27 |
| 538 | Q17RG0 | ATG16L1 protein | ATG16L1 | 3 | 64.9 | 11 | -2.68 | -1.58 | 1.1 |
| 539 | O15116 | U6 snRNA-associated Sm-like protein LSm1 | LSM1 | 2 | 15.2 | 26 | -2.68 | -2.88 | -0.2 |
| 540 | P25325 | 3-mercaptopyruvate sulfurtransferase | MPST | 3 | 33.2 | 15 | -2.68 | -2.64 | 0.04 |
| 541 | Q13085 | Acetyl-CoA carboxylase 1 | ACACA | 13 | 265.4 | 11 | -2.68 | -2.87 | -0.18 |
| 542 | Q6P2I3 | Fumarylacetoacetate hydrolase domain-containing protein 2B | FAHD2B | 7 | 34.6 | 30 | -2.68 | -3.32 | -0.64 |
| 543 | H3BTA2 | Serine/threonine-protein phosphatase | PPP4C | 4 | 30.5 | 35 | -2.67 | -2.8 | -0.13 |
| 544 | A0A024R1X8 | Junction plakoglobin | JUP | 3 | 81.7 | 13 | -2.67 | -1.44 | 1.23 |
| 545 | B4DDP9 | Selenocysteine lyase, isoform | SCLY | 2 | 44.8 | 12 | -2.67 | -2.59 | 0.09 |
| 546 | A0A0A6YY92 | Adenylosuccinate lyase | ADSL | 5 | 56.2 | 16 | -2.67 | -1.97 | 0.7 |
| 547 | B4DKY1 | Cysteinyl-tRNA synthetase | CARS1 | 9 | 84.2 | 18 | -2.67 | -3.12 | -0.45 |
| 548 | A0A087WW40 | Endophilin-B1 | SH3GLB1 | 3 | 44.2 | 13 | -2.67 | -3.01 | -0.34 |
| 549 | P00374 | Dihydrofolate reductase | DHFR | 2 | 21.4 | 20 | -2.68 | -2.59 | 0.08 |
| 550 | P28070 | Proteasome subunit beta type-4 | PSMB4 | 4 | 29.2 | 33 | -2.67 | -2.7 | -0.03 |
| 551 | Q6ZRP7 | Sulfhydryl oxidase 2 | QSOX2 | 3 | 77.5 | 9 | -2.67 | -1.86 | 0.81 |
| 552 | Q9BXW7 | Haloacid dehalogenase-like hydrolase domain-containing 5 | HDHD5 | 5 | 46.3 | 26 | -2.67 | -1.22 | 1.45 |
| 553 | H0Y8P4 | U3 small nucleolar RNA-associated protein 15 homolog | UTP15 | 2 | 61.4 | 9 | -2.67 | -1.28 | 1.38 |
| 554 | B4DZ55 | Dolichyl-phosphate-mannose--protein mannosyltransferase | POMT1 | 2 | 103.8 | 3 | -2.65 | -2.72 | -0.07 |
| 555 | V9HW38 | Cytosol aminopeptidase | HEL-S-106 | 13 | 56 | 37 | -2.66 | -2.46 | 0.19 |
| 556 | Q2TU84 | Aspartate aminotransferase | GIG18 | 6 | 46.3 | 30 | -2.65 | -1.48 | 1.18 |
| 557 | A0A0U1RQD1 | Chromosome 12 open reading frame 5, isoform | TIGAR | 4 | 23.6 | 39 | -2.65 | -2.3 | 0.35 |
| 558 | F5H1S8 | Malectin | MLEC | 3 | 16.7 | 36 | -2.65 | -2.1 | 0.54 |
| 559 | P30086 | Phosphatidylethanolamine-binding protein 1 | PEBP1 | 3 | 21 | 37 | -2.64 | -1.35 | 1.3 |
| 560 | P31930 | Cytochrome b-c1 complex subunit 1, mitochondrial | UQCRC1 | 9 | 52.6 | 26 | -2.65 | -3.81 | -1.17 |
| 561 | D3DW56 | Ubiquitin-like 7 (Bone marrow stromal cell-derived), isoform | UBL7 | 2 | 45.1 | 10 | -2.65 | -2.8 | -0.15 |
| 562 | K7EP71 | Heparan sulfate 2-O-sulfotransferase 1 | HS2ST1 | 2 | 12.9 | 32 | -2.64 | -2.77 | -0.13 |
| 563 | Q9BUA3 | Spindlin interactor and repressor of chromatin-binding protein | SPINDOC | 2 | 41 | 8 | -2.64 | -3.7 | -1.06 |
| 564 | Q9BW85 | Splicing factor YJU2 | YJU2 | 3 | 37.1 | 13 | -2.64 | -3.29 | -0.65 |
| 565 | E9PRJ8 | Tetraspanin | CD81 | 2 | 22.5 | 19 | -2.63 | -2.24 | 0.4 |
| 566 | Q9BW34 | EEF1D protein | EEF1D | 7 | 60.8 | 18 | -2.63 | -2.36 | 0.28 |
| 567 | A0A1S5UZH4 | Neuronal cell adhesion molecule | NRCAM | 5 | 129.2 | 10 | -2.64 | -1.85 | 0.79 |
| 568 | V9HWC6 | Peptidyl-prolyl cis-trans isomerase | PPIs | 4 | 22.7 | 25 | -2.63 | -1.57 | 1.06 |
| 569 | P30041 | Peroxiredoxin-6 | PRDX6 | 5 | 25 | 29 | -2.63 | -2.64 | -0.02 |
| 570 | Q59G46 | Thioredoxin-like 1 variant | TXNL1 | 3 | 31.3 | 28 | -2.63 | -2.84 | -0.22 |
| 571 | O75533 | Splicing factor 3B subunit 1 | SF3B1 | 9 | 145.7 | 14 | -2.62 | -1.81 | 0.81 |
| 572 | P11172 | Uridine 5'-monophosphate synthase | UMPS | 4 | 52.2 | 19 | -2.62 | -2.3 | 0.32 |
| 573 | K7ER16 | Phenylalanine--tRNA ligase alpha subunit | FARSA | 4 | 26 | 24 | -2.61 | -3.17 | -0.56 |
| 574 | Q9UQ03 | Coronin-2B | CORO2B | 3 | 54.9 | 12 | -2.62 | -1.99 | 0.63 |
| 575 | Q9Y570 | Protein phosphatase methylesterase 1 | PPME1 | 3 | 42.3 | 15 | -2.62 | 0.51 | 3.13 |
| 576 | G5EA09 | Syndecan binding protein (Syntenin), isoform | SDCBP | 4 | 34.8 | 31 | -2.62 | -3.34 | -0.72 |
| 577 | B2RBD5 | Tubulin beta chain | TUBB | 8 | 50.4 | 48 | -2.62 | -2.25 | 0.37 |
| 578 | A8K4Z4 | 60S acidic ribosomal protein P0 | RPLP2 | 8 | 34.2 | 37 | -2.61 | -2.67 | -0.06 |
| 579 | P09110 | 3-ketoacyl-CoA thiolase, peroxisomal | ACAA1 | 5 | 44.3 | 20 | -2.61 | -2.77 | -0.16 |
| 580 | P41091 | Eukaryotic translation initiation factor 2 subunit 3 | EIF2S3 | 4 | 51.1 | 17 | -2.61 | -2.48 | 0.13 |
| 581 | P55036 | 26S proteasome non-ATPase regulatory subunit 4 | PSMD4 | 6 | 40.7 | 32 | -2.61 | -2.67 | -0.07 |
| 582 | Q6IBR0 | Dolichyl-diphosphooligosaccharide--protein glycosyltransferase subunit 1 | RPN1 | 7 | 68.6 | 18 | -2.59 | -2.41 | 0.18 |
| 583 | Q59GJ0 | Eukaryotic translation initiation factor 4 gamma, 3 variant | EIF4G3 | 5 | 195.8 | 5 | -2.59 | -2.81 | -0.22 |
| 584 | Q53FG6 | Splicing factor 3b, subunit 4 variant | SF3B4 | 3 | 44.4 | 14 | -2.59 | -4.27 | -1.67 |
| 585 | Q75L23 | 26S proteasome AAA-ATPase subunit RPT1 | PSMC2 | 4 | 45.8 | 19 | -2.59 | -1.45 | 1.14 |
| 586 | Q12792 | Twinfilin-1 | TWF1 | 4 | 40.3 | 22 | -2.59 | -1.99 | 0.6 |
| 587 | Q9GZZ9 | Ubiquitin-like modifier-activating enzyme 5 | UBA5 | 2 | 44.8 | 12 | -2.59 | -3.99 | -1.4 |
| 588 | Q9H6T3 | RNA polymerase II-associated protein 3 | RPAP3 | 4 | 75.7 | 14 | -2.59 | -1.65 | 0.93 |
| 589 | B4DN59 | CD44 antigen | CD44 | 2 | 36 | 7 | -2.58 | -3.09 | -0.51 |
| 590 | Q6FHU3 | PSME1 protein | PSME1 | 3 | 28.7 | 18 | -2.58 | -2.18 | 0.4 |
| 591 | P05023 | Sodium/potassium-transporting ATPase subunit alpha-1 | ATP1A1 | 2 | 112.8 | 17 | -2.58 | -2.06 | 0.52 |
| 592 | P56537 | Eukaryotic translation initiation factor 6 | EIF6 | 3 | 26.6 | 29 | -2.58 | -1.83 | 0.75 |
| 593 | Q9NVH0 | Exonuclease 3'-5' domain-containing protein 2 | EXD2 | 4 | 70.3 | 13 | -2.58 | -2.81 | -0.23 |
| 594 | Q9P265 | Disco-interacting protein 2 homolog B | DIP2B | 4 | 171.4 | 7 | -2.58 | -1.64 | 0.94 |
| 595 | A0A2R8Y566 | RAB11-binding protein RELCH | RELCH | 2 | 134.5 | 5 | -2.57 | -4.03 | -1.46 |
| 596 | A0A087WUZ3 | Spectrin beta chain | SPTBN1 | 17 | 274.7 | 14 | -2.57 | -2.21 | 0.36 |
| 597 | A0A6Q8PFX2 | Tyrosine--tRNA ligase, cytoplasmic | YARS1 | 3 | 53.6 | 11 | -2.58 | -2.59 | -0.01 |
| 598 | E5RI99 | 60S ribosomal protein L30 | RPL30 | 4 | 12.6 | 37 | -2.58 | -1.62 | 0.95 |
| 599 | P40855 | Peroxisomal biogenesis factor 19 | PEX19 | 4 | 32.8 | 28 | -2.57 | -2.58 | -0.01 |
| 600 | Q6UXN9 | WD repeat-containing protein 82 | WDR82 | 5 | 35.1 | 36 | -2.57 | -1.19 | 1.38 |
| 601 | Q9UBV8 | Peflin | PEF1 | 2 | 30.4 | 15 | -2.58 | -1.98 | 0.59 |
| 602 | Q5VXN0 | Ribosome production factor 2 homolog | RPF2 | 3 | 24.6 | 15 | -2.56 | -2.65 | -0.09 |
| 603 | R4GMR5 | 26S proteasome non-ATPase regulatory subunit 8 | PSMD8 | 3 | 32.5 | 20 | -2.57 | -2.26 | 0.3 |
| 604 | Q7Z759 | CCT-theta | CCT8 | 8 | 54.1 | 29 | -2.56 | -3.33 | -0.77 |
| 605 | A0A024R4E5 | High density lipoprotein binding protein (Vigilin), isoform | HDLBP | 13 | 141.4 | 18 | -2.56 | -2.21 | 0.35 |
| 606 | C9JND6 | Programmed cell death protein 10 | PDCD10 | 2 | 16.8 | 26 | -2.55 | -1.83 | 0.73 |
| 607 | Q5VT52 | Regulation of nuclear pre-mRNA domain-containing protein 2 | RPRD2 | 6 | 155.9 | 8 | -2.55 | -1.78 | 0.78 |
| 608 | B2R7C5 | DNA helicase | ATRX | 12 | 91 | 25 | -2.56 | -2.14 | 0.41 |
| 609 | A8K5W7 | Isoleucyl-tRNA synthetase | IARS1 | 7 | 105.9 | 19 | -2.56 | -3.76 | -1.2 |
| 610 | B2R858 | RNA helicase | DHX9 | 5 | 53.2 | 15 | -2.55 | -2.2 | 0.35 |
| 611 | Q8TC62 | Septin-7 | Sep-07 | 8 | 48.6 | 25 | -2.55 | -1.81 | 0.74 |
| 612 | H0Y8X4 | 2'-deoxynucleoside 5'-phosphate N-hydrolase 1 | DNPH1 | 4 | 25.9 | 34 | -2.55 | -1.65 | 0.9 |
| 613 | A0A499FJL1 | Post-proline cleaving enzyme | PREP | 8 | 72.9 | 22 | -2.55 | -3.1 | -0.55 |
| 614 | E9PMI6 | Chloride channel, nucleotide sensitive 1A | CLNS1A | 3 | 18.2 | 22 | -2.54 | -2.12 | 0.43 |
| 615 | Q12797 | Aspartyl/asparaginyl beta-hydroxylase | ASPH | 4 | 85.8 | 11 | -2.54 | -2.29 | 0.25 |
| 616 | Q9C0H2 | Protein tweety homolog 3 | TTYH3 | 2 | 57.5 | 7 | -2.54 | -1.98 | 0.56 |
| 617 | E9PR70 | Serpin H1 | SERPINH1 | 12 | 32.6 | 53 | -2.53 | -2.8 | -0.27 |
| 618 | Q6ZVI6 | NADH-cytochrome b5 reductase | CYB5R | 5 | 28.9 | 46 | -2.53 | -2.77 | -0.23 |
| 619 | K7ELG9 | Protein LSM12 homolog | LSM12 | 4 | 24.9 | 33 | -2.54 | -0.87 | 1.66 |
| 620 | A7E2D7 | EP400 protein | EP400 | 2 | 188.1 | 5 | -2.52 | -0.39 | 2.14 |
| 621 | K7EQA9 | Hsp90 chaperone protein kinase-targeting subunit | CDC37 | 3 | 27.4 | 28 | -2.52 | -3.38 | -0.86 |
| 622 | F5H7W8 | Protein CUSTOS | C12orf43 | 2 | 31.7 | 7 | -2.52 | -2.52 | 0.01 |
| 623 | Q9UBU9 | Nuclear RNA export factor 1 | NXF1 | 4 | 70.1 | 17 | -2.51 | -1.6 | 0.91 |
| 624 | A0A087WWF6 | DNA polymerase delta subunit 2 | POLD2 | 2 | 54.7 | 6 | -2.52 | -4.2 | -1.69 |
| 625 | Q9BZQ8 | Protein Niban 1 | NIBAN1 | 2 | 103.1 | 4 | -2.51 | -3.28 | -0.77 |
| 626 | A0A1X7SBR3 | Glial fibrillary acidic protein | GFAP | 9 | 54.2 | 30 | -2.5 | -2.4 | 0.1 |
| 627 | P51398 | 28S ribosomal protein S29, mitochondrial | DAP3 | 2 | 45.5 | 11 | -2.5 | -2.36 | 0.14 |
| 628 | Q9Y265 | RuvB-like 1 | RUVBL1 | 14 | 50.2 | 43 | -2.5 | -2.87 | -0.38 |
| 629 | E5KLM0 | Dynamin-like 120 kDa protein, form S1 | OPA1 | 2 | 113.4 | 3 | -2.48 | -2.25 | 0.23 |
| 630 | A0A0A1HAN9 | H.sapiens ras-related Hrab5 protein | RAB5A | 4 | 23.4 | 27 | -2.48 | -2.89 | -0.41 |
| 631 | F8WBG8 | Drebrin-like protein | DBNL | 2 | 13.7 | 26 | -2.47 | -2.18 | 0.29 |
| 632 | Q53GW1 | Vesicle transport-related protein isoform a variant | GOT1A | 2 | 72.3 | 6 | -2.48 | -2.92 | -0.44 |
| 633 | J3KP58 | CAP-Gly domain-containing linker protein 1 | CLIP1 | 3 | 148 | 3 | -2.47 | -3.38 | -0.91 |
| 634 | P36405 | ADP-ribosylation factor-like protein 3 | ARL3 | 4 | 20.4 | 36 | -2.47 | -3.12 | -0.65 |
| 635 | A0A024RD08 | Mitochondrial carrier homolog 1 (C. elegans), isoform | MTCH1 | 2 | 39.9 | 21 | -2.46 | -1.33 | 1.13 |
| 636 | P11498 | Pyruvate carboxylase, mitochondrial | PC | 9 | 129.6 | 16 | -2.46 | -1.55 | 0.92 |
| 637 | Q9UKU7 | Isobutyryl-CoA dehydrogenase, mitochondrial | ACAD8 | 2 | 45 | 10 | -2.46 | -2.73 | -0.27 |
| 638 | B4DHW9 | 4'-phosphopantetheine phosphatase | PANK4 | 3 | 86.9 | 7 | -2.45 | -2.5 | -0.05 |
| 639 | Q59H65 | Aldehyde dehydrogenase 3A2 variant | ALDH3A2 | 5 | 51.9 | 26 | -2.45 | -2.93 | -0.48 |
| 640 | Q9HA11 | Histone H2A | H2AC | 2 | 29.9 | 19 | -2.45 | -1.12 | 1.32 |
| 641 | A0A024R850 | Mitochondrial ribosome recycling factor, isoform | MRRF | 3 | 36.9 | 16 | -2.45 | -2.97 | -0.52 |
| 642 | Q9NQ50 | 39S ribosomal protein L40, mitochondrial | MRPL40 | 2 | 24.5 | 17 | -2.45 | -2.09 | 0.36 |
| 643 | F5GZQ3 | Trifunctional enzyme subunit beta, mitochondrial | HADHB | 6 | 49.6 | 27 | -2.45 | -2.63 | -0.19 |
| 644 | A0A2R8Y6J3 | 60S ribosomal protein L5 | RPL5 | 2 | 27 | 22 | -2.44 | -2.08 | 0.36 |
| 645 | A0A3B3IRT8 | Signal sequence receptor subunit alpha | SSR1 | 2 | 32.4 | 14 | -2.44 | -1.93 | 0.51 |
| 646 | P60842 | Eukaryotic initiation factor 4A-I | EIF4A1 | 8 | 46.1 | 48 | -2.42 | -2.77 | -0.34 |
| 647 | B4DZJ7 | Transcription elongation factor SPT5 | SPT5 | 7 | 118.1 | 17 | -2.43 | -2.67 | -0.24 |
| 648 | C9J2Y9 | DNA-directed RNA polymerase subunit beta | POLR2B | 5 | 133 | 10 | -2.42 | -2.88 | -0.47 |
| 649 | Q96F88 | Processing of 1, ribonuclease P/MRP subunit (S. cerevisiae) | POP1 | 3 | 114.6 | 7 | -2.41 | -2.69 | -0.28 |
| 650 | A0A3B3ITJ4 | Heterogeneous nuclear ribonucleoprotein L | HNRNPL | 8 | 59.2 | 28 | -2.41 | -2.95 | -0.53 |
| 651 | A0A0C4DGN7 | NADPH:adrenodoxin oxidoreductase, mitochondrial | FDXR | 6 | 54.4 | 24 | -2.41 | -2.22 | 0.19 |
| 652 | P78527 | DNA-dependent protein kinase catalytic subunit | PRKDC | 27 | 468.8 | 11 | -2.41 | -0.96 | 1.45 |
| 653 | Q13148 | TAR DNA-binding protein 43 | TARDBP | 7 | 44.7 | 37 | -2.41 | -0.83 | 1.58 |
| 654 | Q9Y281 | Cofilin-2 | CFL2 | 2 | 18.7 | 20 | -2.41 | -2.46 | -0.05 |
| 655 | Q53HM6 | Splicing factor 3a, subunit 3 variant | SF3A3 | 3 | 58.8 | 12 | -2.4 | -2.11 | 0.29 |
| 656 | Q5HY57 | Emerin | EMD | 4 | 24.9 | 28 | -2.4 | -2.42 | -0.02 |
| 657 | D3DVQ1 | Leucine zipper-EF-hand-containing transmembrane protein 1 | LETM1 | 9 | 63.5 | 25 | -2.39 | -2.61 | -0.22 |
| 658 | H3BSK8 | E3 ubiquitin-protein ligase RBBP6 | RBBP6 | 2 | 113.4 | 4 | -2.38 | -3.37 | -1 |
| 659 | A8K548 | Modulator of non-genomic activity of estrogen receptor | caveolin-1 | 5 | 119.6 | 10 | -2.37 | -2.42 | -0.05 |
| 660 | H0YCY6 | ATP-dependent dihydroxyacetone kinase | TKFC | 8 | 54.6 | 27 | -2.37 | -2.64 | -0.27 |
| 661 | F5H039 | Molybdopterin molybdenumtransferase | GPHN | 5 | 84.7 | 14 | -2.37 | -2.69 | -0.32 |
| 662 | P17987 | T-complex protein 1 subunit alpha | TCP1 | 17 | 60.3 | 50 | -2.37 | -1.8 | 0.57 |
| 663 | P23528 | Cofilin-1 | CFL1 | 5 | 18.5 | 39 | -2.37 | -2.35 | 0.02 |
| 664 | Q14008 | Cytoskeleton-associated protein 5 | CKAP5 | 13 | 225.4 | 11 | -2.36 | -0.31 | 2.06 |
| 665 | B3KSH1 | Eukaryotic translation initiation factor 3 subunit F | EIF3F | 5 | 39.1 | 27 | -2.37 | -2.12 | 0.25 |
| 666 | A0A024RCA6 | Solute carrier family 25 (Mitochondrial carrier: glutamate), member 22, isoform | SLC25 | 2 | 26.6 | 18 | -2.36 | -3.04 | -0.67 |
| 667 | Q96AJ9 | Vesicle transport through interaction with t-SNAREs homolog 1A | VTI1A | 2 | 25.2 | 15 | -2.36 | -1.05 | 1.31 |
| 668 | B2R7L2 | Annexin | ANNEXIN | 2 | 50.3 | 7 | -2.35 | -3.63 | -1.28 |
| 669 | Q63HL4 | NADPH--cytochrome P450 reductase | DKFZp686G04235 | 7 | 77 | 18 | -2.35 | -1.99 | 0.37 |
| 670 | A0A087WW65 | ATP-binding cassette sub-family B member 7, mitochondrial | ABCB7 | 6 | 78.2 | 12 | -2.35 | -0.87 | 1.48 |
| 671 | O15269 | Serine palmitoyltransferase 1 | SPTLC1 | 3 | 52.7 | 12 | -2.35 | -1.88 | 0.47 |
| 672 | C6EMX8 | DNA replication licensing factor MCM7 | MCM7 | 8 | 81.2 | 20 | -2.34 | -1.77 | 0.58 |
| 673 | P04040 | Catalase | CAT | 4 | 59.7 | 13 | -2.34 | -1.01 | 1.33 |
| 674 | A0A087X0R7 | SENP3-EIF4A1 readthrough (NMD candidate) | SENP3-EIF4A1 | 2 | 61.2 | 11 | -2.33 | -2.33 | 0 |
| 675 | A0AVT1 | Ubiquitin-like modifier-activating enzyme 6 | UBA6 | 12 | 117.9 | 24 | -2.32 | -2.45 | -0.13 |
| 676 | B4DXW1 | Actin-related protein 3 | ACTR3 | 3 | 42 | 17 | -2.32 | -1.89 | 0.43 |

**Supplementary Table S1:** List of all identified deregulated proteins (up-regulated and down-regulated) above -5-fold in sodium arsenate-exposed human NPCs by the high-resolution mass spectrometry (HRMS).
