# Supplementary material for: A protein–miRNA biomic analysis approach to explore neuroprotective potential of nobiletin in human neural progenitor cells (hNPCs)
Source: Front Pharmacol. 2024 Jan 25;15:1343569. doi: 10.3389/fphar.2024.1343569 (PMC10860404; doi:10.3389/fphar.2024.1343569)
Supplement: Supplementary file 2 [file Table8.DOCX]

**Supplementary Table S8**

**A. Mitochondrial and Oxidative Stress**

| **S. No** | **Accession** | **Description** | **Gene symbol** | **Abundance Ratio (log_2_): (NA) / (CON)** | **Abundance Ratio (log_2_): (NA_NOB) / (NA)** |
| --- | --- | --- | --- | --- | --- |
|  | A0A087X2D5 | 39S ribosomal protein L45, mitochondrial | MRPL45 | -6.13 | -0.94 |
|  | I3NI32 | Dihydroorotate dehydrogenase (quinone), mitochondrial | DHODH | -5.86 | 1.24 |
|  | A0A096LNH5 | Glutamine amidotransferase-like class 1 domain-containing protein 3B, mitochondrial | GATD3B | -5.18 | 0.13 |
|  | Q13268 | Dehydrogenase/reductase SDR family member 2, mitochondrial | DHRS2 | -4.94 | 2.77 |
|  | Q8IXI1 | Mitochondrial Rho GTPase 2 | RHOT2 | -4.94 | 2.28 |
|  | Q6IAL5 | Succinate--CoA ligase ADP/GDP-forming] subunit alpha, mitochondrial | SUCLG1 | -4.85 | 0.13 |
|  | P30048 | Thioredoxin-dependent peroxide reductase, mitochondrial | PRDX3 | -4.35 | 0.58 |
|  | Q10713 | Mitochondrial-processing peptidase subunit alpha | PMPCA | -3.95 | 0.56 |
|  | Q567R6 | Single-stranded DNA-binding protein, mitochondrial | SSBP1 | -3.91 | 1.08 |
|  | A0A0A0MS29 | Mitochondrial fission factor | MFF | -3.89 | 0.04 |
|  | Q16698 | 2,4-dienoyl-CoA reductase (3E)-enoyl-CoA-producing], mitochondrial | DECR1 | -3.57 | 0.55 |
|  | P30038 | Delta-1-pyrroline-5-carboxylate dehydrogenase, mitochondrial | ALDH4A1 | -3.53 | 0.17 |
|  | P11182 | Lipoamide acyltransferase component of branched-chain alpha-keto acid dehydrogenase complex, mitochondrial | DBT | -3.51 | 0.66 |
|  | P08559 | Pyruvate dehydrogenase E1 component subunit alpha, somatic form, mitochondrial | PDHA1 | -3.45 | 0.58 |
|  | H0YEW4 | ATP synthase mitochondrial F1 complex assembly factor 1 (Fragment) | ATPAF1 | -3.43 | 1.85 |
|  | E9PDQ8 | Succinate--CoA ligase GDP-forming] subunit beta, mitochondrial | SUCLG2 | -3.37 | 2.25 |
|  | Q99798 | Aconitate hydratase, mitochondrial | ACO2 | -3.34 | 0.72 |
|  | P13804 | Electron transfer flavoprotein subunit alpha, mitochondrial | ETFA | -3.28 | 0.57 |
|  | Q9Y5J9 | Mitochondrial import inner membrane translocase subunit Tim8 B | TIMM8B | -3.21 | 1.23 |
|  | Q6DKK2 | Tetratricopeptide repeat protein 19, mitochondrial | TTC19 | -3.19 | 1.26 |
|  | Q7RU05 | Mitochondrial import inner membrane translocase subunit TIM17 | TIM17A | -3.1 | 0.84 |
|  | E9PC15 | Acylglycerol kinase, mitochondrial | AGK | -3.09 | 0.53 |
|  | P23378 | Glycine dehydrogenase (decarboxylating), mitochondrial | GLDC | -3.05 | 0.17 |
|  | P07954 | Fumarate hydratase, mitochondrial | FH | -3.04 | 0.3 |
|  | Q99797 | Mitochondrial intermediate peptidase | MIPEP | -3.03 | 0.67 |
|  | B2RBJ8 | Glutamyl-tRNA(Gln) amidotransferase subunit A, mitochondrial | QRSL1 | -2.75 | 1.2 |
|  | P00390 | Glutathione reductase, mitochondrial | GSR | -2.76 | 0.12 |
|  | P51398 | 28S ribosomal protein S29, mitochondrial | DAP3 | -2.5 | 0.14 |
|  | A0A024RD08 | Mitochondrial carrier homolog 1 (C. elegans), isoform | MTCH1 | -2.46 | 1.13 |
|  | Q9UKU7 | Isobutyryl-CoA dehydrogenase, mitochondrial | ACAD8 | -2.46 | 0.92 |
|  | A0A024R850 | Mitochondrial ribosome recycling factor, isoform | MRRF | -2.45 | 1.32 |
|  | A0A0C4DGN7 | NADPH:adrenodoxin oxidoreductase, mitochondrial | FDXR | -2.41 | 0.19 |
|  | A0A087WW65 | ATP-binding cassette sub-family B member 7, mitochondrial | ABCB7 | -2.35 | 1.48 |
|  | Q96DI8 | Heme oxygenase | HMOX1 | 5.99 | -5.89 |
|  | Q5T440 | Putative transferase CAF17, mitochondrial | IBA57 | 3.55 | -1.25 |
|  | E7ESZ7 | NADH dehydrogenase [ubiquinone] 1 alpha subcomplex subunit 10, mitochondrial | NDUFA10 | 3.51 | -0.31 |
|  | D6REA0 | Glutamyl-tRNA(Gln) amidotransferase subunit B, mitochondrial | GATB | 2.62 | -0.04 |
|  | A0A384MDW7 | Enoyl Coenzyme A hydratase, short chain, 1, mitochondrial | ECHS1 | 2.52 | -0.21 |

**B. Ubiquitin proteasome system, autophagy, chaperons**

| **S. No.** | **Accession** | **Description** | **Gene Symbol** | **log_2_ Fold change: (NA) / (CON)** | **log_2_ Fold change: (NA_NOB) / (NA)** |
| --- | --- | --- | --- | --- | --- |
|  | K7EPJ5 | E3 ubiquitin-protein ligase MGRN1 | MGRN1 | -5.18 | 0.69 |
|  | Q8WVY7 | Ubiquitin-like domain-containing CTD phosphatase 1 | UBLCP1 | -3.94 | 1.68 |
|  | D3DUG9 | Ubiquitin carboxyl-terminal hydrolase | USP14 | -3.66 | 1.74 |
|  | Q8TF42 | ubiquitin-associated and SH3 domain-containing protein B | UBASH3B | -3.64 | 0.5 |
|  | A0A087WTW0 | RING-type E3 ubiquitin transferase | UHRF1 | -3.6 | -0.04 |
|  | P61088 | Ubiquitin-conjugating enzyme E2 N | UBE2N | -2.7 | 0.4 |
|  | Q9GZZ9 | Ubiquitin-like modifier-activating enzyme 5 | UBA5 | -2.59 | -1.4 |
|  | A0AVT1 | Ubiquitin-like modifier-activating enzyme 6 | UBA6 | -2.32 | -0.13 |
|  | A0A1W2PRF6 | Lysosome membrane protein 2 | SCARB2 | -2.73 | 1.48 |
|  | Q9ULT8 | E3 ubiquitin-protein ligase HECTD1 | HECTD1 | 4.28 | -1.15 |

**C. Neurodegeneration**

| **S. No.** | **Accession** | **Description** | **Gene Symbol** | **log2 Fold change: (NA) / (CON)** | **log2 Fold change: (NA_NOB) / (NA)** |
| --- | --- | --- | --- | --- | --- |
|  | P16615 | Sarcoplasmic/endoplasmic reticulum calcium ATPase 2 | ATP2A2 | -2.84 | 0.72 |
|  | P42574 | Caspase-3 | CASP3 | -2.83 | 0.9 |
|  | P04040 | Catalase | CAT | -2.34 | 1.33 |
|  | P20674 | Cytochrome c oxidase subunit 5A, mitochondrial | COX5A | -3.24 | -0.16 |
|  | P19784 | Casein kinase II subunit alpha | CSNK2A2 | -4.71 | -1.65 |
|  | A0A2R8Y7Z0 | Catenin beta-1 | CTNNB1 | -3.95 | -0.05 |
|  | E7EX90 | Dynactin subunit 1 | DCTN1 | -2.93 | 0.46 |
|  | O00399 | Dynactin subunit 6 | DCTN6 | -2.97 | 1.67 |
|  | Q8TBR3 | Fusion (Involved in t(1216) in malignant liposarcoma) | FUS | -3.04 | 0.3 |
|  | P11021 | Endoplasmic reticulum chaperone BiP | HSPA5 | -4.72 | 2.57 |
|  | D7P639 | NADH-ubiquinone oxidoreductase chain 5 | ND5 | -2.77 | 0.36 |
|  | A0A1L1ZH79 | NADH-ubiquinone oxidoreductase chain 5 | ND5 | -2.75 | 1.2 |
|  | B4DJ81 | NADH-ubiquinone oxidoreductase 75 kDa subunit, mitochondrial | NDUFS1 | -3.6 | 0.96 |
|  | Q4LE43 | Phosphoinositide phospholipase C (Fragment) | PLCG1 | -4.33 | 0.82 |
|  | P28066 | Proteasome subunit alpha type-5 | PSMA5 | -3.93 | 1.44 |
|  | P60900 | Proteasome subunit alpha type-6 | PSMA6 | -4.64 | 0.88 |
|  | P49720 | Proteasome subunit beta type-3 | PSMB3 | -4.11 | 1.06 |
|  | P28074 | Proteasome subunit beta type-5 | PSMB5 | -3.59 | 0.39 |
|  | P28072 | Proteasome subunit beta type-6 | PSMB6 | -4.85 | 0.66 |
|  | Q75L23 | 26S proteasome AAA-ATPase subunit RPT1 (Fragment) | PSMC2 | -2.59 | 1.14 |
|  | A0A087X2I1 | 26S proteasome regulatory subunit 10B | PSMC6 | -3.93 | 0.51 |
|  | O00231 | 26S proteasome non-ATPase regulatory subunit 11 | PSMD11 | -3.4 | 0.54 |
|  | H3BNT7 | 26S proteasome non-ATPase regulatory subunit 7 | PSMD7 | -4.21 | 1.1 |
|  | R4GMR5 | 26S proteasome non-ATPase regulatory subunit 8 | PSMD8 | -2.57 | 0.3 |
|  | E2QRB3 | Pyrroline-5-carboxylate reductase | PYCR1 | -3.97 | 0.88 |
|  | J3KR12 | Pyrroline-5-carboxylate reductase | PYCR1 | -2.98 | 0.19 |
|  | Q6NVC0 | SLC25A5 protein (Fragment) | SLC25A5 | -5.42 | 1.2 |
|  | P00441 | Superoxide dismutase [Cu-Zn] | SOD1 | -4.86 | -0.17 |
|  | Q13148 | TAR DNA-binding protein 43 | TARDBP | -2.41 | 1.58 |
|  | C9JQ00 | Tubulin alpha chain (Fragment) | TUBA4A | -2.7 | 1.99 |
|  | Q9BVA1 | Tubulin beta-2B chain | TUBB2B | -4.17 | 3.55 |
|  | Q9BUF5 | Tubulin beta-6 chain | TUBB6 | -2.72 | 0.94 |
|  | A0A384MDW7 | Enoyl Coenzyme A hydratase, short chain, 1, mitochondrial | ECHS1 | 2.52 | -0.21 |
|  | A0A0U1RRM8 | Fermitin family homolog 2 (Fragment) | FERMT2 | 2.33 | -1.2 |
|  | Q96DI8 | Heme oxygenase | HMOX1 | 5.99 | -5.89 |
|  | E7ESZ7 | NADH dehydrogenase [ubiquinone] 1 alpha subcomplex subunit 10, mitochondrial | NDUFA10 | 3.51 | -0.31 |
|  | A0A494C0D4 | Protoporphyrinogen oxidase (Fragment) | PPOX | 3.57 | -0.39 |
|  | Q5QPM7 | Proteasome inhibitor PI31 subunit | PSMF1 | 3.17 | -0.51 |

**D. Programmed Cell Death**

| **S. No.** | **Accession** | **Description** | **Gene Symbol** | **log_2_ Fold change: (NA) / (CON)** | **log_2_ Fold change: (NA_NOB) / (NA)** |
| --- | --- | --- | --- | --- | --- |
|  | S4R3H4 | Apoptotic chromatin condensation inducer in the nucleus | ACIN1 | -3.59 | -0.45 |
|  | Q07021 | Complement component 1 Q subcomponent-binding protein, mitochondrial | C1QBP | -3.64 | 1.36 |
|  | P42574 | Caspase-3 | CASP3 | -2.83 | 0.9 |
|  | F8WBG8 | Drebrin-like protein | DBNL | -2.47 | 0.29 |
|  | Q5T7C4 | High mobility group protein B1 | HMGB1 | -5.25 | 1.92 |
|  | A0A6Q8PFJ0 | Prelamin-A/C | LMNA | -3.45 | 0.99 |
|  | D7P639 | NADH-ubiquinone oxidoreductase chain 5 | ND5 | -2.77 | 0.36 |
|  | A0A1L1ZH79 | NADH-ubiquinone oxidoreductase chain 5 | ND5 | -2.75 | 1.2 |
|  | P28066 | Proteasome subunit alpha type-5 | PSMA5 | -3.93 | 1.44 |
|  | P60900 | Proteasome subunit alpha type-6 | PSMA6 | -4.64 | 0.88 |
|  | P49720 | Proteasome subunit beta type-3 | PSMB3 | -4.11 | 1.06 |
|  | P28074 | Proteasome subunit beta type-5 | PSMB5 | -3.59 | 0.39 |
|  | P28072 | Proteasome subunit beta type-6 | PSMB6 | -4.85 | 0.66 |
|  | Q75L23 | 26S proteasome AAA-ATPase subunit RPT1 (Fragment) | PSMC2 | -2.59 | 1.14 |
|  | A0A087X2I1 | 26S proteasome regulatory subunit 10B | PSMC6 | -3.93 | 0.51 |
|  | B1AJY5 | 26S proteasome non-ATPase regulatory subunit 10 | PSMD10 | -3.78 | 0.69 |
|  | O00231 | 26S proteasome non-ATPase regulatory subunit 11 | PSMD11 | -3.4 | 0.54 |
|  | H3BNT7 | 26S proteasome non-ATPase regulatory subunit 7 | PSMD7 | -4.21 | 1.1 |
|  | R4GMR5 | 26S proteasome non-ATPase regulatory subunit 8 | PSMD8 | -2.57 | 0.3 |
|  | Q6FHU3 | PSME1 protein (Fragment) | PSME1 | -2.58 | 0.4 |
|  | E2QRB3 | Pyrroline-5-carboxylate reductase | PYCR1 | -3.97 | 0.88 |
|  | J3KR12 | Pyrroline-5-carboxylate reductase | PYCR1 | -2.98 | 0.19 |
|  | E5RI99 | 60S ribosomal protein L30 (Fragment) | RPL30 | -2.58 | 0.95 |
|  | A0A087X0K9 | Tight junction protein ZO-1 | TJP1 | -3.14 | -0.48 |
|  | A0A0A6YYA0 | Protein TMED7-TICAM2 | TMED7-TICAM2 | -3.33 | 0.43 |
|  | Q96DI8 | Heme oxygenase | HMOX1 | 5.99 | -5.89 |
|  | J3KRA9 | Non-specific serine/threonine protein kinase (Fragment) | SMG1 | 2.85 | -1.61 |

**Supplementary Table S8:** List of comparative analysis showing the significant alteration in the protein deregulation pattern in different pathways following the exposure of NPCs to NA and reversal effect of NOB on NA exposure.
