# Supplementary material for: A protein–miRNA biomic analysis approach to explore neuroprotective potential of nobiletin in human neural progenitor cells (hNPCs)
Source: Front Pharmacol. 2024 Jan 25;15:1343569. doi: 10.3389/fphar.2024.1343569 (PMC10860404; doi:10.3389/fphar.2024.1343569)
Supplement: Supplementary file 3 [file Table7.DOCX]

**Supplementary Table S7**

1. **Up-regulated Proteins**

| **S.No.** | **Reactome term** | **Count** | **Reactome identifier** | **P-value** | **Proteins (Up-regulated)** |
| --- | --- | --- | --- | --- | --- |
| 1 | tRNA processing in the nucleus | 5 | R-HSA-6784531 | 6.98E-05 | NUP205, NUP107, NUP188, NUP210, XPOT |
| 2 | Metabolism | 20 | R-HSA-1430728 | 1.73E-04 | GSTM3, PDXK, NUP205, NUP107, ECHS1, MVK, NUP188, NUP210, GBE1, AGL, NDUFA10, PYGL, WASL, PPOX, THTPA, GNAQ, HMOX1, PSMF1, NUDT16, AASDHPPT |
| 3 | Regulation of Glucokinase-by-Glucokinase Regulatory Protein | 4 | R-HSA-170822 | 2.34E-04 | NUP205, NUP107, NUP188, NUP210 |
| 4 | Transport of Ribonucleoproteins into the Host Nucleus | 4 | R-HSA-168271 | 2.34E-04 | NUP205, NUP107, NUP188, NUP210 |
| 5 | Defective TPR may confer susceptibility towards thyroid papillary carcinoma (TPC) | 4 | R-HSA-5619107 | 2.34E-04 | NUP205, NUP107, NUP188, NUP210 |
| 6 | NEP/NS2 Interacts with the Cellular Export Machinery | 4 | R-HSA-168333 | 2.34E-04 | NUP205, NUP107, NUP188, NUP210 |
| 7 | Nuclear import of Rev protein | 4 | R-HSA-180746 | 2.83E-04 | NUP205, NUP107, NUP188, NUP210 |
| 8 | Vpr-mediated nuclear import of PICs | 4 | R-HSA-180910 | 2.83E-04 | NUP205, NUP107, NUP188, NUP210 |
| 9 | Export of Viral Ribonucleoproteins from Nucleus | 4 | R-HSA-168274 | 2.83E-04 | NUP205, NUP107, NUP188, NUP210 |
| 10 | Rev-mediated nuclear export of HIV RNA | 4 | R-HSA-165054 | 3.10E-04 | NUP205, NUP107, NUP188, NUP210 |
| 11 | Transport of the SLBP independent Mature mRNA | 4 | R-HSA-159227 | 3.10E-04 | NUP205, NUP107, NUP188, NUP210 |
| 12 | SUMOylation of SUMOylation proteins | 4 | R-HSA-4085377 | 3.10E-04 | NUP205, NUP107, NUP188, NUP210 |
| 13 | Transport of the SLBP Dependant Mature mRNA | 4 | R-HSA-159230 | 3.38E-04 | NUP205, NUP107, NUP188, NUP210 |
| 14 | Nuclear Pore Complex (NPC) Disassembly | 4 | R-HSA-3301854 | 3.38E-04 | NUP205, NUP107, NUP188, NUP210 |
| 15 | Interactions of Vpr with host cellular proteins | 4 | R-HSA-176033 | 3.67E-04 | NUP205, NUP107, NUP188, NUP210 |
| 16 | Interactions of Rev with host cellular proteins | 4 | R-HSA-177243 | 3.67E-04 | NUP205, NUP107, NUP188, NUP210 |
| 17 | SUMOylation of ubiquitinylation proteins | 4 | R-HSA-3232142 | 4.32E-04 | NUP205, NUP107, NUP188, NUP210 |
| 18 | NS1 Mediated Effects on Host Pathways | 4 | R-HSA-168276 | 5.03E-04 | NUP205, NUP107, NUP188, NUP210 |
| 19 | Transport of Mature mRNA Derived from an Intronless Transcript | 4 | R-HSA-159231 | 5.41E-04 | NUP205, NUP107, NUP188, NUP210 |
| 20 | Transport of Mature mRNAs Derived from Intronless Transcripts | 4 | R-HSA-159234 | 5.81E-04 | NUP205, NUP107, NUP188, NUP210 |
| 21 | Viral Messenger RNA Synthesis | 4 | R-HSA-168325 | 6.23E-04 | NUP205, NUP107, NUP188, NUP210 |
| 22 | SUMOylation of DNA replication proteins | 4 | R-HSA-4615885 | 7.12E-04 | NUP205, NUP107, NUP188, NUP210 |
| 23 | tRNA processing | 5 | R-HSA-72306 | 7.91E-04 | NUP205, NUP107, NUP188, NUP210, XPOT |
| 24 | SUMOylation of RNA binding proteins | 4 | R-HSA-4570464 | 8.09E-04 | NUP205, NUP107, NUP188, NUP210 |
| 25 | Metabolism of carbohydrates | 7 | R-HSA-71387 | 9.88E-04 | NUP205, NUP107, NUP188, NUP210, AGL, GBE1, PYGL |
| 26 | Nuclear Envelope Breakdown | 4 | R-HSA-2980766 | 1.09E-03 | NUP205, NUP107, NUP188, NUP210 |
| 27 | snRNP Assembly | 4 | R-HSA-191859 | 1.15E-03 | NUP205, NUP107, NUP188, NUP210 |
| 28 | Metabolism of non-coding RNA | 4 | R-HSA-194441 | 1.15E-03 | NUP205, NUP107, NUP188, NUP210 |
| 29 | Host Interactions of HIV factors | 5 | R-HSA-162909 | 1.63E-03 | NUP205, NUP107, NUP188, NUP210, PSMF1 |
| 30 | Regulation of HSF1-mediated heat shock response | 4 | R-HSA-3371453 | 2.46E-03 | NUP205, NUP107, NUP188, NUP210 |
| 31 | SUMOylation of chromatin organization proteins | 4 | R-HSA-4551638 | 2.67E-03 | NUP205, NUP107, NUP188, NUP210 |
| 32 | ISG15 antiviral mechanism | 4 | R-HSA-1169408 | 2.78E-03 | NUP205, NUP107, NUP188, NUP210 |
| 33 | Glycolysis | 4 | R-HSA-70171 | 2.89E-03 | NUP205, NUP107, NUP188, NUP210 |
| 34 | Transport of Mature mRNA derived from an Intron-Containing Transcript | 4 | R-HSA-159236 | 3.12E-03 | NUP205, NUP107, NUP188, NUP210 |
| 35 | SUMOylation of DNA damage response and repair proteins | 4 | R-HSA-3108214 | 3.37E-03 | NUP205, NUP107, NUP188, NUP210 |
| 36 | Antiviral mechanism by IFN-stimulated genes | 4 | R-HSA-1169410 | 3.75E-03 | NUP205, NUP107, NUP188, NUP210 |
| 37 | Transport of Mature Transcript to Cytoplasm | 4 | R-HSA-72202 | 4.30E-03 | NUP205, NUP107, NUP188, NUP210 |
| 38 | Disorders of transmembrane transporters | 5 | R-HSA-5619115 | 4.87E-03 | NUP205, NUP107, NUP188, NUP210, PSMF1 |
| 39 | Glycogen metabolism | 3 | R-HSA-8982491 | 4.96E-03 | AGL, GBE1, PYGL |
| 40 | Postmitotic nuclear pore complex (NPC) reformation | 3 | R-HSA-9615933 | 4.96E-03 | NUP205, NUP107, NUP188 |
| 41 | Cellular response to heat stress | 4 | R-HSA-3371556 | 5.06E-03 | NUP205, NUP107, NUP188, NUP210 |
| 42 | Glucose metabolism | 4 | R-HSA-70326 | 5.72E-03 | NUP205, NUP107, NUP188, NUP210 |
| 43 | Metabolism of RNA | 9 | R-HSA-8953854 | 6.02E-03 | LSM8, SMG1, NUP205, NUP107, NUP188, NUP210, XPOT, TNKS1BP1, PSMF1 |
| 44 | SLC transporter disorders | 4 | R-HSA-5619102 | 6.61E-03 | NUP205, NUP107, NUP188, NUP210 |
| 45 | Transcriptional regulation by small RNAs | 4 | R-HSA-5578749 | 7.99E-03 | NUP205, NUP107, NUP188, NUP210 |
| 46 | HCMV Late Events | 4 | R-HSA-9610379 | 1.05E-02 | NUP205, NUP107, NUP188, NUP210 |
| 47 | HIV Infection | 5 | R-HSA-162906 | 1.25E-02 | NUP205, NUP107, NUP188, NUP210, PSMF1 |
| 48 | SARS-CoV-2 activates/modulates innate and adaptive immune responses | 4 | R-HSA-9705671 | 1.31E-02 | NUP205, NUP107, NUP188, NUP210 |
| 49 | HCMV Early Events | 4 | R-HSA-9609690 | 1.58E-02 | NUP205, NUP107, NUP188, NUP210 |
| 50 | Influenza Viral RNA Transcription and Replication | 4 | R-HSA-168273 | 1.61E-02 | NUP205, NUP107, NUP188, NUP210 |
| 51 | Late Phase of HIV Life Cycle | 4 | R-HSA-162599 | 1.61E-02 | NUP205, NUP107, NUP188, NUP210 |
| 52 | Gene Silencing by RNA | 4 | R-HSA-211000 | 1.64E-02 | NUP205, NUP107, NUP188, NUP210 |
| 53 | Mitotic Prophase | 4 | R-HSA-68875 | 1.77E-02 | NUP205, NUP107, NUP188, NUP210 |
| 54 | HIV Life Cycle | 4 | R-HSA-162587 | 2.05E-02 | NUP205, NUP107, NUP188, NUP210 |
| 55 | Signaling by Rho GTPases, Miro GTPases and RHOBTB3 | 8 | R-HSA-9716542 | 2.11E-02 | DIAPH1, GIT2, NUP107, KIDINS220, MFN2, MYO9B, WASL, FERMT2 |
| 56 | Influenza Infection | 4 | R-HSA-168255 | 2.34E-02 | NUP205, NUP107, NUP188, NUP210 |
| 57 | HCMV Infection | 4 | R-HSA-9609646 | 2.46E-02 | NUP205, NUP107, NUP188, NUP210 |
| 58 | Processing of Capped Intron-Containing Pre-mRNA | 5 | R-HSA-72203 | 2.50E-02 | LSM8, NUP205, NUP107, NUP188, NUP210 |
| 59 | RHO GTPase cycle | 6 | R-HSA-9012999 | 3.02E-02 | DIAPH1, GIT2, KIDINS220, MYO9B, WASL, FERMT2 |
| 60 | Infectious disease | 9 | R-HSA-5663205 | 3.11E-02 | NUP205, NUP107, FNTA, NUP188, NUP210, HMOX1, MYO9B, WASL, PSMF1 |
| 61 | SUMO E3 ligases SUMOylate target proteins | 4 | R-HSA-3108232 | 3.42E-02 | NUP205, NUP107, NUP188, NUP210 |
| 62 | Nuclear Envelope (NE) Reassembly | 3 | R-HSA-2995410 | 3.48E-02 | NUP205, NUP107, NUP188 |
| 63 | RAC1 GTPase cycle | 4 | R-HSA-9013149 | 3.57E-02 | GIT2, MYO9B, WASL, FERMT2 |
| 64 | SUMOylation | 4 | R-HSA-2990846 | 3.72E-02 | NUP205, NUP107, NUP188, NUP210 |
| 65 | Immune System | 14 | R-HSA-168256 | 4.24E-02 | PDXK, NUP205, NUP107, NUP188, NUP210, AGL, SURF4, MYO9B, PYGL, WASL, DIAPH1, HECTD1, HMOX1, PSMF1 |
| 66 | Interferon Signaling | 4 | R-HSA-913531 | 4.50E-02 | NUP205, NUP107, NUP188, NUP210 |

**B. Down-regulated Proteins**

| **S.No.** | **Reactome term** | **Count** | **Reactome identifier** | **P-value** | **Proteins (Down-regulated)** |
| --- | --- | --- | --- | --- | --- |
| 1 | Metabolism of RNA | 95 | R-HSA-8953854 | 2.82E-27 | EIF4A1, RPL5, RPL30, RBM25, POP1, RPLP1, RTCB, DDX42, RPL10A, CCAR1, RPS15, PSMD8, PSMD7, PSMD4, RPLP2, UTP14A, UTP15, SRRM2, RPL23, ELAC2, RPSA, GTF2F2, SRRM1, GNL3, PSMA5, PSMA6, SKIC8, CLNS1A, SARNP, PSME1, NHP2, SRSF3, PPIG, PSMD10, SF3B2, PSMD11, ANP32A, RBM8A, PSMD13, SF3B6, NIP7, RPL12, SRRT, NOP2, PSMA7, PSMB6, EXOSC6, NXF1, PSMB4, EXOSC10, PSMB5, PUF60, FIP1L1, POLR2B, PSMB3, PPP1R8, PCBP2, SAP18, IGF2BP3, UTP20, SNRPB2, IGF2BP2, RPS2, HNRNPA1, SF3B1, RAE1, EXOSC2, RPL17, RANBP2, HNRNPA3, CPSF7, FUS, WDR18, ALYREF, YJU2, LSM1, NUP153, U2SURP, HNRNPL, BOP1, RPS28, PSMC6, EPRS1, ERCC3, LAS1L, PSMC4, HNRNPF, PSMC2, HNRNPD, TSR1, ACIN1, RNPS1, HNRNPC, RPS21, NUP37 |
| 2 | Regulation of expression of SLITs and ROBOs | 32 | R-HSA-9010553 | 4.76E-13 | PSMD10, RPL5, RPL30, PSMD11, RBM8A, PSMD13, RPLP1, RPL12, RPL10A, PSMA7, RPS15, PSMD8, PSMB6, PSMB4, PSMD7, PSMB5, PSMD4, PSMB3, RPLP2, RPS2, RPL17, RPL23, RPSA, PSMA5, PSMA6, RPS28, PSMC6, PSMC4, PSMC2, PSME1, RNPS1, RPS21 |
| 3 | Metabolism of amino acids and derivatives | 47 | R-HSA-71291 | 2.55E-12 | PSMD10, RPL5, RPL30, PSMD11, PSMD13, GLDC, RPLP1, RPL12, PDHB, RPL10A, PSMA7, RPS15, PSMD8, PSMB6, PSMB4, PSMD7, PSMB5, PSMD4, PSMB3, DBT, RPLP2, RPS2, ENOPH1, RPL17, MPST, ACAD8, PDHA1, RPL23, GSR, PYCR1, RPSA, FAH, ALDH4A1, AIMP1, PSMA5, GRHPR, PSMA6, RPS28, PSMC6, EPRS1, PSMC4, IVD, PSMC2, SMS, PSME1, SCLY, RPS21 |
| 4 | Host Interactions of HIV factors | 27 | R-HSA-162909 | 3.21E-12 | PSMD10, PSMD11, PSMD13, PSIP1, PSMA7, PSMD8, PSMB6, PSMB4, PSMD7, PSMB5, PSMD4, PSMB3, RCC1, RAE1, AP2M1, SKP1, RANBP2, NPM1, NUP153, PSMA5, PSMA6, PSMC6, PSMC4, PSMC2, PSME1, SLC25A5, NUP37 |
| 5 | GSK3B and BTRC: CUL1-mediated-degradation of NFE2L2 | 18 | R-HSA-9762114 | 4.33E-12 | PSMD10, PSMD11, PSMD13, PSMA7, PSMD8, PSMA5, PSMB6, PSMA6, PSMB4, PSMC6, PSMD7, PSMB5, PSMD4, PSMC4, PSMB3, PSMC2, PSME1, SKP1 |
| 6 | Vpu mediated degradation of CD4 | 18 | R-HSA-180534 | 4.33E-12 | PSMD10, PSMD11, PSMD13, PSMA7, PSMD8, PSMA5, PSMB6, PSMA6, PSMB4, PSMC6, PSMD7, PSMB5, PSMD4, PSMC4, PSMB3, PSMC2, PSME1, SKP1 |
| 7 | Somitogenesis | 18 | R-HSA-9824272 | 6.15E-12 | PSMD10, PSMD11, PSMD13, PSMA7, PSMD8, PSMA5, PSMB6, PSMA6, PSMB4, PSMC6, PSMD7, PSMB5, PSMD4, PSMC4, PSMB3, PSMC2, PSME1, CTNNB1 |
| 8 | The role of GTSE1 in G2/M progression after G2 checkpoint | 21 | R-HSA-8852276 | 6.70E-12 | PSMD10, PSMD11, PSMD13, TUBA4A, PSMA7, PSMD8, PSMA5, PSMB6, PSMA6, TUBB6, TUBB2B, PSMB4, PSMC6, PSMD7, PSMB5, PSMD4, PSMC4, PSMB3, PSMC2, PSME1, MAPRE1 |
| 9 | FBXL7 down-regulates AURKA during mitotic entry and in early mitosis | 18 | R-HSA-8854050 | 8.66E-12 | PSMD10, PSMD11, PSMD13, PSMA7, PSMD8, PSMA5, PSMB6, PSMA6, PSMB4, PSMC6, PSMD7, PSMB5, PSMD4, PSMC4, PSMB3, PSMC2, PSME1, SKP1 |
| 10 | Negative regulation of NOTCH4 signaling | 18 | R-HSA-9604323 | 8.66E-12 | PSMD10, PSMD11, PSMD13, PSMA7, PSMD8, PSMA5, PSMB6, PSMA6, PSMB4, PSMC6, PSMD7, PSMB5, PSMD4, PSMC4, PSMB3, PSMC2, PSME1, SKP1 |
| 11 | SCF-beta-TrCP mediated degradation of Emi1 | 18 | R-HSA-174113 | 1.21E-11 | PSMD10, PSMD11, PSMD13, PSMA7, PSMD8, PSMA5, PSMB6, PSMA6, PSMB4, PSMC6, PSMD7, PSMB5, PSMD4, PSMC4, PSMB3, PSMC2, PSME1, SKP1 |
| 12 | Orc1 removal from chromatin | 20 | R-HSA-68949 | 1.28E-11 | PSMD10, PSMD11, MCM7, PSMD13, PSMA7, PSMD8, PSMA5, PSMB6, PSMA6, PSMB4, PSMC6, PSMD7, PSMB5, PSMD4, PSMC4, PSMB3, PSMC2, PSME1, MCM6, SKP1 |
| 13 | AUF1 (hnRNP D0) binds and destabilizes mRNA | 18 | R-HSA-450408 | 1.68E-11 | PSMD10, PSMD11, PSMD13, PSMA7, PSMD8, PSMA5, PSMB6, PSMA6, PSMB4, PSMC6, PSMD7, PSMB5, PSMD4, PSMC4, PSMB3, PSMC2, HNRNPD, PSME1 |
| 14 | Processing of Capped Intron-Containing Pre-mRNA | 39 | R-HSA-72203 | 1.74E-11 | RBM25, SF3B2, RBM8A, SF3B6, SRRT, DDX42, CCAR1, NXF1, FIP1L1, PUF60, POLR2B, PPP1R8, PCBP2, SAP18, SNRPB2, HNRNPA1, SF3B1, RAE1, RANBP2, SRRM2, CPSF7, HNRNPA3, FUS, ALYREF, YJU2, NUP153, GTF2F2, U2SURP, SRRM1, HNRNPL, SARNP, HNRNPF, HNRNPD, ACIN1, SRSF3, RNPS1, PPIG, HNRNPC, NUP37 |
| 15 | Cross-presentation of soluble exogenous antigens (endosomes) | 17 | R-HSA-1236978 | 1.91E-11 | PSMD10, PSMD11, PSMD13, PSMA7, PSMD8, PSMA5, PSMB6, PSMA6, PSMB4, PSMC6, PSMD7, PSMB5, PSMD4, PSMC4, PSMB3, PSMC2, PSME1 |
| 16 | Regulation of activated PAK-2p34 by proteasome mediated degradation | 17 | R-HSA-211733 | 2.71E-11 | PSMD10, PSMD11, PSMD13, PSMA7, PSMD8, PSMA5, PSMB6, PSMA6, PSMB4, PSMC6, PSMD7, PSMB5, PSMD4, PSMC4, PSMB3, PSMC2, PSME1 |
| 17 | Regulation of ornithine decarboxylase (ODC) | 17 | R-HSA-350562 | 3.81E-11 | PSMD10, PSMD11, PSMD13, PSMA7, PSMD8, PSMA5, PSMB6, PSMA6, PSMB4, PSMC6, PSMD7, PSMB5, PSMD4, PSMC4, PSMB3, PSMC2, PSME1 |
| 18 | Metabolism of polyamines | 18 | R-HSA-351202 | 4.26E-11 | PSMD10, PSMD11, PSMD13, PSMA7, PSMD8, PSMA5, PSMB6, PSMA6, PSMB4, PSMC6, PSMD7, PSMB5, PSMD4, PSMC4, PSMB3, PSMC2, SMS, PSME1 |
| 19 | NIK-->noncanonical NF-kB signaling | 18 | R-HSA-5676590 | 4.26E-11 | PSMD10, PSMD11, PSMD13, PSMA7, PSMD8, PSMA5, PSMB6, PSMA6, PSMB4, PSMC6, PSMD7, PSMB5, PSMD4, PSMC4, PSMB3, PSMC2, PSME1, SKP1 |
| 20 | Autodegradation of the E3 ubiquitin ligase COP1 | 17 | R-HSA-349425 | 5.31E-11 | PSMD10, PSMD11, PSMD13, PSMA7, PSMD8, PSMA5, PSMB6, PSMA6, PSMB4, PSMC6, PSMD7, PSMB5, PSMD4, PSMC4, PSMB3, PSMC2, PSME1 |
| 21 | Ubiquitin Mediated Degradation of Phosphorylated Cdc25A | 17 | R-HSA-69601 | 5.31E-11 | PSMD10, PSMD11, PSMD13, PSMA7, PSMD8, PSMA5, PSMB6, PSMA6, PSMB4, PSMC6, PSMD7, PSMB5, PSMD4, PSMC4, PSMB3, PSMC2, PSME1 |
| 22 | p53-Independent DNA Damage Response | 17 | R-HSA-69610 | 5.31E-11 | PSMD10, PSMD11, PSMD13, PSMA7, PSMD8, PSMA5, PSMB6, PSMA6, PSMB4, PSMC6, PSMD7, PSMB5, PSMD4, PSMC4, PSMB3, PSMC2, PSME1 |
| 23 | p53-Independent G1/S DNA damage checkpoint | 17 | R-HSA-69613 | 5.31E-11 | PSMD10, PSMD11, PSMD13, PSMA7, PSMD8, PSMA5, PSMB6, PSMA6, PSMB4, PSMC6, PSMD7, PSMB5, PSMD4, PSMC4, PSMB3, PSMC2, PSME1 |
| 24 | Ubiquitin-dependent degradation of Cyclin D | 17 | R-HSA-75815 | 5.31E-11 | PSMD10, PSMD11, PSMD13, PSMA7, PSMD8, PSMA5, PSMB6, PSMA6, PSMB4, PSMC6, PSMD7, PSMB5, PSMD4, PSMC4, PSMB3, PSMC2, PSME1 |
| 25 | Degradation of GLI2 by the proteasome | 18 | R-HSA-5610783 | 5.74E-11 | PSMD10, PSMD11, PSMD13, PSMA7, PSMD8, PSMA5, PSMB6, PSMA6, PSMB4, PSMC6, PSMD7, PSMB5, PSMD4, PSMC4, PSMB3, PSMC2, PSME1, SKP1 |
| 26 | Dectin-1 mediated noncanonical NF-kB signaling | 18 | R-HSA-5607761 | 5.74E-11 | PSMD10, PSMD11, PSMD13, PSMA7, PSMD8, PSMA5, PSMB6, PSMA6, PSMB4, PSMC6, PSMD7, PSMB5, PSMD4, PSMC4, PSMB3, PSMC2, PSME1, SKP1 |
| 27 | SCF(Skp2)-mediated degradation of p27/p21 | 18 | R-HSA-187577 | 5.74E-11 | PSMD10, PSMD11, PSMD13, PSMA7, PSMD8, PSMA5, PSMB6, PSMA6, PSMB4, PSMC6, PSMD7, PSMB5, PSMD4, PSMC4, PSMB3, PSMC2, PSME1, SKP1 |
| 28 | Degradation of GLI1 by the proteasome | 18 | R-HSA-5610780 | 5.74E-11 | PSMD10, PSMD11, PSMD13, PSMA7, PSMD8, PSMA5, PSMB6, PSMA6, PSMB4, PSMC6, PSMD7, PSMB5, PSMD4, PSMC4, PSMB3, PSMC2, PSME1, SKP1 |
| 29 | GLI3 is processed to GLI3R by the proteasome | 18 | R-HSA-5610785 | 5.74E-11 | PSMD10, PSMD11, PSMD13, PSMA7, PSMD8, PSMA5, PSMB6, PSMA6, PSMB4, PSMC6, PSMD7, PSMB5, PSMD4, PSMC4, PSMB3, PSMC2, PSME1, SKP1 |
| 30 | HIV Infection | 34 | R-HSA-162906 | 6.21E-11 | PSMD10, GTF2A1, FEN1, PSMD11, PSMD13, PSIP1, PSMA7, PSMD8, PSMB6, PSMB4, PSMD7, PSMB5, PSMD4, POLR2B, PSMB3, RCC1, NELFB, RAE1, AP2M1, SKP1, RANBP2, NPM1, NUP153, GTF2F2, PSMA5, PSMA6, PSMC6, ERCC3, PSMC4, PSMC2, PSME1, CHMP4B, SLC25A5, NUP37 |
| 31 | Regulation of Apoptosis | 17 | R-HSA-169911 | 7.34E-11 | PSMD10, PSMD11, PSMD13, PSMA7, PSMD8, PSMA5, PSMB6, PSMA6, PSMB4, PSMC6, PSMD7, PSMB5, PSMD4, PSMC4, PSMB3, PSMC2, PSME1 |
| 32 | Regulation of mRNA stability by proteins that bind AU-rich elements | 21 | R-HSA-450531 | 9.44E-11 | PSMD10, PSMD11, ANP32A, PSMD13, PSMA7, PSMD8, PSMA5, PSMB6, PSMA6, EXOSC6, PSMB4, PSMC6, PSMD7, PSMB5, PSMD4, PSMC4, PSMB3, PSMC2, HNRNPD, PSME1, EXOSC2 |
| 33 | Nuclear events mediated by NFE2L2 | 20 | R-HSA-9759194 | 9.79E-11 | PSMD10, PSMD11, PSMD13, IDH1, GSR, PSMA7, PSMD8, PSMA5, PSMB6, PSMA6, PSMB4, PSMC6, PSMD7, PSMB5, PSMD4, PSMC4, PSMB3, PSMC2, PSME1, SKP1 |
| 34 | Vif-mediated degradation of APOBEC3G | 17 | R-HSA-180585 | 1.01E-10 | PSMD10, PSMD11, PSMD13, PSMA7, PSMD8, PSMA5, PSMB6, PSMA6, PSMB4, PSMC6, PSMD7, PSMB5, PSMD4, PSMC4, PSMB3, PSMC2, PSME1 |
| 35 | Mitotic Anaphase | 34 | R-HSA-68882 | 1.10E-10 | PSMD10, PSMD11, PSMD13, PSMA7, LMNB1, PSMD8, PSMB6, TUBB6, PSMB4, PSMD7, PSMB5, PSMD4, PSMB3, LMNA, RCC1, BUB3, LBR, EMD, RANBP2, SMC1A, CKAP5, TUBA4A, PSMA5, PSMA6, TUBB2B, CLIP1, PSMC6, PSMC4, PSMC2, PSME1, CHMP4B, MAPRE1, ANAPC1, NUP37 |
| 36 | Mitotic Metaphase and Anaphase | 34 | R-HSA-2555396 | 1.25E-10 | PSMD10, PSMD11, PSMD13, PSMA7, LMNB1, PSMD8, PSMB6, TUBB6, PSMB4, PSMD7, PSMB5, PSMD4, PSMB3, LMNA, RCC1, BUB3, LBR, EMD, RANBP2, SMC1A, CKAP5, TUBA4A, PSMA5, PSMA6, TUBB2B, CLIP1, PSMC6, PSMC4, PSMC2, PSME1, CHMP4B, MAPRE1, ANAPC1, NUP37 |
| 37 | Degradation of AXIN | 17 | R-HSA-4641257 | 1.37E-10 | PSMD10, PSMD11, PSMD13, PSMA7, PSMD8, PSMA5, PSMB6, PSMA6, PSMB4, PSMC6, PSMD7, PSMB5, PSMD4, PSMC4, PSMB3, PSMC2, PSME1 |
| 38 | Regulation of RUNX3 expression and activity | 17 | R-HSA-8941858 | 1.37E-10 | PSMD10, PSMD11, PSMD13, PSMA7, PSMD8, PSMA5, PSMB6, PSMA6, PSMB4, PSMC6, PSMD7, PSMB5, PSMD4, PSMC4, PSMB3, PSMC2, PSME1 |
| 39 | Viral Infection Pathways | 68 | R-HSA-9824446 | 1.49E-10 | ITGB1, RPL5, RPL30, FEN1, RPLP1, PSIP1, RPL10A, RPS15, PSMD8, TUBB6, PSMD7, TRIM28, PSMD4, RPLP2, RCC1, NELFB, AP2M1, SKP1, RPL23, RPSA, ATP1B3, GTF2F2, TUBA4A, DDOST, PSMA5, PSMA6, TUBB2B, TBL1XR1, PSME1, CHMP4B, PPIG, SLC25A5, PSMD10, GTF2A1, PSMD11, ROCK2, PSMD13, RPL12, RPN1, ATP1A1, PSMA7, PSMB6, PSMB4, TKFC, PSMB5, LARP1, RBBP4, POLR2B, PSMB3, PCBP2, SAP18, RPS2, HNRNPA1, RAE1, RPL17, RANBP2, NPM1, NUP153, TJP1, RPS28, PSMC6, ERCC3, PSMC4, IMPDH2, PSMC2, UBE2N, RPS21, NUP37 |
| 40 | Regulation of APC/C activators between G1/S and early anaphase | 20 | R-HSA-176408 | 1.56E-10 | PSMD10, PSMD11, PSMD13, PSMA7, PSMD8, PSMA5, PSMB6, PSMA6, PSMB4, PSMC6, PSMD7, PSMB5, PSMD4, PSMC4, PSMB3, PSMC2, PSME1, BUB3, ANAPC1, SKP1 |
| 41 | Synthesis of DNA | 24 | R-HSA-69239 | 1.74E-10 | PSMD10, FEN1, PSMD11, MCM7, PSMD13, PSMA7, PSMD8, PSMA5, PSMB6, POLA1, PSMA6, PSMB4, PSMC6, PSMD7, PSMB5, PSMD4, PSMC4, PSMB3, PSMC2, POLD2, PSME1, MCM6, ANAPC1, SKP1 |
| 42 | Autodegradation of Cdh1 by Cdh1:APC/C | 18 | R-HSA-174084 | 1.77E-10 | PSMD10, PSMD11, PSMD13, PSMA7, PSMD8, PSMA5, PSMB6, PSMA6, PSMB4, PSMC6, PSMD7, PSMB5, PSMD4, PSMC4, PSMB3, PSMC2, PSME1, ANAPC1 |
| 43 | Hh mutants are degraded by ERAD | 17 | R-HSA-5362768 | 1.85E-10 | PSMD10, PSMD11, PSMD13, PSMA7, PSMD8, PSMA5, PSMB6, PSMA6, PSMB4, PSMC6, PSMD7, PSMB5, PSMD4, PSMC4, PSMB3, PSMC2, PSME1 |
| 44 | Cdc20:Phospho-APC/C mediated degradation of Cyclin A | 19 | R-HSA-174184 | 1.97E-10 | PSMD10, PSMD11, PSMD13, PSMA7, PSMD8, PSMA5, PSMB6, PSMA6, PSMB4, PSMC6, PSMD7, PSMB5, PSMD4, PSMC4, PSMB3, PSMC2, PSME1, BUB3, ANAPC1 |
| 45 | Switching of origins to a post-replicative state | 21 | R-HSA-69052 | 2.22E-10 | PSMD10, PSMD11, MCM7, PSMD13, PSMA7, PSMD8, PSMA5, PSMB6, PSMA6, PSMB4, PSMC6, PSMD7, PSMB5, PSMD4, PSMC4, PSMB3, PSMC2, PSME1, MCM6, ANAPC1, SKP1 |
| 46 | Degradation of DVL | 17 | R-HSA-4641258 | 2.48E-10 | PSMD10, PSMD11, PSMD13, PSMA7, PSMD8, PSMA5, PSMB6, PSMA6, PSMB4, PSMC6, PSMD7, PSMB5, PSMD4, PSMC4, PSMB3, PSMC2, PSME1 |
| 47 | Stabilization of p53 | 17 | R-HSA-69541 | 2.48E-10 | PSMD10, PSMD11, PSMD13, PSMA7, PSMD8, PSMA5, PSMB6, PSMA6, PSMB4, PSMC6, PSMD7, PSMB5, PSMD4, PSMC4, PSMB3, PSMC2, PSME1 |
| 48 | APC:Cdc20 mediated degradation of cell cycle proteins prior to satisfation of the cell cycle checkpoint | 19 | R-HSA-179419 | 2.51E-10 | PSMD10, PSMD11, PSMD13, PSMA7, PSMD8, PSMA5, PSMB6, PSMA6, PSMB4, PSMC6, PSMD7, PSMB5, PSMD4, PSMC4, PSMB3, PSMC2, PSME1, BUB3, ANAPC1 |
| 49 | Signaling by ROBO receptors | 32 | R-HSA-376176 | 3.05E-10 | PSMD10, RPL5, RPL30, PSMD11, RBM8A, PSMD13, RPLP1, RPL12, RPL10A, PSMA7, RPS15, PSMD8, PSMB6, PSMB4, PSMD7, PSMB5, PSMD4, PSMB3, RPLP2, RPS2, RPL17, RPL23, RPSA, PSMA5, PSMA6, RPS28, PSMC6, PSMC4, PSMC2, PSME1, RNPS1, RPS21 |
| 50 | Infectious disease | 78 | R-HSA-5663205 | 3.07E-10 | ITGB1, RPL5, RPL30, FEN1, AHCYL1, RPLP1, CTNND1, PSIP1, RPL10A, ACTB, RPS15, PSMD8, TUBB6, PSMD7, TRIM28, PSMD4, RPLP2, RCC1, NELFB, AP2M1, SKP1, ACTR3, RPL23, RPSA, ATP1B3, GTF2F2, TUBA4A, DDOST, PSMA5, PSMA6, TUBB2B, TBL1XR1, PSME1, CHMP4B, PPIG, SLC25A5, RAB7A, PSMD10, GTF2A1, PSMD11, ROCK2, PSMD13, RPL12, RPN1, GNAI3, ATP1A1, PSMA7, PSMB6, PSMB4, TKFC, PSMB5, LARP1, RBBP4, POLR2B, PSMB3, PCBP2, PGK1, SAP18, RPS2, PLCG1, HNRNPA1, RAE1, RPL17, RANBP2, NPM1, NUP153, TJP1, RPS28, PSMC6, ERCC3, PSMC4, IMPDH2, GNB2, PSMC2, UBE2N, CTNNB1, RPS21, NUP37 |
| 51 | mRNA Splicing - Major Pathway | 31 | R-HSA-72163 | 3.32E-10 | RBM25, SF3B2, RBM8A, SF3B6, SRRT, DDX42, CCAR1, PUF60, POLR2B, PPP1R8, PCBP2, SAP18, SNRPB2, HNRNPA1, SF3B1, SRRM2, HNRNPA3, FUS, ALYREF, YJU2, GTF2F2, U2SURP, SRRM1, HNRNPL, HNRNPF, HNRNPD, ACIN1, SRSF3, RNPS1, PPIG, HNRNPC |
| 52 | Activation of NF-kappaB in B cells | 18 | R-HSA-1169091 | 3.90E-10 | PSMD10, PSMD11, PSMD13, PSMA7, PSMD8, PSMA5, PSMB6, PSMA6, PSMB4, PSMC6, PSMD7, PSMB5, PSMD4, PSMC4, PSMB3, PSMC2, PSME1, SKP1 |
| 53 | Formation of paraxial mesoderm | 18 | R-HSA-9793380 | 3.90E-10 | PSMD10, PSMD11, PSMD13, PSMA7, PSMD8, PSMA5, PSMB6, PSMA6, PSMB4, PSMC6, PSMD7, PSMB5, PSMD4, PSMC4, PSMB3, PSMC2, PSME1, CTNNB1 |
| 54 | APC/C:Cdc20 mediated degradation of mitotic proteins | 19 | R-HSA-176409 | 4.03E-10 | PSMD10, PSMD11, PSMD13, PSMA7, PSMD8, PSMA5, PSMB6, PSMA6, PSMB4, PSMC6, PSMD7, PSMB5, PSMD4, PSMC4, PSMB3, PSMC2, PSME1, BUB3, ANAPC1 |
| 55 | Hh mutants abrogate ligand secretion | 17 | R-HSA-5387390 | 4.38E-10 | PSMD10, PSMD11, PSMD13, PSMA7, PSMD8, PSMA5, PSMB6, PSMA6, PSMB4, PSMC6, PSMD7, PSMB5, PSMD4, PSMC4, PSMB3, PSMC2, PSME1 |
| 56 | APC/C:Cdc20 mediated degradation of Securin | 18 | R-HSA-174154 | 5.01E-10 | PSMD10, PSMD11, PSMD13, PSMA7, PSMD8, PSMA5, PSMB6, PSMA6, PSMB4, PSMC6, PSMD7, PSMB5, PSMD4, PSMC4, PSMB3, PSMC2, PSME1, ANAPC1 |
| 57 | Activation of APC/C and APC/C:Cdc20 mediated degradation of mitotic proteins | 19 | R-HSA-176814 | 5.08E-10 | PSMD10, PSMD11, PSMD13, PSMA7, PSMD8, PSMA5, PSMB6, PSMA6, PSMB4, PSMC6, PSMD7, PSMB5, PSMD4, PSMC4, PSMB3, PSMC2, PSME1, BUB3, ANAPC1 |
| 58 | Translation | 37 | R-HSA-72766 | 5.68E-10 | RPL5, EIF4A1, RPL30, RPLP1, RPL12, RPN1, SRP54, RPL10A, YARS1, RPS15, EEF1B2, MRPL40, SSR1, RPLP2, RPS2, SEC11A, RPL17, EIF1AX, RPL23, RPSA, MRPL21, DDOST, AIMP1, RPS28, EIF3M, SARS2, EIF2S3, EIF3K, EPRS1, EEF1D, EIF3F, CARS1, FARSA, EIF3C, RPS21, MRRF, DAP3 |
| 59 | KEAP1-NFE2L2 pathway | 22 | R-HSA-9755511 | 5.89E-10 | PSMD10, PSMD11, PSMD13, IDH1, CSNK2A2, GSR, PSMA7, PSMD8, PSMA5, PSMB6, PSMA6, PSMB4, PSMC6, PSMD7, PSMB5, PSMD4, PSMC4, PSMB3, PSMC2, PSME1, UBXN7, SKP1 |
| 60 | Regulation of PTEN stability and activity | 18 | R-HSA-8948751 | 6.42E-10 | PSMD10, PSMD11, PSMD13, CSNK2A2, PSMA7, PSMD8, PSMA5, PSMB6, PSMA6, PSMB4, PSMC6, PSMD7, PSMB5, PSMD4, PSMC4, PSMB3, PSMC2, PSME1 |
| 61 | Programmed Cell Death | 31 | R-HSA-5357801 | 6.74E-10 | AVEN, PSMD10, PSMD11, PSMD13, HMGB1, PSMA7, LMNB1, PSMD8, PSMB6, SDCBP, PSMB4, PSMD7, PSMB5, PSMD4, PSMB3, C1QBP, CASP3, LMNA, DBNL, TMED7-TICAM2, TJP1, PSMA5, PSMA6, PSMC6, PSMC4, CDC37, PSMC2, PSME1, ACIN1, CTNNB1, CHMP4B |
| 62 | APC/C-mediated degradation of cell cycle proteins | 20 | R-HSA-174143 | 7.16E-10 | PSMD10, PSMD11, PSMD13, PSMA7, PSMD8, PSMA5, PSMB6, PSMA6, PSMB4, PSMC6, PSMD7, PSMB5, PSMD4, PSMC4, PSMB3, PSMC2, PSME1, BUB3, ANAPC1, SKP1 |
| 63 | Regulation of mitotic cell cycle | 20 | R-HSA-453276 | 7.16E-10 | PSMD10, PSMD11, PSMD13, PSMA7, PSMD8, PSMA5, PSMB6, PSMA6, PSMB4, PSMC6, PSMD7, PSMB5, PSMD4, PSMC4, PSMB3, PSMC2, PSME1, BUB3, ANAPC1, SKP1 |
| 64 | Defective CFTR causes cystic fibrosis | 17 | R-HSA-5678895 | 7.55E-10 | PSMD10, PSMD11, PSMD13, PSMA7, PSMD8, PSMA5, PSMB6, PSMA6, PSMB4, PSMC6, PSMD7, PSMB5, PSMD4, PSMC4, PSMB3, PSMC2, PSME1 |
| 65 | mRNA Splicing | 31 | R-HSA-72172 | 8.48E-10 | RBM25, SF3B2, RBM8A, SF3B6, SRRT, DDX42, CCAR1, PUF60, POLR2B, PPP1R8, PCBP2, SAP18, SNRPB2, HNRNPA1, SF3B1, SRRM2, HNRNPA3, FUS, ALYREF, YJU2, GTF2F2, U2SURP, SRRM1, HNRNPL, HNRNPF, HNRNPD, ACIN1, SRSF3, RNPS1, PPIG, HNRNPC |
| 66 | Apoptosis | 28 | R-HSA-109581 | 1.20E-09 | AVEN, PSMD10, PSMD11, PSMD13, HMGB1, PSMA7, LMNB1, PSMD8, PSMB6, PSMB4, PSMD7, PSMB5, PSMD4, PSMB3, C1QBP, CASP3, LMNA, DBNL, TMED7-TICAM2, TJP1, PSMA5, PSMA6, PSMC6, PSMC4, PSMC2, PSME1, ACIN1, CTNNB1 |
| 67 | Cellular responses to stress | 67 | R-HSA-2262752 | 1.23E-09 | RPL5, RPL30, ACADVL, RPLP1, RPL10A, LMNB1, RPS15, PSMD8, TUBB6, PSMD7, PSMD4, RPLP2, UBXN7, ATP6V1E1, SKP1, RPL23, CSNK2A2, RPSA, TUBA4A, PSMA5, PSMA6, TUBB2B, DNAJC7, TBL1XR1, DNAJB11, CAT, PSME1, ANAPC1, DCTN6, PSMD10, PSMD11, PSMD13, DCTN1, RPL12, COX5A, PSMA7, PRDX3, PSMB6, EXOSC6, PSMB4, PSMB5, RBBP4, PSMB3, LMNA, EP400, SSR1, RPS2, RAE1, EXOSC2, RPL17, RANBP2, HSPA9, HSPA5, IDH1, GSR, NUP153, EIF2AK4, SOD2, PRDX6, SOD1, RPS28, EIF2S3, PSMC6, PSMC4, PSMC2, RPS21, NUP37 |
| 68 | FCERI mediated NF-kB activation | 19 | R-HSA-2871837 | 1.52E-09 | PSMD10, PSMD11, PSMD13, PSMA7, PSMD8, PSMA5, PSMB6, PSMA6, PSMB4, PSMC6, PSMD7, PSMB5, PSMD4, PSMC4, PSMB3, PSMC2, UBE2N, PSME1, SKP1 |
| 69 | Asymmetric localization of PCP proteins | 17 | R-HSA-4608870 | 1.63E-09 | PSMD10, PSMD11, PSMD13, PSMA7, PSMD8, PSMA5, PSMB6, PSMA6, PSMB4, PSMC6, PSMD7, PSMB5, PSMD4, PSMC4, PSMB3, PSMC2, PSME1 |
| 70 | CDK-mediated phosphorylation and removal of Cdc6 | 18 | R-HSA-69017 | 1.65E-09 | PSMD10, PSMD11, PSMD13, PSMA7, PSMD8, PSMA5, PSMB6, PSMA6, PSMB4, PSMC6, PSMD7, PSMB5, PSMD4, PSMC4, PSMB3, PSMC2, PSME1, ANAPC1 |
| 71 | Regulation of RUNX2 expression and activity | 18 | R-HSA-8939902 | 1.65E-09 | PSMD10, PSMD11, PSMD13, PSMA7, PSMD8, PSMA5, PSMB6, PSMA6, PSMB4, PSMC6, PSMD7, PSMB5, PSMD4, PSMC4, PSMB3, PSMC2, PSME1, SKP1 |
| 72 | Axon guidance | 53 | R-HSA-422475 | 1.78E-09 | ITGB1, RPL5, RPL30, RPLP1, RPL10A, ACTB, RPS15, PSMD8, TUBB6, PSMD7, PSMD4, CFL1, RPLP2, NRCAM, ITGAV, PLXNC1, AP2M1, ACTR3, RPL23, CSNK2A2, RPSA, TUBA4A, DNM2, PSMA5, PSMA6, TUBB2B, PSME1, EPHA2, PSMD10, PSMD11, RBM8A, ROCK2, PSMD13, RPL12, MYL12A, PSMA7, PSMB6, SDCBP, PSMB4, PSMB5, PSMB3, RPS2, PLCG1, RPL17, SPTBN1, DLG1, RPS28, PSMC6, PSMC4, PSMC2, CNTN2, RNPS1, RPS21 |
| 73 | ABC-family proteins mediated transport | 21 | R-HSA-382556 | 1.86E-09 | PSMD10, PEX19, PSMD11, ABCB7, PSMD13, PSMA7, PSMD8, PSMA5, PSMB6, PSMA6, PSMB4, PSMC6, EIF2S3, PSMD7, PSMB5, PSMD4, PSMC4, PEX3, PSMB3, PSMC2, PSME1 |
| 74 | Cyclin E associated events during G1/S transition | 19 | R-HSA-69202 | 1.87E-09 | PSMD10, PSMD11, PSMD13, PSMA7, PSMD8, PSMA5, PSMB6, PSMA6, PSMB4, PSMC6, PSMD7, PSMB5, RBBP4, PSMD4, PSMC4, PSMB3, PSMC2, PSME1, SKP1 |
| 75 | Degradation of beta-catenin by the destruction complex | 19 | R-HSA-195253 | 1.87E-09 | PSMD10, PSMD11, PSMD13, PSMA7, PSMD8, PSMA5, PSMB6, PSMA6, PSMB4, PSMC6, PSMD7, PSMB5, PSMD4, PSMC4, PSMB3, PSMC2, PSME1, CTNNB1, SKP1 |
| 76 | APC/C:Cdh1 mediated degradation of Cdc20 and other APC/C:Cdh1 targeted proteins in late mitosis/early G1 | 18 | R-HSA-174178 | 2.07E-09 | PSMD10, PSMD11, PSMD13, PSMA7, PSMD8, PSMA5, PSMB6, PSMA6, PSMB4, PSMC6, PSMD7, PSMB5, PSMD4, PSMC4, PSMB3, PSMC2, PSME1, ANAPC1 |
| 77 | Hedgehog ligand biogenesis | 17 | R-HSA-5358346 | 2.09E-09 | PSMD10, PSMD11, PSMD13, PSMA7, PSMD8, PSMA5, PSMB6, PSMA6, PSMB4, PSMC6, PSMD7, PSMB5, PSMD4, PSMC4, PSMB3, PSMC2, PSME1 |
| 78 | Cellular responses to stimuli | 67 | R-HSA-8953897 | 2.55E-09 | RPL5, RPL30, ACADVL, RPLP1, RPL10A, LMNB1, RPS15, PSMD8, TUBB6, PSMD7, PSMD4, RPLP2, UBXN7, ATP6V1E1, SKP1, RPL23, CSNK2A2, RPSA, TUBA4A, PSMA5, PSMA6, TUBB2B, DNAJC7, TBL1XR1, DNAJB11, CAT, PSME1, ANAPC1, DCTN6, PSMD10, PSMD11, PSMD13, DCTN1, RPL12, COX5A, PSMA7, PRDX3, PSMB6, EXOSC6, PSMB4, PSMB5, RBBP4, PSMB3, LMNA, EP400, SSR1, RPS2, RAE1, EXOSC2, RPL17, RANBP2, HSPA9, HSPA5, IDH1, GSR, NUP153, EIF2AK4, SOD2, PRDX6, SOD1, RPS28, EIF2S3, PSMC6, PSMC4, PSMC2, RPS21, NUP37 |
| 79 | Cellular response to chemical stress | 29 | R-HSA-9711123 | 2.60E-09 | PSMD10, PSMD11, PSMD13, COX5A, PSMA7, PSMD8, PRDX3, PSMB6, PSMB4, PSMD7, PSMB5, PSMD4, PSMB3, UBXN7, SKP1, IDH1, CSNK2A2, GSR, SOD2, PRDX6, SOD1, PSMA5, PSMA6, PSMC6, PSMC4, TBL1XR1, PSMC2, CAT, PSME1 |
| 80 | p53-Dependent G1 DNA Damage Response | 17 | R-HSA-69563 | 2.66E-09 | PSMD10, PSMD11, PSMD13, PSMA7, PSMD8, PSMA5, PSMB6, PSMA6, PSMB4, PSMC6, PSMD7, PSMB5, PSMD4, PSMC4, PSMB3, PSMC2, PSME1 |
| 81 | p53-Dependent G1/S DNA damage checkpoint | 17 | R-HSA-69580 | 2.66E-09 | PSMD10, PSMD11, PSMD13, PSMA7, PSMD8, PSMA5, PSMB6, PSMA6, PSMB4, PSMC6, PSMD7, PSMB5, PSMD4, PSMC4, PSMB3, PSMC2, PSME1 |
| 82 | Oxygen-dependent proline hydroxylation of Hypoxia-inducible Factor Alpha | 17 | R-HSA-1234176 | 2.66E-09 | PSMD10, PSMD11, PSMD13, PSMA7, PSMD8, PSMA5, PSMB6, PSMA6, PSMB4, PSMC6, PSMD7, PSMB5, PSMD4, PSMC4, PSMB3, PSMC2, PSME1 |
| 83 | Cyclin A:Cdk2-associated events at S phase entry | 19 | R-HSA-69656 | 2.82E-09 | PSMD10, PSMD11, PSMD13, PSMA7, PSMD8, PSMA5, PSMB6, PSMA6, PSMB4, PSMC6, PSMD7, PSMB5, RBBP4, PSMD4, PSMC4, PSMB3, PSMC2, PSME1, SKP1 |
| 84 | Nervous system development | 54 | R-HSA-9675108 | 2.98E-09 | ITGB1, RPL5, RPL30, RPLP1, RPL10A, ACTB, RPS15, PSMD8, TUBB6, PSMD7, PSMD4, CFL1, RPLP2, NRCAM, ITGAV, PLXNC1, AP2M1, ACTR3, RPL23, CSNK2A2, RPSA, TUBA4A, DNM2, PSMA5, PSMA6, TUBB2B, PSME1, EPHA2, PSMD10, PSMD11, RBM8A, ROCK2, PSMD13, RPL12, MYL12A, PSMA7, PSMB6, SDCBP, PSMB4, PSMB5, PSMB3, RPS2, PLCG1, RPL17, SPTBN1, SMARCA4, DLG1, RPS28, PSMC6, PSMC4, PSMC2, CNTN2, RNPS1, RPS21 |
| 85 | S Phase | 26 | R-HSA-69242 | 3.13E-09 | PSMD10, FEN1, PSMD11, MCM7, PSMD13, PSMA7, PSMD8, PSMB6, PSMB4, PSMD7, PSMB5, PSMD4, RBBP4, PSMB3, POLD2, SKP1, SMC1A, PSMA5, POLA1, PSMA6, PSMC6, PSMC4, PSMC2, PSME1, MCM6, ANAPC1 |
| 86 | RHOBTB GTPase Cycle | 13 | R-HSA-9706574 | 3.50E-09 | CPSF7, ROCK2, ACTN1, TXNL1, TWF1, SRRM1, COPS4, CDC37, MYO6, RBBP6, PHIP, HNRNPC, DBN1 |
| 87 | Regulation of RAS by GAPs | 17 | R-HSA-5658442 | 4.26E-09 | PSMD10, PSMD11, PSMD13, PSMA7, PSMD8, PSMA5, PSMB6, PSMA6, PSMB4, PSMC6, PSMD7, PSMB5, PSMD4, PSMC4, PSMB3, PSMC2, PSME1 |
| 88 | G1/S DNA Damage Checkpoints | 17 | R-HSA-69615 | 4.26E-09 | PSMD10, PSMD11, PSMD13, PSMA7, PSMD8, PSMA5, PSMB6, PSMA6, PSMB4, PSMC6, PSMD7, PSMB5, PSMD4, PSMC4, PSMB3, PSMC2, PSME1 |
| 89 | Separation of Sister Chromatids | 28 | R-HSA-2467813 | 4.55E-09 | PSMD10, PSMD11, PSMD13, PSMA7, PSMD8, PSMB6, TUBB6, PSMB4, PSMD7, PSMB5, PSMD4, PSMB3, BUB3, RANBP2, SMC1A, CKAP5, TUBA4A, PSMA5, PSMA6, TUBB2B, CLIP1, PSMC6, PSMC4, PSMC2, PSME1, MAPRE1, ANAPC1, NUP37 |
| 90 | G1/S Transition | 23 | R-HSA-69206 | 5.02E-09 | PSMD10, PSMD11, MCM7, PSMD13, PSMA7, PSMD8, PSMA5, DHFR, PSMB6, POLA1, PSMA6, PSMB4, PSMC6, PSMD7, PSMB5, RBBP4, PSMD4, PSMC4, PSMB3, PSMC2, PSME1, MCM6, SKP1 |
| 91 | ER-Phagosome pathway | 19 | R-HSA-1236974 | 7.41E-09 | PSMD10, PSMD11, PSMD13, HMGB1, PSMA7, PSMD8, PSMA5, PSMB6, PSMA6, PSMB4, PSMC6, PSMD7, PSMB5, PSMD4, PSMC4, PSMB3, PSMC2, PSME1, SEC22B |
| 92 | CLEC7A (Dectin-1) signaling | 20 | R-HSA-5607764 | 8.24E-09 | PSMD10, AHCYL1, PSMD11, PSMD13, PSMA7, PSMD8, PSMA5, PSMB6, PSMA6, PSMB4, PSMC6, PSMD7, PSMB5, PSMD4, PSMC4, PSMB3, PSMC2, UBE2N, PSME1, SKP1 |
| 93 | UCH proteinases | 20 | R-HSA-5689603 | 9.77E-09 | PSMD10, PSMD11, PSMD13, ACTL6A, PSMA7, ACTB, PSMD8, PSMA5, PSMB6, PSMA6, PSMB4, PSMC6, PSMD7, PSMB5, PSMD4, PSMC4, PSMB3, PSMC2, RUVBL1, PSME1 |
| 94 | Hedgehog 'off' state | 21 | R-HSA-5610787 | 9.98E-09 | PSMD10, PSMD11, PSMD13, TUBA4A, PSMA7, PSMD8, PSMA5, PSMB6, PSMA6, TUBB6, TUBB2B, PSMB4, PSMC6, PSMD7, PSMB5, PSMD4, PSMC4, PSMB3, PSMC2, PSME1, SKP1 |
| 95 | Signaling by NOTCH4 | 18 | R-HSA-9013694 | 1.10E-08 | PSMD10, PSMD11, PSMD13, PSMA7, PSMD8, PSMA5, PSMB6, PSMA6, PSMB4, PSMC6, PSMD7, PSMB5, PSMD4, PSMC4, PSMB3, PSMC2, PSME1, SKP1 |
| 96 | Downstream signaling events of B Cell Receptor (BCR) | 18 | R-HSA-1168372 | 1.33E-08 | PSMD10, PSMD11, PSMD13, PSMA7, PSMD8, PSMA5, PSMB6, PSMA6, PSMB4, PSMC6, PSMD7, PSMB5, PSMD4, PSMC4, PSMB3, PSMC2, PSME1, SKP1 |
| 97 | Interleukin-1 signaling | 21 | R-HSA-9020702 | 1.36E-08 | PSMD10, USP14, PSMD11, PSMD13, HMGB1, PSMA7, PSMD8, PSMA5, PSMB6, PSMA6, PSMB4, PSMC6, PSMD7, PSMB5, PSMD4, PSMC4, PSMB3, PSMC2, UBE2N, PSME1, SKP1 |
| 98 | Antigen processing-Cross presentation | 20 | R-HSA-1236975 | 1.61E-08 | PSMD10, PSMD11, PSMD13, HMGB1, PSMA7, PSMD8, PSMA5, PSMB6, PSMA6, PSMB4, PSMC6, PSMD7, PSMB5, PSMD4, PSMC4, PSMB3, PSMC2, PSME1, ITGAV, SEC22B |
| 99 | Cellular response to hypoxia | 17 | R-HSA-1234174 | 1.93E-08 | PSMD10, PSMD11, PSMD13, PSMA7, PSMD8, PSMA5, PSMB6, PSMA6, PSMB4, PSMC6, PSMD7, PSMB5, PSMD4, PSMC4, PSMB3, PSMC2, PSME1 |
| 100 | Cell Cycle, Mitotic | 51 | R-HSA-69278 | 2.10E-08 | FEN1, MCM7, LMNB1, SMC2, PSMD8, GOLGA2, TUBB6, PCM1, PSMD7, PSMD4, PPME1, RCC1, LBR, EMD, SKP1, CSNK2A2, SMC1A, CKAP5, TUBA4A, PSMA5, PSMA6, TUBB2B, CLIP1, PSME1, CHMP4B, MCM6, MAPRE1, ANAPC1, PSMD10, PSMD11, PSMD13, DCTN1, RAB1B, PSMA7, PSMB6, PSMB4, PSMB5, RBBP4, PSMB3, LMNA, POLD2, BUB3, RAE1, RANBP2, NUP153, DHFR, POLA1, PSMC6, PSMC4, PSMC2, NUP37 |
| 101 | rRNA processing | 28 | R-HSA-72312 | 2.14E-08 | RPL5, RPL30, RPLP1, RPL12, NIP7, NOP2, RPL10A, RPS15, EXOSC6, EXOSC10, RPLP2, UTP20, RPS2, EXOSC2, RPL17, UTP14A, UTP15, RPL23, WDR18, ELAC2, RPSA, GNL3, BOP1, RPS28, LAS1L, NHP2, TSR1, RPS21 |
| 102 | Downstream TCR signaling | 19 | R-HSA-202424 | 3.02E-08 | PSMD10, PSMD11, PSMD13, PSMA7, PSMD8, PSMA5, PSMB6, PSMA6, PSMB4, PSMC6, PSMD7, PSMB5, PSMD4, PSMC4, PSMB3, PSMC2, UBE2N, PSME1, SKP1 |
| 103 | rRNA processing in the nucleus and cytosol | 27 | R-HSA-8868773 | 3.04E-08 | RPL5, RPL30, RPLP1, RPL12, NIP7, NOP2, RPL10A, RPS15, EXOSC6, EXOSC10, RPLP2, UTP20, RPS2, EXOSC2, RPL17, UTP14A, UTP15, RPL23, WDR18, RPSA, GNL3, BOP1, RPS28, LAS1L, NHP2, TSR1, RPS21 |
| 104 | M Phase | 42 | R-HSA-68886 | 3.08E-08 | PSMD10, PSMD11, PSMD13, DCTN1, RAB1B, PSMA7, SMC2, LMNB1, PSMD8, GOLGA2, PSMB6, TUBB6, PCM1, PSMB4, PSMD7, PSMB5, PSMD4, PSMB3, LMNA, RCC1, BUB3, LBR, EMD, RAE1, RANBP2, CSNK2A2, NUP153, SMC1A, CKAP5, TUBA4A, PSMA5, PSMA6, TUBB2B, CLIP1, PSMC6, PSMC4, PSMC2, PSME1, CHMP4B, MAPRE1, ANAPC1, NUP37 |
| 105 | Diseases of signal transduction by growth factor receptors and second messengers | 43 | R-HSA-5663202 | 3.09E-08 | PSMD10, PSMD11, FAM114A2, PSMD13, DCTN1, PEBP1, RRBP1, KLC1, PSMA7, ACTB, PSMD8, FXR1, PSMB6, PSMB4, ZMYM2, PSMD7, PSMB5, FIP1L1, PSMD4, POLR2B, PSMB3, LMNA, PLCG1, LRRFIP1, SPTBN1, SKP1, RANBP2, NPM1, TPM3, AGK, GTF2F2, QKI, PSMA5, PSMA6, PSMC6, PSMC4, TBL1XR1, CDC37, GOLGB1, PSMC2, PSME1, CTNNB1, CARS1 |
| 106 | G2/M Transition | 27 | R-HSA-69275 | 3.39E-08 | PSMD10, PSMD11, PSMD13, DCTN1, PSMA7, PSMD8, PSMB6, TUBB6, PCM1, PSMB4, PSMD7, PSMB5, PSMD4, RBBP4, PPME1, PSMB3, SKP1, CKAP5, TUBA4A, PSMA5, PSMA6, TUBB2B, PSMC6, PSMC4, PSMC2, PSME1, MAPRE1 |
| 107 | ABC transporter disorders | 17 | R-HSA-5619084 | 3.50E-08 | PSMD10, PSMD11, PSMD13, PSMA7, PSMD8, PSMA5, PSMB6, PSMA6, PSMB4, PSMC6, PSMD7, PSMB5, PSMD4, PSMC4, PSMB3, PSMC2, PSME1 |
| 108 | Mitotic G2-G2/M phases | 27 | R-HSA-453274 | 4.18E-08 | PSMD10, PSMD11, PSMD13, DCTN1, PSMA7, PSMD8, PSMB6, TUBB6, PCM1, PSMB4, PSMD7, PSMB5, PSMD4, RBBP4, PPME1, PSMB3, SKP1, CKAP5, TUBA4A, PSMA5, PSMA6, TUBB2B, PSMC6, PSMC4, PSMC2, PSME1, MAPRE1 |
| 109 | L13a-mediated translational silencing of Ceruloplasmin expression | 20 | R-HSA-156827 | 4.79E-08 | RPL5, EIF4A1, RPL30, EIF1AX, RPLP1, RPL23, RPL12, RPSA, RPL10A, RPS15, EIF3M, RPS28, EIF2S3, EIF3K, RPLP2, RPS2, EIF3F, EIF3C, RPS21, RPL17 |
| 110 | GTP hydrolysis and joining of the 60S ribosomal subunit | 20 | R-HSA-72706 | 5.56E-08 | RPL5, EIF4A1, RPL30, EIF1AX, RPLP1, RPL23, RPL12, RPSA, RPL10A, RPS15, EIF3M, RPS28, EIF2S3, EIF3K, RPLP2, RPS2, EIF3F, EIF3C, RPS21, RPL17 |
| 111 | Mitotic G1 phase and G1/S transition | 23 | R-HSA-453279 | 5.68E-08 | PSMD10, PSMD11, MCM7, PSMD13, PSMA7, PSMD8, PSMA5, DHFR, PSMB6, POLA1, PSMA6, PSMB4, PSMC6, PSMD7, PSMB5, RBBP4, PSMD4, PSMC4, PSMB3, PSMC2, PSME1, MCM6, SKP1 |
| 112 | PCP/CE pathway | 18 | R-HSA-4086400 | 6.69E-08 | PSMD10, PSMD11, PSMD13, PSMA7, PSMD8, PSMA5, PSMB6, PSMA6, PSMB4, PSMC6, PSMD7, PSMB5, PSMD4, PSMC4, PSMB3, PSMC2, PSME1, AP2M1 |
| 113 | Cell Cycle | 57 | R-HSA-1640170 | 8.21E-08 | FEN1, MCM7, LMNB1, SMC2, PSMD8, GOLGA2, TUBB6, PCM1, PSMD7, PSMD4, PPME1, RUVBL1, PPP6R3, RCC1, LBR, EMD, SKP1, CSNK2A2, SMC1A, CKAP5, TUBA4A, PSMA5, PSMA6, TUBB2B, CLIP1, PSME1, NHP2, CHMP4B, MCM6, MAPRE1, ANAPC1, PSMD10, PSMD11, PSMD13, DCTN1, RAB1B, PSMA7, PSMB6, PSMB4, PSMB5, RBBP4, POLR2B, PSMB3, LMNA, POLD2, BUB3, RAE1, RANBP2, NPM1, NUP153, DHFR, POLA1, PSMC6, PSMC4, PSMC2, UBE2N, NUP37 |
| 114 | Disease | 110 | R-HSA-1643685 | 8.49E-08 | RPL5, RPL30, AHCYL1, CTNND1, MPI, PEBP1, RPL10A, ACTB, RPS15, PSMD8, GOLGA2, TUBB6, PSMD7, PSMD4, TRIM28, FDXR, RCC1, AP2M1, SKP1, TPM3, RPL23, RPSA, ATP1B3, GTF2F2, TUBA4A, TBL1XR1, CDC37, PSME1, CHMP4B, PPIG, HPRT1, CARS1, ANAPC1, PSMD10, PSMD11, FAM114A2, PSMD13, RPN1, KLC1, ACACA, FXR1, LARP1, TKFC, PCBP2, PGK1, PLCG1, SPTBN1, IDH1, NUP153, QKI, POLA1, RPS28, GNB2, GOLGB1, UBE2N, RPS21, ITGB1, FEN1, RPLP1, PSIP1, HMGB1, LMNB1, ZMYM2, C1QBP, RPLP2, NELFB, LRRFIP1, ACTR3, GAA, DDOST, PSMA5, PSMA6, TUBB2B, SLC25A5, RAB7A, GTF2A1, ROCK2, DCTN1, RPL12, GNAI3, RRBP1, ATP1A1, PSMA7, PSMB6, PSMB4, PSMB5, RBBP4, FIP1L1, POLR2B, PSMB3, LMNA, SAP18, RPS2, HNRNPA1, RAE1, RPL17, RANBP2, NPM1, AGK, SOD2, TJP1, GALE, PSMC6, PC, ERCC3, PSMC4, IMPDH2, PSMC2, CTNNB1, NUP37 |
| 115 | Hedgehog 'on' state | 17 | R-HSA-5632684 | 1.25E-07 | PSMD10, PSMD11, PSMD13, PSMA7, PSMD8, PSMA5, PSMB6, PSMA6, PSMB4, PSMC6, PSMD7, PSMB5, PSMD4, PSMC4, PSMB3, PSMC2, PSME1 |
| 116 | Transcriptional regulation by RUNX3 | 18 | R-HSA-8878159 | 1.28E-07 | PSMD10, PSMD11, PSMD13, PSMA7, PSMD8, PSMA5, PSMB6, PSMA6, PSMB4, PSMC6, PSMD7, PSMB5, PSMD4, PSMC4, PSMB3, PSMC2, PSME1, CTNNB1 |
| 117 | Eukaryotic Translation Initiation | 20 | R-HSA-72613 | 1.50E-07 | RPL5, EIF4A1, RPL30, EIF1AX, RPLP1, RPL23, RPL12, RPSA, RPL10A, RPS15, EIF3M, RPS28, EIF2S3, EIF3K, RPLP2, RPS2, EIF3F, EIF3C, RPS21, RPL17 |
| 118 | Cap-dependent Translation Initiation | 20 | R-HSA-72737 | 1.50E-07 | RPL5, EIF4A1, RPL30, EIF1AX, RPLP1, RPL23, RPL12, RPSA, RPL10A, RPS15, EIF3M, RPS28, EIF2S3, EIF3K, RPLP2, RPS2, EIF3F, EIF3C, RPS21, RPL17 |
| 119 | TCR signaling | 20 | R-HSA-202403 | 1.50E-07 | PSMD10, PSMD11, PSMD13, PSMA7, PSMD8, PSMA5, PSMB6, PSMA6, PSMB4, PSMC6, PSMD7, PSMB5, PSMD4, PSMC4, PSMB3, PSMC2, UBE2N, PSME1, PLCG1, SKP1 |
| 120 | Major pathway of rRNA processing in the nucleolus and cytosol | 25 | R-HSA-6791226 | 1.76E-07 | RPL5, RPL30, RPLP1, RPL12, NIP7, RPL10A, RPS15, EXOSC6, EXOSC10, RPLP2, UTP20, RPS2, EXOSC2, RPL17, UTP14A, UTP15, RPL23, WDR18, RPSA, GNL3, BOP1, RPS28, LAS1L, TSR1, RPS21 |
| 121 | Metabolism | 126 | R-HSA-1430728 | 1.82E-07 | RPL5, RPL30, AHCYL1, GLDC, ECI2, RPL10A, NUDT5, CNDP2, RPS15, PSMD8, PSMD7, PSMD4, FDXR, CHP1, CPNE3, ACAA1, CIAO2B, RPL23, CSNK2A2, RPSA, ACLY, MTHFD1, TBL1XR1, PSME1, SMS, UQCRC1, SUCLG2, SUCLG1, HPRT1, PSMD10, ISYNA1, PSMD11, ABCB7, PSMD13, ACACA, NME1-NME2, TKFC, PSAP, PGK1, PLCG1, DECR1, MPST, PDHA1, OSBPL6, IDH1, PYCR1, NUP153, AIMP1, DHFR, GRHPR, RPS28, EPRS1, GNB2, ACO2, RPS21, ITPA, LGMN, ACADVL, RPLP1, TECR, LPCAT1, ETFA, DNPH1, GPHN, HS2ST1, SPTLC1, NNT, DBT, RPLP2, LBR, ENOPH1, ACAD8, CBR1, ADSL, TPI1, GAA, AKR1A1, DNM2, PSMA5, HADHB, PSMA6, DDAH1, DDAH2, IVD, AGPS, PLIN3, NDUFS1, UMPS, GAPDH, ASAH1, FH, GMPR2, RPE, RPL12, PDHB, COX5A, PSMA7, PSMB6, HMGCL, PSMB4, PSMB5, MTHFD1L, MAT2A, PSMB3, BPNT2, RPS2, RAE1, RPL17, RANBP2, MDH1, MDH2, AGK, GSR, FAH, DHODH, ALDH4A1, GALE, PSMC6, PC, RAB14, GNPDA2, PSMC4, IMPDH2, PSMC2, SCLY, NUP37 |
| 122 | Fc epsilon receptor (FCERI) signaling | 21 | R-HSA-2454202 | 1.92E-07 | PSMD10, AHCYL1, PSMD11, PSMD13, PSMA7, PSMD8, PSMA5, PSMB6, PSMA6, PSMB4, PSMC6, PSMD7, PSMB5, PSMD4, PSMC4, PSMB3, PSMC2, UBE2N, PSME1, PLCG1, SKP1 |
| 123 | Metabolism of proteins | 119 | R-HSA-392499 | 1.96E-07 | EIF4A1, RPL5, RPL30, MPI, YARS1, RPL10A, ACTB, RPS15, PSMD8, EEF1B2, GOLGA2, PCMT1, TUBB6, PSMD7, PSMD4, TRIM28, CDH2, RUVBL1, LONP2, SKP1, EIF1AX, RPL23, CSNK2A2, RPSA, RNF40, TUBA4A, PSME1, CARS1, DAP3, PSMD10, RAB5B, PSMD11, RAB5C, PSMD13, UBA6, PRKDC, RPN1, COPS7A, MLEC, SSR1, RAB6A, SPTBN1, NUP153, MRPL21, AIMP1, COPS4, RPS28, EIF2S3, COPS5, EPRS1, GNB2, GOLGB1, SP3, TBCE, CALU, UBE2N, PAF1, HNRNPC, FARSA, COPS8, RPS21, MCFD2, RPLP1, SRP54, MIA3, MRPL40, PPP6R1, PPP6R3, WDR5, PGM3, RPLP2, UBXN7, ACTL6A, ATP6AP2, SMC1A, DDOST, PSMA5, PSMA6, RCN1, TUBB2B, SKIC8, SARS2, EEF1D, PTRH2, TCP1, PFDN2, SEC22B, MRRF, RAB7A, DCTN6, USP14, DCTN1, RAB1B, RPL12, PSMA7, PSMB6, PSMB4, PSMB5, PSMB3, RPS2, CCT8, RAE1, UGGT1, SEC11A, RPL17, RANBP2, NPM1, TMED7-TICAM2, KTN1, EIF3M, PSMC6, RAB14, EIF3K, PSMC4, PSMC2, CTNNB1, EIF3F, EIF3C, NUP37 |
| 124 | Gastrulation | 18 | R-HSA-9758941 | 2.04E-07 | PSMD10, PSMD11, PSMD13, PSMA7, PSMD8, PSMA5, PSMB6, PSMA6, PSMB4, PSMC6, PSMD7, PSMB5, PSMD4, PSMC4, PSMB3, PSMC2, PSME1, CTNNB1 |
| 125 | MAPK6/MAPK4 signaling | 17 | R-HSA-5687128 | 2.43E-07 | PSMD10, PSMD11, PSMD13, PSMA7, PSMD8, PSMA5, PSMB6, PSMA6, PSMB4, PSMC6, PSMD7, PSMB5, PSMD4, PSMC4, PSMB3, PSMC2, PSME1 |
| 126 | Signaling by the B Cell Receptor (BCR) | 19 | R-HSA-983705 | 2.53E-07 | PSMD10, AHCYL1, PSMD11, PSMD13, PSMA7, PSMD8, PSMA5, PSMB6, PSMA6, PSMB4, PSMC6, PSMD7, PSMB5, PSMD4, PSMC4, PSMB3, PSMC2, PSME1, SKP1 |
| 127 | TNFR2 non-canonical NF-kB pathway | 18 | R-HSA-5668541 | 3.19E-07 | PSMD10, PSMD11, PSMD13, PSMA7, PSMD8, PSMA5, PSMB6, PSMA6, PSMB4, PSMC6, PSMD7, PSMB5, PSMD4, PSMC4, PSMB3, PSMC2, PSME1, SKP1 |
| 128 | Formation of a pool of free 40S subunits | 18 | R-HSA-72689 | 3.19E-07 | RPL5, RPL30, EIF1AX, RPLP1, RPL23, RPL12, RPSA, RPL10A, RPS15, EIF3M, RPS28, EIF3K, RPLP2, RPS2, EIF3F, EIF3C, RPS21, RPL17 |
| 129 | DNA Replication | 24 | R-HSA-69306 | 8.34E-07 | PSMD10, FEN1, PSMD11, MCM7, PSMD13, PSMA7, PSMD8, PSMA5, PSMB6, POLA1, PSMA6, PSMB4, PSMC6, PSMD7, PSMB5, PSMD4, PSMC4, PSMB3, PSMC2, POLD2, PSME1, MCM6, ANAPC1, SKP1 |
| 130 | Signaling by Hedgehog | 21 | R-HSA-5358351 | 1.10E-06 | PSMD10, PSMD11, PSMD13, TUBA4A, PSMA7, PSMD8, PSMA5, PSMB6, PSMA6, TUBB6, TUBB2B, PSMB4, PSMC6, PSMD7, PSMB5, PSMD4, PSMC4, PSMB3, PSMC2, PSME1, SKP1 |
| 131 | RHOBTB2 GTPase cycle | 9 | R-HSA-9013418 | 1.36E-06 | CDC37, ACTN1, MYO6, TXNL1, TWF1, PHIP, HNRNPC, DBN1, SRRM1 |
| 132 | RHOBTB1 GTPase cycle | 9 | R-HSA-9013422 | 1.36E-06 | COPS4, CPSF7, ROCK2, MYO6, TXNL1, RBBP6, HNRNPC, DBN1, SRRM1 |
| 133 | SRP-dependent cotranslational protein targeting to membrane | 18 | R-HSA-1799339 | 1.42E-06 | RPL5, RPL30, RPLP1, RPL23, RPL12, RPN1, SRP54, RPSA, RPL10A, DDOST, RPS15, RPS28, RPLP2, SSR1, RPS2, RPS21, SEC11A, RPL17 |
| 134 | Interleukin-1 family signaling | 21 | R-HSA-446652 | 1.68E-06 | PSMD10, USP14, PSMD11, PSMD13, HMGB1, PSMA7, PSMD8, PSMA5, PSMB6, PSMA6, PSMB4, PSMC6, PSMD7, PSMB5, PSMD4, PSMC4, PSMB3, PSMC2, UBE2N, PSME1, SKP1 |
| 135 | C-type lectin receptors (CLRs) | 20 | R-HSA-5621481 | 2.14E-06 | PSMD10, AHCYL1, PSMD11, PSMD13, PSMA7, PSMD8, PSMA5, PSMB6, PSMA6, PSMB4, PSMC6, PSMD7, PSMB5, PSMD4, PSMC4, PSMB3, PSMC2, UBE2N, PSME1, SKP1 |
| 136 | Assembly of the pre-replicative complex | 20 | R-HSA-68867 | 2.14E-06 | PSMD10, PSMD11, MCM7, PSMD13, PSMA7, PSMD8, PSMA5, PSMB6, PSMA6, PSMB4, PSMC6, PSMD7, PSMB5, PSMD4, PSMC4, PSMB3, PSMC2, PSME1, MCM6, ANAPC1 |
| 137 | DNA Replication Pre-Initiation | 21 | R-HSA-69002 | 3.07E-06 | PSMD10, PSMD11, MCM7, PSMD13, PSMA7, PSMD8, PSMA5, PSMB6, POLA1, PSMA6, PSMB4, PSMC6, PSMD7, PSMB5, PSMD4, PSMC4, PSMB3, PSMC2, PSME1, MCM6, ANAPC1 |
| 138 | Beta-catenin independent WNT signaling | 20 | R-HSA-3858494 | 3.26E-06 | PSMD10, PSMD11, PSMD13, PSMA7, PSMD8, PSMA5, PSMB6, PSMA6, PSMB4, PSMC6, PSMD7, PSMB5, PSMD4, PSMC4, PSMB3, GNB2, PSMC2, PSME1, CTNNB1, AP2M1 |
| 139 | Transcriptional regulation by RUNX2 | 18 | R-HSA-8878166 | 3.74E-06 | PSMD10, PSMD11, PSMD13, PSMA7, PSMD8, PSMA5, PSMB6, PSMA6, PSMB4, PSMC6, PSMD7, PSMB5, PSMD4, PSMC4, PSMB3, PSMC2, PSME1, SKP1 |
| 140 | Influenza Viral RNA Transcription and Replication | 19 | R-HSA-168273 | 4.64E-06 | RANBP2, RPL5, RPL30, RPLP1, RPL23, RPL12, RPSA, NUP153, RPL10A, GTF2F2, RPS15, RPS28, POLR2B, RPLP2, RPS2, RAE1, RPS21, RPL17, NUP37 |
| 141 | PTEN Regulation | 19 | R-HSA-6807070 | 7.02E-06 | PSMD10, PSMD11, PSMD13, CSNK2A2, PSMA7, PSMD8, PSMA5, PSMB6, PSMA6, PSMB4, PSMC6, PSMD7, PSMB5, RBBP4, PSMD4, PSMC4, PSMB3, PSMC2, PSME1 |
| 142 | Selenoamino acid metabolism | 17 | R-HSA-2408522 | 1.28E-05 | RPL5, RPL30, RPLP1, RPL23, RPL12, GSR, RPSA, RPL10A, RPS15, AIMP1, RPS28, EPRS1, RPLP2, RPS2, SCLY, RPS21, RPL17 |
| 143 | Translation initiation complex formation | 12 | R-HSA-72649 | 1.31E-05 | RPS15, EIF4A1, EIF3M, RPS28, EIF2S3, EIF3K, EIF1AX, RPSA, EIF3F, RPS2, EIF3C, RPS21 |
| 144 | Ribosomal scanning and start codon recognition | 12 | R-HSA-72702 | 1.31E-05 | RPS15, EIF4A1, EIF3M, RPS28, EIF2S3, EIF3K, EIF1AX, RPSA, EIF3F, RPS2, EIF3C, RPS21 |
| 145 | Eukaryotic Translation Elongation | 15 | R-HSA-156842 | 1.36E-05 | RPL5, RPL30, RPLP1, RPL23, RPL12, RPSA, RPL10A, RPS15, EEF1B2, RPS28, EEF1D, RPLP2, RPS2, RPS21, RPL17 |
| 146 | Disorders of transmembrane transporters | 21 | R-HSA-5619115 | 1.45E-05 | RANBP2, PSMD10, PSMD11, PSMD13, NUP153, PSMA7, PSMD8, PSMA5, PSMB6, PSMA6, PSMB4, PSMC6, PSMD7, PSMB5, PSMD4, PSMC4, PSMB3, PSMC2, PSME1, RAE1, NUP37 |
| 147 | Activation of the mRNA upon binding of the cap-binding complex and eIFs, and subsequent binding to 43S | 12 | R-HSA-72662 | 1.55E-05 | RPS15, EIF4A1, EIF3M, RPS28, EIF2S3, EIF3K, EIF1AX, RPSA, EIF3F, RPS2, EIF3C, RPS21 |
| 148 | G2/M Checkpoints | 20 | R-HSA-69481 | 2.33E-05 | PSMD10, PSMD11, MCM7, PSMD13, PSMA7, PSMD8, PSMA5, PSMB6, PSMA6, PSMB4, PSMC6, PSMD7, PSMB5, PSMD4, PSMC4, PSMB3, PSMC2, UBE2N, PSME1, MCM6 |
| 149 | Formation of the ternary complex, and subsequently, the 43S complex | 11 | R-HSA-72695 | 2.44E-05 | RPS15, EIF3M, RPS28, EIF2S3, EIF3K, EIF1AX, RPSA, EIF3F, RPS2, EIF3C, RPS21 |
| 150 | Influenza Infection | 19 | R-HSA-168255 | 3.44E-05 | RANBP2, RPL5, RPL30, RPLP1, RPL23, RPL12, RPSA, NUP153, RPL10A, GTF2F2, RPS15, RPS28, POLR2B, RPLP2, RPS2, RAE1, RPS21, RPL17, NUP37 |
| 151 | Response of EIF2AK4 (GCN2) to amino acid deficiency | 15 | R-HSA-9633012 | 3.51E-05 | RPL5, RPL30, RPLP1, RPL23, RPL12, RPSA, EIF2AK4, RPL10A, RPS15, RPS28, EIF2S3, RPLP2, RPS2, RPS21, RPL17 |
| 152 | RUNX1 regulates transcription of genes involved in differentiation of HSCs | 17 | R-HSA-8939236 | 3.56E-05 | PSMD10, PSMD11, PSMD13, PSMA7, PSMD8, PSMA5, PSMB6, PSMA6, PSMB4, PSMC6, PSMD7, PSMB5, PSMD4, PSMC4, PSMB3, PSMC2, PSME1 |
| 153 | Cell Cycle Checkpoints | 27 | R-HSA-69620 | 6.66E-05 | PSMD10, PSMD11, MCM7, PSMD13, PSMA7, PSMD8, PSMB6, PSMB4, PSMD7, PSMB5, PSMD4, PSMB3, BUB3, RANBP2, CKAP5, PSMA5, PSMA6, CLIP1, PSMC6, PSMC4, PSMC2, PSME1, UBE2N, MCM6, MAPRE1, ANAPC1, NUP37 |
| 154 | Signaling by Rho GTPases, Miro GTPases and RHOBTB3 | 50 | R-HSA-9716542 | 7.30E-05 | ITGB1, STEAP3, ACTB, LMNB1, AKAP12, TUBB6, C1QBP, CFL1, DBT, LBR, EMD, ACTR3, TPM3, ACTN1, CKAP5, TUBA4A, SRRM1, TUBB2B, CLIP1, CDC37, PLIN3, PHIP, MAPRE1, DBN1, EPHA2, RAB7A, ROCK2, TWF1, KLC1, ADD3, MYL12A, RHOT2, SAMM50, MYO6, RBBP6, BUB3, SPTBN1, RANBP2, FARP1, CPSF7, JUP, TXNL1, KTN1, COPS4, LETM1, CPD, PIN1, CTNNB1, HNRNPC, NUP37 |
| 155 | Neddylation | 24 | R-HSA-8951664 | 7.42E-05 | PSMD10, PSMD11, PSMD13, COPS7A, PSMA7, COPS4, PSMD8, PSMA5, PSMB6, PSMA6, PSMB4, PSMC6, COPS5, PSMD7, PSMB5, PSMD4, PSMC4, PSMB3, PSMC2, WDR5, PSME1, UBXN7, COPS8, SKP1 |
| 156 | Gene and protein expression by JAK-STAT signaling after Interleukin-12 stimulation | 9 | R-HSA-8950505 | 8.14E-05 | HSPA9, HNRNPF, CFL1, TCP1, PDCD4, MIF, SOD2, LMNB1, SOD1 |
| 157 | Transcriptional regulation by RUNX1 | 23 | R-HSA-8878171 | 1.25E-04 | PSMD10, SMARCD1, PSMD11, PSMD13, CSNK2A2, ACTL6A, PSMA7, SMARCA4, TJP1, PSMD8, PSMA5, PSMB6, PSMA6, PSMB4, PSMC6, PSMD7, PSMB5, PSMD4, PSMC4, PSMB3, PSMC2, WDR5, PSME1 |
| 158 | Nonsense-Mediated Decay (NMD) | 15 | R-HSA-927802 | 1.46E-04 | RPL5, RPL30, RBM8A, RPLP1, RPL23, RPL12, RPSA, RPL10A, RPS15, RPS28, RPLP2, RNPS1, RPS2, RPS21, RPL17 |
| 159 | Nonsense Mediated Decay (NMD) enhanced by the Exon Junction Complex (EJC) | 15 | R-HSA-975957 | 1.46E-04 | RPL5, RPL30, RBM8A, RPLP1, RPL23, RPL12, RPSA, RPL10A, RPS15, RPS28, RPLP2, RNPS1, RPS2, RPS21, RPL17 |
| 160 | Signaling by Rho GTPases | 48 | R-HSA-194315 | 1.64E-04 | ITGB1, STEAP3, ROCK2, TWF1, KLC1, ADD3, MYL12A, ACTB, LMNB1, AKAP12, TUBB6, SAMM50, C1QBP, DBT, MYO6, CFL1, RBBP6, BUB3, LBR, EMD, SPTBN1, RANBP2, ACTR3, FARP1, CPSF7, JUP, TPM3, ACTN1, TXNL1, CKAP5, TUBA4A, SRRM1, KTN1, COPS4, LETM1, TUBB2B, CLIP1, CDC37, CPD, CTNNB1, PIN1, PHIP, HNRNPC, MAPRE1, DBN1, RAB7A, NUP37, EPHA2 |
| 161 | Peptide chain elongation | 13 | R-HSA-156902 | 1.67E-04 | RPL5, RPL30, RPLP1, RPL23, RPL12, RPSA, RPL10A, RPS15, RPS28, RPLP2, RPS2, RPS21, RPL17 |
| 162 | Viral mRNA Translation | 13 | R-HSA-192823 | 1.67E-04 | RPL5, RPL30, RPLP1, RPL23, RPL12, RPSA, RPL10A, RPS15, RPS28, RPLP2, RPS2, RPS21, RPL17 |
| 163 | Innate Immune System | 65 | R-HSA-168249 | 2.26E-04 | AHCYL1, CD81, LPCAT1, HMGB1, ACTB, PSMD8, PSMD7, PSMD4, CFL1, CPNE3, ITGAV, LRRFIP1, ATP6V1E1, ACAA1, SKP1, ACTR3, DBNL, GAA, ATP6AP2, MIF, DDOST, DNM2, PSMA5, ACLY, PSMA6, CAT, PSME1, PAFAH1B2, RAB7A, PSMD10, USP14, ASAH1, RAB5B, PSMD11, RAB5C, PRKDC, PSMD13, PSMA7, NME1-NME2, PSMB6, SDCBP, PSMB4, TKFC, PSMB5, PSMB3, PSAP, PCBP2, MLEC, CCT8, PLCG1, RAB6A, JUP, IDH1, TMED7-TICAM2, PRDX6, ERP44, PSMC6, RAB14, PSMC4, IMPDH2, PSMC2, UBE2N, PIN1, CTNNB1, LGMN |
| 164 | Eukaryotic Translation Termination | 13 | R-HSA-72764 | 2.54E-04 | RPL5, RPL30, RPLP1, RPL23, RPL12, RPSA, RPL10A, RPS15, RPS28, RPLP2, RPS2, RPS21, RPL17 |
| 165 | Selenocysteine synthesis | 13 | R-HSA-2408557 | 2.54E-04 | RPL5, RPL30, RPLP1, RPL23, RPL12, RPSA, RPL10A, RPS15, RPS28, RPLP2, RPS2, RPS21, RPL17 |
| 166 | Signaling by NOTCH | 22 | R-HSA-157118 | 3.00E-04 | PSMD10, PSMD11, PSMD13, ATP2A2, PSMA7, PBX1, PSMD8, PSMA5, PSMB6, PSMA6, PSMB4, PSMC6, PSMD7, PSMB5, PSMD4, TBL1XR1, PSMC4, PSMB3, PSMC2, PSME1, RAB6A, SKP1 |
| 167 | Nonsense Mediated Decay (NMD) independent of the Exon Junction Complex (EJC) | 13 | R-HSA-975956 | 3.10E-04 | RPL5, RPL30, RPLP1, RPL23, RPL12, RPSA, RPL10A, RPS15, RPS28, RPLP2, RPS2, RPS21, RPL17 |
| 168 | Transport of Mature Transcript to Cytoplasm | 12 | R-HSA-72202 | 3.65E-04 | RANBP2, NXF1, RBM8A, FIP1L1, SARNP, ALYREF, SRSF3, NUP153, RNPS1, RAE1, SRRM1, NUP37 |
| 169 | Interleukin-12 signaling | 9 | R-HSA-9020591 | 3.86E-04 | HSPA9, HNRNPF, CFL1, TCP1, PDCD4, MIF, SOD2, LMNB1, SOD1 |
| 170 | Neutrophil degranulation | 35 | R-HSA-6798695 | 4.73E-04 | ASAH1, RAB5B, PSMD11, RAB5C, PSMD13, LPCAT1, HMGB1, NME1-NME2, SDCBP, PSMD7, PSAP, MLEC, ITGAV, CPNE3, CCT8, ACAA1, RAB6A, DBNL, JUP, IDH1, GAA, TMED7-TICAM2, ATP6AP2, MIF, PRDX6, DDOST, PSMA5, ERP44, ACLY, RAB14, IMPDH2, PSMC2, CAT, PAFAH1B2, RAB7A |
| 171 | Transport of Mature mRNA derived from an Intron-Containing Transcript | 11 | R-HSA-159236 | 5.79E-04 | RANBP2, NXF1, RBM8A, SARNP, ALYREF, SRSF3, NUP153, RNPS1, RAE1, SRRM1, NUP37 |
| 172 | Prefoldin mediated transfer of substrate to CCT/TriC | 7 | R-HSA-389957 | 5.84E-04 | TUBB2B, TUBB6, TCP1, PFDN2, CCT8, ACTB, TUBA4A |
| 173 | RHO GTPase cycle | 33 | R-HSA-9012999 | 6.01E-04 | ITGB1, STEAP3, ROCK2, TWF1, ADD3, ACTB, LMNB1, AKAP12, SAMM50, C1QBP, DBT, MYO6, RBBP6, LBR, EMD, SPTBN1, FARP1, CPSF7, JUP, TPM3, ACTN1, TXNL1, SRRM1, KTN1, COPS4, LETM1, CDC37, CPD, PHIP, HNRNPC, DBN1, RAB7A, EPHA2 |
| 174 | TCF dependent signaling in response to WNT | 21 | R-HSA-201681 | 6.57E-04 | PSMD10, PSMD11, PSMD13, CSNK2A2, PSMA7, SMARCA4, PSMD8, PSMA5, PSMB6, PSMA6, PSMB4, PSMC6, PSMD7, PSMB5, PSMD4, PSMC4, PSMB3, PSMC2, RUVBL1, PSME1, CTNNB1 |
| 175 | L1CAM interactions | 14 | R-HSA-373760 | 6.65E-04 | ITGB1, CSNK2A2, TUBA4A, ACTB, DNM2, SDCBP, TUBB6, DLG1, TUBB2B, CNTN2, NRCAM, ITGAV, AP2M1, SPTBN1 |
| 176 | Signaling by Interleukins | 34 | R-HSA-449147 | 7.14E-04 | PSMD10, ITGB1, USP14, PSMD11, PSMD13, HMGB1, PSMA7, LMNB1, CRKL, PSMD8, PSMB6, PSMB4, PSMD7, PSMB5, PSMD4, PSMB3, CASP3, CFL1, SKP1, HSPA9, MIF, SOD2, SMARCA4, SOD1, PSMA5, PSMA6, PSMC6, PSMC4, HNRNPF, PSMC2, TCP1, PSME1, UBE2N, PDCD4 |
| 177 | Class I MHC mediated antigen processing & presentation | 29 | R-HSA-983169 | 8.25E-04 | PSMD10, PSMD11, UBA6, PSMD13, MGRN1, UBA5, HMGB1, PSMA7, PSMD8, PSMB6, PSMB4, PSMD7, PSMB5, PSMD4, PSMB3, ITGAV, RBBP6, SKP1, HSPA5, PSMA5, PSMA6, PSMC6, PSMC4, PSMC2, PSME1, UBE2N, BLMH, SEC22B, ANAPC1 |
| 178 | Ub-specific processing proteases | 20 | R-HSA-5689880 | 8.72E-04 | PSMD10, USP14, PSMD11, PSMD13, PSMA7, PSMD8, PSMA5, PSMB6, PSMA6, PSMB4, PSMC6, PSMD7, PSMB5, PSMD4, PSMC4, PSMB3, PTRH2, PSMC2, RUVBL1, PSME1 |
| 179 | Antigen processing: Ubiquitination & Proteasome degradation | 25 | R-HSA-983168 | 8.92E-04 | PSMD10, PSMD11, UBA6, PSMD13, MGRN1, UBA5, PSMA7, PSMD8, PSMB6, PSMB4, PSMD7, PSMB5, PSMD4, PSMB3, RBBP6, SKP1, PSMA5, PSMA6, PSMC6, PSMC4, PSMC2, PSME1, UBE2N, BLMH, ANAPC1 |
| 180 | Antiviral mechanism by IFN-stimulated genes | 11 | R-HSA-1169410 | 9.70E-04 | RANBP2, EIF4A1, UBE2N, PIN1, NUP153, FLNB, PLCG1, ABCE1, RAE1, EIF4G3, NUP37 |
| 181 | RHOD GTPase cycle | 9 | R-HSA-9013405 | 1.01E-03 | AKAP12, STEAP3, ACTN1, ADD3, LBR, EMD, DBN1, LMNB1, RAB7A |
| 182 | RHOF GTPase cycle | 8 | R-HSA-9035034 | 1.02E-03 | AKAP12, FARP1, STEAP3, ACTN1, ADD3, ACTB, LMNB1, RAB7A |
| 183 | Cellular response to starvation | 16 | R-HSA-9711097 | 1.09E-03 | RPL5, RPL30, RPLP1, RPL23, RPL12, RPSA, EIF2AK4, RPL10A, RPS15, RPS28, EIF2S3, RPLP2, RPS2, ATP6V1E1, RPS21, RPL17 |
| 184 | Signaling by ALK in cancer | 9 | R-HSA-9700206 | 1.14E-03 | RANBP2, NPM1, TPM3, DCTN1, RRBP1, PLCG1, CARS1, KLC1, SKP1 |
| 185 | Signaling by ALK fusions and activated point mutants | 9 | R-HSA-9725370 | 1.14E-03 | RANBP2, NPM1, TPM3, DCTN1, RRBP1, PLCG1, CARS1, KLC1, SKP1 |
| 186 | Cooperation of Prefoldin and TriC/CCT in actin and tubulin folding | 7 | R-HSA-389958 | 1.23E-03 | TUBB2B, TUBB6, TCP1, PFDN2, CCT8, ACTB, TUBA4A |
| 187 | Citric acid cycle (TCA cycle) | 6 | R-HSA-71403 | 1.28E-03 | FH, NNT, MDH2, SUCLG2, SUCLG1, ACO2 |
| 188 | Membrane Trafficking | 41 | R-HSA-199991 | 1.44E-03 | DCTN6, SCARB2, MCFD2, RAB5B, RAB5C, DCTN1, RAB1B, MIA3, COPS7A, KLC1, ACTB, GOLGA2, TUBB6, PPP6R1, MYO6, PPP6R3, VTI1A, KIF21A, KIF1B, RAB6A, AP2M1, SPTBN1, AGFG1, ACTR3, TMED7-TICAM2, GAPVD1, TUBA4A, DNM2, COPS4, TJP1, TUBB2B, COPS5, RAB14, CPD, GOLGB1, CHMP4B, PLIN3, SEC22B, COPS8, PAFAH1B2, RAB7A |
| 189 | Interleukin-12 family signaling | 9 | R-HSA-447115 | 1.45E-03 | HSPA9, HNRNPF, CFL1, TCP1, PDCD4, MIF, SOD2, LMNB1, SOD1 |
| 190 | ISG15 antiviral mechanism | 10 | R-HSA-1169408 | 1.72E-03 | RANBP2, EIF4A1, UBE2N, PIN1, NUP153, FLNB, PLCG1, RAE1, EIF4G3, NUP37 |
| 191 | Cytokine Signaling in Immune system | 45 | R-HSA-1280215 | 2.17E-03 | PSMD10, ITGB1, USP14, EIF4A1, PSMD11, PSMD13, HMGB1, PSMA7, LMNB1, CRKL, PSMD8, PSMB6, PSMB4, PSMD7, PSMB5, PSMD4, PSMB3, CASP3, TRIM2, CFL1, FLNB, PLCG1, RAE1, SKP1, RANBP2, HSPA9, NUP153, MIF, SOD2, SMARCA4, SOD1, PSMA5, PSMA6, PSMC6, PSMC4, HNRNPF, PSMC2, TCP1, PSME1, UBE2N, PDCD4, PIN1, ABCE1, EIF4G3, NUP37 |
| 192 | Nuclear Envelope (NE) Reassembly | 10 | R-HSA-2995410 | 2.30E-03 | TUBB2B, TUBB6, LMNA, RCC1, CHMP4B, LBR, EMD, TUBA4A, LMNB1, NUP37 |
| 193 | Developmental Biology | 65 | R-HSA-1266738 | 2.33E-03 | ITGB1, RPL5, RPL30, RPLP1, RPL10A, ACTB, RPS15, PSMD8, TUBB6, PSMD7, PSMD4, CDH2, CFL1, WDR5, RPLP2, NRCAM, ITGAV, PLXNC1, AP2M1, ACTR3, RPL23, CSNK2A2, RPSA, TUBA4A, DNM2, PSMA5, PSMA6, TUBB2B, TBL1XR1, ZNF638, PSME1, EPHA2, PSMD10, PSMD11, RBM8A, ROCK2, PSMD13, RPL12, MYL12A, PSMA7, PSMB6, SDCBP, PSMB4, PSMB5, RBBP4, POLR2B, PSMB3, RPS2, PLCG1, RPL17, SPTBN1, SPAG9, JUP, DEK, PBX1, SMARCA4, DLG1, RPS28, PSMC6, PSMC4, PSMC2, CTNNB1, CNTN2, RNPS1, RPS21 |
| 194 | ER to Golgi Anterograde Transport | 15 | R-HSA-199977 | 2.52E-03 | DCTN6, MCFD2, DCTN1, RAB1B, TMED7-TICAM2, MIA3, TUBA4A, GOLGA2, TUBB6, TUBB2B, PPP6R1, GOLGB1, PPP6R3, SEC22B, SPTBN1 |
| 195 | Gap junction trafficking and regulation | 8 | R-HSA-157858 | 2.57E-03 | TJP1, TUBB2B, TUBB6, MYO6, AP2M1, ACTB, TUBA4A, DNM2 |
| 196 | SARS-CoV Infections | 29 | R-HSA-9679506 | 2.85E-03 | ITGB1, ROCK2, RPN1, ATP1A1, RPS15, LARP1, TKFC, RBBP4, PCBP2, SAP18, RPS2, HNRNPA1, RAE1, AP2M1, RANBP2, NPM1, RPSA, ATP1B3, NUP153, DDOST, TJP1, RPS28, PSMC6, IMPDH2, UBE2N, CHMP4B, PPIG, RPS21, NUP37 |
| 197 | Glucose metabolism | 11 | R-HSA-70326 | 3.05E-03 | RANBP2, PC, TPI1, GNPDA2, MDH1, MDH2, PGK1, NUP153, RAE1, GAPDH, NUP37 |
| 198 | DNA Damage Recognition in GG-NER | 7 | R-HSA-5696394 | 3.09E-03 | COPS4, COPS5, ACTL6A, RUVBL1, COPS7A, COPS8, ACTB |
| 199 | Apoptotic cleavage of cellular proteins | 7 | R-HSA-111465 | 3.09E-03 | TJP1, DBNL, CASP3, LMNA, ACIN1, CTNNB1, LMNB1 |
| 200 | Apoptotic execution phase | 8 | R-HSA-75153 | 3.62E-03 | TJP1, DBNL, CASP3, LMNA, ACIN1, CTNNB1, HMGB1, LMNB1 |
| 201 | SARS-CoV-1-host interactions | 11 | R-HSA-9692914 | 3.85E-03 | RPS15, NPM1, RPS28, TKFC, PSMC6, PCBP2, RPSA, PPIG, RPS2, HNRNPA1, RPS21 |
| 202 | Protein folding | 11 | R-HSA-391251 | 4.15E-03 | TUBB2B, TUBB6, GNB2, CSNK2A2, TCP1, TBCE, LONP2, PFDN2, CCT8, ACTB, TUBA4A |
| 203 | Vesicle-mediated transport | 41 | R-HSA-5653656 | 4.22E-03 | DCTN6, SCARB2, MCFD2, RAB5B, RAB5C, DCTN1, RAB1B, MIA3, COPS7A, KLC1, ACTB, GOLGA2, TUBB6, PPP6R1, MYO6, PPP6R3, VTI1A, KIF21A, KIF1B, RAB6A, AP2M1, SPTBN1, AGFG1, ACTR3, TMED7-TICAM2, GAPVD1, TUBA4A, DNM2, COPS4, TJP1, TUBB2B, COPS5, RAB14, CPD, GOLGB1, CHMP4B, PLIN3, SEC22B, COPS8, PAFAH1B2, RAB7A |
| 204 | Protein localization | 15 | R-HSA-9609507 | 4.46E-03 | HSPA9, TIMM8B, PEX19, IDH1, ECI2, HMGCL, SAMM50, PEX3, CAT, AGPS, LONP2, PMPCA, ACO2, ACAA1, EMD |
| 205 | Transport of Mature mRNA Derived from an Intronless Transcript | 7 | R-HSA-159231 | 4.56E-03 | RANBP2, NXF1, FIP1L1, ALYREF, NUP153, RAE1, NUP37 |
| 206 | Signaling by WNT | 24 | R-HSA-195721 | 4.66E-03 | PSMD10, PSMD11, PSMD13, CSNK2A2, PSMA7, SMARCA4, PSMD8, PSMA5, PSMB6, PSMA6, PSMB4, PSMC6, PSMD7, PSMB5, PSMD4, PSMC4, PSMB3, GNB2, PSMC2, RUVBL1, PSME1, CTNNB1, AP2M1, SKP1 |
| 207 | Metabolism of nucleotides | 11 | R-HSA-15869 | 4.80E-03 | NME1-NME2, ADSL, GMPR2, IMPDH2, GSR, HPRT1, UMPS, NUDT5, DNPH1, ITPA, DHODH |
| 208 | Global Genome Nucleotide Excision Repair (GG-NER) | 10 | R-HSA-5696399 | 4.97E-03 | COPS4, COPS5, ERCC3, POLD2, ACTL6A, RUVBL1, UBE2N, COPS7A, COPS8, ACTB |
| 209 | Pyruvate metabolism and Citric Acid (TCA) cycle | 8 | R-HSA-71406 | 4.98E-03 | FH, PDHA1, NNT, MDH2, SUCLG2, SUCLG1, ACO2, PDHB |
| 210 | Transport of Mature mRNAs Derived from Intronless Transcripts | 7 | R-HSA-159234 | 5.16E-03 | RANBP2, NXF1, FIP1L1, ALYREF, NUP153, RAE1, NUP37 |
| 211 | Golgi-to-ER retrograde transport | 13 | R-HSA-8856688 | 5.30E-03 | DCTN6, DCTN1, RAB1B, TMED7-TICAM2, KLC1, TUBA4A, TUBB6, TUBB2B, KIF21A, KIF1B, RAB6A, SEC22B, PAFAH1B2 |
| 212 | Post-translational protein modification | 75 | R-HSA-597592 | 5.49E-03 | MCFD2, MPI, MIA3, ACTB, PSMD8, GOLGA2, TUBB6, PSMD7, TRIM28, PSMD4, CDH2, PPP6R1, RUVBL1, PPP6R3, WDR5, PGM3, UBXN7, SKP1, ACTL6A, SMC1A, RNF40, TUBA4A, DDOST, PSMA5, PSMA6, RCN1, TUBB2B, SKIC8, PTRH2, PSME1, SEC22B, RAB7A, DCTN6, PSMD10, USP14, RAB5B, PSMD11, RAB5C, UBA6, PRKDC, PSMD13, DCTN1, RAB1B, RPN1, COPS7A, PSMA7, PSMB6, PSMB4, PSMB5, PSMB3, MLEC, RPS2, RAB6A, RAE1, UGGT1, SPTBN1, RANBP2, NPM1, TMED7-TICAM2, NUP153, KTN1, COPS4, PSMC6, COPS5, RAB14, PSMC4, GOLGB1, SP3, PSMC2, UBE2N, CALU, PAF1, HNRNPC, COPS8, NUP37 |
| 213 | Immune System | 100 | R-HSA-168256 | 5.65E-03 | EIF4A1, AHCYL1, CD81, ACTB, CRKL, PSMD8, TUBB6, PSMD7, PSMD4, CFL1, CPNE3, ATP6V1E1, ACAA1, AP2M1, SKP1, MIF, TUBA4A, ACLY, PSME1, ANAPC1, PSMD10, RAB5B, PSMD11, RAB5C, PSMD13, UBA6, PRKDC, UBA5, KLC1, NME1-NME2, SDCBP, TKFC, PSAP, PCBP2, MLEC, PLCG1, RAB6A, HSPA9, JUP, HSPA5, IDH1, NUP153, PRDX6, SMARCA4, HNRNPF, UBE2N, PIN1, BLMH, EIF4G3, LGMN, ITGB1, LPCAT1, HMGB1, LMNB1, TRIM2, CASP3, ITGAV, LRRFIP1, ACTR3, DBNL, GAA, ATP6AP2, DDOST, DNM2, PSMA5, PSMA6, TUBB2B, TCP1, CAT, SEC22B, PAFAH1B2, RAB7A, DCTN6, USP14, ASAH1, DCTN1, MGRN1, PSMA7, PSMB6, PSMB4, PSMB5, PSMB3, FLNB, RBBP6, CCT8, RAE1, RANBP2, TMED7-TICAM2, SOD2, SOD1, ERP44, PSMC6, RAB14, PSMC4, IMPDH2, PSMC2, PDCD4, CTNNB1, ABCE1, NUP37 |
| 214 | Deubiquitination | 22 | R-HSA-5688426 | 5.76E-03 | PSMD10, USP14, PSMD11, PSMD13, ACTL6A, PSMA7, ACTB, PSMD8, PSMA5, PSMB6, PSMA6, PSMB4, PSMC6, PSMD7, PSMB5, PSMD4, PSMC4, PSMB3, PTRH2, PSMC2, RUVBL1, PSME1 |
| 215 | RAF/MAP kinase cascade | 21 | R-HSA-5673001 | 6.04E-03 | PSMD10, PSMD11, PSMD13, PEBP1, PSMA7, ACTB, PSMD8, PSMA5, PSMB6, PSMA6, DLG1, PSMB4, PSMC6, PSMD7, PSMB5, PSMD4, PSMC4, PSMB3, PSMC2, PSME1, SPTBN1 |
| 216 | Glyoxylate metabolism and glycine degradation | 6 | R-HSA-389661 | 6.21E-03 | ALDH4A1, GRHPR, PDHA1, GLDC, DBT, PDHB |
| 217 | RHO GTPases activate IQGAPs | 6 | R-HSA-5626467 | 7.13E-03 | TUBB2B, TUBB6, CLIP1, CTNNB1, ACTB, TUBA4A |
| 218 | mRNA 3'-end processing | 8 | R-HSA-72187 | 7.34E-03 | CPSF7, RBM8A, FIP1L1, SARNP, ALYREF, SRSF3, RNPS1, SRRM1 |
| 219 | MAPK1/MAPK3 signaling | 21 | R-HSA-5684996 | 7.60E-03 | PSMD10, PSMD11, PSMD13, PEBP1, PSMA7, ACTB, PSMD8, PSMA5, PSMB6, PSMA6, DLG1, PSMB4, PSMC6, PSMD7, PSMB5, PSMD4, PSMC4, PSMB3, PSMC2, PSME1, SPTBN1 |
| 220 | Nuclear import of Rev protein | 6 | R-HSA-180746 | 8.14E-03 | RANBP2, NPM1, RCC1, NUP153, RAE1, NUP37 |
| 221 | Chaperonin-mediated protein folding | 10 | R-HSA-390466 | 8.36E-03 | TUBB2B, TUBB6, GNB2, CSNK2A2, TCP1, LONP2, PFDN2, CCT8, ACTB, TUBA4A |
| 222 | Gap junction trafficking | 7 | R-HSA-190828 | 8.98E-03 | TUBB2B, TUBB6, MYO6, AP2M1, ACTB, TUBA4A, DNM2 |
| 223 | Transport of the SLBP independent Mature mRNA | 6 | R-HSA-159227 | 9.25E-03 | RANBP2, NXF1, ALYREF, NUP153, RAE1, NUP37 |
| 224 | Gap junction degradation | 4 | R-HSA-190873 | 9.69E-03 | MYO6, AP2M1, ACTB, DNM2 |
| 225 | Transport of the SLBP Dependant Mature mRNA | 6 | R-HSA-159230 | 1.05E-02 | RANBP2, NXF1, ALYREF, NUP153, RAE1, NUP37 |
| 226 | Gluconeogenesis | 6 | R-HSA-70263 | 1.05E-02 | PC, TPI1, MDH1, MDH2, PGK1, GAPDH |
| 227 | Nucleotide Excision Repair | 11 | R-HSA-5696398 | 1.06E-02 | COPS4, COPS5, ERCC3, POLR2B, POLD2, ACTL6A, RUVBL1, UBE2N, COPS7A, COPS8, ACTB |
| 228 | Clathrin-mediated endocytosis | 13 | R-HSA-8856828 | 1.09E-02 | ACTR3, SCARB2, RAB5B, RAB5C, COPS7A, GAPVD1, ACTB, DNM2, COPS4, COPS5, COPS8, AP2M1, AGFG1 |
| 229 | Platelet degranulation | 12 | R-HSA-114608 | 1.12E-02 | LGALS3BP, TMX3, HSPA5, TEX264, ACTN1, CFL1, PSAP, CALU, ACTN4, FAM3C, TUBA4A, SOD1 |
| 230 | Interactions of Vpr with host cellular proteins | 6 | R-HSA-176033 | 1.18E-02 | RANBP2, PSIP1, NUP153, SLC25A5, RAE1, NUP37 |
| 231 | Interactions of Rev with host cellular proteins | 6 | R-HSA-177243 | 1.18E-02 | RANBP2, NPM1, RCC1, NUP153, RAE1, NUP37 |
| 232 | Transport to the Golgi and subsequent modification | 15 | R-HSA-948021 | 1.25E-02 | DCTN6, MCFD2, DCTN1, RAB1B, TMED7-TICAM2, MIA3, TUBA4A, GOLGA2, TUBB6, TUBB2B, PPP6R1, GOLGB1, PPP6R3, SEC22B, SPTBN1 |
| 233 | Cytosolic tRNA aminoacylation | 5 | R-HSA-379716 | 1.26E-02 | AIMP1, EPRS1, FARSA, CARS1, YARS1 |
| 234 | HIV Life Cycle | 13 | R-HSA-162587 | 1.27E-02 | RANBP2, GTF2A1, FEN1, PSIP1, NUP153, GTF2F2, ERCC3, POLR2B, CHMP4B, RCC1, NELFB, RAE1, NUP37 |
| 235 | SARS-CoV-1 modulates host translation machinery | 6 | R-HSA-9735869 | 1.32E-02 | RPS15, RPS28, RPSA, RPS2, HNRNPA1, RPS21 |
| 236 | Detoxification of Reactive Oxygen Species | 6 | R-HSA-3299685 | 1.32E-02 | PRDX3, GSR, CAT, SOD2, PRDX6, SOD1 |
| 237 | COPI-independent Golgi-to-ER retrograde traffic | 7 | R-HSA-6811436 | 1.32E-02 | DCTN6, TUBB2B, TUBB6, DCTN1, RAB6A, PAFAH1B2, TUBA4A |
| 238 | Regulation of CDH19 Expression and Function | 3 | R-HSA-9764302 | 1.38E-02 | JUP, CTNND1, CTNNB1 |
| 239 | Adaptive Immune System | 43 | R-HSA-1280218 | 1.39E-02 | PSMD10, ITGB1, DCTN6, AHCYL1, PSMD11, UBA6, CD81, PSMD13, DCTN1, MGRN1, UBA5, HMGB1, KLC1, PSMA7, PSMD8, PSMB6, TUBB6, PSMB4, PSMD7, PSMB5, PSMD4, PSMB3, ITGAV, RBBP6, PLCG1, AP2M1, SKP1, HSPA5, TUBA4A, DNM2, PSMA5, PSMA6, TUBB2B, PSMC6, PSMC4, PSMC2, PSME1, UBE2N, BLMH, SEC22B, ANAPC1, RAB7A, LGMN |
| 240 | Asparagine N-linked glycosylation | 21 | R-HSA-446203 | 1.42E-02 | DCTN6, MCFD2, DCTN1, RAB1B, RPN1, TMED7-TICAM2, MPI, MIA3, TUBA4A, DDOST, GOLGA2, TUBB6, TUBB2B, PPP6R1, GOLGB1, PPP6R3, PGM3, MLEC, SEC22B, UGGT1, SPTBN1 |
| 241 | Nuclear Envelope Breakdown | 7 | R-HSA-2980766 | 1.45E-02 | RANBP2, LMNA, NUP153, RAE1, EMD, LMNB1, NUP37 |
| 242 | Defective Intrinsic Pathway for Apoptosis | 5 | R-HSA-9734009 | 1.46E-02 | GOLGA2, C1QBP, LMNA, SOD2, LMNB1 |
| 243 | Formation of tubulin folding intermediates by CCT/TriC | 5 | R-HSA-389960 | 1.46E-02 | TUBB2B, TUBB6, TCP1, CCT8, TUBA4A |
| 244 | Response to elevated platelet cytosolic Ca2+ | 12 | R-HSA-76005 | 1.46E-02 | LGALS3BP, TMX3, HSPA5, TEX264, ACTN1, CFL1, PSAP, CALU, ACTN4, FAM3C, TUBA4A, SOD1 |
| 245 | Nucleotide biosynthesis | 4 | R-HSA-8956320 | 1.51E-02 | ADSL, IMPDH2, UMPS, DHODH |
| 246 | RNA Polymerase II Transcription Termination | 8 | R-HSA-73856 | 1.55E-02 | CPSF7, RBM8A, FIP1L1, SARNP, ALYREF, SRSF3, RNPS1, SRRM1 |
| 247 | COPI-mediated anterograde transport | 10 | R-HSA-6807878 | 1.59E-02 | GOLGA2, DCTN6, TUBB2B, TUBB6, DCTN1, GOLGB1, RAB1B, TMED7-TICAM2, SPTBN1, TUBA4A |
| 248 | PIP3 activates AKT signaling | 19 | R-HSA-1257604 | 1.62E-02 | PSMD10, PSMD11, PSMD13, CSNK2A2, PSMA7, PSMD8, PSMA5, PSMB6, PSMA6, PSMB4, PSMC6, PSMD7, PSMB5, RBBP4, PSMD4, PSMC4, PSMB3, PSMC2, PSME1 |
| 249 | FGFR2 alternative splicing | 5 | R-HSA-6803529 | 1.67E-02 | TIA1, POLR2B, HNRNPF, HNRNPA1, GTF2F2 |
| 250 | Intracellular signaling by second messengers | 21 | R-HSA-9006925 | 1.73E-02 | PSMD10, AHCYL1, PSMD11, PSMD13, CSNK2A2, PSMA7, PSMD8, PSMA5, PSMB6, PSMA6, PSMB4, PSMC6, PSMD7, PSMB5, RBBP4, PSMD4, PSMC4, PSMB3, PSMC2, PSME1, PLCG1 |
| 251 | SARS-CoV-1 Infection | 12 | R-HSA-9678108 | 2.15E-02 | RPS15, NPM1, RPS28, TKFC, PSMC6, PCBP2, CHMP4B, RPSA, PPIG, RPS2, HNRNPA1, RPS21 |
| 252 | tRNA Aminoacylation | 6 | R-HSA-379724 | 2.20E-02 | AIMP1, SARS2, EPRS1, FARSA, CARS1, YARS1 |
| 253 | tRNA processing in the nucleus | 7 | R-HSA-6784531 | 2.20E-02 | RANBP2, POP1, ELAC2, RTCB, NUP153, RAE1, NUP37 |
| 254 | Glycolysis | 8 | R-HSA-70171 | 2.22E-02 | RANBP2, TPI1, GNPDA2, PGK1, NUP153, RAE1, GAPDH, NUP37 |
| 255 | Transport of small molecules | 40 | R-HSA-382551 | 2.31E-02 | PSMD10, STEAP3, PSMD11, TTYH3, ABCB7, PSMD13, HDLBP, ATP2A2, ATP1A1, SPG7, ADD3, PSMA7, ADD2, PSMD8, PSMB6, PSMB4, PSMD7, PSMB5, PSMD4, PSMB3, PMPCA, ATP6V1E1, SLC12A7, AP2M1, SKP1, PEX19, ATP1B3, LETM1, PSMA5, PSMA6, PSMC6, EIF2S3, ASPH, PSMC4, PEX3, GNB2, PSMC2, PSME1, SLC25A5, MCU |
| 256 | Viral Messenger RNA Synthesis | 6 | R-HSA-168325 | 2.41E-02 | RANBP2, POLR2B, NUP153, RAE1, GTF2F2, NUP37 |
| 257 | Cell-Cell communication | 12 | R-HSA-1500931 | 2.47E-02 | ITGB1, JUP, CDH2, CADM1, ACTN1, CTNND1, CTNNB1, ITGA6, ACTN4, SPTBN1, ACTB, CD2AP |
| 258 | Intra-Golgi and retrograde Golgi-to-ER traffic | 15 | R-HSA-6811442 | 2.48E-02 | DCTN6, DCTN1, RAB1B, TMED7-TICAM2, KLC1, TUBA4A, TUBB6, TUBB2B, VTI1A, PLIN3, KIF21A, KIF1B, RAB6A, SEC22B, PAFAH1B2 |
| 259 | MAPK family signaling cascades | 21 | R-HSA-5683057 | 2.70E-02 | PSMD10, PSMD11, PSMD13, PEBP1, PSMA7, ACTB, PSMD8, PSMA5, PSMB6, PSMA6, DLG1, PSMB4, PSMC6, PSMD7, PSMB5, PSMD4, PSMC4, PSMB3, PSMC2, PSME1, SPTBN1 |
| 260 | NrCAM interactions | 3 | R-HSA-447038 | 2.75E-02 | DLG1, CNTN2, NRCAM |
| 261 | CDH11 homotypic and heterotypic interactions | 3 | R-HSA-9833576 | 2.75E-02 | JUP, CTNND1, CTNNB1 |
| 262 | Unfolded Protein Response (UPR) | 9 | R-HSA-381119 | 2.86E-02 | EXOSC6, ACADVL, EIF2S3, HSPA5, DCTN1, LMNA, DNAJB11, SSR1, EXOSC2 |
| 263 | DNA strand elongation | 5 | R-HSA-69190 | 3.35E-02 | POLA1, FEN1, MCM7, POLD2, MCM6 |
| 264 | Recycling pathway of L1 | 6 | R-HSA-437239 | 3.40E-02 | TUBB2B, TUBB6, AP2M1, ACTB, TUBA4A, DNM2 |
| 265 | Peroxisomal protein import | 7 | R-HSA-9033241 | 3.41E-02 | HMGCL, IDH1, ECI2, AGPS, CAT, LONP2, ACAA1 |
| 266 | Initiation of Nuclear Envelope (NE) Reformation | 4 | R-HSA-2995383 | 3.50E-02 | LMNA, LBR, EMD, LMNB1 |
| 267 | HCMV Early Events | 11 | R-HSA-9609690 | 3.61E-02 | ITGB1, RANBP2, TUBB2B, TUBB6, RBBP4, TRIM28, TBL1XR1, NUP153, RAE1, TUBA4A, NUP37 |
| 268 | Vpr-mediated nuclear import of PICs | 5 | R-HSA-180910 | 3.70E-02 | RANBP2, PSIP1, NUP153, RAE1, NUP37 |
| 269 | COPI-dependent Golgi-to-ER retrograde traffic | 9 | R-HSA-6811434 | 3.73E-02 | TUBB2B, TUBB6, RAB1B, TMED7-TICAM2, KIF21A, KIF1B, SEC22B, KLC1, TUBA4A |
| 270 | Late Phase of HIV Life Cycle | 11 | R-HSA-162599 | 3.77E-02 | RANBP2, GTF2A1, ERCC3, POLR2B, RCC1, CHMP4B, NELFB, NUP153, RAE1, GTF2F2, NUP37 |
| 271 | Adherens junctions interactions | 6 | R-HSA-418990 | 3.97E-02 | JUP, CDH2, CADM1, CTNND1, CTNNB1, ACTB |
| 272 | Rev-mediated nuclear export of HIV RNA | 5 | R-HSA-165054 | 4.07E-02 | RANBP2, RCC1, NUP153, RAE1, NUP37 |
| 273 | mRNA Splicing - Minor Pathway | 6 | R-HSA-72165 | 4.28E-02 | SF3B2, POLR2B, SF3B6, DDX42, GTF2F2, SF3B1 |
| 274 | Signaling by BRAF and RAF1 fusions | 7 | R-HSA-6802952 | 4.41E-02 | FXR1, AGK, FAM114A2, LMNA, PEBP1, QKI, ACTB |
| 275 | RHO GTPases Activate Formins | 11 | R-HSA-5663220 | 4.45E-02 | ITGB1, RANBP2, TUBB2B, TUBB6, CLIP1, BUB3, MAPRE1, CKAP5, ACTB, TUBA4A, NUP37 |
| 276 | HCMV Infection | 12 | R-HSA-9609646 | 4.57E-02 | ITGB1, RANBP2, TUBB2B, TUBB6, RBBP4, TRIM28, TBL1XR1, CHMP4B, NUP153, RAE1, TUBA4A, NUP37 |
| 277 | Extension of Telomeres | 6 | R-HSA-180786 | 4.60E-02 | POLA1, FEN1, POLD2, PPP6R3, RUVBL1, NHP2 |
| 278 | MHC class II antigen presentation | 10 | R-HSA-2132295 | 4.84E-02 | DCTN6, TUBB2B, TUBB6, DCTN1, KLC1, AP2M1, TUBA4A, RAB7A, LGMN, DNM2 |
| 279 | Gene expression (Transcription) | 73 | R-HSA-74160 | 4.89E-02 | STEAP3, PSIP1, ACTB, PSMD8, PSMD7, TRIM28, PSMD4, WDR5, NELFB, LBR, SKP1, ELAC2, CSNK2A2, ACTL6A, BAZ1B, GTF2F2, TSN, SRRM1, PSMA5, PSMA6, SKIC8, POLR1A, WDR82, TBL1XR1, SARNP, CAT, PSME1, SRSF3, ANAPC1, GTF3C1, PSMD10, GTF2A1, SMARCD1, PSMD11, RBM8A, UHRF1, PSMD13, SRRT, NOP2, COX5A, PSMA7, PSMB6, PSMB4, PSMB5, RBBP4, FIP1L1, POLR2B, PSMB3, SAP18, SF3B1, RAE1, RANBP2, NPM1, TIGAR, CPSF7, ALYREF, GSR, NUP153, RPRD2, DEK, SOD2, SMARCA4, TJP1, TP53I3, PSMC6, ERCC3, PSMC4, PSMC2, PIN1, CTNNB1, PAF1, RNPS1, NUP37 |
| 280 | IRE1alpha activates chaperones | 6 | R-HSA-381070 | 4.93E-02 | ACADVL, HSPA5, DCTN1, LMNA, DNAJB11, SSR1 |

**Supplementary Table S7:** List of all identified Molecular functions with their protein count and protein involved particular biological processes, is analysed by Database for Annotation, Visualization and Integrated Discovery (DAVID) platform with a significant *p*-value ≤ 0.05 of differentially expressed proteins (up-regulated and down-regulated) identified by the high-resolution mass spectrometry (HRMS).
