# Supplementary material for: A protein–miRNA biomic analysis approach to explore neuroprotective potential of nobiletin in human neural progenitor cells (hNPCs)
Source: Front Pharmacol. 2024 Jan 25;15:1343569. doi: 10.3389/fphar.2024.1343569 (PMC10860404; doi:10.3389/fphar.2024.1343569)
Supplement: Supplementary file 4 [file Table9.DOCX]

**Supplementary Table S10**

| **S. No.** | **Gene Name** | **Forward Primer** | **Forward Primer** |
| --- | --- | --- | --- |
| 1 | Apaf1 | GGCTGTGGGAAGTCTGTATTGA | CAACCGTGTGCAAAGATTCTG |
| 2 | Cas3 | ACTGGACTGTGGCATTGAG | GAGCCATCCTTTGAATTTCGC |
| 3 | SQSTM1 | AATCAGCTTCTGGTCCATCG | TTCTTTTCCCTCCGTGCTC |
| 4 | LC3B | AAGTTCCTTGTACCTGACCATG | CTGAGATTGGTGTGGAGACG |
| 5 | HSP70 | AGGACATCAGCCAGAACAAG | CTGGTGATGGACGTGTAGAAG |
| 6 | HMOX1 | TCAGGCAGAGGGTGATAGAAG | TTGGTGTCATGGGTCAGC |
| 7 | SNCA1 | GACCAGTTGGGCAAGAATGAA | CACAGGCATATCTTCCAGAAT |
| 8 | ACTB | CACCATTGGCAATGAGCGGTTC | AGGTCTTTGCGGATGTCCACGT |

**Supplementary Table S10:** List of primers used in RT PCR and their sequences.
