# Supplementary material for: A protein–miRNA biomic analysis approach to explore neuroprotective potential of nobiletin in human neural progenitor cells (hNPCs)
Source: Front Pharmacol. 2024 Jan 25;15:1343569. doi: 10.3389/fphar.2024.1343569 (PMC10860404; doi:10.3389/fphar.2024.1343569)
Supplement: Supplementary file 5 [file Table2.DOCX]

**Supplementary Table S2**

**A. Mitochondrial and Oxidative Stress**

| **S. No** | **Accession** | **Description** | **Gene symbol** | **Abundance Ratio (log_2_): (NA) / (CON)** | **Abundance Ratio (log_2_): (NA_NOB) / (CON)** | **Abundance Ratio (log_2_):**  **(NA_NOB) / (NA)** |
| --- | --- | --- | --- | --- | --- | --- |
|  | A0A087X2D5 | 39S ribosomal protein L45, mitochondrial | MRPL45 | -6.13 | -6.64 | -0.94 |
|  | I3NI32 | Dihydroorotate dehydrogenase (quinone), mitochondrial | DHODH | -5.86 | -4.67 | 1.24 |
|  | A0A096LNH5 | Glutamine amidotransferase-like class 1 domain-containing protein 3B, mitochondrial | GATD3B | -5.18 | -4.96 | 0.13 |
|  | Q13268 | Dehydrogenase/reductase SDR family member 2, mitochondrial | DHRS2 | -4.94 | -2.18 | 2.77 |
|  | Q8IXI1 | Mitochondrial Rho GTPase 2 | RHOT2 | -4.94 | -3.23 | 2.28 |
|  | Q6IAL5 | Succinate--CoA ligase ADP/GDP-forming] subunit alpha, mitochondrial | SUCLG1 | -4.85 | -4.43 | 0.13 |
|  | P30048 | Thioredoxin-dependent peroxide reductase, mitochondrial | PRDX3 | -4.35 | -4.01 | 0.58 |
|  | A0A087WYV9 | Cytochrome c oxidase assembly protein COX16 homolog, mitochondrial | SYNJ2BP-COX16 | -4.12 | -3.69 | -0.22 |
|  | Q10713 | Mitochondrial-processing peptidase subunit alpha | PMPCA | -3.95 | -3.39 | 0.56 |
|  | Q567R6 | Single-stranded DNA-binding protein, mitochondrial | SSBP1 | -3.91 | -3.38 | 1.08 |
|  | A0A0A0MS29 | Mitochondrial fission factor | MFF | -3.89 | -3.86 | 0.04 |
|  | B4DYN5 | Succinate dehydrogenase ubiquinone flavoprotein subunit, mitochondrial | SDHA | -3.78 | -3.06 | 0.72 |
|  | Q07021 | Complement component 1 Q subcomponent-binding protein, mitochondrial | C1QBP | -3.65 | -3.77 | -0.12 |
|  | B4DJ81 | NADH-ubiquinone oxidoreductase 75 kDa subunit, mitochondrial | NDUFS1 | -3.6 | -3.64 | -0.04 |
|  | Q16698 | 2,4-dienoyl-CoA reductase (3E)-enoyl-CoA-producing], mitochondrial | DECR1 | -3.57 | -3.02 | 0.55 |
|  | P30038 | Delta-1-pyrroline-5-carboxylate dehydrogenase, mitochondrial | ALDH4A1 | -3.53 | -3.36 | 0.17 |
|  | P11182 | Lipoamide acyltransferase component of branched-chain alpha-keto acid dehydrogenase complex, mitochondrial | DBT | -3.51 | -2.94 | 0.66 |
|  | Q53FB6 | Mitochondrial aldehyde dehydrogenase 2 variant (Fragment) | None | -3.48 | -3.77 | -0.29 |
|  | P08559 | Pyruvate dehydrogenase E1 component subunit alpha, somatic form, mitochondrial | PDHA1 | -3.45 | -2.88 | 0.58 |
|  | H0YEW4 | ATP synthase mitochondrial F1 complex assembly factor 1 (Fragment) | ATPAF1 | -3.43 | -1.57 | 1.85 |
|  | E9PDQ8 | Succinate--CoA ligase GDP-forming] subunit beta, mitochondrial | SUCLG2 | -3.37 | -1.12 | 2.25 |
|  | Q99798 | Aconitate hydratase, mitochondrial | ACO2 | -3.34 | -2.63 | 0.72 |
|  | P13804 | Electron transfer flavoprotein subunit alpha, mitochondrial | ETFA | -3.28 | -2.72 | 0.57 |
|  | P20674 | Cytochrome c oxidase subunit 5A, mitochondrial | COX5A | -3.23 | -3.26 | -0.03 |
|  | Q9Y5J9 | Mitochondrial import inner membrane translocase subunit Tim8 B | TIMM8B | -3.21 | -1.98 | 1.23 |
|  | Q6DKK2 | Tetratricopeptide repeat protein 19, mitochondrial | TTC19 | -3.19 | -1.93 | 1.26 |
|  | Q7RU05 | Mitochondrial import inner membrane translocase subunit TIM17 | TIM17A | -3.1 | -2.26 | 0.84 |
|  | E9PC15 | Acylglycerol kinase, mitochondrial | AGK | -3.09 | -2.57 | 0.53 |
|  | P23378 | Glycine dehydrogenase (decarboxylating), mitochondrial | GLDC | -3.05 | -2.88 | 0.17 |
|  | P07954 | Fumarate hydratase, mitochondrial | FH | -3.04 | -2.74 | 0.3 |
|  | Q99797 | Mitochondrial intermediate peptidase | MIPEP | -3.03 | -2.36 | 0.67 |
|  | V5IRT4 | Mitochondrial nucleoid factor 1 (Fragment) | UQCC2 | -2.96 | -3.46 | -0.5 |
|  | A0A0C4DGA2 | Enoyl-CoA delta isomerase 2, mitochondrial | ECI2 | -2.78 | -3 | -0.22 |
|  | B2RBJ8 | Glutamyl-tRNA(Gln) amidotransferase subunit A, mitochondrial | QRSL1 | -2.75 | -1.55 | 1.2 |
|  | P00390 | Glutathione reductase, mitochondrial | GSR | -2.76 | -2.64 | 0.12 |
|  | H3BUY0 | 39S ribosomal protein L21, mitochondrial | MRPL21 | -2.7 | -1.55 | 1.14 |
|  | P31930 | Cytochrome b-c1 complex subunit 1, mitochondrial | UQCRC1 | -2.65 | -2.8 | -0.15 |
|  | P51398 | 28S ribosomal protein S29, mitochondrial | DAP3 | -2.5 | -2.36 | 0.14 |
|  | A0A024RD08 | Mitochondrial carrier homolog 1 (C. elegans), isoform | MTCH1 | -2.46 | -1.33 | 1.13 |
|  | Q9UKU7 | Isobutyryl-CoA dehydrogenase, mitochondrial | ACAD8 | -2.46 | -1.55 | 0.92 |
|  | P11498 | Pyruvate carboxylase, mitochondrial | PC | -2.46 | -2.73 | -0.27 |
|  | A0A024R850 | Mitochondrial ribosome recycling factor, isoform | MRRF | -2.45 | -1.12 | 1.32 |
|  | Q9NQ50 | 39S ribosomal protein L40, mitochondrial | MRPL40 | -2.45 | -2.97 | -0.52 |
|  | F5GZQ3 | Trifunctional enzyme subunit beta, mitochondrial | HADHB | -2.45 | -2.09 | 0.36 |
|  | A0A0C4DGN7 | NADPH:adrenodoxin oxidoreductase, mitochondrial | FDXR | -2.41 | -2.22 | 0.19 |
|  | A0A024RCA6 | Solute carrier family 25 (mitochondrial carrier: glutamate), member 22, isoform | None | -2.36 | -1.05 | 1.31 |
|  | A0A087WW65 | ATP-binding cassette sub-family B member 7, mitochondrial | ABCB7 | -2.35 | -0.87 | 1.48 |
|  | Q96DI8 | Heme oxygenase | HMOX1 | 5.99 | 0.78 | -5.89 |
|  | Q5T440 | Putative transferase CAF17, mitochondrial | IBA57 | 3.55 | 2.31 | -1.25 |
|  | E7ESZ7 | NADH dehydrogenase [ubiquinone] 1 alpha subcomplex subunit 10, mitochondrial | NDUFA10 | 3.51 | 3.2 | -0.31 |
|  | Q53GN7 | Mitochondrial ribosomal protein S30 variant (Fragment) | MRPS30 | 2.74 | 2.96 | 0.22 |
|  | D6REA0 | Glutamyl-tRNA(Gln) amidotransferase subunit B, mitochondrial | GATB | 2.62 | 2.58 | -0.04 |
|  | A0A384MDW7 | Enoyl Coenzyme A hydratase, short chain, 1, mitochondrial | ECHS1 | 2.52 | 2.31 | -0.21 |
|  | Q13084 | 39S ribosomal protein L28, mitochondrial | MRPL28 | 2.33 | 2.61 | 0.29 |

**B. Ubiquitin proteasome system, autophagy, chaperons**

| **S. No.** | **Accession** | **Description** | **Gene Symbol** | **log_2_ Fold change: (NA) / (CON)** | **log_2_ Fold change: (NA_NOB) / (CON)** | **log_2_ Fold change: (NA_NOB) / (NA)** |
| --- | --- | --- | --- | --- | --- | --- |
|  | K7EPJ5 | E3 ubiquitin-protein ligase MGRN1 | MGRN1 | -5.18 | -4.47 | 0.69 |
|  | Q8WVY7 | Ubiquitin-like domain-containing CTD phosphatase 1 | UBLCP1 | -3.94 | -2.97 | 1.68 |
|  | D3DUG9 | Ubiquitin carboxyl-terminal hydrolase | USP14 | -3.66 | -1.93 | 1.74 |
|  | Q8TF42 | ubiquitin-associated and SH3 domain-containing protein B | UBASH3B | -3.64 | -3.14 | 0.5 |
|  | A0A087WTW0 | RING-type E3 ubiquitin transferase | UHRF1 | -3.6 | -3.64 | -0.04 |
|  | A0A0S2Z537 | E3 ubiquitin protein ligase | RNF40 | -2.91 | -5.25 | -2.34 |
|  | P61088 | Ubiquitin-conjugating enzyme E2 N | UBE2N | -2.7 | -2.3 | 0.4 |
|  | D3DW56 | Ubiquitin-like 7 (Bone marrow stromal cell-derived) | UBL7 | -2.65 | -2.8 | -0.15 |
|  | Q9GZZ9 | Ubiquitin-like modifier-activating enzyme 5 | UBA5 | -2.59 | -3.99 | -1.4 |
|  | H3BSK8 | E3 ubiquitin-protein ligase RBBP6 | RBBP6 | -2.38 | -3.37 | -1 |
|  | A0AVT1 | Ubiquitin-like modifier-activating enzyme 6 | UBA6 | -2.32 | -2.45 | -0.13 |
|  | A0A1W2PRF6 | Lysosome membrane protein 2 | SCARB2 | -2.73 | -1.25 | 1.48 |
|  | Q9ULT8 | E3 ubiquitin-protein ligase HECTD1 | HECTD1 | 4.28 | 3.13 | -1.15 |
|  | B2RB57 | Ubiquitin-like modifier-activating enzyme | ATG7 | 2.8 | 3.6 | 0.8 |

**C. Nuclear transport**

| **S. No.** | **Accession** | **Description** | **Gene Symbol** | **log_2_ Fold change: (NA) / (CON)** | **log_2_ Fold change: (NA_NOB) / (CON)** | **log_2_ Fold change: (NA_NOB) / (NA)** |
| --- | --- | --- | --- | --- | --- | --- |
|  | H0Y8G5 | Heterogeneous nuclear ribonucleoprotein D0 (Fragment) | HNRNPD | -5.19 | -4.37 | 0.58 |
|  | Q12972 | Nuclear inhibitor of protein phosphatase 1 | PPP1R8 | -5.15 | -4.38 | 0.77 |
|  | B5BTZ8 | Small nuclear ribonucleoprotein polypeptide B'' | SNRPB2 | -4.93 | -4.02 | 0.91 |
|  | D6RBZ0 | Heterogeneous nuclear ribonucleoprotein A/B | HNRNPAB | -4.86 | -3.35 | 1.51 |
|  | B4DDB6 | Heterogeneous nuclear ribonucleoprotein A3, isoform | HNRNPA3 | -4.78 | -3.92 | 1.18 |
|  | H3BRV9 | Nuclear transport factor 2 (Fragment) | NUTF2 | -4.28 | -5.44 | -1.15 |
|  | Q53FW7 | GA binding protein transcription factor, alpha subunit (60kD) variant (Fragment) | GABPA | -3.9 | -2.85 | 1.05 |
|  | B4DY08 | Heterogeneous nuclear ribonucleoproteins C1/C2 | HNRNPC | -3.34 | -2.17 | 1.17 |
|  | P52597 | Heterogeneous nuclear ribonucleoprotein F | HNRNPF | -3.33 | -2.76 | 0.57 |
|  | Q13151 | Heterogeneous nuclear ribonucleoprotein A0 | HNRNPA0 | -3.22 | -4.81 | -1.59 |
|  | Q12789 | General transcription factor 3C polypeptide 1 | GTF3C1 | -3.13 | -2.46 | 0.67 |
|  | A0A2R8YDA1 | Transcription activator BRG1 (Fragment) | SMARCA4 | -3.05 | -2.88 | 0.17 |
|  | P13984 | General transcription factor IIF subunit 2 | GTF2F2 | -2.93 | -2.35 | 0.59 |
|  | Q53F20 | Acidic (Leucine-rich) nuclear phosphoprotein 32 family, member E variant (Fragment) | ANP32E | -2.85 | -3.39 | -0.54 |
|  | Q5VT52 | Regulation of nuclear pre-mRNA domain-containing protein 2 | RPRD2 | -2.55 | -1.78 | 0.78 |
|  | Q9UBU9 | Nuclear RNA export factor 1 | NXF1 | -2.51 | -1.6 | 0.91 |
|  | A0A3B3ITJ4 | Heterogeneous nuclear ribonucleoprotein L (Fragment) | HNRNPL | -2.41 | -2.95 | -0.53 |
|  | Q6P486 | Nucleoporin 205 protein (Fragment) | NUP205 | 4.66 | 5.21 | 0.73 |
|  | P57740 | Nuclear pore complex protein Nup107 | NUP107 | 3.83 | 2.6 | -1.23 |
|  | Q9NQG5 | Regulation of nuclear pre-mRNA domain-containing protein 1B | RPRD1B | 3.44 | 2.23 | -1.2 |
|  | Q5SRE5 | Nucleoporin NUP188 | NUP188 | 3.13 | 2 | -1.13 |
|  | Q8TEM1 | Nuclear pore membrane glycoprotein 210 | NUP210 | 2.78 | 2.48 | -0.3 |
|  | G3V5V3 | Nuclear export mediator factor NEMF (Fragment) | NEMF | 2.43 | 0.9 | -1.53 |

**D. Neurodegeneration**

| **S. No.** | **Accession** | **Description** | **Gene Symbol** | **log_2_ Fold change: (NA) / (CON)** | **log_2_ Fold change: (NA_NOB) / (CON)** | **log_2_ Fold change: (NA_NOB) / (NA)** |
| --- | --- | --- | --- | --- | --- | --- |
|  | P16615 | Sarcoplasmic/endoplasmic reticulum calcium ATPase 2 | ATP2A2 | -2.84 | -2.13 | 0.72 |
|  | P42574 | Caspase-3 | CASP3 | -2.83 | -1.93 | 0.9 |
|  | P04040 | Catalase | CAT | -2.34 | -1.01 | 1.33 |
|  | P20674 | Cytochrome c oxidase subunit 5A, mitochondrial | COX5A | -3.24 | -3.4 | -0.16 |
|  | P19784 | Casein kinase II subunit alpha | CSNK2A2 | -4.71 | -5.93 | -1.65 |
|  | A0A2R8Y7Z0 | Catenin beta-1 | CTNNB1 | -3.95 | -4 | -0.05 |
|  | E7EX90 | Dynactin subunit 1 | DCTN1 | -2.93 | -2.47 | 0.46 |
|  | O00399 | Dynactin subunit 6 | DCTN6 | -2.97 | -1.29 | 1.67 |
|  | Q8TBR3 | Fusion (Involved in t(1216) in malignant liposarcoma) | FUS | -3.04 | -2.74 | 0.3 |
|  | P11021 | Endoplasmic reticulum chaperone BiP | HSPA5 | -4.72 | -2.15 | 2.57 |
|  | D7P639 | NADH-ubiquinone oxidoreductase chain 5 | ND5 | -2.77 | -2.86 | 0.36 |
|  | A0A1L1ZH79 | NADH-ubiquinone oxidoreductase chain 5 | ND5 | -2.75 | -1.55 | 1.2 |
|  | B4DJ81 | NADH-ubiquinone oxidoreductase 75 kDa subunit, mitochondrial | NDUFS1 | -3.6 | -2.63 | 0.96 |
|  | Q4LE43 | Phosphoinositide phospholipase C (Fragment) | PLCG1 | -4.33 | -3.51 | 0.82 |
|  | P28066 | Proteasome subunit alpha type-5 | PSMA5 | -3.93 | -2.49 | 1.44 |
|  | P60900 | Proteasome subunit alpha type-6 | PSMA6 | -4.64 | -3.94 | 0.88 |
|  | P49720 | Proteasome subunit beta type-3 | PSMB3 | -4.11 | -3.05 | 1.06 |
|  | P28070 | Proteasome subunit beta type-4 | PSMB4 | -2.67 | -2.7 | -0.03 |
|  | P28074 | Proteasome subunit beta type-5 | PSMB5 | -3.59 | -3.2 | 0.39 |
|  | P28072 | Proteasome subunit beta type-6 | PSMB6 | -4.85 | -4.19 | 0.66 |
|  | Q75L23 | 26S proteasome AAA-ATPase subunit RPT1 (Fragment) | PSMC2 | -2.59 | -1.45 | 1.14 |
|  | P43686 | 26S proteasome regulatory subunit 6B | PSMC4 | -2.93 | -2.98 | -0.05 |
|  | A0A087X2I1 | 26S proteasome regulatory subunit 10B | PSMC6 | -3.93 | -3.42 | 0.51 |
|  | O00231 | 26S proteasome non-ATPase regulatory subunit 11 | PSMD11 | -3.4 | -2.86 | 0.54 |
|  | A0A087WUL9 | 26S proteasome non-ATPase regulatory subunit 13 | PSMD13 | -2.99 | -3.09 | -0.1 |
|  | P55036 | 26S proteasome non-ATPase regulatory subunit 4 | PSMD4 | -2.61 | -2.67 | -0.07 |
|  | H3BNT7 | 26S proteasome non-ATPase regulatory subunit 7 | PSMD7 | -4.21 | -3.11 | 1.1 |
|  | R4GMR5 | 26S proteasome non-ATPase regulatory subunit 8 | PSMD8 | -2.57 | -2.26 | 0.3 |
|  | E2QRB3 | Pyrroline-5-carboxylate reductase | PYCR1 | -3.97 | -3.21 | 0.88 |
|  | J3KR12 | Pyrroline-5-carboxylate reductase | PYCR1 | -2.98 | -2.79 | 0.19 |
|  | Q6NVC0 | SLC25A5 protein (Fragment) | SLC25A5 | -5.42 | -4.22 | 1.2 |
|  | P00441 | Superoxide dismutase [Cu-Zn] | SOD1 | -4.86 | -5.03 | -0.17 |
|  | Q13148 | TAR DNA-binding protein 43 | TARDBP | -2.41 | -0.83 | 1.58 |
|  | C9JQ00 | Tubulin alpha chain (Fragment) | TUBA4A | -2.7 | -0.7 | 1.99 |
|  | Q9BVA1 | Tubulin beta-2B chain | TUBB2B | -4.17 | -0.62 | 3.55 |
|  | Q9BUF5 | Tubulin beta-6 chain | TUBB6 | -2.72 | -1.78 | 0.94 |
|  | P31930 | Cytochrome b-c1 complex subunit 1, mitochondrial | UQCRC1 | -2.65 | -3.81 | -1.17 |
|  | A0A384MDW7 | Enoyl Coenzyme A hydratase, short chain, 1, mitochondrial | ECHS1 | 2.52 | 2.31 | -0.21 |
|  | A0A0U1RRM8 | Fermitin family homolog 2 (Fragment) | FERMT2 | 2.33 | 1.14 | -1.2 |
|  | Q96DI8 | Heme oxygenase | HMOX1 | 5.99 | 0.78 | -5.89 |
|  | A0A6Q8PFJ4 | Mitofusin-2 | MFN2 | 2.33 | 3.46 | 1.12 |
|  | E7ESZ7 | NADH dehydrogenase [ubiquinone] 1 alpha subcomplex subunit 10, mitochondrial | NDUFA10 | 3.51 | 3.2 | -0.31 |
|  | A0A494C0D4 | Protoporphyrinogen oxidase (Fragment) | PPOX | 3.57 | 3.18 | -0.39 |
|  | Q5QPM7 | Proteasome inhibitor PI31 subunit | PSMF1 | 3.17 | 2.66 | -0.51 |
|  | A5D8Z4 | WNK1 protein (Fragment) | WNK1 | 2.51 | 3.12 | 0.61 |

**E. Programmed Cell Death**

| **S. No.** | **Accession** | **Description** | **Gene Symbol** | **log_2_ Fold change: (NA) / (CON)** | **log_2_ Fold change: (NA_NOB) / (CON)** | **log_2_ Fold change: (NA_NOB) / (NA)** |
| --- | --- | --- | --- | --- | --- | --- |
|  | S4R3H4 | Apoptotic chromatin condensation inducer in the nucleus | ACIN1 | -3.59 | -4.04 | -0.45 |
|  | Q9NQS1 | Cell death regulator Aven | AVEN | -3.31 | -3.63 | -0.31 |
|  | Q07021 | Complement component 1 Q subcomponent-binding protein, mitochondrial | C1QBP | -3.64 | -3.08 | 1.36 |
|  | P42574 | Caspase-3 | CASP3 | -2.83 | -1.93 | 0.9 |
|  | K7EQA9 | Hsp90 chaperone protein kinase-targeting subunit (Fragment) | CDC37 | -2.52 | -3.38 | -0.86 |
|  | Q9H444 | Charged multivesicular body protein 4b | CHMP4B | -3.32 | -3.92 | -0.59 |
|  | A0A2R8Y7Z0 | Catenin beta-1 | CTNNB1 | -3.95 | -4 | -0.05 |
|  | F8WBG8 | Drebrin-like protein | DBNL | -2.47 | -2.18 | 0.29 |
|  | Q5T7C4 | High mobility group protein B1 | HMGB1 | -5.25 | -3.36 | 1.92 |
|  | A0A6Q8PFJ0 | Prelamin-A/C | LMNA | -3.45 | -2.47 | 0.99 |
|  | P20700 | Lamin-B1 | LMNB1 | -2.79 | -3.14 | -0.34 |
|  | D7P639 | NADH-ubiquinone oxidoreductase chain 5 | ND5 | -2.77 | -2.86 | 0.36 |
|  | A0A1L1ZH79 | NADH-ubiquinone oxidoreductase chain 5 | ND5 | -2.75 | -1.55 | 1.2 |
|  | P28066 | Proteasome subunit alpha type-5 | PSMA5 | -3.93 | -2.49 | 1.44 |
|  | P60900 | Proteasome subunit alpha type-6 | PSMA6 | -4.64 | -3.94 | 0.88 |
|  | P49720 | Proteasome subunit beta type-3 | PSMB3 | -4.11 | -3.05 | 1.06 |
|  | P28070 | Proteasome subunit beta type-4 | PSMB4 | -2.67 | -2.7 | -0.03 |
|  | P28074 | Proteasome subunit beta type-5 | PSMB5 | -3.59 | -3.2 | 0.39 |
|  | P28072 | Proteasome subunit beta type-6 | PSMB6 | -4.85 | -4.19 | 0.66 |
|  | Q75L23 | 26S proteasome AAA-ATPase subunit RPT1 (Fragment) | PSMC2 | -2.59 | -1.45 | 1.14 |
|  | P43686 | 26S proteasome regulatory subunit 6B | PSMC4 | -2.93 | -2.98 | -0.05 |
|  | A0A087X2I1 | 26S proteasome regulatory subunit 10B | PSMC6 | -3.93 | -3.42 | 0.51 |
|  | B1AJY5 | 26S proteasome non-ATPase regulatory subunit 10 | PSMD10 | -3.78 | -3.09 | 0.69 |
|  | O00231 | 26S proteasome non-ATPase regulatory subunit 11 | PSMD11 | -3.4 | -2.86 | 0.54 |
|  | A0A087WUL9 | 26S proteasome non-ATPase regulatory subunit 13 | PSMD13 | -2.99 | -3.09 | -0.1 |
|  | P55036 | 26S proteasome non-ATPase regulatory subunit 4 | PSMD4 | -2.61 | -2.67 | -0.07 |
|  | H3BNT7 | 26S proteasome non-ATPase regulatory subunit 7 | PSMD7 | -4.21 | -3.11 | 1.1 |
|  | R4GMR5 | 26S proteasome non-ATPase regulatory subunit 8 | PSMD8 | -2.57 | -2.26 | 0.3 |
|  | Q6FHU3 | PSME1 protein (Fragment) | PSME1 | -2.58 | -2.18 | 0.4 |
|  | E2QRB3 | Pyrroline-5-carboxylate reductase | PYCR1 | -3.97 | -3.21 | 0.88 |
|  | J3KR12 | Pyrroline-5-carboxylate reductase | PYCR1 | -2.98 | -2.79 | 0.19 |
|  | E5RI99 | 60S ribosomal protein L30 (Fragment) | RPL30 | -2.58 | -1.62 | 0.95 |
|  | G5EA09 | Syndecan binding protein (Syntenin), | SDCBP | -2.62 | -3.34 | -0.72 |
|  | A0A087X0K9 | Tight junction protein ZO-1 | TJP1 | -3.14 | -3.62 | -0.48 |
|  | A0A0A6YYA0 | Protein TMED7-TICAM2 | TMED7-TICAM2 | -3.33 | -2.9 | 0.43 |
|  | Q96DI8 | Heme oxygenase | HMOX1 | 5.99 | 0.78 | -5.89 |
|  | J3KRA9 | Non-specific serine/threonine protein kinase (Fragment) | SMG1 | 2.85 | 1.24 | -1.61 |

**Supplementary Table S2:** List of proteins showing alteration in different pathways followed by NA exposure.
