# Supplementary material for: A protein–miRNA biomic analysis approach to explore neuroprotective potential of nobiletin in human neural progenitor cells (hNPCs)
Source: Front Pharmacol. 2024 Jan 25;15:1343569. doi: 10.3389/fphar.2024.1343569 (PMC10860404; doi:10.3389/fphar.2024.1343569)
Supplement: Supplementary file 6 [file Table5.DOCX]

**Supplementary Table S5**

**A. For up-regulated Proteins**

| **GO (Molecular function)** | | | | | |
| --- | --- | --- | --- | --- | --- |
| **S.No.** | **GO term** | **Count** | **GO: MF Term ID** | **P-value** | **Proteins (Up-regulated)** |
| 1 | Structural constituent of nuclear pore | 3 | GO:0017056 | 3.31E-03 | NUP205, NUP107, NUP188 |
| 2 | Magnesium ion binding | 5 | GO:0000287 | 4.97E-03 | THTPA, PDXK, MVK, NUDT16, AASDHPPT |

**B. For Down-regulated Proteins**

| **GO (Molecular function)** | | | | | |
| --- | --- | --- | --- | --- | --- |
| **S.No.** | **GO term** | **Count** | **GO: MF Term ID** | **P-value** | **Proteins (Down-regulated)** |
| 1 | RNA binding | 158 | GO:0003723 | 1.54E-49 | EIF4A1, RPL5, RPL30, AHCYL1, POP1, FMR1, RTCB, HDLBP, THUMPD3, PEBP1, YARS1, RPL10A, MKI67, CRKL, CCAR1, RPS15, PSMD4, TRIM28, CDH2, ZNF207, CPNE3, AGFG1, SRRM2, EIF1AX, RPL23, CIRBP, DNTTIP2, RPSA, ACTN4, SRRM1, PDIA4, SARNP, CLNS1A, MRTO4, NHP2, SBDS, SRSF3, SUCLG1, PPIG, DAP3, LRRC59, ANP32A, RBM8A, PEF1, PRKDC, RPN1, NOP2, RPF2, FXR1, LARP1, PCBP2, SERPINH1, UTP20, MAP4, ZC3H15, SPTBN1, HSPA9, TIA1, CPSF7, NOP16, FUS, ALYREF, LSM1, MRPL21, QKI, SMARCA4, HNRNPL, RPS28, SON, LAS1L, HNRNPF, GOLGB1, HNRNPD, UBE2N, RNPS1, HNRNPC, FARSA, RPS21, EIF4G3, RBM25, DAZAP1, CELF1, DDX42, SRP54, PSIP1, HMGB1, RO60, MRPL40, NELFB, METTL16, UTP14A, LBR, UTP15, ELAC2, ZFR, SMC1A, MRPL45, TSN, GNL3, HADHB, PSMA6, SARS2, ZNF638, TCP1, SLC25A5, MAPRE1, SF3B2, STAU1, AHNAK, ROCK2, SF3B6, NIP7, RPL12, SRRT, AKAP8, RRBP1, EXOSC6, NXF1, EXOSC10, PUF60, FIP1L1, POLR2B, PPP1R8, SAP18, FLNB, RBBP6, IGF2BP3, SNRPB2, RPS2, IGF2BP2, HNRNPA1, RAE1, EXOSC2, SF3B1, RPL17, HNRNPA0, RANBP2, NPM1, HNRNPA3, MYEF2, MDH2, YLPM1, IMMT, NAP1L1, DEK, HNRNPAB, U2SURP, KTN1, BOP1, EIF3K, IMPDH2, PDCD4, TSR1, ACIN1, FAM98A, SSBP1, TARDBP, EIF3C |
| 2 | Protein binding | 472 | GO:0005515 | 2.19E-24 | RPL5, RPL30, RNH1, CLPB, POP1, SMC2, PSMD8, PCMT1, LSM12, PSMD7, PSMD4, UBASH3B, STMN1, RPL23, CSNK2A2, DNTTIP2, GAPVD1, CD2AP, MTHFD1, WDR82, CLNS1A, SARNP, ZFYVE19, UQCRC1, PSME1, HPRT1, EXD2, ABCB7, PRKDC, RPN1, COPS7A, PRDX3, RHOT2, PPP4C, LARP1, EPB41L2, MYO6, VTI1A, SERPINH1, SSR1, PLCG1, CADM1, JUP, GPN1, PRDX6, PBX1, HNRNPL, EIF6, LAS1L, HNRNPF, HNRNPD, HNRNPC, FARSA, GMEB2, RPLP1, SRP54, C12ORF43, MRPL40, TRIM2, ANXA6, RPLP2, NELFB, ENOPH1, EMD, UBL7, TPI1, ACTL6A, NUTF2, MRPL45, BAZ1B, GNL3, PSMA5, HADHB, PSMA6, TUBB2B, PTRH2, IVD, AGPS, TCP1, PLIN3, PAFAH1B2, CRB2, FH, ASAH1, SF3B2, AHNAK, ATL3, STAU1, NIP7, SF3B6, RPL12, SRRT, PSMA7, PSMB6, PSMB4, EXOSC10, PSMB5, PSMB3, PPP1R8, FLNB, RPS2, SF3B1, RPL17, FARP1, TIGAR, MDH1, AGK, WDR18, ELP1, YLPM1, DHRS2, KTN1, LETM1, ERP44, DHRS7, PSMC6, PSMC4, PSMC2, TSR1, NUCB2, MTCH1, FMR1, CTNND1, RPL10A, TUBB6, CDH2, CFL2, CFL1, RUVBL1, MACROD1, ZNF207, CHP1, PMPCA, PLXNC1, AP2M1, SH3GLB1, TPM3, EIF1AX, RPSA, GTF2F2, TUBA4A, RNF40, ACLY, CLIP1, MRTO4, NHP2, SRSF3, PHIP, AAGAB, LRRC59, RAB5B, RAB5C, PEF1, FAM114A2, ARL3, KLC1, MACROH2A1, BCL2L13, TKFC, PSAP, PGK1, RAB6A, TIA1, OSBPL6, FUS, YJU2, LSM1, CORO2B, QKI, GFAP, COPS4, SON, COPS5, UBLCP1, SP3, PIN1, NACAD, CLPTM1L, COPS8, EIF4G3, MCU, ITGB1, MCFD2, MCM7, CELF1, ETFA, ECE1, PPP6R1, PPP6R3, WDR5, ITGAV, ACP1, LBR, ACTR3, SMC1A, TSN, DDOST, SKIC8, SARS2, DNAJC7, ASPH, POLR1A, ITGA7, ITGA6, DNAJC9, MCM6, SLC25A5, SEC22B, GAPDH, GTF3C1, SMARCD1, GTF2A1, ROCK2, UHRF1, MGRN1, RAB1B, RPE, PDHB, MFF, PUF60, FIP1L1, RBBP4, POLR2B, SAP18, IGF2BP3, SNRPB2, RBBP6, IGF2BP2, HNRNPA1, UGGT1, SEC11A, SPAG9, HNRNPA3, AHNAK2, IMMT, HNRNPAB, DHODH, EIF3M, PC, RAB14, EIF3K, IMPDH2, PDCD4, EIF3F, EIF3C, EIF4A1, AHCYL1, ECI2, RTCB, PREP, THUMPD3, TXNDC12, MKI67, NUDT5, ACTB, CRKL, CCAR1, TXNDC17, RPS15, GOLGA2, TRIM28, CHORDC1, RCC1, LONP2, KIF21A, CPNE3, SEPHS1, CIAO2B, ATP6V1E1, AGFG1, SKP1, EIF2A, SRRM2, ACTN1, CIRBP, ACTN4, MIF, SRRM1, CARHSP1, CDC37, CD2BP2, SUCLG2, SUCLG1, EPHA2, ANP32A, TEX264, TWF1, MYL12A, RPAP3, FXR1, PDCD10, PCBP2, MLEC, MAP4, SPTBN1, PYCR1, DCLK2, SMARCA4, AIMP1, POLA1, RPS28, EPRS1, PTK7, GOLGB1, CWF19L1, ECHDC1, CALU, PAF1, RNPS1, RPS21, FEN1, DAZAP1, FKBP15, TECR, MIA3, PEPD, AKAP12, HINT1, PCM1, SPTLC1, ZMYM2, C1QBP, SH3PXD2B, CHAMP1, UBXN7, KIF1B, UTP14A, DBNL, ZFR, AKR1A1, VSNL1, MAPRE1, MRRF, DBN1, ATPAF1, RAB7A, SPINDOC, AKAP8, GNAI3, COX5A, LIMA1, SAMM50, LMNA, CCT8, EMC8, RAE1, EXOSC2, RANBP2, NPM1, PEX19, FAH, HYPK, ALDH4A1, TJP1, BOP1, CTNNB1, TARDBP, SCARB2, STEAP3, CD81, HDLBP, PEBP1, YARS1, EEF1B2, PPME1, POGZ, NRCAM, FAM3C, ACAA1, ATP1B3, PDIA4, TMX3, TBL1XR1, DNAJB11, SBDS, CHMP4B, PPIG, CARS1, HDGFL2, DAP3, PSMD10, ISYNA1, PSMD11, RBM8A, UBA6, UBA5, NOP2, RPF2, ACACA, SDCBP, EP400, NLRP2, UTP20, ZC3H15, HSPA9, MPST, NIBAN1, CPSF7, PDHA1, HSPA5, UQCC2, IDH1, ALYREF, NUP153, GRHPR, DLG1, EIF2S3, ATG16L1, GNB2, TBCE, UBE2N, CNTN2, IFT27, BLMH, AVEN, LGALS3BP, ACADVL, RBM25, DDX42, ATP2A2, PSIP1, HMGB1, DNPH1, GPHN, LMNB1, NTMT1, CASP3, LRRFIP2, LRRFIP1, UTP15, ATP6AP2, QRSL1, CKAP5, DNM2, RCN3, RCN1, RCN2, DDAH2, PEX3, EEF1D, PFDN2, NDUFS1, USP14, DCTN1, PNKP, GMPR2, SELENOF, ATP1A1, SPG7, STRN3, PDLIM1, NXF1, MAT2A, POLD2, BUB3, IGBP1, NIPSNAP3A, NAP1L1, DEK, SOD2, TTC19, U2SURP, SOD1, ERCC3, GNPDA2, ACIN1, QRICH1, FAM98A, SCLY, SSBP1, ABCE1, NUP37 |
| 3 | Cadherin binding | 45 | GO:0045296 | 4.69E-18 | ITGB1, LRRC59, AHNAK, CTNND1, HDLBP, TWF1, CRKL, GOLGA2, PSMB6, LIMA1, PCMT1, LARP1, CDH2, PUF60, PPME1, RUVBL1, FLNB, CCT8, RPS2, LRRFIP1, EMD, ZC3H15, SPTBN1, EIF2A, SH3GLB1, DBNL, JUP, HSPA5, IDH1, GAPVD1, CKAP5, PRDX6, CD2AP, KTN1, TJP1, DLG1, EIF2S3, EEF1D, CTNNB1, CHMP4B, PLIN3, ITGA6, MAPRE1, DBN1, EPHA2 |
| 4 | mRNA binding | 31 | GO:0003729 | 5.69E-11 | EIF4A1, RBM25, RBM8A, STAU1, CELF1, FMR1, SF3B6, HDLBP, FXR1, NXF1, C1QBP, PPP1R8, PCBP2, IGF2BP3, IGF2BP2, RPS2, SF3B1, HNRNPA0, EIF2A, SRRM2, CPSF7, HNRNPA3, MYEF2, ALYREF, HNRNPAB, TSN, QKI, HNRNPL, DHFR, ZNF638, EIF4G3 |
| 5 | Translation initiation factor activity | 11 | GO:0003743 | 1.47E-05 | EIF4A1, EIF3M, EIF2S3, COPS5, EIF3K, EIF6, EIF1AX, EIF3F, EIF3C, EIF4G3, EIF2A |
| 6 | mRNA 3'-UTR binding | 21 | GO:0003730 | 3.68E-05 | RPL5, DAZAP1, TIA1, HNRNPA3, CELF1, FUS, FMR1, CIRBP, RNF40, HNRNPL, FXR1, CARHSP1, LARP1, NHP2, RNPS1, IGF2BP3, HNRNPC, IGF2BP2, HNRNPA1, TARDBP, HNRNPA0 |
| 7 | Identical protein binding | 79 | GO:0042802 | 3.78E-05 | ACADVL, STEAP3, AHCYL1, GMEB2, FMR1, NUDT5, DNPH1, GPHN, ACTB, CRKL, GOLGA2, PCM1, PSMD4, UBASH3B, CDH2, SACS, ANXA6, SEPHS1, SH3GLB1, ADSL, NUTF2, MIF, TSN, CD2AP, CLIP1, IVD, CAT, CHMP4B, HPRT1, MCM6, MAPRE1, CARS1, UMPS, GAPDH, PAFAH1B2, AHNAK, ATL3, PEF1, UHRF1, RPE, MFF, ACACA, PSMA7, PRDX3, SDCBP, PUF60, MAT2A, PSAP, LMNA, MYO6, FLNB, HNRNPA1, DECR1, MPST, SPAG9, FUS, IDH1, PYCR1, NUP153, SOD2, PRDX6, GFAP, SOD1, ALDH4A1, GALE, PSMC6, PC, EPRS1, GNPDA2, ATG16L1, IMPDH2, CNTN2, BLMH, SSBP1, EIF3F, HNRNPC, TARDBP, MCU, ITPA |
| 8 | Catalytic activity | 14 | GO:0003824 | 1.98E-04 | ADSL, GAA, GMPR2, ATP6AP2, ECI2, QRSL1, DHODH, HINT1, PC, SPTLC1, DDAH1, DDAH2, ECHDC1, SCLY |
| 9 | Protein homodimerization activity | 40 | GO:0042803 | 2.43E-04 | SCARB2, EXD2, ABCB7, GLDC, UBA5, FMR1, RPE, ECE1, NUDT5, MFF, DNPH1, ADD2, FXR1, PSMD7, MTHFD1L, PDCD10, PSAP, LRRFIP1, SEPHS1, SH3GLB1, NPM1, TPI1, JUP, CADM1, ACTN1, IDH1, ACTN4, RNF40, AIMP1, GRHPR, GALE, TP53I3, EPRS1, XPNPEP1, CAT, CHMP4B, SCLY, SSBP1, CARS1, PAFAH1B2 |
| 10 | ATPase activity | 27 | GO:0016887 | 2.56E-04 | EIF4A1, MCM7, CLPB, ABCB7, DDX42, ATP2A2, ATP1A1, SPG7, SMC2, RUVBL1, LONP2, KIF21A, KIF1B, CCT8, HSPA9, HSPA5, SMC1A, GTF2F2, SMARCA4, PSMC6, ERCC3, PSMC4, PSMC2, TCP1, ACIN1, MCM6, ABCE1 |
| 11 | Structural constituent of cytoskeleton | 12 | GO:0005200 | 2.61E-04 | ACTR3, TUBB2B, TUBB6, LMNA, ADD3, SPTBN1, ACTB, TUBA4A, LMNB1, CD2AP, ADD2, GFAP |
| 12 | tRNA binding | 10 | GO:0000049 | 3.88E-04 | AIMP1, EIF2S3, SARS2, ELP1, THUMPD3, EIF2AK4, FARSA, CARS1, YARS1, EIF2A |
| 13 | Structural constituent of ribosome | 16 | GO:0003735 | 4.59E-04 | RPL5, RPL30, RPLP1, RPL23, RPL12, RPSA, RPL10A, MRPL21, RPS15, RPS28, TBCE, RPLP2, RPS2, RPS21, RPL17, DAP3 |
| 14 | Oxidoreductase activity, acting on the CH-CH group of donors | 5 | GO:0016627 | 7.48E-04 | ACAD8, ACADVL, IVD, TECR, LBR |
| 15 | GTP binding | 25 | GO:0005525 | 7.54E-04 | RAB5B, RAB5C, ATL3, ARL3, RAB1B, RTCB, SRP54, GNAI3, TUBB6, RHOT2, ANXA6, SEPHS1, RAB6A, GPN1, TUBA4A, GNL3, DNM2, TUBB2B, EIF2S3, RAB14, IFT27, SUCLG2, TSR1, RAB7A, DAP3 |
| 16 | mRNA 5'-UTR binding | 6 | GO:0048027 | 8.10E-04 | RPL5, LARP1, FMR1, IGF2BP3, IGF2BP2, GNL3 |
| 17 | NADPH binding | 5 | GO:0070402 | 9.75E-04 | DHFR, GRHPR, TP53I3, LBR, DECR1 |
| 18 | Nucleosomal DNA binding | 7 | GO:0031492 | 1.11E-03 | RBBP4, ACTL6A, RCC1, HNRNPC, MACROH2A1, ACTB, SMARCA4 |
| 19 | Protein N-terminus binding | 11 | GO:0047485 | 1.12E-03 | SRRM2, SDCBP, PEX19, ERCC3, PDCD10, TBL1XR1, CSNK2A2, ACTN4, RPS21, HYPK, SMARCA4 |
| 20 | Actin filament binding | 17 | GO:0051015 | 1.13E-03 | ACTR3, DBNL, TPM3, ACTN1, TWF1, ACTN4, ADD3, CORO2B, ADD2, LIMA1, CFL2, MYO6, CFL1, ANXA6, FLNB, SPTBN1, DBN1 |
| 21 | NADP binding | 7 | GO:0050661 | 1.27E-03 | DHFR, GRHPR, NNT, IDH1, GSR, CAT, GAPDH |
| 22 | Ribosome binding | 9 | GO:0043022 | 1.36E-03 | LETM1, EIF3K, EIF6, HSPA5, FMR1, SBDS, RPSA, EIF3C, EIF2A |
| 23 | Unfolded protein binding | 12 | GO:0051082 | 1.47E-03 | HSPA9, NPM1, HSPA5, CDC37, TCP1, DNAJB11, SERPINH1, PFDN2, NACAD, CCT8, SPG7, UGGT1 |
| 24 | ATP binding | 66 | GO:0005524 | 1.60E-03 | EIF4A1, CLPB, MCM7, DDX42, PEBP1, ATP2A2, MKI67, YARS1, GPHN, ACTB, SMC2, RUVBL1, CHORDC1, LONP2, KIF21A, KIF1B, SEPHS1, ACTR3, CSNK2A2, SMC1A, BAZ1B, QRSL1, GTF2F2, ACLY, SARS2, MTHFD1, TCP1, SUCLG2, MCM6, CARS1, EPHA2, ROCK2, ABCB7, UBA6, PRKDC, UBA5, PNKP, TWF1, SPG7, ATP1A1, ACACA, NME1-NME2, TKFC, MTHFD1L, MAT2A, MYO6, EP400, NLRP2, PGK1, CCT8, HSPA9, HSPA5, AGK, DCLK2, EIF2AK4, SMARCA4, PSMC6, PC, EPRS1, ERCC3, PSMC4, PTK7, PSMC2, UBE2N, FARSA, ABCE1 |
| 25 | Carbonyl reductase (NADPH) activity | 4 | GO:0004090 | 1.80E-03 | CBR1, DHRS7, DHRS4L2, DHRS2 |
| 26 | Purine nucleotide binding | 3 | GO:0017076 | 2.48E-03 | POLA1, FAM114A2, PNKP |
| 27 | Oxidoreductase activity | 16 | GO:0016491 | 2.63E-03 | CBR1, GMPR2, TECR, SELENOF, ETFA, COX5A, DHRS2, ALDH4A1, DHRS7, TP53I3, NNT, AGPS, FDXR, DHRS4L2, RDH13, DECR1 |
| 28 | N6-methyladenosine-containing RNA binding | 4 | GO:1990247 | 3.39E-03 | FMR1, IGF2BP3, IGF2BP2, HNRNPC |
| 29 | Translation factor activity, RNA binding | 5 | GO:0008135 | 4.04E-03 | EIF4A1, EIF2S3, EEF1D, EIF1AX, EIF4G3 |
| 30 | Translation initiation factor binding | 5 | GO:0031369 | 4.04E-03 | EIF3M, LARP1, FMR1, EIF3F, EIF3C |
| 31 | GTPase activity | 21 | GO:0003924 | 4.28E-03 | RAB5B, RAB5C, ATL3, ARL3, RAB1B, SRP54, GNAI3, GPN1, TUBA4A, DNM2, TUBB6, TUBB2B, RHOT2, EIF2S3, RAB14, GNB2, TSR1, IFT27, ABCE1, RAB6A, RAB7A |
| 32 | RNA cap binding | 4 | GO:0000339 | 4.42E-03 | EIF4A1, LARP1, LSM1, EIF4G3 |
| 33 | Microtubule binding | 17 | GO:0008017 | 4.69E-03 | DCTN1, ARL3, FMR1, DCLK2, RMDN1, CKAP5, DNM2, GOLGA2, CLIP1, ZNF207, SBDS, CHP1, KIF21A, KIF1B, MAP4, RAE1, GAPDH |
| 34 | Single-stranded DNA binding | 10 | GO:0003697 | 5.74E-03 | POLA1, MYEF2, MCM7, PCBP2, SSBP1, HMGB1, MCM6, HNRNPA1, TSN, SMC2 |
| 35 | Lyase activity | 6 | GO:0016829 | 5.80E-03 | HMGCL, ACLY, ADSL, GLDC, SCLY, HMGB1 |
| 36 | Nucleotide binding | 9 | GO:0000166 | 6.07E-03 | POLA1, HINT1, EXOSC10, SARS2, IMPDH2, SUCLG1, HPRT1, ATP1A1, ITPA |
| 37 | Threonine-type endopeptidase activity | 4 | GO:0004298 | 7.01E-03 | PSMB6, PSMB4, PSMB5, PSMB3 |
| 38 | GTPase activating protein binding | 4 | GO:0032794 | 8.58E-03 | ARL3, GNAI3, PIN1, GAPVD1 |
| 39 | Ubiquitin protein ligase binding | 18 | GO:0031625 | 1.02E-02 | HSPA9, RPL5, TPI1, HSPA5, RPL23, ACTN4, PRDX6, RNF40, TRIM28, UBASH3B, TCP1, PCBP2, DBT, UBE2N, UQCRC1, UBXN7, CTNNB1, SLC25A5 |
| 40 | Histone binding | 13 | GO:0042393 | 1.02E-02 | NPM1, FH, ANP32A, UHRF1, NAP1L1, DEK, BAZ1B, SMARCA4, RBBP4, TBL1XR1, WDR5, RCC1, DNAJC9 |
| 41 | Peptidase activity | 9 | GO:0008233 | 1.16E-02 | PSMB5, CASP3, PSMC2, LONP2, SPG7, PEPD, SEC11A, CNDP2, LGMN |
| 42 | Proteasome-activating ATPase activity | 3 | GO:0036402 | 1.17E-02 | PSMC6, PSMC4, PSMC2 |
| 43 | Membrane insertase activity | 4 | GO:0032977 | 1.23E-02 | MTCH1, EMC1, EMC10, EMC8 |
| 44 | Electron carrier activity | 7 | GO:0009055 | 1.31E-02 | ALDH4A1, ASPH, GLDC, GSR, ETFA, NDUFS1, COX5A |
| 45 | Hydrolase activity | 15 | GO:0016787 | 1.37E-02 | EIF4A1, ASAH1, AHCYL1, NUDT5, TUBA4A, ACTB, CNDP2, SMARCA4, FAHD2B, HINT1, ERCC3, XPNPEP1, EP400, BPNT2, ATP6V1E1 |
| 46 | Protein disulfide isomerase activity | 4 | GO:0003756 | 1.44E-02 | ERP44, TMX3, QSOX2, PDIA4 |
| 47 | Protein binding involved in protein folding | 6 | GO:0044183 | 1.60E-02 | HSPA9, HSPA5, TCP1, PFDN2, CCT8, HYPK |
| 48 | Thioredoxin peroxidase activity | 3 | GO:0008379 | 1.61E-02 | PRDX3, SELENOF, PRDX6 |
| 49 | Ribonucleoprotein complex binding | 5 | GO:0043021 | 1.65E-02 | BOP1, RPLP1, SRP54, CD2BP2, CKAP5 |
| 50 | GTPase binding | 5 | GO:0051020 | 1.82E-02 | AIMP1, EPRS1, ATG16L1, GNB2, SPTBN1 |
| 51 | Protein kinase binding | 25 | GO:0019901 | 1.88E-02 | DCTN1, CTNND1, PEBP1, MACROH2A1, ACTB, STRN3, ADD2, GOLGA2, PRDX3, CDH2, PPME1, PDCD10, RBBP6, PLCG1, SLC12A7, HNRNPA0, NPM1, JUP, TUBA4A, DNM2, POLA1, DLG1, CDC37, CTNNB1, MAPRE1 |
| 52 | GDP binding | 7 | GO:0019003 | 2.06E-02 | RAB5B, RAB5C, RAB14, ARL3, GNAI3, SRP54, RAB7A |
| 53 | Double-stranded DNA binding | 9 | GO:0003690 | 2.10E-02 | FEN1, PRKDC, PNKP, ZNF638, AKAP8, PSIP1, HMGB1, TARDBP, HDGFL2 |
| 54 | Transcription coactivator activity | 15 | GO:0003713 | 2.36E-02 | SMARCD1, NPM1, JUP, FUS, ACTL6A, PSIP1, HMGB1, ACTN4, CCAR1, SMARCA4, PDLIM1, COPS5, TRIM28, RUVBL1, CTNNB1 |
| 55 | Microtubule plus-end binding | 4 | GO:0051010 | 2.50E-02 | CLIP1, DCTN1, MAPRE1, CKAP5 |
| 56 | Oxidoreductase activity, acting on the CH-OH group of donors, NAD or NADP as acceptor | 5 | GO:0016616 | 2.61E-02 | GRHPR, CBR1, DHRS7, MDH1, DHRS2 |
| 57 | Ubiquitin ligase inhibitor activity | 3 | GO:1990948 | 2.65E-02 | RPS15, RPL5, RPL23 |
| 58 | RNA stem-loop binding | 4 | GO:0035613 | 3.15E-02 | DAZAP1, EPRS1, FMR1, METTL16 |
| 59 | Protein disulfide oxidoreductase activity | 4 | GO:0015035 | 3.15E-02 | TMX3, TXNL1, TXNDC12, PDIA4 |
| 60 | Tat protein binding | 3 | GO:0030957 | 3.26E-02 | NPM1, ACTB, SMARCA4 |
| 61 | Heat shock protein binding | 6 | GO:0031072 | 3.40E-02 | HSPA9, DNAJC7, HSPA5, EEF1D, CDC37, DNAJC9 |
| 62 | Chromatin binding | 22 | GO:0003682 | 3.43E-02 | SMARCD1, NPM1, FUS, FMR1, ACTL6A, PSIP1, NAP1L1, SMC1A, SMC2, POLA1, TRIM28, WDR82, POLR2B, POLR1A, SARNP, SP3, EP400, HNRNPD, RCC1, CTNNB1, PAF1, SSBP1 |
| 63 | Tau protein binding | 5 | GO:0048156 | 3.57E-02 | ROCK2, DCTN1, PIN1, ACTB, LGMN |
| 64 | Nucleic acid binding | 17 | GO:0003676 | 3.59E-02 | EXD2, EIF4A1, DAZAP1, NPM1, RBM25, CPSF7, UHRF1, SRRT, ZFR, CIRBP, CARHSP1, ZNF638, HNRNPF, ACIN1, RBBP6, IGF2BP2, HNRNPA1 |
| 65 | Alpha-catenin binding | 3 | GO:0045294 | 3.90E-02 | JUP, CDH2, CTNNB1 |
| 66 | U3 snoRNA binding | 3 | GO:0034511 | 3.90E-02 | PRKDC, NHP2, TSR1 |
| 67 | Acyl-CaA dehydrogenase activity | 3 | GO:0003995 | 3.90E-02 | ACAD8, ACADVL, IVD |
| 68 | Chaperone binding | 8 | GO:0051087 | 3.99E-02 | SCARB2, HSPA5, CDC37, TBCE, SACS, DNAJC9, ATP1A1, SOD1 |
| 69 | Actin binding | 17 | GO:0003779 | 4.19E-02 | ITGB1, ACTR3, DBNL, FKBP15, TPM3, ACTN1, TWF1, ACTN4, CORO2B, ADD2, PDLIM1, EPB41L2, MYO6, FLNB, EMD, SPTBN1, DBN1 |
| 70 | Vinculin binding | 3 | GO:0017166 | 4.60E-02 | ACTN1, RTCB, CORO2B |
| 71 | Isopeptidase activity | 3 | GO:0070122 | 4.60E-02 | PSMD7, COPS5, EIF3F |
| 72 | mRNA 3'-UTR AU-rich region binding | 4 | GO:0035925 | 4.68E-02 | FXR1, TIA1, HNRNPD, HNRNPA0 |
| 73 | Kinesin binding | 5 | GO:0019894 | 4.72E-02 | SPAG9, KIF1B, KLC1, ACTB, KTN1 |
| 74 | Flavin adenine dinucleotide binding | 6 | GO:0050660 | 4.77E-02 | ACAD8, ACADVL, IVD, GSR, AGPS, ETFA |
| 75 | Manganese ion binding | 6 | GO:0030145 | 4.77E-02 | EXD2, HMGCL, FEN1, XPNPEP1, PEPD, SOD2 |
| 76 | Cell adhesion molecule binding | 6 | GO:0050839 | 5.03E-02 | ITGB1, TJP1, SDCBP, JUP, CADM1, PTK7 |

**Supplementary Table S5:** List of all identified Molecular functions with their protein count and proteins were analysed by Database for Annotation, Visualization and Integrated Discovery (DAVID) platform with a significant *p*-value ≤ 0.05 of differentially expressed proteins (up-regulated and down-regulated) identified by the high-resolution mass spectrometry (HRMS).
