# Supplementary material for: A protein–miRNA biomic analysis approach to explore neuroprotective potential of nobiletin in human neural progenitor cells (hNPCs)
Source: Front Pharmacol. 2024 Jan 25;15:1343569. doi: 10.3389/fphar.2024.1343569 (PMC10860404; doi:10.3389/fphar.2024.1343569)
Supplement: Supplementary file 7 [file Table3.DOCX]

**Supplementary Table S3**

**A. Up-regulated Proteins**

| **GO (Biological process)** | | | | | |
| --- | --- | --- | --- | --- | --- |
| **S.No.** | **Gene Ontology Term** | **Count** | **GO: BP Term ID** | **P-value** | **Proteins (Up-regulated)** |
| 1 | Nucleocytoplasmic transport | 4 | GO:0006913 | 5.07E-04 | NUP205, NUP107, NUP188, NUP210 |
| 2 | Protein imports into nucleus | 4 | GO:0006606 | 3.64E-03 | NUP107, NUP188, PPP1R10, RANBP6 |
| 3 | Chromatin assembly | 2 | GO:0031497 | 2.61E-02 | CDAN1, RSF1 |
| 4 | mRNA transport | 3 | GO:0051028 | 2.64E-02 | NUP205, NUP188, NUP210 |
| 5 | Glycogen catabolic process | 2 | GO:0005980 | 2.90E-02 | AGL, PYGL |
| 6 | Nuclear pore complex assembly | 2 | GO:0051292 | 2.90E-02 | NUP205, NUP107 |
| 7 | Mitochondrial translation | 3 | GO:0032543 | 3.24E-02 | GATB, MRPL28, MRPS30 |
| 8 | TFG- beta receptor signaling pathway | 3 | GO:0007179 | 3.55E-02 | FNTA, ADAM9, FERMT2 |
| 9 | Negative regulation of macroautophagy | 2 | GO:0016242 | 4.32E-02 | SMG1, HMOX1 |
| 10 | Actin filament-based movement | 2 | GO:0030048 | 4.88E-02 | MYO9B, WASL |

**B. Down-regulated Proteins**

| **GO (Biological process)** | | | | | |
| --- | --- | --- | --- | --- | --- |
| **S.No.** | **Gene Ontology Term** | **Count** | **GO: BP Term ID** | **P-value** | **Proteins (Down-regulated)** |
| 1 | Tricarboxylic acid cycle | 11 | GO:0006099 | 2.88E-08 | ACLY, FH, PDHA1, MDH1, NNT, MDH2, IDH1, SUCLG2, SUCLG1, ACO2, PDHB |
| 2 | Translational initiation | 13 | GO:0006413 | 3.29E-08 | EIF4A1, EIF1AX, EIF3M, LARP1, COPS5, EIF2S3, EIF3K, EIF6, EIF3F, ABCE1, EIF3C, EIF4G3, EIF2A |
| 3 | RNA splicing | 23 | GO:0008380 | 1.25E-07 | TIA1, SF3B2, RBM25, RBM8A, FUS, FMR1, YJU2, LSM1, QKI, SRRM1, SON, PUF60, C1QBP, ZNF638, PPP1R8, ACIN1, SAP18, RNPS1, HNRNPC, PPIG, HNRNPA1, TARDBP, SF3B1 |
| 4 | Proteasome-mediated ubiquitin-dependent protein catabolic process | 23 | GO:0043161 | 1.89E-07 | USP14, PSMD11, PSMD13, PSMA7, CD2AP, PSMD8, PSMA5, PSMB6, PSMA6, PSMB4, PSMC6, PSMD7, PSMB5, PSMD4, TBL1XR1, PSMC4, PSMB3, TRIM2, PSMC2, PCBP2, UBXN7, CTNNB1, SKP1 |
| 5 | Regulation of mRNA stability | 12 | GO:0043488 | 1.13E-06 | PSMA5, FXR1, PSMB6, PSMA6, CARHSP1, PSMB4, PSMC6, PSMB5, PSMC4, PSMB3, FMR1, PSMA7 |
| 6 | Cytoplasmic translation | 14 | GO:0002181 | 1.41E-06 | RPL5, RPL30, RPLP1, RPL23, RPL12, RPSA, RPL10A, RPS15, RPS28, RPLP2, RPS2, RPS21, RPL17, ZC3H15 |
| 7 | mRNA processing | 22 | GO:0006397 | 2.51E-06 | TIA1, SF3B2, RBM25, RBM8A, CELF1, FMR1, SRRT, LSM1, QKI, SRRM1, HNRNPL, SON, FIP1L1, PUF60, C1QBP, PPP1R8, ACIN1, SAP18, RBBP6, HNRNPA1, TARDBP, HNRNPA0 |
| 8 | Positive regulation of translation | 13 | GO:0045727 | 4.03E-06 | NIBAN1, RPL5, NPM1, PRKDC, FMR1, CIRBP, HNRNPL, FXR1, LARP1, EIF6, HNRNPD, EIF3C, EIF4G3 |
| 9 | Translation | 21 | GO:0006412 | 4.94E-06 | RPL5, RPL30, RPLP1, RPL23, RPL12, RPSA, RRBP1, RPL10A, MRPL21, RPS15, AIMP1, RPS28, COPS5, TBCE, RPLP2, IGF2BP3, RPS2, FARSA, MRRF, RPS21, RPL17 |
| 10 | Regulation of alternative mRNA splicing, via spliceosome | 11 | GO:0000381 | 1.14E-05 | HNRNPL, FXR1, TIA1, RBM25, RBM8A, PUF60, CELF1, FMR1, SAP18, RNPS1, HNRNPA1 |
| 11 | MRNA transport | 12 | GO:0051028 | 2.96E-05 | RANBP2, NXF1, HNRNPA3, RBM8A, FMR1, IGF2BP3, NUP153, NUTF2, IGF2BP2, HNRNPA1, QKI, NUP37 |
| 12 | Negative regulation of G2/M transition of mitotic cell cycle | 8 | GO:0010972 | 3.10E-05 | PSMA5, AVEN, PSMB6, PSMA6, PSMB4, PSMB5, PSMB3, PSMA7 |
| 13 | RRNA processing | 15 | GO:0006364 | 3.13E-05 | UTP15, RPL5, WDR18, RPS15, BOP1, EXOSC6, RPS28, EXOSC10, LAS1L, MRTO4, NHP2, SBDS, UTP20, EXOSC2, UTP14A |
| 14 | Pre-replicative complex assembly | 7 | GO:0036388 | 3.30E-05 | PSMA5, PSMB6, PSMA6, PSMB4, PSMB5, PSMB3, PSMA7 |
| 15 | Regulation of hematopoietic stem cell differentiation | 8 | GO:1902036 | 3.81E-05 | PSMA5, PSMB6, PSMA6, PSMB4, PSMB5, PRKDC, PSMB3, PSMA7 |
| 16 | Maturation of LSU-rRNA | 6 | GO:0000470 | 4.18E-05 | EIF6, LAS1L, NOP2, NHP2, RPL10A, RPF2 |
| 17 | Regulation of cellular amino acid metabolic process | 7 | GO:0006521 | 4.29E-05 | PSMA5, PSMB6, PSMA6, PSMB4, PSMB5, PSMB3, PSMA7 |
| 18 | Antigen processing and presentation of exogenous peptide antigen via MHC class I, TAP-dependent | 7 | GO:0002479 | 5.52E-05 | PSMA5, PSMB6, PSMA6, PSMB4, PSMB5, PSMB3, PSMA7 |
| 19 | Regulation of transcription from RNA polymerase II promoter in response to hypoxia | 7 | GO:0061418 | 8.79E-05 | PSMA5, PSMB6, PSMA6, PSMB4, PSMB5, PSMB3, PSMA7 |
| 20 | Negative regulation of translation | 11 | GO:0017148 | 2.04E-04 | DHFR, FXR1, TIA1, LARP1, EPRS1, TRIM2, FMR1, CIRBP, IGF2BP3, IGF2BP2, GAPDH |
| 21 | Protein folding | 16 | GO:0006457 | 2.38E-04 | RANBP2, HSPA9, HYPK, PDIA4, ERP44, DNAJC7, CDC37, GNB2, TCP1, DNAJB11, TBCE, SACS, PFDN2, CCT8, PPIG, QSOX2 |
| 22 | Regulation of mRNA splicing, via spliceosome | 6 | GO:0048024 | 2.46E-04 | TIA1, SON, SRSF3, METTL16, QKI, SRRM1 |
| 23 | Fc-epsilon receptor signaling pathway | 8 | GO:0038095 | 3.71E-04 | PSMA5, PSMB6, PSMA6, PSMB4, PSMB5, PSMB3, PLCG1, PSMA7 |
| 24 | Anaphase-promoting complex-dependent catabolic process | 8 | GO:0031145 | 3.71E-04 | PSMA5, PSMB6, PSMA6, PSMB4, PSMB5, PSMB3, PSMA7, ANAPC1 |
| 25 | Positive regulation of canonical Wnt signaling pathway | 13 | GO:0090263 | 3.93E-04 | JUP, ATP6AP2, PSMA7, PSMA5, PSMB6, PSMA6, PSMB4, PSMB5, TBL1XR1, PTK7, PSMB3, RUVBL1, PIN1 |
| 26 | Proteasomal protein catabolic process | 7 | GO:0010498 | 6.30E-04 | PSMA5, PSMB6, PSMA6, PSMB4, PSMB5, PSMB3, PSMA7 |
| 27 | Positive regulation of double-strand break repair via homologous recombination | 7 | GO:1905168 | 7.27E-04 | FUS, ACTL6A, POGZ, RUVBL1, EP400, HDGFL2, ACTB |
| 28 | Protein stabilization | 17 | GO:0050821 | 7.56E-04 | RPL5, PEX19, RPL23, CTNND1, ATP1B3, HYPK, RPAP3, PDCD10, CDC37, TCP1, RUVBL1, ZNF207, PIN1, CHP1, PFDN2, CCT8, GAPDH |
| 29 | NIK/NF-kappab signaling | 8 | GO:0038061 | 9.62E-04 | PSMA5, PSMB6, PSMA6, PSMB4, PPP4C, PSMB5, PSMB3, PSMA7 |
| 30 | Regulation of double-strand break repair | 6 | GO:2000779 | 1.01E-03 | PPP4C, ACTL6A, RUVBL1, EP400, DEK, ACTB |
| 31 | Post-translational protein modification | 9 | GO:0043687 | 1.03E-03 | PSMA5, PSMB6, PSMA6, PSMB4, COPS5, PSMB5, PSMB3, BAZ1B, PSMA7 |
| 32 | Mitotic spindle organization | 8 | GO:0007052 | 1.19E-03 | DCTN6, STMN1, TBCE, SBDS, RCC1, MAP4, RMDN1, CKAP5 |
| 33 | MRNA splicing, via spliceosome | 15 | GO:0000398 | 1.39E-03 | SRRM2, SF3B2, HNRNPA3, RBM8A, ALYREF, SF3B6, SRRM1, HNRNPF, CWF19L1, SRSF3, SNRPB2, RNPS1, HNRNPC, HNRNPA1, SF3B1 |
| 34 | Purine nucleotide biosynthetic process | 5 | GO:0006164 | 1.40E-03 | ADSL, MTHFD1, GMPR2, IMPDH2, HPRT1 |
| 35 | MRNA export from nucleus | 8 | GO:0006406 | 1.45E-03 | NXF1, RBM8A, SARNP, FMR1, ALYREF, SRSF3, RAE1, AGFG1 |
| 36 | Interleukin-1-mediated signaling pathway | 7 | GO:0070498 | 1.57E-03 | PSMA5, PSMB6, PSMA6, PSMB4, PSMB5, PSMB3, PSMA7 |
| 37 | Tumor necrosis factor-mediated signaling pathway | 9 | GO:0033209 | 1.59E-03 | PSMA5, PSMB6, PSMA6, PSMB4, PSMB5, PSMB3, ACTN4, PSMA7, TXNDC17 |
| 38 | Positive regulation of proteasomal protein catabolic process | 5 | GO:1901800 | 1.74E-03 | PSMC6, PSMC4, FMR1, PSMC2, RNF40 |
| 39 | Stimulatory C-type lectin receptor signaling pathway | 7 | GO:0002223 | 1.77E-03 | PSMA5, PSMB6, PSMA6, PSMB4, PSMB5, PSMB3, PSMA7 |
| 40 | Regulation of RNA splicing | 9 | GO:0043484 | 2.02E-03 | HNRNPL, SON, AHNAK, CELF1, FUS, HNRNPF, ZNF638, AHNAK2, HNRNPA1 |
| 41 | Ribosomal large subunit biogenesis | 6 | GO:0042273 | 2.49E-03 | RPL5, NPM1, NOP16, NIP7, MRTO4, RPF2 |
| 42 | Mitotic cytokinesis | 8 | GO:0000281 | 2.77E-03 | SON, ROCK2, ARL3, CFL1, STMN1, CHMP4B, TTC19, SPTBN1 |
| 43 | Protein deneddylation | 4 | GO:0000338 | 3.12E-03 | COPS4, COPS5, COPS7A, COPS8 |
| 44 | Regulation of translation | 9 | GO:0006417 | 3.17E-03 | FXR1, RBM8A, RPLP1, SARNP, FMR1, ELP1, ABCE1, QKI, EIF2A |
| 45 | DNA-dependent DNA replication | 6 | GO:0006261 | 3.22E-03 | PSMC6, PSMC4, PNKP, WDR18, POLD2, PSMC2 |
| 46 | Ribosomal large subunit assembly | 5 | GO:0000027 | 3.63E-03 | RPL5, BOP1, MRTO4, NOP2, RPF2 |
| 47 | Negative regulation of mRNA splicing, via spliceosome | 5 | GO:0048025 | 3.63E-03 | HNRNPL, C1QBP, ACIN1, SAP18, RNPS1 |
| 48 | Nucleocytoplasmic transport | 7 | GO:0006913 | 3.67E-03 | RANBP2, NPM1, ANP32A, NUP153, NUTF2, RAE1, NUP37 |
| 49 | NADP metabolic process | 4 | GO:0006739 | 4.07E-03 | PC, TP53I3, MDH1, IDH1 |
| 50 | Chaperone-mediated protein folding | 6 | GO:0061077 | 4.59E-03 | FKBP11, PEX19, TCP1, CHORDC1, CCT8, PDIA4 |
| 51 | Wnt signaling pathway, planar cell polarity pathway | 7 | GO:0060071 | 4.83E-03 | PSMA5, PSMB6, PSMA6, PSMB4, PSMB5, PSMB3, PSMA7 |
| 52 | SCF-dependent proteasomal ubiquitin-dependent protein catabolic process | 8 | GO:0031146 | 4.84E-03 | PSMA5, PSMB6, PSMA6, PSMB4, PSMB5, PSMB3, PSMA7, SKP1 |
| 53 | Regulation of protein stability | 9 | GO:0031647 | 5.43E-03 | SH3GLB1, ASPH, CD81, CASP3, LMNA, PIN1, TARDBP, QRSL1, DDOST |
| 54 | Maturation of 5.8S rRNA | 4 | GO:0000460 | 6.45E-03 | EXOSC10, EIF6, LAS1L, PRKDC |
| 55 | Response to hydrogen peroxide | 6 | GO:0042542 | 6.99E-03 | PRDX3, PDCD10, CASP3, CAT, SOD2, SOD1 |
| 56 | Nuclear pore localization | 3 | GO:0051664 | 7.52E-03 | FXR1, LMNA, LMNB1 |
| 57 | Regulation of protein neddylation | 4 | GO:2000434 | 7.90E-03 | COPS4, COPS5, COPS7A, COPS8 |
| 58 | Positive regulation of telomerase RNA localization to Cajal body | 4 | GO:1904874 | 7.90E-03 | TCP1, RUVBL1, NHP2, CCT8 |
| 59 | Microtubule polymerization | 4 | GO:0046785 | 9.52E-03 | ZNF207, MAP4, MAPRE1, CKAP5 |
| 60 | Mitochondrial calcium ion homeostasis | 4 | GO:0051560 | 9.52E-03 | LETM1, ANXA6, IMMT, MCU |
| 61 | DNA repair | 17 | GO:0006281 | 1.06E-02 | NPM1, FEN1, FH, UHRF1, PNKP, FMR1, ACTL6A, HMGB1, SMC1A, MACROH2A1, POLA1, TRIM28, ERCC3, POLD2, RUVBL1, EP400, HDGFL2 |
| 62 | Mature ribosome assembly | 3 | GO:0042256 | 1.11E-02 | EIF6, C1QBP, SBDS |
| 63 | MRNA destabilization | 4 | GO:0061157 | 1.13E-02 | FXR1, CELF1, ROCK2, METTL16 |
| 64 | Formation of cytoplasmic translation initiation complex | 4 | GO:0001732 | 1.13E-02 | EIF3M, EIF3K, EIF3F, EIF3C |
| 65 | Regulation of mitochondrial membrane potential | 5 | GO:0051881 | 1.21E-02 | PRDX3, PYCR1, NDUFS1, SOD2, SOD1 |
| 66 | Gluconeogenesis | 6 | GO:0006094 | 1.21E-02 | PC, TPI1, MDH1, MDH2, WDR5, PGK1 |
| 67 | Protein deubiquitination | 10 | GO:0016579 | 1.26E-02 | PSMA5, PSMB6, PSMA6, USP14, PSMB4, COPS5, PSMB5, PSMB3, EIF3F, PSMA7 |
| 68 | Positive regulation of telomere maintenance via telomerase | 5 | GO:0032212 | 1.49E-02 | PNKP, TCP1, CTNNB1, CCT8, HNRNPA1 |
| 69 | RNA catabolic process | 5 | GO:0006401 | 1.49E-02 | EXOSC6, EXOSC10, PPP1R8, HNRNPD, EXOSC2 |
| 70 | Arginine catabolic process | 3 | GO:0006527 | 1.52E-02 | DDAH1, DDAH2, FAH |
| 71 | Polyadenylation-dependent snoRNA 3'-end processing | 3 | GO:0071051 | 1.52E-02 | EXOSC6, EXOSC10, EXOSC2 |
| 72 | Positive regulation of mRNA binding | 3 | GO:1902416 | 1.52E-02 | HNRNPL, FMR1, EIF3C |
| 73 | Postsynaptic actin cytoskeleton organization | 4 | GO:0098974 | 1.55E-02 | FARP1, DBNL, DBN1, ACTB |
| 74 | T cell receptor signaling pathway | 10 | GO:0050852 | 1.58E-02 | PSMA5, PSMB6, PSMA6, PSMB4, PSMB5, PSMB3, UBE2N, PLCG1, PSMA7, CRKL |
| 75 | Protein polyubiquitination | 13 | GO:0000209 | 1.65E-02 | MGRN1, PSMA7, PSMA5, PSMB6, PSMA6, PSMB4, PSMB5, PSMB3, TRIM2, UBE2N, CTNNB1, BLMH, SKP1 |
| 76 | Ribosomal small subunit biogenesis | 7 | GO:0042274 | 1.70E-02 | RPS15, UTP15, NPM1, RPS28, EXOSC10, DNTTIP2, UTP20 |
| 77 | RNA export from nucleus | 4 | GO:0006405 | 1.78E-02 | ALYREF, NUP153, HNRNPA1, RAE1 |
| 78 | Cellular oxidant detoxification | 7 | GO:0098869 | 1.81E-02 | PRDX3, PRXL2A, GSR, PXDN, SELENOF, PRDX6, TXNDC17 |
| 79 | Actin cytoskeleton organization | 12 | GO:0030036 | 1.87E-02 | PDLIM1, SPECC1, TJP1, SDCBP, ROCK2, ACTN1, CFL1, FLNB, ACTN4, CORO2B, SPTBN1, ADD2 |
| 80 | One-carbon metabolic process | 5 | GO:0006730 | 1.99E-02 | DHFR, AHCYL1, MAT2A, MTHFD1, MTHFD1L |
| 81 | Localization | 5 | GO:0051179 | 1.99E-02 | UBA5, ANXA6, HNRNPA1, RAB6A, SRRM1 |
| 82 | Positive regulation of cell proliferation | 25 | GO:0008284 | 1.99E-02 | ITGB1, SMARCD1, CD81, NOP2, ACTB, CCAR1, CRKL, PRDX3, SDCBP, PDCD10, SSR1, ITGAV, NPM1, RPL23, ACTL6A, NAP1L1, MIF, PBX1, SMARCA4, TJP1, DLG1, PHIP, FAM98A, SLC25A5, LGMN |
| 83 | Ribosome assembly | 3 | GO:0042255 | 1.99E-02 | NPM1, NIP7, EIF2A |
| 84 | DNA strand elongation involved in DNA replication | 3 | GO:0006271 | 1.99E-02 | POLA1, MCM7, POLD2 |
| 85 | Inner mitochondrial membrane organization | 4 | GO:0007007 | 2.04E-02 | HSPA9, LETM1, SAMM50, IMMT |
| 86 | DNA replication | 9 | GO:0006260 | 2.15E-02 | POLA1, FEN1, MCM7, RBBP4, POLD2, NAP1L1, RBBP6, SSBP1, MCM6 |
| 87 | Negative regulation of canonical Wnt signaling pathway | 11 | GO:0090090 | 2.19E-02 | PSMA5, PSMB6, PSMA6, PSMB4, PSMB5, CDH2, PSMB3, CTNND1, CTNNB1, EMD, PSMA7 |
| 88 | Response to oxidative stress | 9 | GO:0006979 | 2.24E-02 | PDLIM1, PRDX3, PSMB5, ERCC3, PNKP, IDH1, PXDN, PSIP1, PRDX6 |
| 89 | Cell-cell adhesion | 12 | GO:0098609 | 2.29E-02 | PDLIM1, TJP1, DLG1, JUP, CDH2, CTNND1, CTNNB1, ITGA7, NRCAM, ITGAV, ITGA6, CD2AP |
| 90 | Histone H2A acetylation | 4 | GO:0043968 | 2.31E-02 | ACTL6A, RUVBL1, EP400, ACTB |
| 91 | Negative regulation of protein phosphorylation | 7 | GO:0001933 | 2.42E-02 | NIBAN1, SPAG9, CADM1, PRKDC, CHP1, TARDBP, CRKL |
| 92 | Oxaloacetate metabolic process | 3 | GO:0006107 | 2.51E-02 | ACLY, MDH1, MDH2 |
| 93 | Malate metabolic process | 3 | GO:0006108 | 2.51E-02 | FH, MDH1, MDH2 |
| 94 | Cell division | 19 | GO:0051301 | 2.59E-02 | DCTN1, GNAI3, SMC1A, CKAP5, SMC2, CD2AP, CDC37, ZFYVE19, EPB41L2, POGZ, RUVBL1, ZNF207, RCC1, BUB3, MAP4, MAPRE1, RAE1, ANAPC1, NUP37 |
| 95 | Protein neddylation | 4 | GO:0045116 | 2.60E-02 | COPS4, COPS5, COPS7A, COPS8 |
| 96 | Chromatin remodeling | 17 | GO:0006338 | 2.71E-02 | SMARCD1, NPM1, ACTL6A, PSIP1, DEK, BAZ1B, NUDT5, ACTB, SMARCA4, RBBP4, NTMT1, RUVBL1, EP400, HNRNPC, HDGFL2, SF3B1, SKP1 |
| 97 | Apoptotic process | 27 | GO:0006915 | 2.77E-02 | AVEN, PSMD10, MTCH1, STEAP3, AHCYL1, YARS1, CCAR1, FXR1, PRDX3, BCL2L13, PUF60, C1QBP, CASP3, NLRP2, KIF1B, SH3GLB1, TIGAR, TIA1, CADM1, CSNK2A2, SOD1, AIMP1, ERCC3, PTRH2, PDCD4, CLPTM1L, DAP3 |
| 98 | U2-type prespliceosome assembly | 4 | GO:1903241 | 2.91E-02 | SF3B2, SF3B6, SNRPB2, SF3B1 |
| 99 | Positive regulation of double-strand break repair | 5 | GO:2000781 | 3.02E-02 | SMARCD1, ACTL6A, UBE2N, ACTB, SMARCA4 |
| 100 | Protein insertion into ER membrane by stop-transfer membrane-anchor sequence | 3 | GO:0045050 | 3.08E-02 | EMC1, EMC10, EMC8 |
| 101 | Regulation of G0 to G1 transition | 4 | GO:0070316 | 3.24E-02 | SMARCD1, ACTL6A, ACTB, SMARCA4 |
| 102 | Positive regulation of transcription from RNA polymerase I promoter | 4 | GO:0045943 | 3.24E-02 | UTP15, DEK, BAZ1B, SF3B1 |
| 103 | Cell aging | 4 | GO:0007569 | 3.24E-02 | NPM1, PDCD4, MIF, SOD1 |
| 104 | Negative regulation of neuron differentiation | 6 | GO:0045665 | 3.43E-02 | SPAG9, ITGB1, LSM1, CNTN2, EIF2AK4, PBX1 |
| 105 | Positive regulation of cell migration | 14 | GO:0030335 | 3.58E-02 | ITGB1, SPAG9, HSPA5, ROCK2, ACTN4, SOD2, CCAR1, TJP1, SDCBP, PDCD10, ITGAV, ITGA6, CPNE3, EPHA2 |
| 106 | Fatty acid beta-oxidation using acyl-CoA dehydrogenase | 3 | GO:0033539 | 3.70E-02 | ACADVL, IVD, ETFA |
| 107 | CRD-mediated mRNA stabilization | 3 | GO:0070934 | 3.70E-02 | HNRNPD, IGF2BP3, IGF2BP2 |
| 108 | Response to UV | 5 | GO:0009411 | 3.76E-02 | RO60, ERCC3, CASP3, CAT, CIRBP |
| 109 | Regulation of nucleotide-excision repair | 4 | GO:2000819 | 3.96E-02 | SMARCD1, ACTL6A, ACTB, SMARCA4 |
| 110 | Sphingolipid metabolic process | 4 | GO:0006665 | 3.96E-02 | SPTLC1, AGK, TECR, PSAP |
| 111 | Positive regulation of DNA repair | 5 | GO:0045739 | 4.03E-02 | TIGAR, TRIM28, ACTL6A, RUVBL1, UBE2N |
| 112 | Negative regulation of apoptotic process | 23 | GO:0043066 | 4.06E-02 | AVEN, HSPA9, PSMD10, NPM1, HSPA5, PRKDC, MIF, SOD2, DHRS2, HYPK, TJP1, PRDX3, COPS5, SON, ARMC10, PDCD10, DDAH2, CASP3, CAT, CFL1, PDCD4, CTNNB1, PHIP |
| 113 | Microtubule cytoskeleton organization | 9 | GO:0000226 | 4.21E-02 | TUBB2B, TUBB6, SON, TBCE, DCLK2, CNTN2, MAP4, GAPDH, TUBA4A |
| 114 | Positive regulation of stem cell population maintenance | 5 | GO:1902459 | 4.31E-02 | SMARCD1, RBBP4, ACTL6A, ACTB, SMARCA4 |
| 115 | Leukocyte migration | 4 | GO:0050900 | 4.35E-02 | AIMP1, ITGA7, ITGA6, ADD2 |
| 116 | Histone monoubiquitination | 3 | GO:0010390 | 4.35E-02 | UHRF1, PAF1, RNF40 |
| 117 | Attachment of mitotic spindle microtubules to kinetochore | 3 | GO:0051315 | 4.35E-02 | CHAMP1, RMDN1, MAPRE1 |
| 118 | Positive regulation of apoptotic process | 16 | GO:0043065 | 4.55E-02 | ITGB1, MTCH1, PRKDC, HMGB1, CCAR1, SOD1, DNM2, ERCC3, C1QBP, CASP3, ACIN1, SAP18, CTNNB1, ITGA6, RNPS1, QRICH1 |
| 119 | Positive regulation of microtubule polymerization | 4 | GO:0031116 | 4.75E-02 | CLIP1, DCTN1, ARL3, MAPRE1 |
| 120 | Heterotypic cell-cell adhesion | 4 | GO:0034113 | 4.75E-02 | ITGB1, ITGA7, NRCAM, ITGAV |
| 121 | Heterochromatin assembly | 6 | GO:0031507 | 4.76E-02 | RBBP4, UHRF1, LMNA, HMGB1, MACROH2A1, LMNB1 |
| 122 | Cellular response to oxidative stress | 7 | GO:0034599 | 4.79E-02 | PRDX3, STAU1, GSR, PYCR1, ATP2A2, SOD2, DHRS2 |

**Supplementary Table S3:** List of all identified biological process with their protein count and protein involved particular biological processes, is analysed by Database for Annotation, Visualization and Integrated Discovery (DAVID) platform with a significant *p*-value ≤ 0.05 of differentially expressed proteins (upregulated and downregulated) identified by the high-resolution mass spectrometry (HRMS).
