# Supplementary material for: A protein–miRNA biomic analysis approach to explore neuroprotective potential of nobiletin in human neural progenitor cells (hNPCs)
Source: Front Pharmacol. 2024 Jan 25;15:1343569. doi: 10.3389/fphar.2024.1343569 (PMC10860404; doi:10.3389/fphar.2024.1343569)
Supplement: Supplementary file 8 [file Table4.DOCX]

**Supplementary Table S4**

**A. Up-regulated Proteins**

| **GO (Cellular component)** | | | | | |
| --- | --- | --- | --- | --- | --- |
| **S.No.** | **GO term** | **Count** | **GO: CC Term ID** | **P-value** | **Proteins (Up-regulated)** |
| 1 | Cytosol | 35 | GO:0005829 | 4.46E-07 | SMG1, PDXK, NUP205, CDCA2, NUP107, MVK, NUP188, GBE1, UAP1, PYGL, WASL, THTPA, BLOC1S6, FNTA, MFN2, TNKS1BP1, HMOX1, PSMF1, AASDHPPT, CDK5RAP3, GSTM3, KIDINS220, AGL, DENND4C, SURF4, MRPL28, MYO9B, DIAPH1, CDAN1, NEMF, XPOT, WNK1, CRYZL1, TIPRL, FERMT2 |
| 2 | Nuclear pore | 5 | GO:0005643 | 1.42E-04 | NUP205, NUP107, NUP188, NUP210, XPOT |
| 3 | Nuclear outer membrane | 4 | GO:0005640 | 7.10E-04 | NUP205, NUP107, NUP188, NUP210 |
| 4 | Nuclear inner membrane | 4 | GO:0005637 | 2.09E-03 | NUP205, NUP107, NUP188, NUP210 |
| 5 | Nuclear membrane | 5 | GO:0031965 | 5.98E-03 | NUP205, NUP107, NUP210, GNAQ, SURF4 |
| 6 | Nuclear pore inner ring | 2 | GO:0044611 | 8.41E-03 | NUP205, NUP188 |
| 7 | Cytoplasm | 25 | GO:0005737 | 1.60E-02 | SMG1, MVK, GBE1, NDUFA10, PYGL, WASL, BLOC1S6, FNTA, TNKS1BP1, PSMF1, NUDT16, CDK5RAP3, GSTM3, AGL, SURF4, MRPL28, MYO9B, RANBP6, ANKHD1, DIAPH1, CDAN1, XPOT, WNK1, GNAQ, FERMT2 |
| 8 | Nuclear envelope | 4 | GO:0005635 | 1.95E-02 | NUP205, NUP107, NUP188, NUP210 |
| 9 | Actin filament | 3 | GO:0005884 | 3.05E-02 | DIAPH1, MYO9B, WASL |
| 10 | Nuclear periphery | 2 | GO:0034399 | 4.14E-02 | NUP205, NUP107 |
| 11 | Secretory granule lumen | 3 | GO:0034774 | 4.16E-02 | PDXK, AGL, PYGL |
| 12 | Mitochondrial inner membrane | 5 | GO:0005743 | 4.68E-02 | NDUFA10, MRPL28, MRPS30, BCS1L, PPOX |

**B. Down-regulated Proteins**

| **GO (Cellular component)** | | | | | |
| --- | --- | --- | --- | --- | --- |
| **S.No.** | **GO term** | **Count** | **GO: CC Term ID** | **P-value** | **Proteins (Down-regulated)** |
| 1 | Cytosol | 304 | GO:0005829 | 2.46E-43 | EIF4A1, RPL5, RPL30, AHCYL1, RNH1, ECI2, RTCB, PREP, THUMPD3, NUDT5, ACTB, TXNDC17, SMC2, CRKL, RPS15, PSMD8, PCMT1, PSMD7, PSMD4, STMN1, LONP2, CPNE3, KIF21A, ATP6V1E1, CIAO2B, AGFG1, SKP1, ACTN1, RPL23, CSNK2A2, ACTN4, MIF, GAPVD1, SRRM1, CD2AP, CARHSP1, MTHFD1, CLNS1A, CDC37, PSME1, CD2BP2, HPRT1, TEX264, PRKDC, RPN1, TWF1, COPS7A, RPAP3, MYL12A, PRDX3, FXR1, LARP1, RAB11FIP1, PPP4C, PDCD10, EPB41L2, MYO6, PCBP2, VTI1A, MAP4, PLCG1, SPTBN1, DECR1, JUP, EIF2AK4, GPN1, PRDX6, AIMP1, HNRNPL, DHFR, POLA1, RPS28, EPRS1, EIF6, HNRNPF, GOLGB1, ECHDC1, HNRNPD, RNPS1, HNRNPC, FARSA, RPS21, ITPA, DAZAP1, GMEB2, RPLP1, SRP54, AKAP12, HINT1, PCM1, ZMYM2, C1QBP, RPLP2, UBXN7, UTP14A, ENOPH1, EMD, UBL7, CBR1, DBNL, TPI1, AKR1A1, NUTF2, PSMA5, PSMA6, VSNL1, PTRH2, TCP1, AGPS, PLIN3, UMPS, MAPRE1, PAFAH1B2, RAB7A, FH, STAU1, AHNAK, NIP7, RPL12, PSMA7, PSMB6, EXOSC6, LIMA1, PSMB4, PSMB5, EXOSC10, MTHFD1L, PSMB3, LMNA, FLNB, RPS2, CCT8, EMC8, EXOSC2, RPL17, RANBP2, FARP1, NPM1, TIGAR, PEX19, MDH1, AGK, GSR, ELP1, YLPM1, FAH, TJP1, ALDH4A1, PSMC6, PSMC4, PSMC2, TSR1, CTNNB1, NUCB2, FMR1, MPI, CTNND1, HDLBP, PEBP1, RPL10A, YARS1, CNDP2, EEF1B2, CFL1, POGZ, RUVBL1, ACAA1, AP2M1, SH3GLB1, TPM3, EIF1AX, RPSA, PPP4R3A, RNF40, TUBA4A, ACLY, CLIP1, SBDS, SMS, CHMP4B, AAGAB, PPIG, CARS1, ANAPC1, PSMD10, ISYNA1, PSMD11, RBM8A, PSMD13, UBA6, UBA5, KLC1, ACACA, NME1-NME2, SDCBP, TKFC, PGK1, NLRP2, RAB6A, ZC3H15, MPST, NIBAN1, SPECC1, TIA1, OSBPL6, HSPA5, IDH1, ALYREF, LSM1, NUP153, GFAP, COPS4, GRHPR, DLG1, COPS5, TP53I3, EIF2S3, ATG16L1, GNB2, SP3, UBE2N, PIN1, BLMH, ACO2, COPS8, EIF4G3, AVEN, MCM7, DDX42, PSIP1, DNPH1, GPHN, RO60, PPP6R1, NTMT1, CASP3, DBT, PPP6R3, PGM3, ITGAV, LRRFIP1, ACP1, TRIM65, ACTR3, ADSL, ELAC2, SMC1A, CKAP5, TSN, DNM2, SKIC8, DDAH1, DNAJC7, DDAH2, EEF1D, CAT, PFDN2, DNAJC9, GAPDH, DCTN6, USP14, GTF2A1, DCTN1, MGRN1, ROCK2, GMPR2, RAB1B, RPE, ADD3, ADD2, HMGCL, NXF1, MAT2A, FIP1L1, RBBP4, SAP18, BPNT2, RBBP6, IGF2BP3, IGF2BP2, BUB3, HNRNPA1, SPAG9, IGBP1, NIPSNAP3A, TXNL1, AHNAK2, DHODH, SOD1, GALE, EIF3M, PC, GNPDA2, EIF3K, XPNPEP1, RAB14, IMPDH2, PDCD4, ACIN1, SCLY, EIF3F, ABCE1, EIF3C, NUP37 |
| 2 | Extracellular exosome | 154 | GO:0070062 | 5.01E-29 | SCARB2, EIF4A1, RPL5, RPL30, AHCYL1, RNH1, CD81, CTNND1, MPI, PEBP1, RPL10A, NUDT5, ACTB, TXNDC17, SMC2, CNDP2, PCMT1, TUBB6, PSMD7, CFL2, CFL1, STMN1, RUVBL1, CHP1, CPNE3, DIP2B, FAM3C, ATP6V1E1, AP2M1, TPM3, CACNA2D1, ACTN1, RPL23, RPSA, ATP1B3, ACTN4, MIF, TUBA4A, CD2AP, ACLY, MTHFD1, CDC37, PSME1, SMS, CHMP4B, HPRT1, RAB5B, TTYH3, RAB5C, PEF1, ARL3, MACROH2A1, MYL12A, GLG1, SDCBP, TKFC, PDCD10, EPB41L2, PSAP, MYO6, PCBP2, PGK1, RAB6A, SPTBN1, MPST, NIBAN1, HSPA9, JUP, HSPA5, IDH1, ALYREF, PRDX6, HNRNPL, GRHPR, RPS28, DLG1, EIF2S3, EIF6, CPD, GNB2, UBE2N, BLMH, HNRNPC, COPS8, LGMN, ITGB1, LGALS3BP, ECE1, PEPD, DNPH1, HINT1, RNPEP, ANXA6, RPLP2, ITGAV, ACP1, ACTR3, CBR1, DBNL, TPI1, GAA, ATP6AP2, AKR1A1, NUTF2, DNM2, PSMA5, PSMA6, DDAH1, DNAJC7, DDAH2, TCP1, CAT, PXDN, GAPDH, PAFAH1B2, RAB7A, CRB2, USP14, ASAH1, FH, STAU1, AHNAK, MGRN1, RAB1B, RPE, RPL12, GNAI3, ATP1A1, PSMA7, PSMB6, PSMB4, PSMB5, SAMM50, PSMB3, FLNB, RPS2, CCT8, HNRNPA1, UGGT1, SPAG9, MDH1, MDH2, GSR, FAH, SOD2, SOD1, ERP44, PSMC6, XPNPEP1, RAB14, IMPDH2, CTNNB1, SSBP1, NUCB2 |
| 3 | Nucleoplasm | 206 | GO:0005654 | 3.03E-22 | RPL5, RNH1, GLDC, POP1, RTCB, MKI67, ACTB, SMC2, CRKL, CCAR1, RPS15, PSMD8, PSMD7, PSMD4, TRIM28, RCC1, CPNE3, CIAO2B, SKP1, SRRM2, RPL23, CSNK2A2, CIRBP, DNTTIP2, MIF, SRRM1, CLNS1A, SARNP, PSME1, CD2BP2, ANP32A, PRKDC, COPS7A, PRDX3, PPP4C, EPB41L2, MYO6, PCBP2, DECR1, GPN1, PBX1, SMARCA4, HNRNPL, POLA1, RPS28, EIF6, LAS1L, HNRNPF, HNRNPD, PAF1, RNPS1, HNRNPC, RPS21, ITPA, FEN1, DAZAP1, GMEB2, HINT1, PCM1, CHAMP1, UBXN7, NELFB, QSOX2, UTP14A, EMD, ACTL6A, NUTF2, BAZ1B, PSMA5, PSMA6, IVD, SF3B2, NIP7, SF3B6, SRRT, AKAP8, GNAI3, PSMA7, PSMB6, EXOSC6, PSMB4, PSMB5, EXOSC10, PSMB3, PPP1R8, LMNA, RPS2, CCT8, RAE1, EXOSC2, SF3B1, RANBP2, NPM1, PEX19, WDR18, YLPM1, DHRS2, HYPK, BOP1, PSMC6, PSMC4, PSMC2, TSR1, CTNNB1, TARDBP, FMR1, CNDP2, PPME1, POGZ, RUVBL1, ZNF207, MACROD1, RPSA, PPP4R3A, GTF2F2, RNF40, ACLY, TBL1XR1, NHP2, SBDS, SRSF3, PPIG, ANAPC1, DAP3, PSMD10, PSMD11, RBM8A, PSMD13, ARL3, NOP2, RPF2, MACROH2A1, SDCBP, EP400, UTP20, SPECC1, TIA1, CPSF7, NOP16, FUS, ALYREF, YJU2, NUP153, COPS4, COPS5, UBLCP1, SP3, UBE2N, PIN1, COPS8, ACADVL, RBM25, MCM7, CELF1, DDX42, PSIP1, HMGB1, LMNB1, RO60, NTMT1, CASP3, PPP6R3, WDR5, TRIM65, UTP15, ELAC2, SMC1A, TSN, SKIC8, DNAJC7, POLR1A, PEX3, EEF1D, ZNF638, DNAJC9, MCM6, GTF3C1, GTF2A1, SMARCD1, UHRF1, PNKP, PDHB, STRN3, NXF1, FIP1L1, RBBP4, PUF60, POLR2B, POLD2, SAP18, BPNT2, SNRPB2, BUB3, HNRNPA1, HNRNPA0, HNRNPA3, RPRD2, DEK, HNRNPAB, U2SURP, DHODH, SOD1, ERCC3, ACIN1, QRICH1, NUP37 |
| 4 | Cytoplasm | 256 | GO:0005737 | 6.43E-21 | EIF4A1, RPL5, RPL30, AHCYL1, RNH1, CLPB, RTCB, PREP, THUMPD3, ACTB, SMC2, CCAR1, RPS15, PCMT1, LSM12, UBASH3B, STMN1, RCC1, CPNE3, KIF21A, DIP2B, SEPHS1, CIAO2B, AGFG1, SKP1, EIF2A, ABHD14A-ACY1, ACTN1, RPL23, CIRBP, ACTN4, MIF, CD2AP, CARHSP1, CDC37, ZFYVE19, PSME1, SUCLG2, CD2BP2, HPRT1, EXD2, ANP32A, TWF1, COPS7A, MYL12A, PRDX3, FXR1, LARP1, PPP4C, PDCD10, MYO6, PCBP2, MAP4, PLCG1, SPTBN1, JUP, DCLK2, RMDN1, GPN1, PRDX6, MRPL21, PBX1, HNRNPL, EEF1E1-BLOC1S5, RPS28, EPRS1, EIF6, PRXL2A, HNRNPD, PAF1, RNPS1, FARSA, RPS21, ITPA, LGMN, RPLP1, SRP54, AKAP12, HINT1, PCM1, C1QBP, TRIM2, SH3PXD2B, ANXA6, CHAMP1, RPLP2, KIF1B, NELFB, EMD, DBNL, ZFR, PSMA5, PSMA6, TUBB2B, PLIN3, UMPS, DBN1, PAFAH1B2, CRB2, FH, STAU1, AHNAK, RPL12, SRRT, GNAI3, PSMA7, PSMB6, PSMB4, PSMB5, EXOSC10, PSMB3, PPP1R8, FLNB, RPS2, CCT8, EMC8, RAE1, EXOSC2, RPL17, RANBP2, NPM1, TIGAR, MYEF2, PEX19, MDH1, MDH2, AGK, ELP1, DHRS2, HYPK, TJP1, PSMC6, PSMC2, CTNNB1, SCARB2, STEAP3, FMR1, CTNND1, HDLBP, RPL10A, YARS1, EEF1B2, TUBB6, CDH2, CFL2, CFL1, POGZ, ZNF207, SACS, CHP1, SH3GLB1, EIF1AX, RPSA, TUBA4A, ACLY, CLIP1, MRTO4, SBDS, SRSF3, CHMP4B, AAGAB, PPIG, CARS1, HDGFL2, PSMD10, LRRC59, ISYNA1, RBM8A, PEF1, UBA6, UBA5, ARL3, KLC1, SDCBP, NLRP2, UTP20, HSPA9, NIBAN1, TIA1, CPSF7, HSPA5, FUS, IDH1, ALYREF, LSM1, CORO2B, QKI, GFAP, COPS4, GRHPR, DLG1, COPS5, EIF2S3, GNB2, TBCE, UBE2N, PIN1, IFT27, NACAD, BLMH, COPS8, ITGB1, RBM25, CELF1, DDX42, GPHN, RO60, NTMT1, CASP3, DBT, PPP6R3, LRRFIP1, METTL16, ACP1, TRIM65, LBR, UTP15, ACTR3, CKAP5, TSN, DNM2, SKIC8, DNAJC7, EEF1D, ZNF638, CAT, PFDN2, DNAJC9, SEC22B, GAPDH, DCTN6, DCTN1, MGRN1, ROCK2, STRN3, PDLIM1, NXF1, IGF2BP3, IGF2BP2, HNRNPA1, SPAG9, IGBP1, HNRNPA3, TXNL1, AHNAK2, NAP1L1, HNRNPAB, SOD1, PC, GNPDA2, XPNPEP1, IMPDH2, PDCD4, QRICH1, FAM98A, ABCE1 |
| 5 | Proteasome complex | 19 | GO:0000502 | 7.39E-15 | PSMD10, USP14, PSMD11, PSMD13, TXNL1, PSMA7, PSMD8, PSMA5, PSMB6, PSMA6, PSMB4, PSMC6, PSMD7, PSMB5, PSMD4, PSMC4, PSMB3, PSMC2, PSME1 |
| 6 | Mitochondrion | 92 | GO:0005739 | 3.82E-14 | MIPEP, MTCH1, ACADVL, FEN1, CLPB, GLDC, ECI2, ETFA, MRPL40, NNT, C1QBP, DBT, SACS, ANXA6, FDXR, PMPCA, KIF1B, CPNE3, MIX23, ELAC2, HDHD5, MRPL45, QRSL1, HADHB, SARS2, MTHFD1, DDAH2, IVD, PTRH2, CAT, AGPS, UQCRC1, SUCLG2, PFDN2, SUCLG1, NDUFS1, SLC25A5, MRRF, DAP3, RAB7A, ATPAF1, EXD2, FH, ABCB7, PNKP, AKAP8, SPG7, PDHB, MFF, ACACA, PRDX3, PSMB6, HMGCL, BCL2L13, SDCBP, PSMB4, RHOT2, SAMM50, MTHFD1L, DECR1, MPST, RANBP2, HSPA9, PDHA1, NIPSNAP3A, HSPA5, AGK, UQCC2, MDH2, IDH1, GSR, PYCR1, IMMT, RMDN1, SOD2, GPN1, DHRS2, PRDX6, TTC19, MRPL21, DHODH, SOD1, DHFR, ALDH4A1, LETM1, PC, ARMC10, ACO2, SSBP1, TARDBP, ABCE1, MCU |
| 7 | Focal adhesion | 44 | GO:0005925 | 1.30E-13 | ITGB1, SCARB2, RPL5, RPL30, AHNAK, CD81, RPLP1, RPL12, TWF1, RPL10A, ACTB, RPS15, PDLIM1, AKAP12, LIMA1, SDCBP, CDH2, EPB41L2, PCBP2, CFL1, ANXA6, RPLP2, CHP1, FLNB, ITGAV, CPNE3, RPS2, ACTR3, HSPA9, NPM1, JUP, HSPA5, RPL23, ACTN1, ACTN4, CORO2B, DNM2, PTK7, GNB2, CAT, CTNNB1, ITGA6, MAPRE1, EPHA2 |
| 8 | Nucleus | 243 | GO:0005634 | 3.92E-13 | RPL5, RPL30, RTCB, PREP, MKI67, NUDT5, ACTB, SMC2, CCAR1, RPS15, PSMD8, PSMD7, PSMD4, TRIM28, UBASH3B, RCC1, LONP2, CPNE3, DIP2B, CIAO2B, SKP1, SRRM2, ACTN1, CSNK2A2, CIRBP, ACTN4, SRRM1, CLNS1A, SARNP, CD2BP2, EXD2, ANP32A, TEX264, PRKDC, COPS7A, FXR1, PPP4C, MYO6, PCBP2, SPTBN1, DECR1, JUP, SENP3-EIF4A1, GPN1, PRDX6, PBX1, SMARCA4, AIMP1, HNRNPL, POLA1, EIF6, HNRNPF, HNRNPD, RNPS1, HNRNPC, PHF3, FEN1, DAZAP1, GMEB2, TECR, SRP54, HINT1, ZMYM2, MRPL40, C1QBP, CHAMP1, UBXN7, NELFB, ENOPH1, TPI1, ACTL6A, ZFR, BAZ1B, GNL3, PSMA5, PSMA6, TUBB2B, UMPS, FH, SF3B2, ASAH1, AHNAK, SPINDOC, SF3B6, SRRT, AKAP8, PSMA7, PSMB6, EXOSC6, PSMB4, PSMB5, EXOSC10, PSMB3, PPP1R8, LMNA, RPS2, RAE1, EXOSC2, SF3B1, RPL17, NPM1, TIGAR, MYEF2, PEX19, MDH2, ELP1, YLPM1, HNRNPUL2-BSCL2, DHRS2, HYPK, TJP1, PSMC6, PSMC4, PSMC2, TSR1, CTNNB1, TARDBP, FMR1, CTNND1, HDLBP, PEBP1, RPL10A, YARS1, TUBB6, CFL1, POGZ, RUVBL1, ZNF207, SACS, MACROD1, CHP1, RPSA, GTF2F2, RNF40, CLIP1, TBL1XR1, DNAJB11, SBDS, CHMP4B, PHIP, PPIG, HDGFL2, PSMD10, PSMD11, RBM8A, PSMD13, UBA6, UBA5, ARL3, MACROH2A1, BCL2L13, SDCBP, TKFC, EP400, ZC3H15, TIA1, CPSF7, PDHA1, HSPA5, FUS, ALYREF, LSM1, QKI, COPS4, DLG1, COPS5, UBLCP1, SP3, UBE2N, PIN1, IFT27, NACAD, BLMH, COPS8, MCM7, CELF1, DDX42, PSIP1, HMGB1, DNPH1, LMNB1, PPP6R1, NTMT1, CASP3, PPP6R3, WDR5, LRRFIP1, METTL16, LBR, ACTR3, ELAC2, SMC1A, TSN, DNM2, SKIC8, POLR1A, EEF1D, ZNF638, PFDN2, DNAJC9, MCM6, SLC25A5, GAPDH, GTF2A1, SMARCD1, DCTN1, MGRN1, ROCK2, UHRF1, PNKP, PDHB, STRN3, NXF1, RBBP4, PUF60, POLR2B, POLD2, SAP18, RBBP6, IGF2BP3, SNRPB2, IGF2BP2, HNRNPA1, HNRNPA0, HNRNPA3, NIPSNAP3A, TXNL1, AHNAK2, NAP1L1, DEK, HNRNPAB, U2SURP, SOD1, ERCC3, GNPDA2, EIF3K, IMPDH2, PDCD4, ACIN1, QRICH1, SSBP1, FAM98A, NUP37 |
| 9 | Mitochondrial matrix | 38 | GO:0005759 | 4.37E-11 | MIPEP, EXD2, FH, ACADVL, GLDC, ETFA, PDHB, PRDX3, HMGCL, MTHFD1L, C1QBP, DBT, FDXR, PMPCA, DECR1, MPST, ACAD8, HSPA9, PDHA1, UQCC2, MDH2, ELAC2, GSR, PYCR1, SOD2, DHRS2, SOD1, ALDH4A1, SARS2, PC, IVD, SUCLG2, SUCLG1, NDUFS1, SSBP1, ACO2, ABCE1, MRRF |
| 10 | Ribonucleoprotein complex | 26 | GO:1990904 | 5.17E-11 | GTF3C1, RPL5, DAZAP1, CELF1, FMR1, SRRT, ACTB, RO60, PUF60, RUVBL1, PCBP2, HNRNPA1, HNRNPA0, NPM1, HNRNPA3, MYEF2, LSM1, ACTN4, HNRNPAB, HNRNPL, BOP1, EPRS1, HNRNPF, NHP2, HNRNPD, GAPDH |
| 11 | Membrane | 158 | GO:0016020 | 4.25E-10 | SCARB2, EIF4A1, MTCH1, RPL5, RPL30, CD81, FMR1, ECI2, PREP, EMC10, RPL10A, MKI67, ACTB, TMEM263, RPS15, PSMD7, CFL1, STMN1, RUVBL1, LONP2, DIP2B, PLXNC1, ACAA1, SH3GLB1, RPL23, RPSA, RNF40, ACLY, MTHFD1, DNAJB11, LRRC59, RAB5B, PSMD11, PSMD13, PRKDC, RPN1, KLC1, GLG1, FXR1, BCL2L13, SDCBP, RHOT2, LARP1, RAB11FIP1, MYO6, PCBP2, PGK1, MLEC, SSR1, RAB6A, NIBAN1, SPECC1, CPSF7, OSBPL6, HSPA5, ALYREF, NUP153, PRDX6, CORO2B, SMARCA4, AIMP1, HNRNPL, DLG1, EPRS1, LAS1L, PTK7, CPD, HNRNPF, GNB2, GOLGB1, CALU, PAF1, HNRNPC, CLPTM1L, FARSA, AVEN, FKBP11, ITGB1, LGALS3BP, FEN1, FKBP15, MCM7, CELF1, LPCAT1, DDX42, ATP2A2, MIA3, ECE1, LMNB1, HS2ST1, PCM1, NNT, C1QBP, ANXA6, RPLP2, ITGAV, LBR, EMD, ACTR3, DBNL, GAA, ATP6AP2, CKAP5, DDOST, GNL3, DNM2, VSNL1, DNAJC7, PEX3, PTRH2, CAT, AGPS, PLIN3, NDUFS1, SLC25A5, SEC22B, GAPDH, GTF3C1, ATL3, STAU1, AHNAK, DCTN1, MGRN1, PNKP, RPL12, AKAP8, GNAI3, RRBP1, ATP1A1, ADD3, STRN3, EXOSC10, SAMM50, MTHFD1L, POLR2B, EMC1, BPNT2, FLNB, RPS2, HNRNPA1, EMC8, RANBP2, NPM1, MDH2, AGK, PCDHGC3, IMMT, NAP1L1, KTN1, DHRS7, PSMC6, EIF3K, PSMC4, IMPDH2, PSMC2, CTNNB1, EIF3F, ABCE1 |
| 12 | Ficolin-1-rich granule lumen | 16 | GO:1904813 | 1.31E-06 | ASAH1, PSMD11, DBNL, JUP, PSMD13, IDH1, HMGB1, MIF, PSMA5, ACLY, PSMD7, IMPDH2, PSMC2, CAT, CCT8, PAFAH1B2 |
| 13 | Nuclear matrix | 16 | GO:0016363 | 1.77E-06 | SMARCD1, UHRF1, ACTL6A, AKAP8, SMC1A, ACTB, LMNB1, SMARCA4, SRRM1, POLA1, PSMA6, CFL2, LMNA, CFL1, RUVBL1, PPIG |
| 14 | Mitochondrial nucleoid | 10 | GO:0042645 | 3.24E-06 | HSPA9, HADHB, LRRC59, ACADVL, UQCC2, ELAC2, DBT, SSBP1, SLC25A5, SOD2 |
| 15 | Proteasome accessory complex | 7 | GO:0022624 | 3.63E-06 | PSMD8, PSMD11, PSMC6, PSMD4, PSMD13, PSMC4, PSMC2 |
| 16 | Cytosolic ribosome | 13 | GO:0022626 | 4.25E-06 | RPL5, RPL30, RPLP1, RPL23, RPL12, RPSA, EIF2AK4, RPL10A, RPS15, RPS2, ABCE1, RPS21, RPL17 |
| 17 | Macromolecular complex | 41 | GO:0032991 | 4.50E-06 | CRB2, RPL5, DAZAP1, FEN1, PRKDC, SRRT, ATP1A1, MFF, ACTB, STRN3, CRKL, PRDX3, HMGCL, PCM1, RBBP4, TRIM28, EMC1, RCC1, RBBP6, SLC12A7, UGGT1, SH3GLB1, NPM1, ADSL, PEX19, HSPA5, RPL23, ACTL6A, ACTN4, CKAP5, HYPK, SMARCA4, SOD1, DNM2, TJP1, EIF3M, PEX3, CAT, UBE2N, CTNNB1, HNRNPC |
| 18 | Intracellular non-membrane-bounded organelle | 7 | GO:0043232 | 1.45E-05 | FXR1, FUS, FMR1, FLNB, HNRNPA1, TARDBP, EIF4G3 |
| 19 | Proteasome core complex | 7 | GO:0005839 | 1.45E-05 | PSMA5, PSMB6, PSMA6, PSMB4, PSMB5, PSMB3, PSMA7 |
| 20 | Nuclear speck | 29 | GO:0016607 | 2.23E-05 | RBM25, SF3B2, RBM8A, SRP54, DDX42, NXF1, PPP1R8, LMNA, EP400, SAP18, SNRPB2, RBBP6, SF3B1, SRRM2, ALYREF, YLPM1, PPP4R3A, SRRM1, COPS4, SON, ZNF638, SARNP, ACIN1, SRSF3, PIN1, CD2BP2, AAGAB, RNPS1, PPIG |
| 21 | Melanosome | 13 | GO:0042470 | 2.42E-05 | ITGB1, RAB5B, RAB5C, HSPA5, RPN1, NAP1L1, ATP1B3, ATP1A1, PDIA4, SDCBP, CALU, ANXA6, SEC22B |
| 22 | Catalytic step 2 spliceosome | 12 | GO:0071013 | 3.05E-05 | SRRM2, SF3B2, HNRNPA3, RBM8A, HNRNPF, SF3B6, ALYREF, SNRPB2, HNRNPC, HNRNPA1, SF3B1, SRRM1 |
| 23 | Secretory granule lumen | 13 | GO:0034774 | 6.59E-05 | PSMD11, DBNL, PSMD13, IDH1, HMGB1, MIF, PSMA5, PSMD7, IMPDH2, PSMC2, CAT, CCT8, PAFAH1B2 |
| 24 | Condensed chromosome | 7 | GO:0000793 | 8.57E-05 | CHAMP1, AKAP8, HMGB1, MKI67, BAZ1B, MACROH2A1, SMC2 |
| 25 | Nuclear envelope | 17 | GO:0005635 | 1.34E-04 | RANBP2, LRRC59, DCTN1, RTCB, NUP153, DHRS2, C12ORF43, LMNB1, FXR1, POLA1, LMNA, CHMP4B, RAE1, LBR, EMD, NUCB2, NUP37 |
| 26 | Peroxisome | 12 | GO:0005777 | 2.09E-04 | HMGCL, PEX19, PEX3, IDH1, ECI2, AGPS, CAT, LONP2, DHRS4L2, MFF, ACAA1, SOD1 |
| 27 | Cytoplasmic stress granule | 11 | GO:0010494 | 2.46E-04 | FXR1, NXF1, TIA1, LARP1, STAU1, CELF1, FMR1, CIRBP, IGF2BP3, IGF2BP2, TARDBP |
| 28 | Cytosolic large ribosomal subunit | 9 | GO:0022625 | 2.52E-04 | RPL5, RPL30, RPLP1, RPL23, RPL12, NHP2, RPLP2, RPL10A, RPL17 |
| 29 | Nucleolus | 61 | GO:0005730 | 3.79E-04 | RPL5, ACADVL, FEN1, POP1, FMR1, THUMPD3, MKI67, SMC2, MRPL40, C1QBP, ZNF207, CPNE3, UTP14A, UTP15, RPL23, DNTTIP2, BAZ1B, CKAP5, GNL3, RCN2, WDR82, MRTO4, AGPS, SBDS, NHP2, PAFAH1B2, GTF3C1, PRKDC, PNKP, NIP7, RPL12, NOP2, AKAP8, GNAI3, TWF1, RPF2, MACROH2A1, FXR1, EXOSC6, EXOSC10, RBBP6, UTP20, SF3B1, EXOSC2, SPTBN1, HSPA9, NPM1, PDHA1, NOP16, WDR18, NUP153, DEK, SMARCA4, POLA1, BOP1, RPS28, LAS1L, UBLCP1, EIF6, TSR1, ACIN1 |
| 30 | Endoplasmic reticulum | 52 | GO:0005783 | 4.15E-04 | FKBP11, RPL5, TECR, LPCAT1, SRP54, ATP2A2, EMC10, TXNDC12, HMGB1, EEF1B2, SPTLC1, CHP1, EMD, UTP15, SEZ6L, TSN, DDOST, PDIA4, HADHB, RCN3, RCN1, RCN2, TMX3, ASPH, EEF1D, PEX3, DNAJB11, PXDN, CAT, LDAH, LRRC59, ANP32A, ATL3, STAU1, PEF1, TEX264, MGRN1, RPN1, RRBP1, ATP1A1, SERPINH1, MLEC, SSR1, UGGT1, EMC8, HSPA5, KTN1, AIMP1, DLG1, ARMC10, CALU, NUCB2 |
| 31 | Kinetochore | 14 | GO:0000776 | 4.42E-04 | DCTN6, SMARCD1, DCTN1, ACTL6A, SMC1A, CKAP5, ACTB, SMARCA4, CLIP1, CHAMP1, ZNF207, CHMP4B, BUB3, NUP37 |
| 32 | Peroxisomal matrix | 8 | GO:0005782 | 4.71E-04 | HMGCL, GRHPR, IDH1, ECI2, AGPS, CAT, LONP2, ACAA1 |
| 33 | Eukaryotic translation initiation factor 3 complex | 5 | GO:0005852 | 7.40E-04 | EIF3M, COPS5, EIF3K, EIF3F, EIF3C |
| 34 | COP9 signalosome | 5 | GO:0008180 | 9.47E-04 | COPS4, COPS5, COPS7A, PLCG1, COPS8 |
| 35 | Actin cytoskeleton | 18 | GO:0015629 | 9.61E-04 | ACTR3, FKBP15, JUP, AHNAK, TPM3, TWF1, ACTN4, CORO2B, ACTB, ACACA, CD2AP, LIMA1, CFL2, MYO6, CFL1, FLNB, HNRNPC, DBN1 |
| 36 | Small ribosomal subunit | 6 | GO:0015935 | 9.80E-04 | RPS15, RPS28, RPSA, RPS2, RPS21, DAP3 |
| 37 | Chaperone complex | 6 | GO:0101031 | 1.15E-03 | CDC37, DNAJB11, RUVBL1, PFDN2, DNAJC9, RPAP3 |
| 38 | Eukaryotic 48S preinitiation complex | 5 | GO:0033290 | 1.19E-03 | EIF3M, EIF3K, EIF1AX, EIF3F, EIF3C |
| 39 | Endoplasmic reticulum-Golgi intermediate compartment | 9 | GO:0005793 | 1.42E-03 | ERP44, HSPA5, GOLGB1, SERPINH1, CHP1, HMGB1, SEC22B, UGGT1, NUCB2 |
| 40 | Proteasome regulatory particle, base subcomplex | 4 | GO:0008540 | 1.46E-03 | PSMC6, PSMD4, PSMC4, PSMC2 |
| 41 | Eukaryotic 43S preinitiation complex | 5 | GO:0016282 | 1.48E-03 | EIF3M, EIF3K, EIF1AX, EIF3F, EIF3C |
| 42 | Postsynaptic density | 17 | GO:0014069 | 1.99E-03 | RPL30, HNRNPA3, DBNL, CADM1, RPL12, FMR1, ATP1A1, ADD3, GPHN, ADD2, FXR1, SDCBP, CDH2, PCBP2, HNRNPD, SPTBN1, DBN1 |
| 43 | Fascia adherens | 4 | GO:0005916 | 2.04E-03 | JUP, CDH2, ACTN1, CTNNB1 |
| 44 | Cell-cell junction | 14 | GO:0005911 | 2.13E-03 | ACTR3, JUP, CADM1, ACTN1, CTNND1, TWF1, ADD3, ACTB, CD2AP, DLG1, CDH2, PTK7, CTNNB1, PLCG1 |
| 45 | Ribosome | 13 | GO:0005840 | 2.42E-03 | RPL5, RPLP1, RPL23, RRBP1, RPL10A, MRPL45, MRPL21, RPS15, RPS28, TBCE, RPS2, RPS21, DAP3 |
| 46 | Mitochondrial intermembrane space | 9 | GO:0005758 | 2.61E-03 | TIMM8B, MIX23, CLPB, AGK, UQCC2, CAT, IMMT, NDUFS1, SOD1 |
| 47 | Preribosome, large subunit precursor | 5 | GO:0030687 | 3.10E-03 | BOP1, EIF6, LAS1L, NIP7, MRTO4 |
| 48 | Mitochondrial inner membrane | 25 | GO:0005743 | 3.25E-03 | SYNJ2BP-COX16, ACADVL, ABCB7, SPG7, COX5A, MRPL40, NNT, FDXR, PMPCA, RDH13, TIMM8B, AGK, UQCC2, IMMT, MRPL45, TTC19, MRPL21, DHODH, HADHB, LETM1, UQCRC1, NDUFS1, SLC25A5, MCU, DAP3 |
| 49 | Microtubule | 19 | GO:0005874 | 3.37E-03 | DCTN1, KLC1, TUBA4A, DNM2, GOLGA2, TUBB6, CLIP1, DLG1, TUBB2B, TCP1, STMN1, TBCE, ZNF207, KIF21A, KIF1B, MAP4, CCT8, MAPRE1, EMD |
| 50 | Fibrillar center | 12 | GO:0001650 | 3.48E-03 | UTP15, SPECC1, EEF1D, UBE2N, CD2BP2, PAF1, SNRPB2, RAE1, PAFAH1B2, ACACA, SMARCA4, CD2AP |
| 51 | Proteasome core complex, beta-subunit complex | 4 | GO:0019774 | 3.59E-03 | PSMB6, PSMB4, PSMB5, PSMB3 |
| 52 | Apicolateral plasma membrane | 5 | GO:0016327 | 3.64E-03 | CRB2, TJP1, JUP, CDH2, CTNNB1 |
| 53 | ASAP complex | 3 | GO:0061574 | 4.21E-03 | ACIN1, SAP18, RNPS1 |
| 54 | Chromosome | 16 | GO:0005694 | 4.27E-03 | FH, MCM7, FMR1, ZFR, DNTTIP2, HMGB1, MKI67, SMC1A, RPF2, SMC2, GNL3, BOP1, POLR1A, RCC1, RBBP6, MCM6 |
| 55 | Cytoplasmic exosome (RNAse complex) | 4 | GO:0000177 | 5.71E-03 | EXOSC6, CARHSP1, EXOSC10, EXOSC2 |
| 56 | Npbaf complex | 4 | GO:0071564 | 5.71E-03 | SMARCD1, ACTL6A, ACTB, SMARCA4 |
| 57 | Lamellipodium | 13 | GO:0030027 | 6.12E-03 | ITGB1, ACTR3, RNH1, DBNL, CTNND1, ACTB, DNM2, CDH2, CFL1, CTNNB1, PLCG1, DBN1, EPHA2 |
| 58 | Microtubule cytoskeleton | 13 | GO:0015630 | 6.12E-03 | DCTN1, ARL3, CKAP5, GTF2F2, HYPK, TUBA4A, TUBB6, CLIP1, TUBB2B, DBT, CHP1, MAP4, GAPDH |
| 59 | Lamin filament | 3 | GO:0005638 | 6.90E-03 | EIF6, LMNA, LMNB1 |
| 60 | Messenger ribonucleoprotein complex | 4 | GO:1990124 | 7.00E-03 | HNRNPA3, STAU1, FMR1, HNRNPAB |
| 61 | RSC complex | 4 | GO:0016586 | 8.44E-03 | SMARCD1, ACTL6A, ACTB, SMARCA4 |
| 62 | Cytosolic small ribosomal subunit | 6 | GO:0022627 | 8.50E-03 | RPS15, RPS28, RPSA, RPS2, RPS21, EIF2A |
| 63 | Cytoplasmic ribonucleoprotein granule | 8 | GO:0036464 | 9.75E-03 | FXR1, STAU1, ROCK2, SARNP, FMR1, PSMC2, SNRPB2, ACTB |
| 64 | Transcriptional preinitiation complex | 4 | GO:0097550 | 1.00E-02 | GTF2A1, ERCC3, RPRD2, GTF2F2 |
| 65 | Spliceosomal complex | 10 | GO:0005681 | 1.02E-02 | SF3B2, CLNS1A, SF3B6, PPP1R8, CIRBP, SNRPB2, HNRNPC, HNRNPA1, SF3B1, SRRM1 |
| 66 | Polysomal ribosome | 5 | GO:0042788 | 1.04E-02 | RPS28, RPL30, LARP1, RPL10A, RPS21 |
| 67 | Nuclear membrane | 15 | GO:0031965 | 1.08E-02 | RANBP2, OSBPL6, NUP153, NUTF2, LMNB1, SDCBP, PCM1, LMNA, MYO6, RCC1, QSOX2, SEPHS1, GAPDH, LBR, EMD |
| 68 | Nuclear inner membrane | 8 | GO:0005637 | 1.10E-02 | RANBP2, NUP153, NUTF2, RAE1, LBR, EMD, LMNB1, NUP37 |
| 69 | Midbody | 12 | GO:0030496 | 1.30E-02 | SH3GLB1, HSPA5, ARL3, ZFYVE19, CTNND1, GNAI3, CHMP4B, PIN1, CIAO2B, TTC19, GNL3, DNM2 |
| 70 | Autophagosome membrane | 6 | GO:0000421 | 1.40E-02 | SH3GLB1, TEX264, ATG16L1, ATP6AP2, CHMP4B, RAB7A |
| 71 | Cytoskeleton | 25 | GO:0005856 | 1.46E-02 | PSMD10, ROCK2, TWF1, ADD3, GPHN, GLG1, ACTB, ADD2, PDLIM1, AKAP12, SDCBP, HINT1, EPB41L2, IGF2BP2, LRRFIP1, FARP1, DBNL, JUP, TPM3, DCLK2, TUBA4A, DNAJC7, CLNS1A, TBCE, DBN1 |
| 72 | Small-subunit processome | 7 | GO:0032040 | 1.49E-02 | UTP15, RPS28, EXOSC10, PRKDC, DNTTIP2, UTP20, UTP14A |
| 73 | Glutamatergic synapse | 20 | GO:0098978 | 1.69E-02 | ITGB1, FARP1, HNRNPA3, DBNL, STAU1, ACTN1, CTNND1, ACTB, DNM2, FXR1, DLG1, C1QBP, HNRNPD, PIN1, CTNNB1, NRCAM, PLCG1, AP2M1, SPTBN1, DBN1 |
| 74 | Proteasome regulatory particle, lid subcomplex | 3 | GO:0008541 | 1.83E-02 | PSMD8, PSMD11, PSMD13 |
| 75 | Ruffle | 8 | GO:0001726 | 1.87E-02 | ITGB1, LIMA1, CLIP1, DBNL, ACTN1, MYO6, PLCG1, CD2AP |
| 76 | Polysome | 5 | GO:0005844 | 1.88E-02 | FXR1, PSMA6, FMR1, HDLBP, EIF2AK4 |
| 77 | Ruffle membrane | 8 | GO:0032587 | 1.97E-02 | ITGB1, CFL1, MYO6, TWF1, ITGAV, PLCG1, EPHA2, DNM2 |
| 78 | Azurophil granule membrane | 6 | GO:0035577 | 2.00E-02 | RAB5C, GAA, PSAP, LPCAT1, CPNE3, DDOST |
| 79 | Endoplasmic reticulum lumen | 16 | GO:0005788 | 2.03E-02 | HSPA5, COL11A1, SELENOF, TXNDC12, MIA3, PDIA4, KTN1, RCN3, ERP44, RCN1, RCN2, CDH2, DNAJB11, SERPINH1, CALU, UGGT1 |
| 80 | Chromosome, telomeric region | 11 | GO:0000781 | 2.08E-02 | FEN1, MCM7, RBBP4, POLR2B, PRKDC, WDR82, ALYREF, NHP2, NLRP2, MCM6, MACROH2A1 |
| 81 | Vesicle | 11 | GO:0031982 | 2.31E-02 | AHNAK, CD81, GNB2, CFL1, MYO6, CHMP4B, ECE1, MIF, GAPDH, ACTB, CD2AP |
| 82 | Euchromatin | 6 | GO:0000791 | 2.43E-02 | SKIC8, EXOSC10, TRIM28, UHRF1, PSIP1, CTNNB1 |
| 83 | Cortical actin cytoskeleton | 6 | GO:0030864 | 2.43E-02 | DBNL, CDH2, ACTN1, ACTN4, SPTBN1, DBN1 |
| 84 | Nuclear outer membrane | 6 | GO:0005640 | 2.59E-02 | RANBP2, NUP153, NUTF2, RAE1, EMD, NUP37 |
| 85 | Sarcolemma | 8 | GO:0042383 | 2.60E-02 | ITGB1, DLG1, AHNAK, CDH2, AHNAK2, ANXA6, ATP1A1, ACP1 |
| 86 | Microtubule plus-end | 4 | GO:0035371 | 2.60E-02 | CLIP1, DCTN1, MAPRE1, CKAP5 |
| 87 | Cell junction | 13 | GO:0030054 | 2.80E-02 | ACTN1, ACTN4, COPS4, TJP1, DLG1, CDH2, PUF60, EPB41L2, SH3PXD2B, CTNNB1, PAF1, CPNE3, SPTBN1 |
| 88 | Protein-DNA complex | 5 | GO:0032993 | 2.83E-02 | NPM1, JUP, PRKDC, SP3, CTNNB1 |
| 89 | Proteasome regulatory particle | 3 | GO:0005838 | 2.84E-02 | PSMD8, PSMD7, PSMD13 |
| 90 | Nucleolar exosome (RNAse complex) | 3 | GO:0101019 | 2.84E-02 | EXOSC6, EXOSC10, EXOSC2 |
| 91 | ER membrane protein complex | 3 | GO:0072546 | 2.84E-02 | EMC1, EMC10, EMC8 |
| 92 | Proteasome core complex, alpha-subunit complex | 3 | GO:0019773 | 3.41E-02 | PSMA5, PSMA6, PSMA7 |
| 93 | Intercalated disc | 5 | GO:0014704 | 3.52E-02 | ITGB1, TJP1, DLG1, JUP, CDH2 |
| 94 | Synapse | 23 | GO:0045202 | 3.55E-02 | MPST, USP14, DBNL, CADM1, EIF1AX, RPL23, FMR1, AKR1A1, QKI, ACTB, DNM2, RPS15, SDCBP, RPS28, EIF6, HNRNPF, HNRNPD, CNTN2, CTNNB1, EIF3F, SLC12A7, RPS21, HNRNPA0 |
| 95 | Integrin complex | 4 | GO:0008305 | 3.89E-02 | ITGB1, ITGA7, ITGAV, ITGA6 |
| 96 | Endoplasmic reticulum membrane | 41 | GO:0005789 | 4.01E-02 | SCARB2, LRRC59, MCFD2, AHCYL1, TEX264, UBA5, RAB1B, RTCB, RPN1, TECR, LPCAT1, GNAI3, ATP2A2, EMC10, MIA3, RRBP1, SDCBP, SPTLC1, EMC1, VTI1A, SSR1, MLEC, RAB6A, LBR, SEC11A, OSBPL6, HSPA5, TMED7-TICAM2, ATP6AP2, SEZ6L, DDOST, KTN1, ERP44, DLG1, DHRS7, TMX3, ARMC10, ASPH, CALU, CLPTM1L, SEC22B |
| 97 | Aminoacyl-tRNA synthetase multienzyme complex | 3 | GO:0017101 | 4.02E-02 | AIMP1, EEF1E1-BLOC1S5, EPRS1 |
| 98 | RPAP3/R2TP/prefoldin-like complex | 3 | GO:1990062 | 4.02E-02 | RUVBL1, PFDN2, RPAP3 |
| 99 | Nuclear inclusion body | 3 | GO:0042405 | 4.02E-02 | RANBP2, NXF1, NUP153 |
| 100 | Nuclear exosome (RNAse complex) | 3 | GO:0000176 | 4.02E-02 | EXOSC6, EXOSC10, EXOSC2 |
| 101 | Nuclear pore | 7 | GO:0005643 | 4.19E-02 | RANBP2, NXF1, CHMP4B, NUP153, RAE1, AGFG1, NUP37 |
| 102 | Filamentous actin | 4 | GO:0031941 | 4.26E-02 | PDLIM1, SPECC1, MYO6, CD2AP |
| 103 | U2 snrnp | 4 | GO:0005686 | 4.26E-02 | SF3B2, SF3B6, SNRPB2, SF3B1 |
| 104 | Stress fiber | 6 | GO:0001725 | 4.51E-02 | PDLIM1, LIMA1, TPM3, ACTN1, FLNB, ACTN4 |
| 105 | SWI/SNF complex | 4 | GO:0016514 | 4.64E-02 | SMARCD1, ACTL6A, ACTB, SMARCA4 |
| 106 | Mitochondrial sorting and assembly machinery complex | 3 | GO:0001401 | 4.66E-02 | HSPA9, SAMM50, IMMT |
| 107 | Postsynaptic density, intracellular component | 3 | GO:0099092 | 4.66E-02 | CTNND1, CTNNB1, DNM2 |
| 108 | Apical part of cell | 6 | GO:0045177 | 4.74E-02 | SPECC1, TJP1, PCM1, CDH2, CTNNB1, LGMN |

**Supplementary Table S4:** List of all identified cellular components with their protein count and proteins were analysed by Database for Annotation, Visualization and Integrated Discovery (DAVID) platform with a significant *p*-value ≤ 0.05 of differentially expressed proteins (up-regulated and down-regulated) identified by the high-resolution mass spectrometry (HRMS).
