# Supplementary material for: A protein–miRNA biomic analysis approach to explore neuroprotective potential of nobiletin in human neural progenitor cells (hNPCs)
Source: Front Pharmacol. 2024 Jan 25;15:1343569. doi: 10.3389/fphar.2024.1343569 (PMC10860404; doi:10.3389/fphar.2024.1343569)
Supplement: Supplementary file 9 [file Table6.DOCX]

**Supplementary Table S6**

**A. Up-regulated Proteins**

| **S.No.** | **KEGG term** | **Count** | **KEGG entry no.** | **P-value** | **Proteins (Up-regulated)** |
| --- | --- | --- | --- | --- | --- |
| 1 | Metabolic pathways | 16 | hsa01100 | 2.85E-04 | PIGS, GSTM3, PDXK, ECHS1, MVK, GBE1, AGL, NDUFA10, GATB, UAP1, PYGL, PPOX, THTPA, HMOX1, NUDT16, AASDHPPT |
| 2 | Nucleocytoplasmic transport | 5 | hsa03013 | 7.71E-04 | NUP205, NUP107, NUP188, NUP210, XPOT |
| 3 | Starch and sucrose metabolism | 3 | hsa00500 | 8.55E-03 | AGL, GBE1, PYGL |

**B. Down-regulated Proteins**

| **S.No.** | **KEGG term** | **Count** | **KEGG entry no.** | **P-value** | **Proteins (Down-regulated)** |
| --- | --- | --- | --- | --- | --- |
| 1 | Proteasome | 16 | hsa03050 | 2.83E-11 | PSMD11, PSMD13, PSMA7, PSMD8, PSMA5, PSMB6, PSMA6, PSMB4, PSMC6, PSMD7, PSMB5, PSMD4, PSMC4, PSMB3, PSMC2, PSME1 |
| 2 | Amyotrophic lateral sclerosis | 41 | hsa05014 | 1.63E-10 | DCTN6, PSMD11, PSMD13, DCTN1, KLC1, COX5A, PSMA7, ACTB, PSMD8, PSMB6, NXF1, TUBB6, PSMB4, PSMD7, PSMB5, PSMD4, PSMB3, CASP3, HNRNPA1, RAE1, RANBP2, HNRNPA3, HSPA5, FUS, ALYREF, NUP153, TUBA4A, SOD1, PSMA5, PSMA6, TUBB2B, PSMC6, PSMC4, PSMC2, CAT, UQCRC1, SRSF3, NDUFS1, TARDBP, MCU, NUP37 |
| 3 | Citrate cycle (TCA cycle) | 11 | hsa00020 | 4.89E-08 | ACLY, FH, PC, PDHA1, MDH1, MDH2, IDH1, SUCLG2, SUCLG1, ACO2, PDHB |
| 4 | Parkinson disease | 29 | hsa05012 | 2.74E-07 | PSMD11, PSMD13, GNAI3, KLC1, COX5A, PSMA7, PSMD8, PSMB6, TUBB6, PSMB4, PSMD7, PSMB5, PSMD4, PSMB3, CASP3, PLCG1, HSPA5, TUBA4A, SOD1, PSMA5, PSMA6, TUBB2B, PSMC6, PSMC4, PSMC2, UQCRC1, NDUFS1, SLC25A5, MCU |
| 5 | Huntington disease | 30 | hsa05016 | 1.51E-06 | DCTN6, PSMD11, PSMD13, DCTN1, KLC1, COX5A, PSMA7, PSMD8, PSMB6, TUBB6, PSMB4, PSMD7, PSMB5, PSMD4, POLR2B, PSMB3, CASP3, AP2M1, SOD2, TUBA4A, SOD1, PSMA5, PSMA6, TUBB2B, PSMC6, PSMC4, PSMC2, UQCRC1, NDUFS1, SLC25A5 |
| 6 | Prion disease | 28 | hsa05020 | 1.55E-06 | PSMD11, PSMD13, KLC1, COX5A, PSMA7, PSMD8, PSMB6, TUBB6, PSMB4, PSMD7, PSMB5, PSMD4, PSMB3, CASP3, HSPA5, CSNK2A2, TUBA4A, SOD1, PSMA5, PSMA6, TUBB2B, PSMC6, PSMC4, PSMC2, UQCRC1, NDUFS1, SLC25A5, MCU |
| 7 | Carbon metabolism | 17 | hsa01200 | 3.09E-06 | FH, PDHA1, TPI1, MDH1, GLDC, MDH2, RPE, IDH1, PDHB, PC, TKFC, CAT, PGK1, SUCLG2, SUCLG1, ACO2, GAPDH |
| 8 | Metabolic pathways | 88 | hsa01100 | 3.98E-06 | ACADVL, AHCYL1, GLDC, TECR, MPI, LPCAT1, TXNDC12, NUDT5, GPHN, CNDP2, SPTLC1, NNT, DBT, PGM3, DHRS4L2, SEPHS1, ATP6V1E1, ACAA1, ACP1, LBR, ENOPH1, ACAD8, ABHD14A-ACY1, CBR1, ADSL, TPI1, GAA, AKR1A1, MIF, QRSL1, DDOST, HADHB, ACLY, MTHFD1, IVD, CAT, AGPS, SMS, UQCRC1, SUCLG2, SUCLG1, NDUFS1, HPRT1, UMPS, GAPDH, PAFAH1B2, ISYNA1, ASAH1, FH, GMPR2, RPE, RPN1, PDHB, COX5A, ACACA, NME1-NME2, HMGCL, TKFC, MTHFD1L, MAT2A, PGK1, BPNT2, RDH13, PLCG1, MPST, TIGAR, PDHA1, MDH1, AGK, MDH2, IDH1, GSR, PYCR1, FAH, PRDX6, DHODH, DHFR, ALDH4A1, GRHPR, GALE, PC, EPRS1, GNPDA2, IMPDH2, ECHDC1, ACO2, SCLY, ITPA |
| 9 | Spinocerebellar ataxia | 18 | hsa05017 | 1.33E-05 | PSMD11, PSMD13, ATP2A2, PSMA7, PSMD8, PSMA5, PSMB6, PSMA6, PSMB4, PSMC6, PSMD7, PSMB5, PSMD4, PSMC4, PSMB3, PSMC2, SLC25A5, MCU |
| 10 | Pathways of neurodegeneration - multiple diseases | 36 | hsa05022 | 3.79E-05 | DCTN6, PSMD11, PSMD13, DCTN1, ATP2A2, KLC1, COX5A, PSMA7, PSMD8, PSMB6, TUBB6, PSMB4, PSMD7, PSMB5, PSMD4, PSMB3, CASP3, PLCG1, HSPA5, FUS, CSNK2A2, TUBA4A, SOD1, PSMA5, PSMA6, TUBB2B, PSMC6, PSMC4, PSMC2, CAT, UQCRC1, CTNNB1, NDUFS1, TARDBP, SLC25A5, MCU |
| 11 | Arrhythmogenic right ventricular cardiomyopathy | 12 | hsa05412 | 8.61E-05 | ITGB1, JUP, CDH2, CACNA2D1, LMNA, ATP2A2, CTNNB1, ITGA7, ITGAV, ITGA6, EMD, ACTB |
| 12 | mRNA surveillance pathway | 13 | hsa03015 | 1.71E-04 | DAZAP1, CPSF7, RBM8A, FUS, ALYREF, SRRM1, NXF1, FIP1L1, WDR82, ACIN1, SAP18, RNPS1, TARDBP |
| 13 | Pyruvate metabolism | 9 | hsa00620 | 2.34E-04 | GRHPR, FH, PC, PDHA1, MDH1, MDH2, AKR1A1, PDHB, ACACA |
| 14 | Salmonella infection | 22 | hsa05132 | 2.34E-04 | DCTN6, ACTR3, RAB5B, RAB5C, AHNAK, ROCK2, DCTN1, AHNAK2, KLC1, MYL12A, TUBA4A, ACTB, DNM2, TUBB6, TUBB2B, CASP3, MYO6, CTNNB1, FLNB, GAPDH, SKP1, RAB7A |
| 15 | Alzheimer disease | 29 | hsa05010 | 2.69E-04 | PSMD11, PSMD13, ATP2A2, KLC1, COX5A, PSMA7, PSMD8, PSMB6, TUBB6, PSMB4, PSMD7, PSMB5, PSMD4, PSMB3, CASP3, CSNK2A2, TUBA4A, PSMA5, PSMA6, TUBB2B, PSMC6, PSMC4, PSMC2, UQCRC1, CTNNB1, NDUFS1, SLC25A5, GAPDH, MCU |
| 16 | Biosynthesis of amino acids | 10 | hsa01230 | 1.38E-03 | ABHD14A-ACY1, PC, TPI1, MAT2A, RPE, IDH1, PGK1, PYCR1, ACO2, GAPDH |
| 17 | Adherens junction | 11 | hsa04520 | 1.78E-03 | TJP1, ROCK2, PDCD10, ACTN1, CSNK2A2, CTNND1, CTNNB1, ACTN4, ACP1, MYL12A, ACTB |
| 18 | Peroxisome | 10 | hsa04146 | 2.59E-03 | HMGCL, PEX19, PEX3, IDH1, ECI2, AGPS, CAT, SOD2, ACAA1, SOD1 |
| 19 | Glyoxylate and dicarboxylate metabolism | 6 | hsa00630 | 3.93E-03 | GRHPR, MDH1, GLDC, MDH2, CAT, ACO2 |
| 20 | Hypertrophic cardiomyopathy | 10 | hsa05410 | 4.86E-03 | ITGB1, TPM3, CACNA2D1, LMNA, ATP2A2, ITGA7, ITGAV, ITGA6, EMD, ACTB |
| 21 | Nucleocytoplasmic transport | 11 | hsa03013 | 5.31E-03 | RANBP2, NXF1, RBM8A, ALYREF, ACIN1, SAP18, NUP153, RNPS1, RAE1, SRRM1, NUP37 |
| 22 | Ribosome | 14 | hsa03010 | 7.03E-03 | RPL5, RPL30, RPLP1, RPL23, RPL12, RPSA, RPL10A, MRPL21, RPS15, RPS28, RPLP2, RPS2, RPS21, RPL17 |
| 23 | Dilated cardiomyopathy | 10 | hsa05414 | 7.40E-03 | ITGB1, TPM3, CACNA2D1, LMNA, ATP2A2, ITGA7, ITGAV, ITGA6, EMD, ACTB |
| 24 | DNA replication | 6 | hsa03030 | 8.74E-03 | POLA1, FEN1, MCM7, POLD2, SSBP1, MCM6 |
| 25 | Biosynthesis of cofactors | 13 | hsa01240 | 8.90E-03 | ADSL, MPI, AKR1A1, GPHN, DHODH, NME1-NME2, DHFR, EPRS1, MTHFD1, MTHFD1L, MAT2A, RDH13, UMPS |
| 26 | Cysteine and methionine metabolism | 7 | hsa00270 | 1.04E-02 | MPST, AHCYL1, MAT2A, MDH1, MDH2, SMS, ENOPH1 |
| 27 | Spliceosome | 16 | hsa03040 | 1.14E-02 | SF3B2, RBM25, HNRNPA3, RBM8A, FUS, ALYREF, SF3B6, DDX42, U2SURP, PUF60, ACIN1, SRSF3, SNRPB2, HNRNPC, HNRNPA1, SF3B1 |
| 28 | Leukocyte transendothelial migration | 10 | hsa04670 | 2.12E-02 | ITGB1, ROCK2, ACTN1, CTNND1, GNAI3, CTNNB1, ACTN4, PLCG1, MYL12A, ACTB |
| 29 | Viral life cycle - HIV-1 | 7 | hsa03250 | 2.50E-02 | RANBP2, CPSF7, PSIP1, CHMP4B, PIN1, NELFB, NUP153 |
| 30 | Propanoate metabolism | 5 | hsa00640 | 2.66E-02 | DBT, ECHDC1, SUCLG2, SUCLG1, ACACA |
| 31 | Valine, leucine and isoleucine degradation | 6 | hsa00280 | 2.80E-02 | HADHB, ACAD8, HMGCL, IVD, DBT, ACAA1 |
| 32 | Lipoic acid metabolism | 4 | hsa00785 | 2.90E-02 | PDHA1, GLDC, DBT, PDHB |
| 33 | Cardiac muscle contraction | 8 | hsa04260 | 3.64E-02 | ASPH, TPM3, CACNA2D1, UQCRC1, ATP2A2, ATP1B3, ATP1A1, COX5A |
| 34 | Phagosome | 11 | hsa04145 | 4.68E-02 | ITGB1, TUBB2B, TUBB6, RAB5B, RAB5C, ITGAV, ATP6V1E1, SEC22B, ACTB, TUBA4A, RAB7A |

**Supplementary Table S6:** List of all identified KEGG pathways in differentially expressed proteins (upregulated and downregulated) of NA vs control NPCs analysed by Database for Annotation, Visualization and Integrated Discovery (DAVID) platform with a significant *p*-value ≤ 0.05.
